# Global meta-analysis of short-term associations between ambient temperature and pathogen-specific respiratory infections, 2004 to 2023

**DOI:** 10.2807/1560-7917.ES.2025.30.11.2400375

**Published:** 2025-03-20

**Authors:** Xue Shang, Ruhao Zhang, Junyao Zheng, Yi Luo, Kangle Guo, Qingqing Zhou, Xu Guang, Ning Zhang, Hao Xue, Haidong Wang, Chunfu Yang, Zhen Zhang, Bin Zhu

**Affiliations:** 1 School of Public Health and Emergency Management, Southern University of Science and Technology, Shenzhen, China; 2 China Institute for Urban Governance, Shanghai Jiao Tong University, Shanghai, China; 3 School of International and Public Affairs, Shanghai Jiao Tong University, Shanghai, China; 4 Shanxi Provincial Health Industry Association Service Center, Shaanxi, China; 5 Department of Infection Management, Gansu Provincial Hospital, Gansu, China; 6 Vanke School of Public Health, Tsinghua University, Beijing, China; 7 Stanford Center on China's Economy and Institutions, Stanford University, Stanford, United States; 8 Shenzhen Center for Disease Control and Prevention, Shenzhen, China; * These authors contributed equally to this work and share last authorship.

**Keywords:** bacterial infections, viral infections, respiratory infections, Haemophilus influenzae, human metapneumovirus, influenza virus, Pseudomonas aeruginosa, respiratory syncytial virus, Streptococcus pneumoniae, climate change

## Abstract

**Background:**

Ambient temperature may affect respiratory health, while the temperature sensitivity of respiratory infections may be pathogen-dependent.

**Aims:**

We sought to explore pathogen-specific associations between ambient temperature and respiratory infections.

**Methods:**

We searched nine databases for a random-effects meta-analysis to pool the relative risk (RR) of respiratory infection by pathogen per 1° C temperature rise, compared to populations unexposed to the same temperature. We conducted pathogen-specific analyses, sensitivity analyses, subgroup analyses and meta-regression.

**Results:**

A total of 137 studies were eligible for meta-analysis. The pooled and single-study estimates revealed that the incidence of respiratory syncytial virus (RR = 0.14; 95% confidence interval (CI): 0.09–0.23), influenza virus (IV) (RR = 0.40; 95% CI: 0.27–0.61), human metapneumovirus (RR = 0.48; 95% CI: 0.32–0.73), human coronavirus (HCoV) (RR = 0.21; 95% CI: 0.07–0.61) and SARS-CoV-2 (RR = 0.52; 95% CI: 0.35–0.78) decreased per 1° C temperature rise, while that of human parainfluenza virus (HPIV) (RR = 2.35; 95% CI: 1.46–3.77), human bocavirus (HBoV) (RR = 1.86; 95% CI: 1.04–3.32) and MERS-CoV (RR = 1.05; 95% CI: 1.04–1.07) increased. The risk of infection was lower for IVA, IVB, HCoV-229E and HCoV-OC43, while HPIV-3, and HBoV-1 were at increased risk. The risk of *Streptococcus pyogenes* pharyngitis (RR = 0.46; 95% CI: 0.30–0.69) decreased per 1° C temperature rise, while *Pseudomonas aeruginosa* (RR = 1.04; 95% CI: 1.03–1.05) and *Legionella pneumophila* infections (RR = 2.69; 95% CI: 1.11–6.53) increased.

**Conclusions:**

Temperature sensitivity of respiratory infections can vary with the specific pathogen type and subtype that causes the infection. As the climatic conditions will become warmer, public health policy makers should act to develop pathogen adaptation strategies.

## Introduction

Globally, annual epidemics of respiratory tract infections (RTIs) result in a substantial health burden [1], especially among infants, young children [2] and older adults (≥ 65 years) [3], with an estimated 0.74 million deaths among children (0–59 months) attributable to RTIs in 2019 [4]. These infections were estimated to cause 17.2 billion cases, 2.50 million deaths and 6.4 million disability-adjusted life years globally across all ages in 2019 [5]. Respiratory tract infection involves more than 200 types of viruses, including influenza virus (IV), respiratory syncytial virus (RSV), human rhinovirus (HRV) and coronavirus (CoV) [6-8]. After viral infection, bacterial colonisation may occur, which worsens the disease and prolongs recovery time [8,9]. More recently, the severe acute respiratory syndrome CoV 2 (SARS-CoV-2) pandemic has further expanded the pool of respiratory viruses [10]. Among the estimated 7.7 million deaths from bacterial infection reported globally in 2019, lower respiratory tract infections (4.0 million) were one of the leading syndromes, involving several pathogens such as *Streptococcus pneumoniae* (829,000 cases) and *Pseudomonas aeruginosa* (559,000 cases) which accounted for 54.9% of deaths among the surveyed bacteria [11]. Respiratory infections remain a major global challenge, with many causes and complex factors affecting their occurrence. This necessitates the exploration of the determinants of respiratory diseases.

The transmission dynamics and pathogenicity of microorganisms are influenced by host factors, medical interventions and environmental conditions, with temperature playing a particularly important role. Temperature increase may accelerate the survival, reproduction and mutation of pathogens, increase the probability of pathogen invasion of the host, and affect the incidence of respiratory infections [12]. The developmental cycle of many pathogens depends on temperature, and temperature changes can affect the basic reproductive rate of pathogens or their vectors. Based on a review of large-scale laboratory and epidemiological data, the role of temperature in the efficiency of respiratory virus transmission has been determined previously, and it was discovered that many respiratory infections increase as ambient temperature decreases [13,14].

Diagnostic advances in human respiratory pathogen detection have led to increased availability of data on pathogen-specific respiratory infections, and numerous studies have further explored the correlation between temperature and respiratory pathogen infections. Previous evidence revealed that RSV prevalence was negatively correlated with temperature [2,3,15]. Similar findings have been observed for IV infections, where high temperatures can reduce the replication and spread of the virus [16-21]. Temperature may also affect the immune status of the population and the replication of human parainfluenza virus (HPIV), potentially promoting its transmission [12]. Globally, low temperatures increase the risk of common human CoV (HCoV) infection [22-24]. For the three highly pathogenic coronaviruses that emerged in the 21st century (SARS-CoV-1 and 2 and Middle East respiratory syndrome (MERS)-CoV), some recent evidence has revealed a possible association between SARS-CoV-2 incidence and temperature [25,26]. However, the above evidence has not yet been effectively summarised, and research results on some viruses remain inconsistent [27,28].

Some reviews have qualitatively summarised the broad association between temperature and the spread and incidence of some pathogens [22,29-31]. However, research evidence that systematically quantifies the relationship between temperature and infection with respiratory-specific pathogens is lacking, and the correlation has not yet been fully established. This uncertainty is important in the context of global climate change, which is expected to lead to large fluctuations in weather patterns, potentially altering traditional respiratory disease epidemiology. In this systematic review, we synthesised all the published evidence on the effects of temperature on respiratory tract-specific pathogen infections. We primarily focused on the common pathogens that cause respiratory infections globally, specifically analysing the effects of different viruses, bacteria, and pathogen subtypes to help determine which pathogens are likely to primarily infect humans in the future.

## Methods

### Study design and participants

The systematic review and meta-analysis followed the Preferred Reporting Items for Systematic Reviews and Meta-Analysis (PRISMA) guidelines [32] (the checklist is appended in the Supplement) and has been registered on PROSPERO (CRD42023466911). Following expert consultation, we narrowed the scope of the initially registered protocol by focusing specifically on temperature rather than multiple meteorological factors. We adopted the modified version of the United States (US) Office of Health Assessment and Translation (OHAT) approach specifically for evidence integration in environmental health issues [33].

We devised the population-exposure-comparators-outcomes-study design (PECOs) framework as follows: assessing the risk of respiratory diseases in the general population (P) per 1° C rise in temperature (E), compared with populations not exposed to the same temperature (C), on the risk of morbidity due to pathogen-specific respiratory infections (O) in observational studies (s). We defined pathogen-specific respiratory infections listed in the International Classification of Diseases 11 Clinical Modification CA40-42 codes.

### Search strategy and selection criteria

The complete search strategy was originally developed by XS and BZ and finalised in consultation with the information specialists of our library. We searched PubMed, Embase, Scopus, Web of Science, Cochrane, China National Knowledge Infrastructure, Wanfang Data, Weipu Database and China Biology Medicine for studies published from database origin up to 10 September 2023. In addition, we manually searched for relevant literature through forward and backward citation tracing using CoCites to obtain the remaining studies [34]. We used the search strategy as outlined in the Box. A full list of the search terms is appended in the Supplement. Retrieved records were imported into Zotero.

Box **Search strategy of systematic review about associations between temperature and respiratory infections, 2004–2023**

(‘temperature’ OR ‘climate’ OR ‘weather’ OR ‘global warming’ OR ‘meteorolog*’ ) AND (‘respiratory syncytial virus’ OR ‘adenovirus’ OR ‘influenza virus’ OR ‘parainfluenza virus’ OR ‘metapneumovirus’ OR ‘rhinovirus’ OR ‘coronavirus’ OR ‘SARS-CoV’ OR ‘SARS-CoV-2’ OR ‘MERS-CoV’ OR ‘HCoV’ OR ‘bocavirus’ OR ‘enterovirus’ OR ‘*Mycoplasma pneumoniae*’ OR ‘*Chlamydophila pneumoniae*’ OR ‘*Streptococcus pneumoniae*’ OR ‘*Legionella pneumophila*’ OR ‘*Pseudomonas aeruginosa*’ OR *‘Haemophilus influenzae*’ OR ‘*Moraxella catarrhalis*’ OR ‘*Streptococcus pyogenes* pharyngitis’).

### Eligibility

Specifically, we identified studies that explored the effect of the relationship between temperature and morbidity for respiratory infections based on the World Health Organization (WHO) standard case definition of acute respiratory infections (ARIs) [35]. The studies met the following criteria: included the general population of all ages; investigated mean, maximum or minimum temperature as the exposure of interest; employed observational study designs such as case–control, cohort, cross-sectional, case-crossover, time series and surveillance studies to compare risk across exposures or time periods. Articles in English, Chinese, Korean and Spanish were eligible for review. We used translation software (Google Translate and DeepL Pro) to translate Korean and Spanish into English for reading and analysis. Reviews, conference abstracts, editorials, commentaries, protocols, duplicate publications, overlapping studies or studies on non-human outcomes (e.g. animal diseases) were excluded.

### Data extraction

Two reviewers (XS, GL) independently extracted the following details: (i) basic characteristics of the included studies (first author, publication year, location, study period); (ii) the main information of population (age, sex, subpopulation); (iii) the characteristics of temperature exposure (temperature range, measurement, temporal resolution); (iv) details of study design and analysis (time sample size, study type, statistical method, modelling approach, temporal lags); (v) case sources (types of respiratory events, hospital cases, reported cases etc.) and (vi) population vulnerability. Measures of vulnerability were based primarily on national income levels and climate classifications. Based on the World Bank Economic Classification criteria, the studies were classified and described by national income for each country [36]. We used the Köppen–Geiger climate classification map to identify the major climate categories for each study (A-tropical, B-arid, C-temperate, D-continental and E-polar) [37]. We classified five studies that pooled data from multiple regions of a country as temperate because most of the sites included in these studies were located in temperate zones [38-42].

We extracted the relative risk (RR) or incidence rate ratio (IRR) and 95% confidence intervals (CIs) for respiratory infections at increased temperatures provided in the study. If the RR/IRR was not provided in the article, we extracted the reported percentage changes, odds ratio (OR), excess risk (ER), hazard ratio (HR), correlation coefficients (CORs), beta coefficients (β), standard errors (SEs) or CIs and converted that into RR; the conversion formula is appended in the Supplement. When calculations were not possible, we made an effort to reach out to the original authors. If calculations remained impossible, we excluded the study. A third author (HX) checked 30% of the extracted data and found no disagreement. Other co-authors validated the accuracy of the remaining studies.

### Quality and strength of evidence

In line with the Navigation Guidelines Framework [33], we systematically assessed the quality and strength of the evidence by (i) assessing the risk of bias in individual studies, (ii) rating the quality of the evidence across all studies, and (iii) rating the strength, or certainty, of the evidence across all studies.

To evaluate the influence of study quality on combined effects, we collaborated with subject experts (BZ, KG, CY) to adapt the assessment criteria, based on the bias risk rating tool from the OHAT [33] and the improved framework by Rhiannon et al. [43], to better fit our research question. Our tool included eight domains separately examining exposure assessment, outcome assessment, confounding bias, selection bias, incomplete outcome data, selective reporting, conflict of interest and other biases. Each domain is divided into four levels: definitely high, probably high, probably low and definitely low; for further detail see Supplementary Table S5.

The certainty of evidence was assessed using the OHAT approach. Following the guide, human evidence was pre-specified as ‘moderate quality’ and could be upgraded or downgraded depending on eight factors. It was mainly downgraded from the risk of bias/internal validity, indirectness, unexplained inconsistency, imprecision and publication bias, and upgraded from large magnitude of effect and dose-response; confounding minimised the effect. The final level of evidence was rated as very low, low, moderate or high.

We determined the overall strength of the evidence for temperature episodes taking into consideration the following four factors: (i) quality of the body of evidence (previous step rating), (ii) direction of effect size, (iii) confidence in the effect (a new study would change conclusions) and (iv) other attributes of the data that might affect certainty. The overall strength of the evidence can be divided into: ‘sufficient’, ‘limited’, ‘inadequate’ or ‘lack of evidence’. All evidence assessment details are appended in the Supplement.

### Meta-analysis

We conducted random effects meta-analyses using inverse variance and restricted maximum-likelihood method to address heterogeneity variance [44,45], pooling effect estimates when studies directly presented RR estimates or converted RRs (such as percentage change, ER, β or SEs). Before pooling, we assessed methodological similarities among included studies. For pathogens with limited studies, we verified consistency before pooling. We calculated the pooled RR using country-specific, region-specific or city-specific effect estimates. The pooled estimates were the RR and 95% CI of a 1° C temperature increase associated with the incidence of respiratory infections. Statistical significance was determined using Z-test, with p < 0.05 considered statistically significant. For each pooled estimate, we considered results significant when the 95% CI did not include 1. For multi-country and multi-site studies, the RR was determined based on pooled site-specific estimates, if applicable. When reporting multiple estimates, following the Cochrane collaboration guidelines, the RR was selected from the final model defined by the authors or the model with the greatest relevant covariates, including mean, minimum and maximum temperature. We extracted RR corresponding to different temperature ranges in the study for meta-analysis. For both cold and heat effects reported in the distributed hysteretic non-linear model, we chose RR for heat effects. If there were multiple lag effects presented in studies, we extacted the lag associated with the greatest risk among single or cumulative lags. When given percentile-based RR estimates, we documented the percentiles, temperature points and corresponding RR values. If RRs from different models (linear and non-linear) were provided in a study, they were both included in the pooled analysis. When adjusted and crude effects were provided, the adjusted effect was chosen. For multiple studies conducted by the same research group, if their study periods overlapped, we prioritised studies with longer duration, broader age group inclusion, and wider geographical scope when synthesising effects. 

### Subgroup analysis

Heterogeneity was assessed by Cochrane Q (p < 0.10 is the significance level) and Higgins I^2^ tests. When p < 0.05, I^2^ > 50 indicates substantial heterogeneity [46]. We performed subgroup analyses for each taxon of respiratory infections to explore differences between pathogen-specific estimates. In addition, we conducted the subgroup analysis by virus subtype, climate zone, national income level, temporal resolution (daily, weekly or monthly), temperature measurement (mean, min or max), modelling method (linear and non-linear) and lag effect.

### Sensitivity analysis

Sensitivity analyses were considered to evaluate the stability of the pooled effects. Leave-one-out analysis was performed to assess the impact of individual estimates on the pooled RR. Studies with a high risk of bias for risk of bias ratings were eliminated to further reduce bias. For studies exhibiting both hot and cold effects, the thermal effect was converted to a cold effect.

### Meta-regression

Meta regression explores the influence of multiple study features on the variance of the combined estimates [47]. We used multivariate meta-regression to model the adjusted association between multiple explanatory variables and incidence estimates. We analysed four potentially influential modifiers: income level, WHO region, study design and age [46,47].

Publication bias was evaluated by a contour-enhanced funnel plot and Egger linear regression test (p < 0.05). The trim-and-fill method accounts for potential publication bias by estimating the number of studies missed due to publication bias and adjusting the overall estimate.

We used ArcGIS10.8 (Environment Systems Research Institute, RedLands City, US) to add coordinates for each study's specific location. R version 4.3.2 (R Core Team, Vienna, Austria) was used for statistical analysis and meta, metaphor, robumeta and forestploter packages for data analysis and meta-analysis. The *metareg* command in Stata 15.1 (Stata Corp, College Station, US) was used to perform meta-regression analysis.

## Results

**Figure 1:**
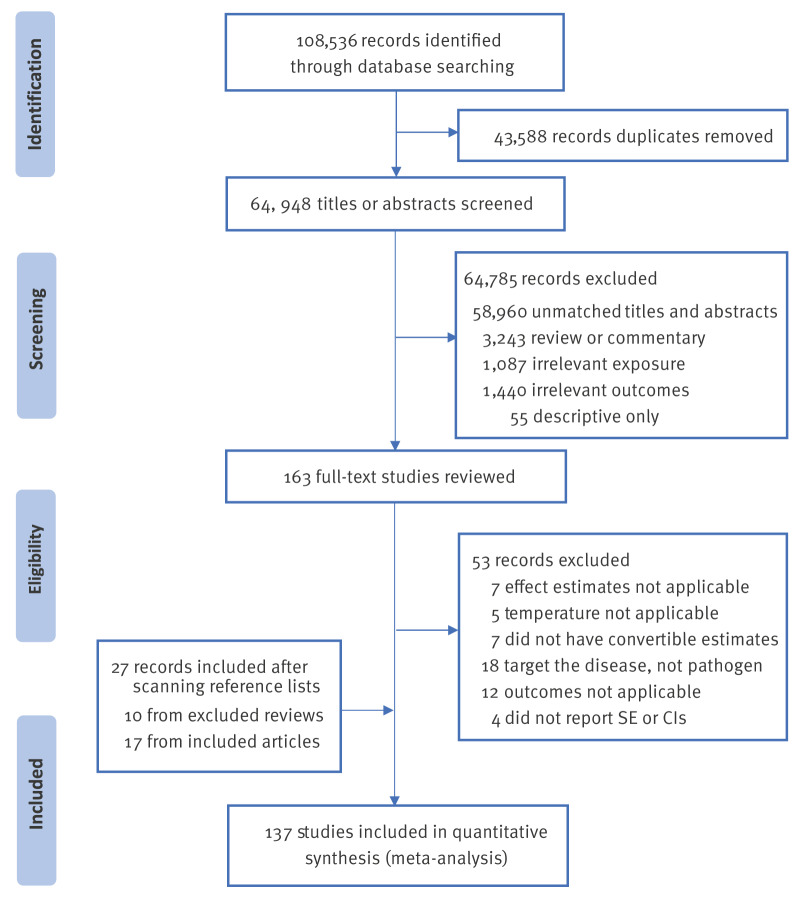
Flowchart of assessment of eligible studies, meta-analysis of associations between temperature and respiratory infections, 2004–2023 (n = 108,536)

We retrieved a total of 108,536 records, of which we removed 42,508 duplicates. By screening 64,948 titles and abstracts, 163 studies were identified in the full text review, and 110 studies ultimately met the inclusion criteria. Details of excluded articles are appended in the Supplement. In addition, we selected 27 studies for the meta-analysis by examining the reference lists (Figure 1).

The PRISMA flow diagram of the search and selection of studies included in this meta-analysis.

**Figure 2:**
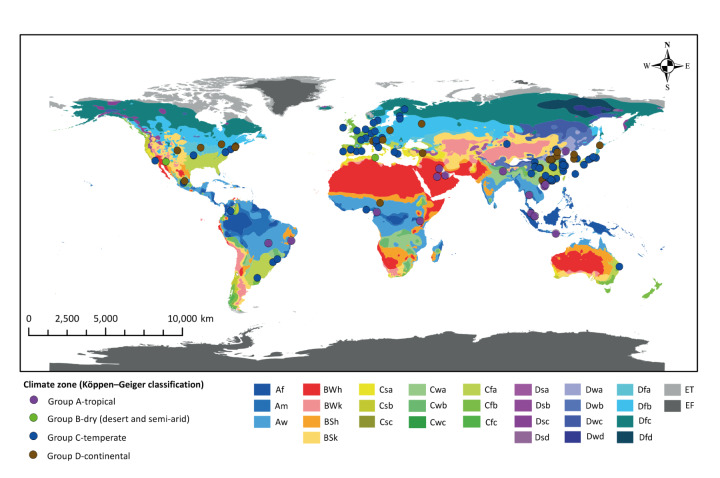
Geographical distribution of the 225 city- or region-specific pathogen-specific respiratory infection estimates included in the meta-analysis, by climate zone, 2004–2023 (n = 7.17 million)

The general characteristics of the included studies are presented in Supplementary Table S9. The publications included in this review were published between 2004 and 2023. Study locations varied and included 225 estimates specific to cities or regions (Figure 2). Additional information on geographical distribution is appended in Supplement. In total, these studies included an estimated 7.17 million respiratory infection events, 744,591 of which involved infant and child patients. The analysis periods across the included studies ranged from a few months to 21 years. The geographical distribution of the included studies spanned four Köppen–Geiger climate zones: tropical (15.94%), dry (0.01%), temperate (marine, Mediterranean, and subtropical; 64.49%) and continental (13.76%).

Af: tropical rainforest climate; Am: tropical monsoon climate; Aw: tropical savanna climate; BWh: hot desert climate; BWk: cold desert climate; BSh: hot semi-arid climate; BSk: cold semi-arid climate; Csa: hot-summer Mediterranean climate; Csb: warm-summer Mediterranean climate; Csc: cold-summer Mediterranean climate; Cwa: monsoon-influenced humid subtropical climate; Cwb: subtropical highland climate or Monsoon-influenced temperate oceanic climate; Cwc: cold subtropical highland climate or Monsoon-influenced subpolar oceanic climate; Cfa: humid subtropical climate; Cfb: temperate oceanic climate; Cfc: subpolar oceanic climate; Dsa: Mediterranean-influenced hot-summer humid continental climate; Dsb: Mediterranean-influenced warm-summer humid continental climate; Dsc: Mediterranean-influenced subarctic climate; Dsd: Mediterranean-influenced extremely cold subarctic climate; Dwa: Monsoon-influenced hot-summer humid continental climate; Dwb: Monsoon-influenced warm-summer humid continental climate; Dwc: Monsoon-influenced subarctic climate; Dwd: Monsoon-influenced extremely cold subarctic climate; Dfa: hot-summer humid continental climate; Dfb: warm-summer humid continental climate; Dfc: subarctic climate: Dfd: extremely cold subarctic climate; ET: tundra climate; EF: ice cap climate.

The map was created with the help of the source in [37].

Of the 137 studies that investigated specific pathogens, 55 (40%) were conducted in high-income countries, 76 (55%) in upper-middle-income countries, four (3%) in lower-middle-income countries, one (1%) in a low-income country. Nine viral infections were involved, namely RSV, IV, HPIV, HRV, human metapneumovirus (HMPV), CoV (SARS-CoV, MERS-CoV, SARS-CoV-2, HCoV, HBoV, human adenovirus (HAdV) and enterovirus (EV), most commonly reported was RSV (62 studies; 45%). Two studies examined both HRV and EV (HRV/EV). Eight bacterial infections were also reported, namely *S. pneumoniae, S. pyogenes*, *M. catarrhalis*, *P. aeruginosa*, *L. pneumophila*, *H. influenzae*, *C. pneumoniae* and *M. pneumoniae*, among which *M. pneumoniae* was the most frequently reported (19 studies; 14%). For statistical analysis, 84 (61%) of the studies used linear models and 53 (39%) used non-linear models.

**Figure 3:**
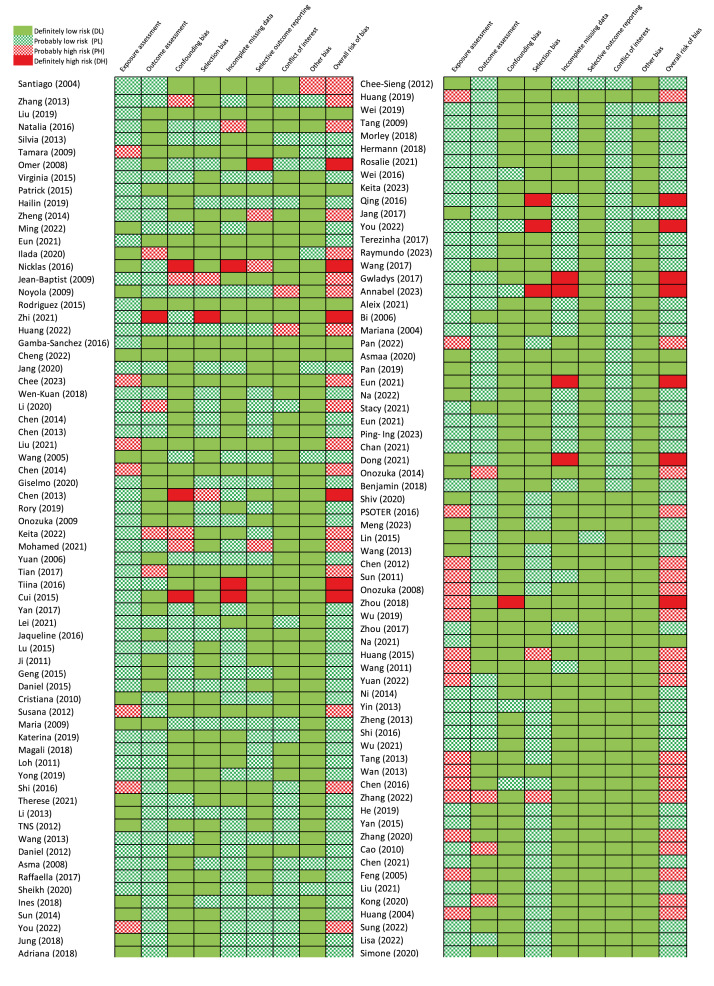
Overview of Navigation Guide systematic review methodology used for rating the quality and strength of the human evidence, meta-analysis of associations between temperature and respiratory infections, 2004–2023 (n = 137 studies)

CI: confidence interval; SE: standard error.

Most studies that reported pathogen-specific estimates were rated as having a definitely low or probably low risk of bias (Figure 3). Additional evaluation information is appended in the Supplement. Thirteen studies had definitely high bias risk scores, with differences in the high-risk domains of each study. For specific domains of evidence, seven studies had definitely high risk of bias in missing data; the authors did not explain whether it affected the results and did not use appropriate methods to adjust. Twenty-five studies had probably high risk of bias in exposure measures because the data in these studies were from a single meteorological station or meteorological base grid. The authors did not provide further information on the monthly resolution. Nine studies had a definitely high or probably high risk of bias in outcome assessment because of cases aggregated from published articles or no description of laboratory confirmation. Eight studies had a definitely high or probably high risk of confounding bias because they did not account for time-varying confounding factors that control, such as trends or seasonality. Eight studies had definitely high or probably high risk of selection bias, because they used data from published studies selected through database search. Four studies had a definitely high or probably high risk of selective outcome reporting bias because they reported only some specific pathogens and temperature-associated risks, while others were not provided for, without further explanation.

The picture shows three parts of evidence evaluation, namely risk of bias, quality of evidence, and strength of evidence, respectively showing the subdomains and determinations/ratings of evaluation. Navigation Guidelines Framework [33]. The graded colours from red to green represent the severity of the risk of bias, with the redder the colour, the higher the risk of bias.

### Meta-analysis and single-study findings

**Figure 4:**
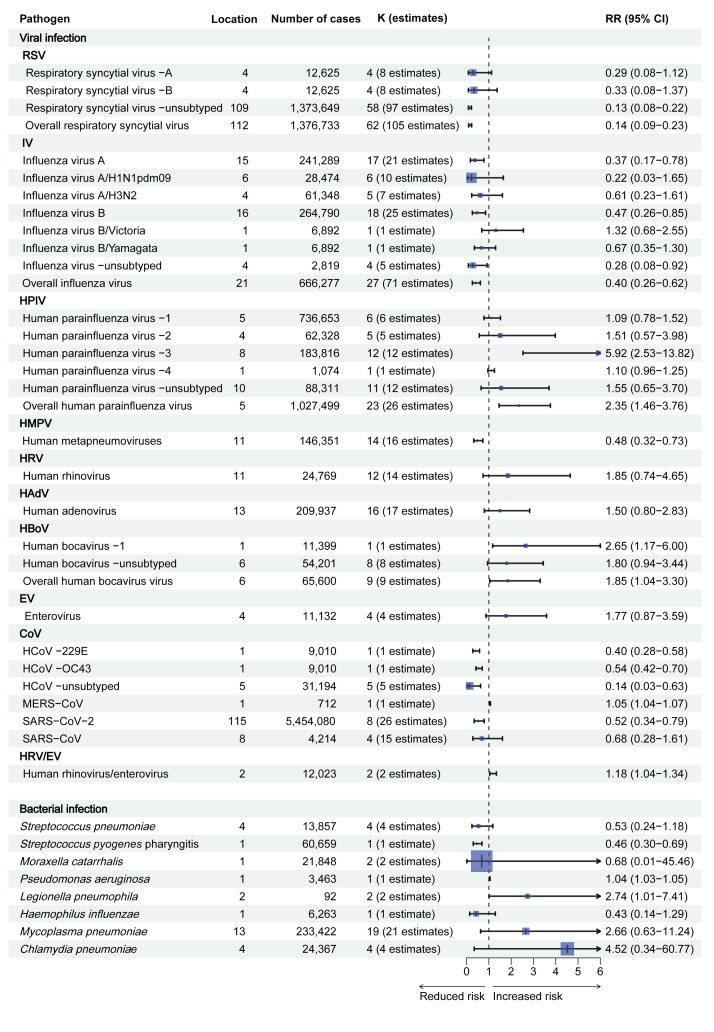
Weighted bar plots indicating the percentage of risk of bias judgements within each bias domain across reviewed studies of high temperatures and pathogen-specific respiratory infection, 2004–2023 (n = 137 studies)

CI: confidence interval; CoV: coronavirus; EV: enterovirus; HAdV: human adenovirus; HBoV: human bocavirus; HCoV: human coronavirus; HMPV: human metapneumovirus; HPIV: human parainfluenza virus; HRV: human rhinovirus; IV: influenza virus; MERS: Middle East respiratory syndrome; RSV: respiratory syncytial virus; SARS: severe acute respiratory syndrome.

We performed a random effects meta-analyses for pathogens with data from multiple studies, while findings from pathogens with only single studies are included individually. The pooled and single-study estimates of pathogen-specific RRs revealed that the overall risk of viral respiratory infection incidence decreased per 1° C rise in temperature by −86% (RR = 0.14; 95% CI: 0.09–0.23; 62 studies) for RSV, −60% (RR = 0.40; 95% CI: 0.27–0.61; 27 studies) for IV, −52% (RR = 0.48; 95% CI: 0.32–0.73; 14 studies) for HMPV, −79% (RR = 0.21; 95% CI: 0.07–0.61; 14 studies) for HCoV and −48% (RR = 0.52; 95% CI: 0.35–0.78; five studies) for SARS-CoV-2. However, increased risks per 1° C temperature rise were observed for HPIV (RR = 2.35; 95% CI: 1.46–3.77; 23 studies), HBoV (RR = 1.86; 95% CI 1.04–3.32; nine studies), MERS-CoV (RR 1.05; 95% CI: 1.04–1.07; one study) and HRV/EV (RR = 1.18; 95% CI: 1.04–1.34; two studies). No effect was observed in the risk of HRV (RR = 1.85; 95% CI: 0.74–4.65; 14 studies), HAdVs (RR = 1.50; 95% CI: 0.78–2.87; 16 studies), SARS-CoV (RR = 0.71; 95% CI: 0.34–1.48; four studies) and EV (RR = 1.78; 95% CI: 0.86–3.69; four studies) per 1° C rise in temperature (Figure 4). The calulcation process for each type of pathogen can be found in Supplementary Table S12.

Location indicates the number of geographical locations where studies were conducted. K(estimates) shows how many effect estimates were pooled, with values in parentheses indicating the total available estimates (for example, a study containing the maximum, minimum and average temperature values) from the original studies. The individual references included for each pathogen are appended in upplementary Tabel S12.

For specific viral subtype analysis (Figure 4), decreased risks per 1° C temperature rise were observed for IVA (RR = 0.43; 95% CI: 0.20–0.93), IVB (RR = 0.47; 95% CI: 0.26–0.85), IV-unsubtyped (RR = 0.28; 95% CI: 0.09–0.88), HCoV-229E (RR = 0.40; 95% CI: 0.28–0.58), HCoV-OC43 (RR = 0.54; 95% CI: 0.42–0.70) and HCoV-unsubtyped (RR = 0.14; 95% CI: 0.03–0.63). Conversely, increased risks were observed for HPIV-3 (RR = 5.92; 95% CI: 2.48–14.13) and HBoV-1 (RR = 2.65; 95% CI: 1.17–6.00). However, no statistically significant difference was observed in the risk of RSV-A, RSV-B, IVA/H3N2, IVA/H1N1pdm09, IVB/Yamagata, IVB/Victoria, HPIV-1, HPIV-2, HPIV-4 and HPIV-unsubtyped (p > 0.05).

For bacterial respiratory infections (Figure 4), pooled estimates of RRs revealed that per 1° C increase in temperature, the overall risk of *S.* *pyogenes* pharyingitis was decreased by −54% (RR = 0.46; 95% CI: 0.30–0.69; one study). However, increased risks per 1° C temperature rise were observed for *P. aeruginosa* (RR = 1.04; 95% CI: 1.03–1.05; one study) and *L. pneumophila* infections (RR = 2.69; 95% CI: 1.11–6.53; two studies). No effect was observed for infections with *H. influenzae, M. catarrhalis, S.pneumoniae, M. pneumoniae* and *C. pneumonia* (p > 0.05). The specific results of each pathogen-specific meta-analysis can be found in the Supplement.

### Subgroup analysis

We performed subgroup analyses based on pooled estimates of population vulnerability (climate, national income) and study design (time resolution, measurements, lag types, models) for RSV, IV, HPIV, HMPV, HRV, HAdV, HBoV, SARS-CoV-2, SARS-CoV, HCoV, EV, HRV/EV, *S. pneumoniae*, *M. pneumoniae* and *C. pneumonia*. All details about subgroup analysis are appended in the Supplement.

For climate subgroup analysis, the risk of RSV, IV and *S. pneumoniae* was relatively low in the tropical regions, while the risk of *M. pneumoniae* was relatively high. Only RSV exhibited a relatively lower risk in arid regions. In temperate regions, the risk of RSV, IV, HMPV and SARS-CoV-2 was relatively low, while the risk of HPIV and *M. pneumoniae* was relatively high. The risk of IV and SARS-CoV-2 in the continental region was relatively lower , while the risk of EV was relatively high. 

For income subgroup analysis, temperature was associated with a lower risk of RSV, IV, SARS-CoV-2 and HCoV in studies in areas with high-income levels. In upper-middle-income countries, the heterogeneity was statistically significant (I^2^: 97–99%; p < 0.05), and temperature was associated with a lower risk of RSV, IV, HMPV and *S. pneumoniae* and a higher risk of HPIV, HAdV, HBoV and EV. The risk of HPIV was relatively high in low-income countries. However, the risk was not obvious in lower-middle-income countries. 

For exposure time resolution, temperature measure and model subgroup analysis, only RSV exhibited differences between subgroups, and heterogeneity was still large (I^2^ = 99% across all subgroups). Infections with HMPV and SARS-CoV-2 differed in the subgroups of temperature measurement and model analysis, respectively. Differences were inconsistent for cumulative or single-lag subgroup analyses. The remaining pathogens were unevenly distributed across time resolution, temperature measurements, models and hysteresis subgroups.

### Sensitivity analysis

Leave-one-out analysis revealed that excluding any single study generally did not alter the pooled effect estimates or heterogeneity substantially, suggesting that the results were robust, with the exception of HRV/EV and most bacteria, which were only covered in one or two studies. We performed additional sensitivity analyses for IVs to study the effects of heat and cold, and converting RR from thermal to cold effects did not change the aggregated estimates and heterogeneity of IVs. When we excluded studies with a high risk of bias, this did not result in important changes in overall effect estimates and heterogeneity for most pathogens. However, omitting one anomalous study each for EV [48] and *C. pneumoniae* [49] reduced heterogeneity for these pathogens significantly, and the effect estimates for EV were statistically significant. All details about the sensitivity analysis are appended in the Supplement. 

### Meta-regression

Meta-regression analysis indicated that age did not modify the effect estimates for any individual pathogens in a statistically significant manner. For HPIV, HAdVs, EV, SARS-CoV-2, *S. pneumoniae* and *C. pneumonia*, income levels showed some variation, especially in high-income countries. With the exception of HRV and *M. pneumoniae*, the study design type had a considerable impact on the estimates for remaining pathogens. In the regional distribution, there were notable differences in estimates for IV and SARS-CoV-2 in Europe compared to other regions. Details of all regression results are attached in the Supplement. We performed funnel plot asymmetric estimation and publication bias test for RSV, IV, HPIV, HMPV, HRV, HAdVs and *M. pneumoniae*, and these included at least 10 studies. In funnel plots and Egger’s test, the effects of RSV, IV and HMPV were estimated to have funnel plot asymmetry, which may have potential publication bias. We used the trim-and-fill method to adjust the efficacy estimates for RSV, IV, HMPV and HRV, and the differences were not notable. All details about publication bias are appended in the Supplement.

### Quality and strength of evidence

Evidence quality assessment revealed moderate quality evidence in RSV, IV, HPIV, HBoV, HCoV, MERS-CoV, HRV/EV and *P. aeruginosa* infections, low-quality evidence in HMPV, EV, SARS-CoV, *M. pneumoniae*, *S. pyogenes*, *L. pneumoniae* and *H. influenzae,* and very low-quality evidence in HAdV, HRV, *C. pneumonia*, *S. pneumoniae* and *M. catarrhalis* infections*.* This was mainly due to the large risk of bias in some studies and the wide CIs of the meta-analysis results, which led to overall reduced quality of ambient temperature evidence. However, because most studies reported similar dose–response patterns, with incidence increasing or decreasing with increasing temperature, the quality of evidence eventually improved.

Comparing the above considerations with the definitions in the Navigation Guidelines, we conclude that there is sufficient evidence to indicate that ambient temperature affects the incidence of viral respiratory infections in humans, while evidence for the association of *Mycoplasma*, *Chlamydia* and bacterial respiratory infections is limited. Details of the quality and strength of the evidence are appended in the Supplement.

## Discussion

The influence of temperature on the risk of respiratory infection is multifactorial, involving biological, laboratory, and epidemiological factors, population behaviour and the interaction of environmental factors. Temperature influences the stability and transmission of respiratory viruses through effects on structural components (e.g. lipid membranes, surface proteins) and functional processes (e.g. replication enzymes). As enveloped viruses, IV [48,50], RSV [30,51], HMPV [48,52] and SARS-CoV-2 [53] survive longer at lower temperatures, and increasing temperature may increase lipid mobility, destroy envelope integrity and lead to decreased infectivity. However, non-enveloped viruses (HRV, HAdVs and EV) remain resistant to temperature fluctuations [48,54-56]. Viral adaptations to respiratory tract temperature gradients may further influence tissue tropism and seasonal transmission patterns. Animal experiments further support temperature’s role in transmission [57-59]. A model of IV in guinea pigs confirmed that aerosol transmission of IV is dependent on temperature. Experiments conducted at 5° C, 20° C and 30° C showed that both cold and dry conditions are conducive to transmission [57]. Such experimental evidence aligns with epidemiological trends, reinforcing the link between environmental conditions and real-world transmission risks.

For bacterial pathogens, *P. aeruginosa* and *L. pneumophila*, the optimal growth temperature range is usually between 25–42° C, and an increase in temperature provides a more suitable growth environment for these bacteria, accelerating growth and reproduction [60-63]. At the right temperature (37° C), it is easier to form a stable biofilm, which protects bacteria from the host immune system and antibiotics, and increases their colonisation ability and survival rate in the host body. At the same time, temperature increase may also affect the gene expression of *P. aeruginosa* and *L.pneumophila,* and produce more virulence factors, resulting in increased invasiveness and pathogenicity [64]. In contrast, *S. pyogenes* exhibits temperature- dependent gene expression patterns affecting secreted proteins and haemolysins, suggesting adaptive responses during infection [65]. For some bacteria (e.g. *S. pneumoniae, M. catarrhalis*) there may be a stronger adaptation to the host environment, and their metabolic characteristics and survival strategies may remain stable over a wider range of temperatures [66,67].

The prevalence of most respiratory pathogens follows a seasonal pattern. Among them, RSV, HMPV, IV, SARS-CoV-2 predominantly peak in colder winter months [13,68]. In contrast, *S. pyogenes* and *P. aeruginosa* exhibit divergent seasonal patterns, and warmer weather potentially enhances their environmental persistence or transmission efficiency. Similarly, high temperatures in summer are more conducive to the propagation and spread of *L. pneumophila*. Middle East respiratory syndrome virus is also more prevalent in hot climates [69]. Parainfluenza virus showed type-specific seasonality, with PIV-3 uniquely peaking in summer [70,71], while HRV, HAdVs and EV exist almost all year round [48,54-56,72,73]. The seasonal patterns of these pathogens are not absolute, and the climate in different geographical regions has different effects on viral activity. Our study further highlights regional climatic effects. For example, the correlation between temperature rise and HPIV infection incidence was positive in temperate regions, while HMPV showed a negative correlation, and *S. pneumoniae* was negatively correlated with temperature only in tropical regions. The incidence of these virus infections may be related to the low temperature and low humidity environment in temperate regions and to the rainy environment in tropical regions. The complexity of the interaction of multiple meteorological factors makes forecasting and intervention challenging.

Temperature may also have a direct impact on the the innate and adaptive immune responses of the host [74], or an indirect one virus transmission through climate-dependent changes in population behavior. When the weather gets cold, inhaling cold, dry air impairs mucociliary clearance function, making it easier for IV to invade [75]. However, long-term exposure to high temperature may cause the host to develop immune tolerance to *L. pneumophila*, reduce the ability to clear bacteria, and provide a favorable environment for bacterial growth [1,2]. During the winter, people tend to move indoors in closed environments, and overcrowding increases the chances of virus spread. In addition, ventilation, the use of air conditioning and heating are modifying factors affecting the spread of respiratory viruses.

Temperature will directly affect the sedimentation and evaporation process of droplets and aerosols, and determine the suspension time and transmission distance of viruses in the air [76]. Low temperature and low relative humidity (RH) are generally associated with increased viral infectivity [77], as demonstrated in studies on IVs which showed greater transmission efficiency at 5 °C compared with 20 °C or 30 °C [57], and MERS-CoV [69] which exhibited greater stability at 20 °C with 40% RH compared with higher temperature conditions. In densely populated settings such as schools, person-to-person contact is more frequent, and bacteria are more easily transmitted through direct contact (e.g. shaking hands) or indirect contact (e.g. contaminated surfaces). Recent animal tests have found that fine atmospheric particles such as PM_2.5_ can absorb and carry a variety of disease-causing microorganisms, breaking through the air-blood barrier in the lung and causing serious infection [78].

Vaccination remains a cornerstone public health intervention for respiratory infection control, and may increase in importance if epidemiological patterns shift as a reaction to climate change. According to the WHO, vaccines prevent 3.5-5 million deaths annually from various infectious diseases [79]. Specifically for influenza, vaccines reduced childhood influenza-related hospitalisation risk by 53.3% (68.9% for inactivated vaccines), with efficacy varying significantly between subtypes [80]. Pneumococcal vaccines such as PCV13 [81] and PPSV23 [82] are recommended for all children younger than 5 years and all adults 50 years or older in the US [83]. COVID-19 vaccines have been rolled out globally since 2020, followed by important reductions in SARS-CoV-2 infection-related mortality [84]. In 2023, the US Food and Drug Administration approved the first RSV vaccine for people 60 years and older [85], In addition, breakthroughs have been made for RSV prevention in children, with the long-acting monoclonal antibody nirsevimab approved in the European Union for infants, and maternal RSV vaccines showing promising results in protecting newborns [86]. Some HAdV vector-based vaccines are available, but their real-world implementation remains limited due to challenges in scalability, cost or infrastructure support. Vaccines against *H. influenzae*, particularly against type b (Hib), have been recommended for childhood immuniation programmes and are being used in many countries around the world [87]. Vaccines against other viruses (e.g. HRV, HMPV) and bacteria (e.g. *P. aeruginosa, L. pneumophila*) are still under development. Personal and public health measures (such as hand hygiene, mask wearing, good sanitation) play a key role in controlling the virus spread [88].

In subgroup analyses across climate regions, national income, temporal resolution, measurement, model, and lag period, we observed considerable differences, and substantial heterogeneity persisted. Sensitivity analyses comfirmed the robustness of the pooled estimates. Further meta-regression analysis found that the age difference was not obvious, and the research results were greatly affected by income level, study region and study design. The pathogens HPIV, HAdVs, SARS-CoV-2, *S. pneumoniae and M. pneumoniae* were particularly prominent in high-income countries, possibly because high-income countries often have better diagnostic tools and treatments. The effect of study design type was remarkable, possibly because the studies included in the analysis were inconsistent in identifying and adjusting for confounding factors (such as RH, wind speed, population density), which can also affect virus transmission. In certain studies without RR effect estimates for temperature and pathogens, we incorporated OR values for synthesis to ensure that the evidence was as comprehensive as possible, which may also explain the large heterogeneity. Compared with the US, the Western Pacific and other regions, the risk of IV and SARS-CoV-2 transmission was lower in Europe. This disparity may be attributed to Europe’s robust public health infrastructure and seasonal intervention strategies targeting respiratory pathogens. For instance, many European countries established hospital surveillance networks during the COVID-19 pandemic, enabling real-time tracking of SARS-CoV-2 variants and rapid implementation of containment measures, including targeted testing and quarantine protocols [89]. Concurrent seasonal interventions in Europe, particularly vaccination campaigns [90,91], along with temporary measures such as ventilation requirements in public spaces [92], informed by predictive outbreak modelling [93], have contributed to respiratory infection control efforts. However, climate-driven shifts in temperature regimes may extend the transmission seasons of pathogens while expanding their geographical ranges, necessitating proactive surveillance systems and dynamic public health frameworks to counter emerging diseases.

Based on the funnel plots and Egger’s test, evidence suggests publication bias in RSV, IV and HMPV estimates. This concern is particularly true for the estimated set of reports included, possibly because studies that find no substantial effect of temperature changes on pathogen behavior may not be published as frequently, resulting in an overrepresentation of studies showing notable results.

Nevertheless, our study identified temperature-sensitive and more susceptible respiratory pathogens, and these findings improve our understanding of how temperature may affect the distribution of respiratory pathogen infections, providing a reference for seasonal and regional patterns of respiratory pathogen transmission, and respiratory infection models on a global scale. Early warning of possible disease outbreaks by monitoring the relationship between temperature changes and pathogen epidemics can help policymakers develop climate-resilient public health policies. Future research and policymaking require several considerations. Firstly, socioeconomic determinants and environmental factors may have modulating effects on population susceptibility, which need to be explored. Secondly, the interaction between changes in multiple climate conditions can lead to more complex effects on health, which may which may underestimate the potential compound risk. Thirdly, underrepresentation of low-income countries and inadequate diagnoses may affect the accuracy of reported results, which may lead to potential underestimation, for example in many parts of Africa. Finally, future studies should consider exploring the impact of ambient temperature on the transmissibility of respiratory pathogens, including effects on effective reproduction numbers and the potential for superspreading events.

## Conclusions

This study found a clear association between temperature increase and respiratory tract specific-pathogen infection, with temperature sensitivity varying according to pathogen types (bacterial, viral) or subtypes. The findings could help to improve modeling of temperature-related respiratory tract infections on a large geographical scale and to develop preventive adaptations tailored to pathogen characteristics (such as vaccine development protocols). This study also highlights the need to consider underlying regional climate conditions when assessing the risk of temperature-related respiratory diseases to gain a more complete understanding of the factors that influence disease occurrence. However, insufficient evidence for bacterial infections has affected our interpretation of the results. We encourage more high-quality future studies to use standardised methods to investigate the association between temperature and respiratory pathogen infection.

## Supplementary Data

15904000 Supplement
