# Supplementary material for: Global meta-analysis of short-term associations between ambient temperature and pathogen-specific respiratory infections, 2004 to 2023
Source: Euro Surveill. 2025 Mar 20;30(11):2400375. doi: 10.2807/1560-7917.ES.2025.30.11.2400375 (PMC11927074; doi:10.2807/1560-7917.ES.2025.30.11.2400375)
Supplement: Supplement [file 24-00375_ZHU_Supplement.pdf]

Supplementary Material

Global meta-analysis of associations between ambient temperature and pathogen-specific respiratory infections

This supplementary material is hosted by Eurosurveillance as supporting information alongside the article "Global meta-analysis of short-term associations between ambient temperature and pathogen-specific respiratory infections", on behalf of the authors, who remain responsible for the accuracy and appropriateness of the content. The same standards for ethics, copyright, attributions and permissions as for the article apply. Supplements are not edited by Eurosurveillance and the journal is not responsible for the maintenance of any links or email addresses provided therein.

Classified as ECDC NORMAL

Other contents

List of abbreviations……………………………………………………………………………..……………………………………14

List of Tables

Table S1. PRISMA Checklist 11

Table S2. Search strategy: terms, databases, limitations and number of articles for review 14

Table S3. Conversion of effect estimates 20

Table S4. Risk of Bias Assessment in individual studies 21

Table S5. Overall risk of bias rating 23

Table S6. Assessing confidence in the Body of Evidence (adapted from Johnson et al. 2014) 24

Table S7. Strength of evidence definitions for human evidence according to the Navigation Guide (adapted from Johnson et al., 2014) 25

Table S8. Full texts excluded on basis of eligibility 26

Table S9. Basic characteristics of 137 included studies 29

Table S10. Global distribution of common respiratory tract specific pathogen infections studies and sites (applied to the Figure 2) 50

Table S11. Risk of bias (RoB) Assessment for each study (n=138) 53

Table S12. Summary of included studies corresponding to each pathogen 60

Table S13. Meta-regression results 63

Table S14: Summary of the assessment of the quality and strength of the evidence on ambient temperature as a risk factor for incidence of viral, mycoplasma and chlamydia respiratory infections 66

Table S15: Summary of the assessment of the quality and strength of the evidence on ambient temperature as a risk factor for incidence of bacterial respiratory infections ... 77

Classified as ECDC NORMAL

Table S16: Summary of the assessment of the quality and strength of the evidence on ambient temperature as a risk factor for incidence of mycoplasma and chlamydia respiratory infections 79

List of Figures

Figure X1 | Overview of Navigation Guide systematic review methodology used for rating the quality and strength of the human evidence. 81

Figure S1. Random-effects meta-analysis of respiratory syncytial virus (RSV) estimates (62 studies) 82

Figure S1-1. Subgroup analysis of respiratory syncytial virus (RSV) meta-analysis by Köppen-Geiger climate 83

Figure S1-2. Subgroup analysis of respiratory syncytial virus (RSV) meta-analysis by World Bank income category 84

Figure S1-3. Subgroup analysis of respiratory syncytial virus (RSV) meta-analysis by temporal resolution 85

Figure S1-4. Subgroup analysis of respiratory syncytial virus (RSV) meta-analysis by exposure measure 86

Figure S1-5. Subgroup analysis of respiratory syncytial virus (RSV) meta-analysis by modelling approach 87

Figure S1-6. Subgroup analysis of respiratory syncytial virus (RSV) meta-analysis by lag type 88

Figure S1-7. Leave-one-out analysis of respiratory syncytial virus (RSV) meta-analysis 89

Figure S1-8. Funnel plot of respiratory syncytial virus (RSV) estimates 89

Figure S1-9. Trim and fill of respiratory syncytial virus (RSV) meta-analysis 90

Figure S2. Random-effects meta-analysis of influenza virus (IV) estimates (27 studies) 91

Figure S2-1. Subgroup analysis of influenza virus (IV) meta-analysis by Köppen-Geiger climate 92

Figure S2-2. Subgroup analysis of influenza virus (IV) meta-analysis by World Bank income category 93

Figure S2-3. Subgroup analysis of influenza virus (IV) meta-analysis by temporal resolution 94

Figure S2-4. Subgroup analysis of influenza virus (IV) meta-analysis exposure measure 95

Classified as ECDC NORMAL

Figure S2-5. Subgroup analysis of influenza virus (IV) meta-analysis by modelling approach 96
Figure S2-6. Subgroup analysis of influenza virus (IV) meta-analysis by lag type 97
Figure S2-7. Leave-one-out analysis of influenza virus (IV) meta-analysis 98
Figure S2-8. Sensitivity analysis by switching the relative risk for cold effects for Li et al 2020 99
Figure S2-9. Funnel plot of influenza virus (IV) estimates 100
Figure S2-10. Trim and fill of influenza virus (IV) meta-analysis 100
Figure S3. Random-effects meta-analysis of human parainfluenza virus (HPIV) estimates (23 studies) 101
Figure S3-1. Subgroup analysis of human parainfluenza virus (HPIV) meta-analysis by Köppen-Geiger climate 102
Figure S3-2. Subgroup analysis of human parainfluenza virus (HPIV) meta-analysis by World Bank income category 102
Figure S3-3. Subgroup analysis of human parainfluenza virus (HPIV) meta-analysis by temporal resolution 103
Figure S3-4. Subgroup analysis of human parainfluenza virus (HPIV) meta-analysis by exposure measure 104
Figure S3-5. Subgroup analysis of human parainfluenza virus (HPIV) meta-analysis by modelling approach 105
Figure S3-6. Subgroup analysis of human parainfluenza virus (HPIV) meta-analysis by lag type 106
Figure S3-7. Leave-one-out analysis of human parainfluenza virus (HPIV) meta-analysis 107
Figure S3-8. Funnel plot of human parainfluenza virus (HPIV) estimates 107
Figure S3-9 Trim and fill of human parainfluenza virus (HPIV) meta-analysis 108
Figure S4. Random-effects meta-analysis of human metapneumoviruses (HMPV) estimates (14 studies) 108
Figure S4-1. Subgroup analysis of human metapneumoviruses (HMPV) meta-analysis by Köppen-Geiger climate 109
Figure S4-2. Subgroup analysis of human metapneumoviruses (HMPV) meta-analysis by World Bank income category 109

Classified as ECDC NORMAL

Figure S4-3. Subgroup analysis of human metapneumoviruses (HMPV) meta-analysis by temporal resolution 110
Figure S4-4. Subgroup analysis of human metapneumoviruses (HMPV) meta-analysis by exposure measure 110
Figure S4-5. Subgroup analysis of human metapneumoviruses (HMPV) meta-analysis by modelling approach 111
Figure S4-6. Leave-one-out analysis of human metapneumoviruses (HMPV) meta-analysis 111
Figure S4-7. Funnel plot of human metapneumoviruses (HMPV) estimates 112
Figure S4-8. Trim and fill of human metapneumoviruses (HMPV) meta-analysis 112
Figure S5. Random-effects meta-analysis of human rhinovirus (HRV) estimates (12 studies) 112
Figure S5-1. Subgroup analysis of human rhinovirus (HRV) meta-analysis by Köppen-Geiger climate 113
Figure S5-2. Subgroup analysis of human rhinovirus (HRV) meta-analysis by World Bank income category 114
Figure S5-3. Subgroup analysis of human rhinovirus (HRV) meta-analysis by temporal resolution 114
Figure S5-4. Subgroup analysis of human rhinovirus (HRV) meta-analysis by exposure measure 115
Figure S5-5. Subgroup analysis of human rhinovirus (HRV) meta-analysis by modelling approach 115
Figure S5-6. Leave-one-out analysis of human rhinovirus (HRV) meta-analysis 116
Figure S5-7. Funnel plot of human rhinovirus (HRV) estimates 116
Figure S5-8. Trim and fill of human rhinovirus (HRV) meta-analysis 116
Figure S6. Random-effects meta-analysis of human adenoviruses (HAdVs) estimates (16 studies) 117
Figure S6-1. Subgroup analysis of human adenoviruses (HAdVs) meta-analysis by Köppen-Geiger climate 117
Figure S6-2. Subgroup analysis of human adenoviruses (HAdVs) meta-analysis by World Bank income category 118
Figure S6-3. Subgroup analysis of human adenoviruses (HAdVs) meta-analysis by temporal resolution 118

Classified as ECDC NORMAL

Figure S6-4. Subgroup analysis of human adenoviruses (HAdVs) meta-analysis by exposure measure ... 119

Figure S6-5. Subgroup analysis of human adenoviruses (HAdVs) meta-analysis by modelling approach ... 119

Figure S6-6. Leave-one-out analysis of human adenoviruses (HAdVs) meta-analysis ... 120

Figure S6-7. Funnel plot of human adenoviruses (HAdVs) estimates ... 120

Figure S6-8. Trim and fill of human adenoviruses (HAdVs) meta-analysis ... 120

Figure S7. Random-effects meta-analysis of human bocavirus (HBoV) estimates (nine studies) ... 121

Figure S7-1. Subgroup analysis of human bocavirus (HBoV) meta-analysis by Köppen-Geiger climate ... 122

Figure S7-2. Subgroup analysis of human bocavirus (HBoV) meta-analysis by World Bank income category ... 122

Figure S7-3. Subgroup analysis of human bocavirus (HBoV) meta-analysis by temporal resolution ... 122

Figure S7-4. Subgroup analysis of human bocavirus (HBoV) meta-analysis by exposure measure ... 122

Figure S7-5. Subgroup analysis of human bocavirus (HBoV) meta-analysis by modelling approach ... 123

Figure S7-6. Leave-one-out analysis of human bocavirus (HBoV) meta-analysis ... 123

Figure S8. Random-effects meta-analysis of enterovirus(EV) estimates (four studies) ... 123

Figure S8-1. Subgroup analysis of enterovirus (EV) meta-analysis by Köppen-Geiger climate ... 123

Figure S8-2. Subgroup analysis of enterovirus (EV) meta-analysis by World Bank income category ... 124

Figure S8-3. Subgroup analysis of enterovirus (EV) meta-analysis by temporal resolution ... 124

Figure S8-4. Subgroup analysis of enterovirus (EV) meta-analysis by exposure measure ... 124

Figure S8-5. Subgroup analysis of enterovirus (EV) meta-analysis by modelling approach ... 124

Figure S8-6. Leave-one-out analysis of enterovirus (EV) meta-analysis ... 125

Classified as ECDC NORMAL

Figure S8-7. Sensitivity analysis by excluding study with high risk of bias for Jean et al 2009 125
Figure S9. Random-effects meta-analysis of severe acute respiratory syndrome coronavirus 2 (SARS-CoV-2) estimates (nine studies) 125
Figure S9-1. Subgroup analysis of severe acute respiratory syndrome coronavirus 2 (SARS-CoV-2) meta-analysis by Köppen-Geiger climate 125
Figure S9-2. Subgroup analysis of severe acute respiratory syndrome coronavirus 2 (SARS-CoV-2) meta-analysis by World Bank income category 127
Figure S9-3. Subgroup analysis of severe acute respiratory syndrome coronavirus 2 (SARS-CoV-2) meta-analysis by temporal resolution 127
Figure S9-4. Subgroup analysis of severe acute respiratory syndrome coronavirus 2 (SARS-CoV-2) meta-analysis by exposure measure 128
Figure S9-5. Subgroup analysis of severe acute respiratory syndrome coronavirus 2 (SARS-CoV-2) meta-analysis by modelling approach 128
Figure S9-6. Subgroup analysis of severe acute respiratory syndrome coronavirus 2 (SARS-CoV-2) meta-analysis by lag type 129
Figure S9-7. Leave-one-out analysis of severe acute respiratory syndrome coronavirus 2 (SARS-CoV-2) meta-analysis 129
Figure S10. Random-effects meta-analysis of severe acute respiratory syndrome coronavirus (SARS-CoV) estimates (four studies) 130
Figure S10-1. Subgroup analysis of severe acute respiratory syndrome (SARS) meta-analysis by Köppen-Geiger climate 130
Figure S10-2. Subgroup analysis of severe acute respiratory syndrome coronavirus (SARS-CoV) meta-analysis by World Bank income category 131
Figure S10-3. Subgroup analysis of severe acute respiratory syndrome coronavirus (SARS-CoV) meta-analysis by temporal resolution 131
Figure S10-4. Subgroup analysis of severe acute respiratory syndrome coronavirus (SARS-CoV) meta-analysis by exposure measure 131
Figure S10-5. Subgroup analysis of severe acute respiratory syndrome coronavirus (SARS-CoV) meta-analysis by modelling approach 132
Figure S10-6. Leave-one-out analysis of severe acute respiratory syndrome coronavirus (SARS-CoV) meta-analysis 132
Figure S11. Random-effects meta-analysis of human coronavirus (HCoV) estimates (five studies) 132
Figure S11-1. Subgroup analysis of human coronavirus (HCoV) meta-analysis by Köppen-Geiger climate 133
Figure S11-2. Subgroup analysis of human coronavirus (HCoV) meta-analysis by World Bank income category 133

Classified as ECDC NORMAL

Figure S11-3. Subgroup analysis of human coronavirus (HCoV) meta-analysis by temporal resolution 133

Figure S11-4. Subgroup analysis of human coronavirus (HCoV) meta-analysis by exposure measure 134

Figure S11-5. Subgroup analysis of human coronavirus (HCoV) meta-analysis by modelling approach 134

Figure S11-6. Leave-one-out analysis of human coronavirus (HCoV) meta-analysis 134

Figure S12. Random-effects meta-analysis of Human rhinoviruses/enteroviruses (HRVs/EVs) estimates (two studies) 134

Figure S12-1. Subgroup analysis of Human rhinoviruses/enteroviruses (HRVs/EVs) meta-analysis by Köppen-Geiger climate 135

Figure S12-2. Subgroup analysis of Human rhinoviruses/enteroviruses (HRVs/EVs) meta-analysis by World Bank income category 135

Figure S12-3. Subgroup analysis of Human rhinoviruses/enteroviruses (HRVs/EVs) meta-analysis by temporal resolution 135

Figure S12-4. Subgroup analysis of Human rhinoviruses/enteroviruses (HRVs/EVs) meta-analysis by exposure measure 135

Figure S12-5. Subgroup analysis of Human rhinoviruses/enteroviruses (HRVs/EVs) meta-analysis by modelling approach 135

Figure S13. Subgroup analysis of bacterial respiratory infections meta-analysis by pathogen 136

Figure S14. Random-effects meta-analysis of *Streptococcus pneumoniae* estimates (four studies) 136

Figure S14-1. Subgroup analysis of *Streptococcus pneumoniae* meta-analysis by Köppen-Geiger climate 136

Figure S14-2. Subgroup analysis of *Streptococcus pneumoniae* meta-analysis by World Bank income category 137

Figure S14-3. Subgroup analysis of *Streptococcus pneumoniae* meta-analysis by temporal resolution 137

Figure S14-4. Subgroup analysis of *Streptococcus pneumoniae* meta-analysis by exposure measure 137

Figure S14-5. Subgroup analysis of *Streptococcus pneumoniae* meta-analysis by modelling approach 137

Figure S14-6. Leave-one-out analysis of *Streptococcus pneumoniae* meta-analysis 138

Figure S15. Random-effects meta-analysis of *Mycoplasma pneumoniae* estimates (19 studies) 138

Classified as ECDC NORMAL

Figure S15-1. Subgroup analysis of *Mycoplasma pneumoniae* meta-analysis by Köppen-Geiger climate 138

Figure S15-2. Subgroup analysis of *Mycoplasma pneumoniae* meta-analysis by World Bank income category 140

Figure S15-3. Subgroup analysis of *Mycoplasma pneumoniae* meta-analysis by temporal resolution 140

Figure S15-4. Subgroup analysis of *Mycoplasma pneumoniae* meta-analysis by exposure measure 141

Figure S15-5. Subgroup analysis of *Mycoplasma pneumoniae* meta-analysis by modelling approach 141

Figure S15-6. Leave-one-out analysis of *Mycoplasma pneumoniae* meta-analysis 142

Figure S15-7. Funnel plot of *Mycoplasma pneumoniae* estimates 142

Figure S15-8. Trim and fill of *Mycoplasma pneumoniae* meta-analysis 142

Figure S16. Random-effects meta-analysis of *Chlamydia pneumoniae* estimates (four studies) 143

Figure S16-1. Subgroup analysis of *Chlamydia pneumoniae* meta-analysis by Köppen-Geiger climate 143

Figure S16-2. Subgroup analysis of *Chlamydia pneumonia* meta-analysis by World Bank income category 143

Figure S16-3. Subgroup analysis of *Chlamydia pneumoniae* meta-analysis by temporal resolution 144

Figure S16-4. Subgroup analysis of *Chlamydia pneumoniae* meta-analysis by exposure measure 144

Figure S16-5. Subgroup analysis of *Chlamydia pneumoniae* meta-analysis by modelling approach 144

Figure S16-6. Leave-one-out analysis of *Chlamydia pneumoniae* meta-analysis 144

Figure S16-7. Sensitivity analysis by excluding study with high risk of bias for Huang et al 2019 144

Classified as ECDC NORMAL

List of abbreviations

OHAT	Office of Health Assessment and Translation
IV	Influenza virus
HPIV	Human parainfluenza virus
RSV	Respiratory syncytial virus
ARI	Acute respiratory infection
GBD	Global Burden of Diseases, Injuries, and Risk Factors Study
DALYs	disability-adjusted life years
RTIs	Respiratory tract infections
HMPV	Human metapneumovirus
HRV	Human rhinovirus
HAdV	Human adenovirus
HBoV	Human bocavirus
MERS-CoV	Middle East Respiratory Syndrome Coronavirus
SARS-CoV	Severe acute respiratory syndrome coronavirus
SARS-CoV-2	Severe acute respiratory syndrome coronavirus 2
HCoV	Human coronavirus
EV	Enterovirus
M. pneumoniae	*Mycoplasma pneumoniae*
C. pneumoniae	*Chlamydophila pneumoniae*
S. pneumoniae	*Streptococcus pneumoniae*
S. pyogenes	*Streptococcus pyogenes pharyngiti*
H. influenzae	*Haemophilus influenzae*
L. pneumoniae	*Legionella pneumoniae*
M. catarrhalis	*Moraxella catarrhalis*
P. aeruginosa	*Pseudomonas aeruginosa*
CBM	China Biology Medicine
WHO	World Health Organization

Classified as ECDC NORMAL

Table S1. PRISMA Checklist

Section and Topic	Item #	Checklist item	Location where item is reported
**TITLE**			
Title	1	Identify the report as a systematic review.	1
**ABSTRACT**			
Abstract	2	See the PRISMA 2020 for Abstracts checklist.	1-2
**INTRODUCTION**			
Rationale	3	Describe the rationale for the review in the context of existing knowledge.	3-4
Objectives	4	Provide an explicit statement of the objective(s) or question(s) the review addresses.	4
**METHODS**			
Eligibility criteria	5	Specify the inclusion and exclusion criteria for the review and how studies were grouped for the syntheses.	4-5
Information sources	6	Specify all databases, registers, websites, organisations, reference lists and other sources searched or consulted to identify studies. Specify the date when each source was last searched or consulted.	5
Search strategy	7	Present the full search strategies for all databases, registers and websites, including any filters and limits used.	5
Selection process	8	Specify the methods used to decide whether a study met the inclusion criteria of the review, including how many reviewers screened each record and each report retrieved, whether they worked independently, and if applicable, details of automation tools used in the process.	5
Data collection process	9	Specify the methods used to collect data from reports, including how many reviewers collected data from each report, whether they worked independently, any processes for obtaining or confirming data from study investigators, and if applicable, details of automation tools used in the process.	6
Data items	10a	List and define all outcomes for which data were sought. Specify whether all results that were compatible with each outcome domain in each study were sought (e.g. for all measures, time points, analyses), and if not, the methods used to decide which results to collect.	6
	10b	List and define all other variables for which data were sought (e.g. participant and intervention characteristics, funding sources). Describe any assumptions made about any missing or unclear information.	6
Study risk of bias assessment	11	Specify the methods used to assess risk of bias in the included studies, including details of the tool(s) used, how many reviewers assessed each study and whether they worked independently, and if applicable, details of automation tools used in the process.	7
Effect measures	12	Specify for each outcome the effect measure(s) (e.g. risk ratio, mean difference) used in the synthesis or presentation of results.	8
Synthesis methods	13a	Describe the processes used to decide which studies were eligible for each synthesis (e.g. tabulating the study intervention characteristics and comparing against the planned groups for each synthesis (item #5)).	8
	13b	Describe any methods required to prepare the data for presentation or synthesis, such as handling of missing summary statistics, or data conversions.	8
	13c	Describe any methods used to tabulate or visually display results of individual studies and syntheses.	8

Classified as ECDC NORMAL

Section and Topic	Item #	Checklist item	Location where item is reported
	13d	Describe any methods used to synthesize results and provide a rationale for the choice(s). If meta-analysis was performed, describe the model(s), method(s) to identify the presence and extent of statistical heterogeneity, and software package(s) used.	8-9
	13e	Describe any methods used to explore possible causes of heterogeneity among study results (e.g. subgroup analysis, meta-regression).	8-9
	13f	Describe any sensitivity analyses conducted to assess robustness of the synthesized results.	8-9
Reporting bias assessment	14	Describe any methods used to assess risk of bias due to missing results in a synthesis (arising from reporting biases).	7-8
Certainty assessment	15	Describe any methods used to assess certainty (or confidence) in the body of evidence for an outcome.	7-8
**RESULTS**			
Study selection	16a	Describe the results of the search and selection process, from the number of records identified in the search to the number of studies included in the review, ideally using a flow diagram.	9
	16b	Cite studies that might appear to meet the inclusion criteria, but which were excluded, and explain why they were excluded.	9
Study characteristics	17	Cite each included study and present its characteristics.	9
Risk of bias in studies	18	Present assessments of risk of bias for each included study.	10
Results of individual studies	19	For all outcomes, present, for each study: (a) summary statistics for each group (where appropriate) and (b) an effect estimate and its precision (e.g. confidence/credible interval), ideally using structured tables or plots.	9-12
Results of syntheses	20a	For each synthesis, briefly summarise the characteristics and risk of bias among contributing studies.	9-12
	20b	Present results of all statistical syntheses conducted. If meta-analysis was done, present for each the summary estimate and its precision (e.g. confidence/credible interval) and measures of statistical heterogeneity. If comparing groups, describe the direction of the effect.	9-12
	20c	Present results of all investigations of possible causes of heterogeneity among study results.	9-12
	20d	Present results of all sensitivity analyses conducted to assess the robustness of the synthesized results.	9-12
Reporting biases	21	Present assessments of risk of bias due to missing results (arising from reporting biases) for each synthesis assessed.	12
Certainty of evidence	22	Present assessments of certainty (or confidence) in the body of evidence for each outcome assessed.	12-13
**DISCUSSION**			
Discussion	23a	Provide a general interpretation of the results in the context of other evidence.	13-14
	23b	Discuss any limitations of the evidence included in the review.	14-16

Classified as ECDC NORMAL

Section and Topic	Item #	Checklist item	Location where item is reported
	23c	Discuss any limitations of the review processes used.	15-16
	23d	Discuss implications of the results for practice, policy, and future research.	16
**OTHER INFORMATION**			
Registration and protocol	24a	Provide registration information for the review, including register name and registration number, or state that the review was not registered.	1
	24b	Indicate where the review protocol can be accessed, or state that a protocol was not prepared.	5
	24c	Describe and explain any amendments to information provided at registration or in the protocol.	5
Support	25	Describe sources of financial or non-financial support for the review, and the role of the funders or sponsors in the review.	Title page
Competing interests	26	Declare any competing interests of review authors.	Title page
Availability of data, code and other materials	27	Report which of the following are publicly available and where they can be found: template data collection forms; data extracted from included studies; data used for all analyses; analytic code; any other materials used in the review.	/

From: Page MJ, McKenzie JE, Bossuyt PM, Boutron I, Hoffmann TC, Mulrow CD, et al. The PRISMA 2020 statement: an updated guideline for reporting systematic reviews. BMJ 2021;372:n71. doi: 10.1136/bmj.n71

For more information, visit: http://www.prisma-statement.org/

Classified as ECDC NORMAL

Table S2. Search strategy: terms, databases, limitations and number of articles for review

PubMed database	
#1	("temperature"[MeSH Terms] OR "temperature"[All Fields] OR "temperatures"[All Fields] OR "temperature s"[All Fields] OR "ambient temperature"[All Fields] OR "seasurface temperature"[All Fields] OR ("climate"[MeSH Terms] OR "climate"[All Fields] OR "climates"[All Fields] OR "climate s"[All Fields] OR "climatic"[All Fields] OR "climatically"[All Fields]) OR ("weather"[MeSH Terms] OR "weather"[All Fields] OR "weatherability"[All Fields] OR "weatherable"[All Fields] OR "weathered"[All Fields] OR "weathering"[All Fields] OR "weathers"[All Fields]) OR ("meteorologic"[All Fields] OR "meteorological"[All Fields] OR "meteorologically"[All Fields]) OR "climate change"[All Fields] OR "meteorolog*"[All Fields] OR "extreme weather"[All Fields] OR "hot weather"[All Fields] OR "cold weather"[All Fields] OR "meteorological factor"[All Fields] OR "climatic factor"[All Fields] OR "global warming"[All Fields])
#2	("Bacterial Pneumonia"[All Fields] OR "pneumonia bacterial"[All Fields] OR "Bacterial Pneumonias"[All Fields] OR "Bacterial Infections and Mycoses"[All Fields] OR "Bacterial Infections"[All Fields] OR "pneumonia pneumococcal"[All Fields] OR "pneumonia staphylococcal"[All Fields] OR "Streptococcus pneumoniae"[All Fields] OR "Chlamydial Pneumonia"[All Fields] OR "pneumonia mycoplasma"[All Fields] OR "Mycoplasma pneumoniae"[All Fields] OR "Schizoplasma pneumoniae"[All Fields] OR "Chlamydophila pneumoniae"[All Fields] OR "Lower respiratory tract"[All Fields] OR "upper respiratory tract"[All Fields] OR "Human Rhinovirus"[All Fields] OR "Human Enterovirus"[All Fields] OR "Respiratory Tract Infections"[All Fields] OR "Respiratory System Infections"[All Fields] OR "Respiratory System Infection"[All Fields] OR "Upper Respiratory Tract Infections"[All Fields] OR "Upper Respiratory Infections"[All Fields] OR "Upper Respiratory Tract Infection"[All Fields] OR "respiratory infections"[All Fields] OR "respiratory virus"[All Fields] OR "Respiratory syncytial virus"[All Fields] OR "Respiratory Syncytial Viruses"[All Fields] OR "RSV"[All Fields] OR "Chimpanzee Coryza Agent"[All Fields] OR ("adenoviridae"[MeSH Terms] OR "adenoviridae"[All Fields] OR "adenoviruses"[All Fields] OR "adenoviruse"[All Fields]) OR "Human Adenovirus"[All Fields] OR Enterovirus [All Fields] OR "Human Adenoviruses"[All Fields] OR "APC Viruses"[All Fields] OR "APC Virus"[All Fields] OR ("orthomyxoviridae"[MeSH Terms] OR "orthomyxoviridae"[All Fields] OR "orthomyxoviruses"[All Fields]) OR ("orthomyxoviridae"[MeSH Terms] OR "orthomyxoviridae"[All Fields] OR "myxoviruses"[All Fields]) OR "Influenza viruses"[All Fields] OR "Influenza A virus"[All Fields] OR "Influenza A viruses"[All Fields] OR "Orthomyxovirus Type A"[All Fields] OR "Influenza Viruses Type A"[All Fields] OR "Influenza B virus"[All Fields] OR "influenza B viruses"[All Fields] OR "Influenza Viruses Type B"[All Fields] OR "Influenza B virus"[All Fields] OR "Influenzavirus C"[All Fields] OR "Influenza C Virus"[All Fields] OR "Influenza C Viruses"[All Fields] OR "Human parainfluenza viruses"[All Fields] OR "parainfluenza virus 2 human"[All Fields] OR "Parainfluenza Virus Type 2"[All Fields] OR "croup associated viruses"[All Fields] OR "croup associated viruses"[All Fields] OR "Human parainfluenza virus 2"[All Fields] OR "croup associated virus"[All Fields] OR "croup associated virus"[All Fields] OR "HPIV"[All Fields] OR ("metapneumovirus"[MeSH Terms] OR "metapneumovirus"[All Fields] OR "metapneumoviruses"[All Fields]) OR ("metapneumovirus"[MeSH Terms] OR "metapneumovirus"[All Fields] OR "metapneumoviruses"[All Fields]) OR "Human Metapneumoviruses"[All Fields] OR "Human Metapneumovirus"[All Fields] OR "HMPV"[All Fields] OR ("rhinovirus"[MeSH Terms] OR "rhinovirus"[All Fields] OR "rhinoviruses"[All Fields]) OR "Human Rhinovirus"[All Fields] OR "Common Cold Virus"[All Fields] OR "Common Cold Viruses"[All Fields] OR "Coryza Virus"[All Fields] OR ("coronavirus"[MeSH Terms] OR "coronavirus"[All Fields] OR "coronaviruses"[All Fields]) OR "SARS coronavirus"[All Fields] OR "severe acute respiratory syndrome related coronavirus"[All Fields] OR "severe acute respiratory syndrome related coronavirus"[All Fields] OR "HCoV-SARS"[All Fields] OR "SARS Virus"[All Fields] OR ("severe acute respiratory syndrome related coronavirus"[MeSH Terms] OR ("severe"[All Fields] AND "acute"[All Fields] AND "respiratory"[All Fields] AND "syndrome related"[All Fields] AND "coronavirus"[All Fields]) OR "severe acute respiratory syndrome related coronavirus"[All Fields] OR "sarbecovirus"[All Fields]) OR ("severe acute respiratory syndrome related coronavirus"[MeSH Terms] OR ("severe"[All Fields] AND "acute"[All Fields] AND "respiratory"[All Fields] AND "syndrome related"[All Fields] AND "coronavirus"[All Fields]) OR "severe acute respiratory syndrome related coronavirus"[All Fields] OR "sarbecoviruses"[All Fields]) OR "Severe acute respiratory syndrome coronavirus"[All Fields] OR "Severe Acute Respiratory Syndrome Virus"[All Fields] OR "SARS-Cov-2"[All Fields] OR "HcoV"[All Fields] OR "NL63"[All Fields] OR "OC43"[All Fields] OR "229E"[All Fields] OR "HKU1"[All Fields] OR "Human bocaviruses"[All Fields] OR "Coxiella burnetii"[All Fields] OR "Chlamydophila psittaci"[All Fields] OR "Legionella pneumophila"[All Fields] OR "Staphylococcus aureus"[All Fields] OR "Haemophilus influenzae"[All Fields] OR "Pseudomonas aeruginosa"[All Fields] OR "Klebsiella pneumoniae"[All Fields] OR "Escherichia coli"[All Fields] OR "Proteus mirabilis"[All Fields] OR "Providencia stuartii"[All Fields] OR "Moraxella catarrhalis"[All Fields] OR "Streptococcus hemolyticus"[All Fields] OR "Streptococcus anginosus"[All Fields] OR "Streptococcus pneumoniae"[All Fields] OR "Gram-positive cocci"[All Fields] OR "Gram-negative bacteria"[All Fields] OR "gram-negative bacillus"[All Fields] OR "Pseudomonas aeruginosa"[All Fields] OR ("candida"[MeSH Terms] OR "candida"[All Fields] OR "candidae"[All Fields] OR "candidas"[All Fields]) OR "Corynebacterium diphtheriae"[All Fields] OR "Bordetella pertussis"[All Fields] OR "M.tuberculosis"[All Fields] OR "Acute bronchitis"[All Fields] OR "acute angina"[All Fields] OR "acute tonsillitis"[All Fields] OR ("palatine tonsil"[MeSH Terms] OR ("palatine"[All Fields] AND "tonsil"[All Fields]) OR "palatine tonsil"[All Fields] OR "tonsil"[All Fields] OR "tonsils"[All Fields] OR "tonsilitis"[All Fields] OR "tonsillitis"[MeSH Terms] OR "tonsillitis"[All Fields] OR "tonsillitides"[All Fields] OR "tonsills"[All Fields]) OR "Sinus Infections"[All Fields] OR "Viral Pneumonias"[All Fields] OR "Acute Coryza"[All Fields] OR "Acute viral pharyngitis"[All Fields] OR ("pharyngitis"[MeSH Terms] OR "pharyngitis"[All Fields] OR "pharyngitides"[All

Classified as ECDC NORMAL

	Fields]) OR "Sore Throats"[All Fields] OR "Acute viral laryngitis"[All Fields] OR "acute pharyngitis"[All Fields] OR "acute epiglottitis"[All Fields] OR ("epiglottal"[All Fields] OR "epiglottis"[MeSH Terms] OR "epiglottis"[All Fields] OR "epiglottic"[All Fields] OR "epiglottitis"[MeSH Terms] OR "epiglottitis"[All Fields] OR "epiglottitides"[All Fields]) OR ("bronchitis"[MeSH Terms] OR "bronchitis"[All Fields] OR "bronchitides"[All Fields]) OR ("larynges"[All Fields] OR "laryngitis"[MeSH Terms] OR "laryngitis"[All Fields] OR "laryngitides"[All Fields] OR "larynx"[MeSH Terms] OR "larynx"[All Fields] OR "laryngeal"[All Fields]) OR ("legionellosis"[MeSH Terms] OR "legionellosis"[All Fields] OR "legionelloses"[All Fields]) OR "Lung Abscess"[All Fields] OR ("blastomycosis"[MeSH Terms] OR "blastomycosis"[All Fields] OR "blastomycoses"[All Fields]) OR "pneumonia pneumocystis"[All Fields] OR ("pharyngitis"[MeSH Terms] OR "pharyngitis"[All Fields] OR "pharyngitides"[All Fields]) OR ("nasopharyngitis"[MeSH Terms] OR "nasopharyngitis"[All Fields]) OR "Retropharyngeal Abscess"[All Fields] OR ("pleurisy"[MeSH Terms] OR "pleurisy"[All Fields] OR "pleurisies"[All Fields]) OR ("pleuropneumonia"[MeSH Terms] OR "pleuropneumonia"[All Fields] OR "pleuropneumonias"[All Fields] OR "pleuropneumoniae"[All Fields]) OR ("pneumonia"[MeSH Terms] OR "pneumonia"[All Fields] OR "pneumonias"[All Fields] OR "pneumoniae"[All Fields] OR "pneumoniae s"[All Fields]) OR ("bronchopneumonia"[MeSH Terms] OR "bronchopneumonia"[All Fields] OR "bronchopneumonias"[All Fields] OR "bronchopneumoniae"[All Fields]) OR ("pleuropneumonia"[MeSH Terms] OR "pleuropneumonia"[All Fields] OR "pleuropneumonias"[All Fields] OR "pleuropneumoniae"[All Fields]) OR ("rhinitis"[MeSH Terms] OR "rhinitis"[All Fields] OR "rhinitides"[All Fields]) OR ("rhinoscleroma"[MeSH Terms] OR "rhinoscleroma"[All Fields] OR "rhinoscleromas"[All Fields]) OR "Severe Acute Respiratory Syndrome"[All Fields] OR ("paranasal sinuses"[MeSH Terms] OR ("paranasal"[All Fields] AND "sinuses"[All Fields]) OR "paranasal sinuses"[All Fields] OR "sinuses"[All Fields] OR "sinusal"[All Fields] OR "sinuse"[All Fields] OR "sinusitis"[MeSH Terms] OR "sinusitis"[All Fields] OR "sinusitides"[All Fields]) OR "Allergic Fungal Sinusitis"[All Fields] OR "Ethmoid Sinusitis"[All Fields] OR "Frontal Sinusitis"[All Fields] OR "Maxillary Sinusitis"[All Fields] OR "Sphenoid Sinusitis"[All Fields] OR ("supraglottitis"[MeSH Terms] OR "supraglottitis"[All Fields]) OR ("epiglottal"[All Fields] OR "epiglottis"[MeSH Terms] OR "epiglottis"[All Fields] OR "epiglottic"[All Fields] OR "epiglottitis"[MeSH Terms] OR "epiglottitis"[All Fields] OR "epiglottitides"[All Fields]) OR ("tracheitis"[MeSH Terms] OR "tracheitis"[All Fields]) OR ("silicotuberculosis"[MeSH Terms] OR "silicotuberculosis"[All Fields]) OR "Whooping Cough"[All Fields])
#3	#1 AND #2
**Cochrane Central Register of Controlled Trials**	
#1	MeSH descriptor: [Temperature] explode all trees
#2	MeSH descriptor: [Climate Change] explode all trees
#3	MeSH descriptor: [Weather] explode all trees
#4	#1 or #2 or #3
#10	(temperature or "ambient temperature" or “seasurface temperature” or climate or weather or meteorological or “climate change” or Meteorolog* or “extreme weather” or “meteorological factor” or “hot weather” or “cold weather” or “climatic factor” or “global warming”):ti,ab,kw
#11	#9 or #10
#12	MeSH descriptor: [Respiratory Tract Infections] explode all trees
#13	MeSH descriptor: [Pneumonia, Bacterial] explode all trees
#14	MeSH descriptor: [Respiratory Syncytial Viruses] explode all trees
#15	MeSH descriptor: [Mycoplasma pneumoniae] explode all trees
#16	MeSH descriptor: [Adenoviridae] explode all trees
#17	#12 or #13 or #14 or #15 or #16
#18	(“Bacterial Pneumonia” or “Pneumonia, Bacterial” or “Bacterial Pneumonias” or “Bacterial Infections and Mycoses” or “Bacterial Infections” or “Pneumonia, Pneumococcal” or “Pneumonia, Staphylococcal” or “Streptococcus pneumoniae” or “Chlamydial Pneumonia” or “Pneumonia, Mycoplasma” or “Mycoplasma pneumoniae” or “Schizoplasma pneumoniae” or “Chlamydophila pneumoniae” or “Lower respiratory tract” or “upper respiratory tract” or “Human Rhinovirus” or “Human Enterovirus” or “Respiratory Tract Infections” or “Respiratory System Infections” or “Respiratory System Infection” or “Upper Respiratory Tract Infections” or “Upper Respiratory Infections” or “Upper Respiratory Tract Infection” or “respiratory infections” or “respiratory virus” or “Respiratory syncytial virus” or “Respiratory Syncytial Viruses” or “RSV” or “Chimpanzee Coryza Agent” or Adenoviruses or “Human Adenovirus” or “Human Adenoviruses” or “APC Viruses” or “APC Virus” or Orthomyxoviruses or Myxoviruses or “Influenza viruses” or “Influenza A virus” or “Influenza A viruses” or “Orthomyxovirus Type A” or “Influenza Viruses Type A” or “Influenza B virus” or “influenza B viruses” or “Influenza Viruses Type B” or “Influenza B virus” or “Influenzavirus C” or “Influenza C Virus” or “Influenza C Viruses” or “Human parainfluenza viruses” or “Parainfluenza Virus 2, Human” or “Parainfluenza Virus Type 2” or “Croup-Associated Viruses” or “Croup Associated Viruses” or “Human parainfluenza virus 2” or “Croup-Associated Virus” or “Croup Associated Virus” or “HPIV” or Metapneumovirus or Metapneumoviruses or “Human Metapneumoviruses” or “Human Metapneumovirus” or “HMPV” or Rhinoviruses or “Human Rhinovirus” or “Common Cold Virus” or “Common Cold Viruses” or “Coryza Virus” or Coronaviruses or “SARS coronavirus” or “Severe acute respiratory syndrome-related coronavirus” or “Severe acute respiratory syndrome related coronavirus” or “HCoV-SARS” or “SARS Virus” or Sarbecovirus or Sarbecoviruses or “Severe acute respiratory syndrome coronavirus” or “Severe Acute Respiratory Syndrome Virus” or“SARS-Cov-2” or “HcoV” or “NL63” or “OC43” or “229E” or “HKU1”or Enterovirus or “Human bocaviruses” or “Coxiella burnetii” or “Chlamydophila psittaci” or “Legionella pneumophila” or “Staphylococcus aureus” or “Haemophilus influenzae” or “Pseudomonas

Classified as ECDC NORMAL

	aeruginosa" or "Klebsiella pneumoniae" or "Proteus mirabilis" or "Providencia stuartii" or "Moraxella catarrhalis" or "Streptococcus hemolyticus" or "Streptococcus anginosus" or "streptococcus pneumoniae" or "Gram-positive cocci" or "Gram-negative bacteria" or "gram-negative bacillus" or "pseudomonas aeruginosa" or Candida or "Corynebacterium diphtheriae" or "Bordetella pertussis" or "M.tuberculosis" or "Acute bronchitis" or "acute angina" or "acute tonsillitis" or tonsillitis or "Sinus Infections" or "Viral Pneumonias" or "Acute Coryza" or "Acute viral pharyngitis" or Pharyngitides or "Sore Throats" or "Acute viral laryngitis" or "acute pharyngitis" or "acute epiglottitis" or Epiglottitides or Bronchitis or Laryngitis or Legionellosis or "Lung Abscess" or Blastomycosis or "Pneumonia, Pneumocystis" or Pharyngitis or Nasopharyngitis or "Retropharyngeal Abscess" or Pleurisy or Pleuropneumonia or Pneumonia or Bronchopneumonia or Pleuropneumonia or Rhinitis or Rhinoscleroma or "Severe Acute Respiratory Syndrome" or Sinusitis or "Allergic Fungal Sinusitis" or "Ethmoid Sinusitis" or "Frontal Sinusitis" or "Maxillary Sinusitis" or "Sphenoid Sinusitis" or Supraglottitis or Epiglottitis or Tracheitis or Silicotuberculosis or "Whooping Cough"):ti,ab,kw
#19	#17 or #18
#20	#11 and #19
**Scopus**	
#1	(TITLE-ABS-KEY ((temperature OR "ambient temperature" OR "seasurface temperature" OR climate OR weather OR meteorological OR "climate change" OR meteorolog* OR "extreme weather" OR "meteorological factor" OR "climatic factor" OR "hot weather" OR "cold weather" OR "global warming")) AND TITLE-ABS-KEY (("Bacterial Pneumonia" OR "Pneumonia, Bacterial" OR "Bacterial Pneumonias" OR "Bacterial Infections and Mycoses" OR "Bacterial Infections" OR "Pneumonia, Pneumococcal" OR "Pneumonia, Staphylococcal" OR "Streptococcus pneumoniae" OR "Chlamydial Pneumonia" OR "Pneumonia, Mycoplasma" OR "Mycoplasma pneumoniae" OR "Schizoplasma pneumoniae" OR "Chlamydophila pneumoniae" OR "Lower respiratory tract" OR "upper respiratory tract" OR "Human Rhinovirus" OR "Human Enterovirus" OR "Respiratory Tract Infections" OR "Respiratory System Infections" OR "Respiratory System Infection" OR "Upper Respiratory Tract Infections" OR "Upper Respiratory Infections" OR "Upper Respiratory Tract Infection" OR "respiratory infections" OR "respiratory virus" OR "Respiratory syncytial virus" OR "Respiratory Syncytial Viruses" OR "RSV" OR "Chimpanzee Coryza Agent" OR adenoviruses OR "Human Adenovirus" OR "Human Adenoviruses" OR "APC Viruses" OR "APC Virus" OR orthomyxoviruses OR myxoviruses OR "Influenza viruses" OR "Influenza A virus" OR "Influenza A viruses" OR "Orthomyxovirus Type A" OR "Influenza Viruses Type A" OR "Influenza B virus" OR "influenza B viruses" OR "Influenza Viruses Type B" OR "Influenza B virus" OR "Influenzavirus C" OR "Influenza C Virus" OR "Influenza C Viruses" OR "Human parainfluenza viruses" OR "Parainfluenza Virus 2, Human" OR "Parainfluenza Virus Type 2" OR "Croup-Associated Viruses" OR "Croup Associated Viruses" OR "Human parainfluenza virus 2" OR "Croup-Associated Virus" OR "Croup Associated Virus" OR "HPIV" OR metapneumovirus OR metapneumoviruses OR "Human Metapneumoviruses" OR "Human Metapneumovirus" OR "HMPV" OR rhinoviruses OR "Human Rhinovirus" OR "Common Cold Virus" OR "Common Cold Viruses" OR "Coryza Virus" OR coronaviruses OR "SARS coronavirus" OR "Severe acute respiratory syndrome-related coronavirus" OR "Severe acute respiratory syndrome related coronavirus" OR "HCoV-SARS" OR "SARS Virus" OR "SARS-Cov-2" OR "HcoV" OR "NL63" OR "OC43" OR "229E" OR "HKU1" OR sarbecovirus OR sarbecoviruses OR "Severe acute respiratory syndrome coronavirus" OR "Severe Acute Respiratory Syndrome Virus" OR "Human bocaviruses" OR "Coxiella burnetii" OR "Chlamydophila psittaci" OR "Legionella pneumophila" OR "Staphylococcus aureus" OR "Haemophilus influenzae" OR enterovirus OR "Pseudomonas aeruginosa" OR "Klebsiella pneumoniae" OR "Proteus mirabilis" OR "Providencia stuartii" OR "Moraxella catarrhalis" OR "Streptococcus hemolyticus" OR "Streptococcus anginosus" OR "streptococcus pneumoniae" OR "Gram-positive cocci" OR "Gram-negative bacteria" OR "gram-negative bacillus" OR "pseudomonas aeruginosa" OR candida OR "Corynebacterium diphtheriae" OR "Bordetella pertussis" OR "M.tuberculosis" OR "Acute bronchitis" OR "acute angina" OR "acute tonsillitis" OR tonsillitis OR "Sinus Infections" OR "Viral Pneumonias" OR "Acute Coryza" OR "Acute viral pharyngitis" OR pharyngitides OR "Sore Throats" OR "Acute viral laryngitis" OR "acute pharyngitis" OR "acute epiglottitis" OR epiglottitides OR bronchitis OR laryngitis OR legionellosis OR "Lung Abscess" OR blastomycosis OR "Pneumonia, Pneumocystis" OR pharyngitis OR nasopharyngitis OR "Retropharyngeal Abscess" OR pleurisy OR pleuropneumonia OR pneumonia OR bronchopneumonia OR pleuropneumonia OR rhinitis OR rhinoscleroma OR "Severe Acute Respiratory Syndrome" OR sinusitis OR "Allergic Fungal Sinusitis" OR "Ethmoid Sinusitis" OR "Frontal Sinusitis" OR "Maxillary Sinusitis" OR "Sphenoid Sinusitis" OR supraglottitis OR epiglottitis OR tracheitis OR silicotuberculosis OR " Whooping Cough")))
**EMBASE**	
#1	temperature:ab,ti OR 'ambient temperature':ab,ti OR 'seasurface temperature':ab,ti OR rain*:ab,ti OR climate:ab,ti OR weather:ab,ti OR meteorological:ab,ti OR 'climate change':ab,ti OR 'extreme weather':ab,ti OR 'meteorological factor':ab,ti OR 'hot weather':ab,ti OR 'cold weather':ab,ti OR snow:ab,ti OR 'climatic factor':ab,ti OR 'global warming':ab,ti
#2	'bacterial pneumonia':ab,ti OR 'pneumonia, bacterial':ab,ti OR 'bacterial pneumonias':ab,ti OR 'bacterial infections':ab,ti OR mycoses:ab,ti OR 'bacterial infections':ab,ti OR 'pneumonia, pneumococcal':ab,ti OR 'pneumonia, staphylococcal':ab,ti OR 'chlamydial pneumonia':ab,ti OR 'pneumonia, mycoplasma':ab,ti OR 'mycoplasma pneumoniae':ab,ti OR 'schizoplasma pneumoniae':ab,ti OR 'chlamydophila pneumoniae':ab,ti OR 'lower respiratory tract':ab,ti OR 'upper respiratory tract':ab,ti OR 'human enterovirus':ab,ti OR 'respiratory tract infections':ab,ti OR 'respiratory system infections':ab,ti OR 'respiratory system infection':ab,ti OR 'upper respiratory tract infections':ab,ti OR 'upper respiratory infections':ab,ti OR 'upper respiratory tract infection':ab,ti OR 'respiratory infections':ab,ti OR 'respiratory virus':ab,ti OR 'respiratory syncytial virus':ab,ti OR 'respiratory

Classified as ECDC NORMAL

	syncytial viruses':ab,ti OR 'rsv':ab,ti OR 'chimpanzee coryza agent':ab,ti OR adenoviruses:ab,ti OR 'human adenovirus':ab,ti OR 'human adenoviruses':ab,ti OR 'apc viruses':ab,ti OR 'apc virus':ab,ti OR orthomyxoviruses:ab,ti OR myxoviruses:ab,ti OR 'influenza viruses':ab,ti OR 'influenza a virus':ab,ti OR 'influenza a viruses':ab,ti OR 'orthomyxovirus type a':ab,ti OR 'influenza viruses type a':ab,ti OR 'influenza b viruses':ab,ti OR 'influenza viruses type b':ab,ti OR 'influenza b virus':ab,ti OR 'influenzavirus c':ab,ti OR 'influenza c virus':ab,ti OR 'influenza c viruses':ab,ti OR 'human parainfluenza viruses':ab,ti OR 'parainfluenza virus 2, human':ab,ti OR 'parainfluenza virus type 2':ab,ti OR 'croup-associated viruses':ab,ti OR 'croup associated viruses':ab,ti OR 'human parainfluenza virus 2':ab,ti OR 'croup-associated virus':ab,ti OR 'croup associated virus':ab,ti OR 'hpiv':ab,ti OR metapneumovirus:ab,ti OR metapneumoviruses:ab,ti OR 'human metapneumoviruses':ab,ti OR 'human metapneumovirus':ab,ti OR 'hmpv':ab,ti OR rhinoviruses:ab,ti OR 'human rhinovirus':ab,ti OR 'common cold virus':ab,ti OR 'common cold viruses':ab,ti OR 'coryza virus':ab,ti OR coronaviruses:ab,ti OR 'sars coronavirus':ab,ti OR 'severe acute respiratory syndrome-related coronavirus':ab,ti OR 'Enterovirus':ab,ti OR 'severe acute respiratory syndrome related coronavirus':ab,ti OR 'hcov-sars':ab,ti OR 'sars virus':ab,ti OR sarbecovirus:ab,ti OR sarbecoviruses:ab,ti OR 'severe acute respiratory syndrome coronavirus':ab,ti OR 'severe acute respiratory syndrome virus':ab,ti OR 'HcoV':ab,ti OR 'NL63':ab,ti OR 'OC43':ab,ti OR '229E':ab,ti OR 'HKU1':ab,ti OR 'human bocaviruses':ab,ti OR 'coxiella burnetii':ab,ti OR 'chlamydophila psittaci':ab,ti OR 'legionella pneumophila':ab,ti OR 'staphylococcus aureus':ab,ti OR 'haemophilus influenzae':ab,ti OR 'klebsiella pneumoniae':ab,ti OR 'proteus mirabilis':ab,ti OR 'providencia stuartii':ab,ti OR 'moraxella catarrhalis':ab,ti OR 'streptococcus hemolyticus':ab,ti OR 'streptococcus anginosus':ab,ti OR 'streptococcus pneumoniae':ab,ti OR 'gram-positive cocci':ab,ti OR 'gram-negative bacteria':ab,ti OR 'gram-negative bacillus':ab,ti OR 'pseudomonas aeruginosa':ab,ti OR candida:ab,ti OR 'corynebacterium diphtheriae':ab,ti OR 'bordetella pertussis':ab,ti OR 'm.tuberculosis':ab,ti OR 'acute bronchitis':ab,ti OR 'acute angina':ab,ti OR 'acute tonsillitis':ab,ti OR tonsillitis:ab,ti OR 'sinus infections':ab,ti OR 'viral pneumonias':ab,ti OR 'acute coryza':ab,ti OR 'acute viral pharyngitis':ab,ti OR pharyngitides:ab,ti OR 'sore throats':ab,ti OR 'acute viral laryngitis':ab,ti OR 'acute pharyngitis':ab,ti OR 'acute epiglottitis':ab,ti OR epiglottitides:ab,ti OR bronchitis:ab,ti OR laryngitis:ab,ti OR legionellosis:ab,ti OR 'lung abscess':ab,ti OR blastomycosis:ab,ti OR 'pneumonia, pneumocystis':ab,ti OR pharyngitis:ab,ti OR nasopharyngitis:ab,ti OR 'retropharyngeal abscess':ab,ti OR pleurisy:ab,ti OR pneumonia:ab,ti OR bronchopneumonia:ab,ti OR pleuropneumonia:ab,ti OR rhinitis:ab,ti OR rhinoscleroma:ab,ti OR 'severe acute respiratory syndrome':ab,ti OR sinusitis:ab,ti OR 'allergic fungal sinusitis':ab,ti OR 'ethmoid sinusitis':ab,ti OR 'frontal sinusitis':ab,ti OR 'maxillary sinusitis':ab,ti OR 'sphenoid sinusitis':ab,ti OR supraglottitis:ab,ti OR epiglottitis:ab,ti OR tracheitis:ab,ti OR silicotuberculosis:ab,ti OR 'whooping cough':ab,ti
#3	#1 AND #2
**Web of Science**	
#1	TS= (temperature or "ambient temperature" or “seasurface temperature” or climate or weather or meteorological or “climate change” or “extreme weather” or “meteorological factor” or “hot weather” or “cold weather” or “climatic factor” or “global warming”)
#2	TS= (“Bacterial Pneumonia” or “Pneumonia, Bacterial” or “Bacterial Pneumonias” or “Bacterial Infections and Mycoses” or “Bacterial Infections” or “Pneumonia, Pneumococcal” or “Pneumonia, Staphylococcal” or “Streptococcus pneumoniae” or “Chlamydial Pneumonia” or “Pneumonia, Mycoplasma” or “Mycoplasma pneumoniae” or “Schizoplasma pneumoniae” or “Chlamydophila pneumoniae” or “Lower respiratory tract” or “upper respiratory tract” or “Human Rhinovirus” or “Human Enterovirus” or “Respiratory Tract Infections” or “Respiratory System Infections” or “Respiratory System Infection” or “Upper Respiratory Tract Infections” or “Upper Respiratory Infections” or “Upper Respiratory Tract Infection” or “respiratory infections” or “respiratory virus” or “Respiratory syncytial virus” or “Respiratory Syncytial Viruses” or “RSV” or “Chimpanzee Coryza Agent” or Adenoviruses or “Human Adenovirus” or “Human Adenoviruses” or “APC Viruses” or “APC Virus” or Orthomyxoviruses or Myxoviruses or “Influenza viruses” or “Influenza A virus” or “Influenza A viruses” or “Orthomyxovirus Type A” or “Influenza Viruses Type A” or “Influenza B virus” or “influenza B viruses” or “Influenza Viruses Type B” or “Influenza B virus” or “Influenzavirus C” or “Influenza C Virus” or “Influenza C Viruses” or “Human parainfluenza viruses” or “Parainfluenza Virus 2, Human” or “Parainfluenza Virus Type 2” or “Croup-Associated Viruses” or “Croup Associated Viruses” or “Human parainfluenza virus 2” or “Croup-Associated Virus” or “Croup Associated Virus” or “HPIV” or Metapneumovirus or Metapneumoviruses or “Human Metapneumoviruses” or “Human Metapneumovirus” or “HMPV” or Rhinoviruses or “Human Rhinovirus” or “Common Cold Virus” or “Common Cold Viruses” or “Coryza Virus” or Coronaviruses or “SARS coronavirus” or “Severe acute respiratory syndrome-related coronavirus” or “Severe acute respiratory syndrome related coronavirus” or “HCoV-SARS” or “SARS Virus” or “SARS-Cov-2” or “HcoV” or “NL63” or “OC43” or “229E” or “HKU1” or Sarbecovirus or Sarbecoviruses or “Severe acute respiratory syndrome coronavirus” or “Severe Acute Respiratory Syndrome Virus” or “Human bocaviruses” or Enterovirus or “Coxiella burnetii” or “Chlamydophila psittaci” or “Legionella pneumophila” or “Staphylococcus aureus” or “Haemophilus influenzae” or “Pseudomonas aeruginosa” or “Klebsiella pneumoniae” or “Proteus mirabilis” or “Providencia stuartii” or “Moraxella catarrhalis” or “Streptococcus hemolyticus” or “Streptococcus anginosus” or “streptococcus pneumoniae” or “Gram-positive cocci” or “Gram-negative bacteria” or “gram-negative bacillus” or “pseudomonas aeruginosa” or Candida or “Corynebacterium diphtheriae” or “Bordetella pertussis” or “M.tuberculosis” or “Acute bronchitis” or “acute angina” or “acute tonsillitis” or tonsillitis or “Sinus Infections” or “Viral Pneumonias” or “Acute Coryza” or “Acute viral pharyngitis” or Pharyngitides or “Sore Throats” or “Acute viral laryngitis” or “acute pharyngitis” or “acute epiglottitis” or Epiglottitides or Bronchitis or Laryngitis or Legionellosis or “Lung Abscess” or Blastomycosis or “Pneumonia, Pneumocystis” or Pharyngitis or Nasopharyngitis or “Retropharyngeal Abscess” or Pleurisy or Pleuropneumonia or Pneumonia or Bronchopneumonia or Pleuropneumonia or Rhinitis or Rhinoscleroma or

Classified as ECDC NORMAL

	"Severe Acute Respiratory Syndrome" or Sinusitis or "Allergic Fungal Sinusitis" or "Ethmoid Sinusitis" or "Frontal Sinusitis" or "Maxillary Sinusitis"or "Sphenoid Sinusitis" or Supraglottitis or Epiglottitis or Tracheitis or Silicotuberculosis or "Whooping Cough")
#3	#1 AND #2

**Chinese National Knowledge Infrastructure (CNKI)**	
#1	SU=（温度+环境温度+海表温度+气候+天气+气象+气候变化+极端天气+高温+炎热天气+寒冷天气+气象因素+气候因素+全球变暖）*（呼吸道感染+肺炎支原体+肺炎衣原体+呼吸道病毒+流感病毒+鼻病毒+腺病毒+呼吸道合胞病毒+人类冠状病毒+博卡病毒+肠道病毒+人偏肺病毒+副流感病毒+严重急性呼吸综合征相关冠状病+严重急性呼吸综合征冠状病毒+革兰氏阳性球菌+溶血性链球菌+草绿色链球菌+肺炎链球菌+金黄色葡萄球菌+革兰氏阴性杆菌+流感嗜血杆菌+嗜麦芽窄食单胞菌+肺囊虫+肺炎克雷伯菌+铜绿假单胞菌+革兰氏阴性球菌+化脓性链球菌+卡他莫拉菌+嗜肺军团菌+鲍曼不动杆菌+念珠菌+结核分枝杆菌+结核杆菌+白喉棒状杆菌+百日咳鲍特菌+细菌性感染和真菌病+细菌性感染+肺炎葡萄球菌+下呼吸道感染+上呼吸道感染+急性支气管炎+急性咽峡炎+急性疱疹性咽峡炎+急性扁桃体炎+急性鼻窦炎+细菌性肺炎+病毒性肺炎+非典型肺炎+普通感冒+急性病毒性咽炎+急性病毒性喉炎+急性咽炎+急性会厌炎+咽炎）
**VIP**	
#1	U= (温度 OR 环境温度 OR 海表温度 OR 气候 OR 天气 OR 气象 OR 气候变化 OR 极端天气 OR 高温 OR 炎热天气 OR 寒冷天气 OR 气象因素 OR 气候因素 OR 全球变暖) AND（呼吸道感染 OR 肺炎支原体 OR 肺炎衣原体 OR 呼吸道病毒 OR 流感病毒 OR 鼻病毒 OR 腺病毒 OR 呼吸道合胞病毒 OR 人类冠状病毒 OR 肠道病毒 OR 博卡病毒 OR 人偏肺病毒 OR 副流感病毒 OR 严重急性呼吸综合征相关冠状病 OR 严重急性呼吸综合征冠状病毒 OR 革兰氏阳性球菌 OR 溶血性链球菌 OR 草绿色链球菌 OR 肺炎链球菌 OR 嗜麦芽窄食单胞菌 OR 肺囊虫 OR 金黄色葡萄球菌 OR 革兰氏阴性杆菌 OR 流感嗜血杆菌 OR 肺炎克雷伯菌 OR 铜绿假单胞菌 OR 革兰氏阴性球菌 OR 卡他莫拉菌 OR 嗜肺军团菌 OR 鲍曼不动杆菌 OR 念珠菌 OR 结核分枝杆菌 OR 结核杆菌 OR 白喉棒状杆菌 OR 百日咳鲍特菌 OR 化脓性链球菌 OR 细菌性感染和真菌病 OR 细菌性感染 OR 肺炎葡萄球菌 OR 下呼吸道感染 OR 上呼吸道感染 OR 急性支气管炎 OR 急性咽峡炎 OR 急性疱疹性咽峡炎 OR 急性扁桃体炎 OR 急性鼻窦炎 OR 细菌性肺炎 OR 病毒性肺炎 OR 非典型肺炎 OR 普通感冒 OR 急性病毒性咽炎 OR 急性病毒性喉炎 OR 急性咽炎 OR 急性会厌炎 OR 咽炎）
**WanFang**	
#1	题名或关键词：(温度 OR 环境温度 OR 海表温度 OR 气候 OR 天气 OR 气象 OR 气候变化 OR 极端天气 OR 高温 OR 炎热天气 OR 寒冷天气 OR 气象因素 OR 气候因素 OR 全球变暖) AND（呼吸道感染 OR 肺炎支原体 OR 肺炎衣原体 OR 呼吸道病毒 OR 流感病毒 OR 鼻病毒 OR 腺病毒 OR 呼吸道合胞病毒 OR 人类冠状病毒 OR 肠道病毒 OR 博卡病毒 OR 人偏肺病毒 OR 副流感病毒 OR 严重急性呼吸综合征相关冠状病 OR 严重急性呼吸综合征冠状病毒 OR 革兰氏阳性球菌 OR 溶血性链球菌 OR 草绿色链球菌 OR 肺炎链球菌 OR 嗜麦芽窄食单胞菌 OR 肺囊虫 OR 金黄色葡萄球菌 OR 革兰氏阴性杆菌 OR 流感嗜血杆菌 OR 肺炎克雷伯菌 OR 铜绿假单胞菌 OR 革兰氏阴性球菌 OR 卡他莫拉菌 OR 嗜肺军团菌 OR 鲍曼不动杆菌 OR 念珠菌 OR 结核分枝杆菌 OR 结核杆菌 OR 白喉棒状杆菌 OR 百日咳鲍特菌 OR 化脓性链球菌 OR 细菌性感染和真菌病 OR 细菌性感染 OR 肺炎葡萄球菌 OR 下呼吸道感染 OR 上呼吸道感染 OR 急性支气管炎 OR 急性咽峡炎 OR 急性疱疹性咽峡炎 OR 急性扁桃体炎 OR 急性鼻窦炎 OR 细菌性肺炎 OR 病毒性肺炎 OR 非典型肺炎 OR 普通感冒 OR 急性病毒性咽炎 OR 急性病毒性喉炎 OR 急性咽炎 OR 急性会厌炎 OR 咽炎）
**Chinese BioMedical Literature Database (CBM)**	
#1	("温度"[不加权:扩展] OR "气象学概念"[不加权:扩展]) OR "气候"[不加权:扩展]) OR "天气"[不加权:扩展] OR "极端天气"[不加权:扩展] OR "天气和发病"[不加权:扩展]) OR "气象学概念"[不加权:扩展])
#2	"温度" [常用字段:智能] OR"环境温度" [常用字段:智能] OR"海表温度" [常用字段:智能] OR "气候" [常用字段:智能] OR "天气" [常用字段:智能] OR "气象" [常用字段:智能] OR "气候变化" [常用字段:智能] OR "极端天气" [常用字段:智能] OR "高温" [常用字段:智能] OR "炎热天气" [常用字段:智能] OR "寒冷天气" [常用字段:智能] OR "气象因素" [常用字段:智能] OR "气候因素" [常用字段:智能] OR "全球变暖" [常用字段:智能]
#3	#1 OR #2
#4	("呼吸道感染"[不加权:扩展] OR "肺炎支原体"[不加权:扩展] OR "肺炎衣原体"[不加权:扩展] OR "呼吸道合胞病毒"[不加权:扩展] OR "冠状病毒 OC43, 人"[不加权:扩展] OR "人博卡病毒"[不加权:扩展] OR "革兰氏阳性球菌"[不加权:扩展] OR "肠道病毒属"[不加权:扩展] OR "肠道病毒感染"[不加权:扩展] OR "嗜麦芽窄食单胞菌"[不加权:扩展] OR "草绿色链球菌"[不加权:扩展] OR "金黄色葡萄球菌"[不加权:扩展] OR "肺炎克雷伯菌"[不加权:扩展] OR "铜绿假单胞菌"[不加权:扩展] OR "白色念珠菌"[不加权:扩展])
#5	("呼吸道感染" [常用字段:智能] OR"肺炎支原体" [常用字段:智能] OR"肺炎衣原体" [常用字段:智能] OR"呼吸道病毒" [常用字段:智能] OR"流感病毒" [常用字段:智能] OR"鼻病毒" [常用字段:智能] OR"腺病毒" [常用字段:智能] OR "呼吸道合胞病毒" [常用字段:智能] OR "人类冠状病毒" [常用字段:智能] OR "博卡病毒" [常用字段:智能] OR "肠道病毒" [常用字段:智能] OR "人偏肺病毒" [常用字段:智能] OR "副流感病毒" [常用字段:智能] OR "严重急性呼吸综合征相关冠状病" [常用字段:智能] OR "严重急性呼吸综合征冠状病毒" [常用字段:智能] OR "革兰氏阳性球菌" [常用字段:智能] OR "溶血性链球菌" [常用字段:智能] OR "草绿色链球菌" [常用字段:智能] OR "肺炎链球菌" [常用字段:智能] OR "金黄色葡萄球菌" [常用字段:智能] OR "革兰氏阴性杆菌" [常

Classified as ECDC NORMAL

	用字段:智能] OR "嗜麦芽窄食单胞菌" [常用字段:智能] OR "肺囊虫" [常用字段:智能] OR "流感嗜血杆菌" [常用字段:智能] OR "肺炎克雷伯菌" [常用字段:智能] OR "铜绿假单胞菌" [常用字段:智能] OR 革兰氏阴性球菌" [常用字段:智能] OR "卡他莫拉菌" [常用字段:智能] OR "嗜肺军团菌" [常用字段:智能] OR "鲍曼不动杆菌" [常用字段:智能] OR "念珠菌" [常用字段:智能] OR "结核分枝杆菌" [常用字段:智能] OR "结核杆菌" [常用字段:智能] OR "化脓性链球菌" [常用字段:智能] OR "白喉棒状杆菌" [常用字段:智能] OR "百日咳鲍特菌" [常用字段:智能] OR "细菌性感染" [常用字段:智能] OR "肺炎葡萄球菌" [常用字段:智能] OR "下呼吸道感染" [常用字段:智能] OR "上呼吸道感染" [常用字段:智能] OR "急性支气管炎" [常用字段:智能] OR "急性咽峡炎" [常用字段:智能] OR "急性疱疹性咽峡炎" [常用字段:智能] OR "急性扁桃体炎" [常用字段:智能] OR "急性鼻窦炎" [常用字段:智能] OR "细菌性肺炎" [常用字段:智能] OR "病毒性肺炎" [常用字段:智能] OR "非典型肺炎" [常用字段:智能] OR "普通感冒" [常用字段:智能] OR "急性病毒性咽炎" [常用字段:智能] OR "急性病毒性喉炎" [常用字段:智能] OR "急性咽炎" [常用字段:智能] OR "急性会厌炎" [常用字段:智能]OR 咽炎" [常用字段:智能]）
#6	#4 OR #5
#7	#3 AND #6

Search terms and protocol

PubMed, Scopus, Embase, the Cochrane Library, Web of Science, China National Knowledge Infrastructure, Wanfang Data, Weipu Database, and China Biology Medicine (CBM) for observational studies were comprehensively searched. we identified studies that explored the effect of relationship between temperature and morbidity, hospital admissions, outpatient visits, mortality, or reported cases of respiratory infection surveillance. Zotero and Covidence tools were used for screening. Additionally manual-searched the literature through forward and backward citation tracing using CoCites to obtain remaining studies

Procedure:

1. Search the terms in the table below
2. Download references
3. Import document management software
3. 3. Review all paper titles for eligibility
4. Make record of how many research results were found and how many papers were retrieved by each platform
5. Remove duplicates; record the number of records before and after
6. Screen these abstracts for eligibility- sometimes a screen of the full paper
7. Select the list of final papers for review, sending these references and the number of papers reviewed at each stage to other reviewer
8. All authors agree on which studies are ultimately included

PECO Statement

***Panel*: Population-Exposure-Comparators-Outcomes framework**
**Population**<br>assessing respiratory disease risk among the general population<br>**Exposure**<br>per 1°C increase in temperature (defined as daily/weekly/monthly/ mean/min/max temperature)<br>**Comparators**<br>Comparable populations not exposed to the relevant ambient temperature<br>**Outcomes**<br>the risk of morbidity due to pathogen-specific respiratory infections

Classified as ECDC NORMAL

Table S3. Conversion of effect estimates

If RR values were not directly provided in the study, we performed the following transformations.

Effect estimates	Conversion
**Excessrisk (ER)**	RR=（ER÷100）+1
**Percentage change**	Divide by 100 plus 1
**Beta coefficients**	exp (beta)
**Correlation coefficient r**	We use the following steps to calculate a confidence interval for the population correlation coefficient based on the sample size n and the sample correlation coefficient r.<br>In order to normalize the distribution of r and to make the variance independent of p, Fisher (1921) proposed the z-transformation<br>$z_r = \ln((1 + r)/(1 - r)) / 2$<br>Find the upper and lower limits of the logarithm<br>$L = z_r - (z_{1-\alpha/2} / \sqrt{n-3})$<br>$U = z_r + (z_{1-\alpha/2} / \sqrt{n-3})$<br>Find the confidence interval<br>Confidence interval = $[(e^{2L} -1)/(e^{2L} +1), (e^{2U} -1)/(e^{2U} +1)]$<br>Then, correlation coefficient convert to effect sizes (OR), refer to the method of Lenhard 2022.<br>Lenhard, W. & Lenhard, A. (2022). Computation of effect sizes. Retrieved from: https://www.psychometrica.de/effect_size.html. Psychometrica. DOI: 10.13140/RG.2.2.17823.9232
**OR, SE**	Calculate 95% CI:<br>L= OR-1.96 x SE<br>U= OR+1.96 x SE<br>If the value is negative and does not conform to normal distribution, log conversion is performed：<br>L= exp(log(OR) - 1.96 x log(SE))<br>U= exp(log(OR) + 1.96 x log(SE))

Classified as ECDC NORMAL

Table S4. Risk of Bias Assessment in individual studies

Adapted from: Office of Health Assessment and Translation (OHAT) Risk of Bias Rating Tool for Human and Animal Studies

Bias domains	Questions and criteria	Risk level assessment
**Exposure assessment**	This includes measurement error or measurement limitations.<br>**Major considerations are:**<br>• Is the exposure data source clearly described, not provided, or difficult to interpret?<br>• Were the temporal and spatial resolution of temperature measurements appropriate (e.g. more than one weather station, meteorological bureau)?<br>• daily temperature measurements were available?<br>• Were missing data present and/or imputed?<br>• Were selected lag(s) appropriate based on biological plausibility and other factors?	**Definitely low** risk of bias (++): There is direct evidence of low risk of bias practices (the true average population exposure at temperature has high confidence).<br>**Probably low** risk of bias (+): There is indirect evidence that the method assessment is robust, it is deemed that deviations from low risk of bias method for these criteria during the study would not appreciably bias results, including consideration of direction and magnitude of bias.<br>**Probably high** risk of bias (-): There is indirect evidence of high risk of bias practices, there is insufficient information (e.g., not reported or "NR"), or two out of the three listed considerations are not applied (e.g. use of single weather station).<br>**Definitely high** risk of bias (--): There is direct evidence of high risk of bias practices, or all listed **considerations** are not applied.
**Outcome assessment**	**This includes measurement error or measurement limitations.**<br>**Major considerations are:**<br>• outcome measurements were not influenced by knowledge of the exposure (data were from different databases)<br>• Was Laboratory-confirmed source for pathogen-specific studies and/or clinical diagnosis appropriate?<br>• Was selected date or timing of outcome appropriate (i.e. close to illness onset)<br>• Was missing data present and/or imputed?	**Definitely low** risk of bias **(++):** Outcome was Laboratory-confirmed pathogen-specific respiratory infection and the selected timing approximates the occurrence of symptoms.<br>**Probably low** risk of bias **(+):** Outcome was clinical diagnosis using standard criteria (e.g. WHO definition and International Classification System – ICD code) and the selected timing approximates the occurrence of symptoms.<br>**Probably high** risk of bias (-): outcome was not based on standard diagnosis criteria, there is evidence that suggests the existence of misclassification bias, or there is insufficient information (e.g. not reported) provided.<br>**Definitely high** risk of bias (--): Outcome was based on self-reports (e.g. parents or family members) and selected timing was inappropriate.
**Confounding bias**	**Major considerations are:**<br>Did the study appropriately adjusted/accounted for all important well studied potential confounders (e.g. time, seasonality, other weather parameters, and model specifications, lag effect)?<br>•Design or analysis accounted for potential confounding or modifying<br>•Consideration of other exposures that might bias results	**Definitely low** risk of bias (++): Study accounted for all important confounders which were measured consistently.<br>**Probably low** risk of bias (+): Study accounted for most of confounding and is not expected to introduce bias.<br>**Probably high** risk of bias (-): Study adjusted for some but not all of confounding and is expected to introduce bias.<br>**Definitely high** risk of bias (--): Study did not account for potential confounders or were inappropriately measured.
**Selection bias**	**Major considerations are:**<br>Was selection of participants into the study done in a manner that might introduce bias in the study?<br>• Was similar baseline characteristics, application of inclusion/exclusion criteria, recruitment strategy?<br>• Was all data available collected?<br>• Was there any form of randomisation of selection of individual cases?	**Definitely low** risk of bias (++): Similar baseline characteristics, the descriptions of the selection of participants were sufficiently detailed to support the assertion that risk of selection effects was minimal.<br>**Probably low** risk of bias (+): Insufficient information about selection of participants to permit a judgment of low risk of bias but there is indirect evidence that suggests low risk of bias.<br>**Probably high** risk of bias (-): Insufficient information about selection of participants to permit a judgment of high risk of bias and there is

Classified as ECDC NORMAL

		indirect evidence that suggests high risk of bias.<br>**Definitely high** risk of bias (--): Direct indications from descriptions of the participant selection of high risk of bias.
**Incomplete outcome data**	**Major considerations are:**<br>• missing data of outcome measures?<br>• missing data of exposures?	**Definitely low** risk of bias (++): no missing data present or missing data does not affect the real results<br>**Probably low** risk of bias (+): indirect evidence that suggests low risk of bias (e.g., <10% missing data, or missing data related to outcome).<br>**Probably high** risk of bias (-):indirect evidence that suggests high risk bias (e.g., ≥10% missing data without imputed using appropriate method, while rational for attrition explained in the study).<br>**Definitely high** risk of bias (--): missing outcome data are related to true outcome (e.g., substantial missing exposure data (≥10%), rationale for missing data not explained in the study).
**Selective reporting**	**Major considerations are:**<br>Was selection of participants into the study done in a manner that might introduce bias in the study?<br>• Selective reporting of entire studies, outcomes, or analyses.<br>• Systematic differences between reported and unreported findings.<br>• Can include potential for bias in reporting through source of funding	**Definitely low** risk of bias (++): Complete report of the entire study results and analysis.<br>**Probably low** risk of bias (+): There is not enough information to judge the risk of low bias, but there is indirect evidence of low risk of bias. (i.e., effect estimates presented for less than all hypotheses tested as per aims; but evidence suggests that effect estimates unlikely to be seriously biased).<br>**Probably high** risk of bias (-): There is not enough information to judge the risk of high bias, but there is indirect evidence of high risk of bias.<br>**Definitely high** risk of bias (--): There is a high risk of selectively reporting entire studies, results, or analyses.
**Conflict of interest**	**Major considerations are:**<br>Potential source of bias in reporting through source of funding<br>• Source of funding (government, academic institution, commercial institution, or no funding?<br>• Is conflict of interest declared?	**Definitely low** risk of bias (++): the study was not funded or funded by government or academic institutions or non-profit organizations, and the authors declare no conflict of interest<br>**Probably low** risk of bias (+): insufficient information to judge for low risk, but indirect evidence suggests study was free of financial interest<br>**Probably high** risk of bias (-): insufficient information to judge for high risk, but indirect evidence suggests study was not free of financial interest<br>**Definitely high** risk of bias (--): study was supported by a commercial institution with a financial interest in the findings, and the authors declare a conflict of interest)
**Other bias**	Bias due to other problems not covered elsewhere	**Definitely low** risk of bias (++): No other bias<br>**Probably low** risk of bias (+): insufficient information to judge for low risk, but indirect evidence suggests study was free of other problems<br>**Probably high** risk of bias (-): insufficient information to judge for high risk, but indirect evidence suggests study was not free of other problems<br>**Definitely high** risk of bias (--): at least one important risk of bias (e.g., selective reporting of subgroups, a potential source of bias related to the specific study design used, study has been claimed to have been fraudulent)

Classified as ECDC NORMAL

Table S5. Overall risk of bias rating

Overall Rating	Combinations (three key components of exposure assessment, outcome assessment, and confounding bias)
Definitely high (DH)	Definitely high + Definitely High + (Definitely high / Probably high / Probably low / Definitely low)<br>Definitely high + Probably high + Probably high
Probably high (PH)	Definitely high + Probably high + (Probably low / Definitely low)<br>Probably high + Probably high + (Probably high / Probably low/Definitely low)
Probably low (PL)	Probably high+(Probably low/Definitely low) + (Probably low / Definitely low)<br>Probably low + Probably low + Definitely Low
Definitely Low (DL)	Probably low + Definitely low + Definitely low<br>Definitely low + Definitely low + Definitely low

Classified as ECDC NORMAL

Table S6. Assessing confidence in the Body of Evidence (adapted from Johnson et al. 2014)

Evaluation factors	Criteria
Risk of bias	Study limitations include a significant risk of bias across the body of evidence.
Indirectness	The research evidence was not similar to the primary objective, i.e., participants, exposure, comparison, Outcome (PECO); Indirect evidence.
Inconsistency	High heterogeneity (I^2); variability in results; inconsistent magnitude and direction of effect magnitude.
Imprecision	Small sample size and wide confidence intervals.
Publication bias	The funnel plot is obviously asymmetrical;<br>Egger's test or Begg's tests show that publication bias exists.<br>Trim and Fill procedure was used to estimate potentially missing studies.
Large magnitude of effect	The rating was upgraded if modeling suggested that confounding alone was unlikely to explain associations that were judged to be of large magnitude.
Dose response	If dose-response relationships in one or more studies and/or dose-response across studies are consistent, the rating was upgraded.
Confounding minimizes effect	Upgraded if the consideration of all plausible residual confounders or biases would underestimate the effect or suggest a spurious effect when results show no effect.

From:: Johnson, P. I., Sutton, P., Atchley, D. S., Koustas, E., Lam, J., Sen, S., Robinson, K. A., Axelrad, D. A., & Woodruff, T. J. (2014). The Navigation Guide - evidence-based medicine meets environmental health: systematic review of human evidence for PFOA effects on fetal growth. Environmental health perspectives, 122(10), 1028–1039. https://doi.org/10.1289/ehp.1307893

Classified as ECDC NORMAL

Table S7. Strength of evidence definitions for human evidence according to the Navigation Guide (adapted from Johnson et al., 2014)

Strength rating	Definition
**Sufficient** evidence	A positive relationship is observed between exposure and outcome, where chance, bias, and confounding can be ruled out with reasonable confidence. The available evidence includes results from one or more well-designed, well conducted studies, and the conclusion is "unlikely to be strongly affected by the results of future studies."
**Limited** evidence	A positive relationship is observed between exposure and outcome, where chance, bias, and confounding cannot be ruled out with reasonable confidence. Confidence in the relationship is constrained by factors such as "the number, size, or quality of individual studies" or "inconsistency of findings across individual studies." As more information becomes available, the observed effect could change, and this change may be large enough to alter the conclusion.
**Inadequate** evidence	"The available evidence is insufficient to assess effects" of the exposure. The evidence is insufficient because of "the limited number or size of studies," low quality of individual studies, or "inconsistency of findings across individual studies." More information may allow an assessment of effects.
**Lack of** evidence	No relationship is observed between exposure and outcome; and chance, bias, and confounding can be ruled out with reasonable confidence. The available evidence includes consistent results from more than one well-designed, well conducted study at the full range of exposure levels that humans are known to encounter, and the conclusion is unlikely to be strongly affected by the results of future studies. The conclusion is limited to the age at exposure and/or other conditions and levels of exposure studied.

From:: Johnson, P. I., Sutton, P., Atchley, D. S., Koustas, E., Lam, J., Sen, S., Robinson, K. A., Axelrad, D. A., & Woodruff, T. J. (2014). The Navigation Guide - evidence-based medicine meets environmental health: systematic review of human evidence for PFOA effects on fetal growth. Environmental health perspectives, 122(10), 1028–1039. https://doi.org/10.1289/ehp.1307893

Classified as ECDC NORMAL

Table S8. Full texts excluded on basis of eligibility

Reference	Title	Reason
Sohn S 2019	'Pneumonia Weather': Short-term Effects of Meteorological Factors on Emergency Room Visits Due to Pneumonia in Seoul, Korea	Target the disease, not the pathogen
Bishop-Williams KE 2017	A protocol for a systematic literature review: comparing the impact of seasonal and meteorological parameters on acute respiratory infections in Indigenous and non-Indigenous peoples	Effect estimates are not applicable
Chen J 2021	Ambient Temperature Is an Independent Risk Factor for Acute Tonsillitis Incidence	Target the disease, not the pathogen
Wenfang G	Assessing the effects of meteorological factors on daily children's respiratory disease hospitalizations: A retrospective study	Effect estimates are not applicable
Zhang Lei 2017	Analysis of Mycoplasma pneumoniae infection among children with respiratory tract infection in hospital in Chengdu from 2013 to 2015	Outcome not applicable
Zhang L 2021	Analysis of mycoplasma pneumoniae infection among children with respiratory tract infections in hospital in Chengdu from 2014 to 2020	Outcome not applicable
Fang J 2021	Association between ambient temperature and childhood respiratory hospital visits in Beijing, China: a time-series study (2013-2017)	Target the disease, not the pathogen
Liu Y 2015	Association between Temperature Change and Outpatient Visits for Respiratory Tract Infections among Children in Guangzhou, China	Effect estimates are not applicable
Ruchiraset A 2020	Association of climate factors and air pollutants with pneumonia incidence in Lampang province, Thailand: findings from a 12-year longitudinal study	Target the disease, not the pathogen
Zhang Y 2015	Burden of respiratory syncytial virus infections in China: Systematic review and meta-analysis	Outcome not applicable
Ashmita Gosai 2009	Climate and respiratory disease in Auckland, New Zealand	Target the disease, not the pathogen
Ghia C 2021	Disease Burden Due to Respiratory Syncytial Virus in Indian Pediatric Population: A Literature Review.	Outcome not applicable
Álvaro-Meca A 2022	Environmental factors linked to hospital admissions in young children due to acute viral lower respiratory infections: A bidirectional case-crossover study	Focusing on lower respiratory tract infections
Falagas ME 2008	Effect of meteorological variables on the incidence of respiratory tract infections	Target the upper and lower respiratory tract infections
Jang JY 2021	Effect of diurnal temperature range on emergency room visits for acute upper respiratory tract infections	Improper temperature
Xie MY 2017	Effect of diurnal temperature range on the outpatient visits for acute bronchitis in children: a time-series study in Hefei, China	Target the disease
Álvaro-Meca A 2022	Environmental factors linked to hospital admissions in young children due to acute viral lower respiratory infections: A bidirectional case-crossover study	Target the lower respiratory tract infection
Li Y 2019	Global patterns in monthly activity of influenza virus, respiratory syncytial virus, parainfluenza virus, and metapneumovirus: a systematic analysis	Outcome not applicable
Hossain MZ 2019	Sociodemographic, climatic variability and lower respiratory tract infections: a systematic literature review	Outcome not applicable
Choe YJ 2019	Seasonality of respiratory viruses and bacterial pathogens	Not specific to bacteria
Nastos PT 2006	Weather impacts on respiratory infections in Athens, Greece	Target the disease
Simmering JE 2017	Weather-Dependent Risk for Legionnaires' Disease, United States	Outcome not applicable
TNS 2012	The seasonal variations of respiratory syncytial virus infections in Turkey: a 2-year epidemiological study	Outcome not applicable
Gleason JA 2016	Under the Weather: Legionellosis and Meteorological Factors	Outcome not applicable
Karagiannis I 2009	Warm, wet weather associated with increased Legionnaires' disease incidence in The Netherlands	Target the disease, not the pathogen

Classified as ECDC NORMAL

Brandsema PS 2014	Summer increase of Legionnaires’ disease 2010 in The Netherlands associated with weather conditions and implications for source findin	Target the disease, not the pathogen
Han XY 2019	Solar and Climate Effects Explain the Wide Variation in Legionellosis Incidence Rates in the United States	Outcome not applicable/Temperature not applicable
Conza L 2013	Meteorological factors and risk of community-acquired Legionnaires’ disease in Switzerland: an epidemiological study	Temperature not applicable
Fisman DN 2005	It's not the heat, it's the humidity: wet weather increases legionellosis risk in the greater Philadelphia metropolitan area	Outcome not applicable
Ng V 2008	Going with the flow: legionellosis risk in Toronto, Canada is strongly associated with local watershed hydrology	Temperature not applicable
Yuan L 2011	Relationship between Influenza and Meteorological Conditions in Tianjin	Target the disease, not the pathogen
Paynter S 2013	Sunshine, rainfall, humidity and child pneumonia in the tropics: time-series analyses	Temperature not applicable
WU Zhi-qiang 2022	A single-center study on the relationship between meteorological factors and the number of visits for respiratory diseases in children	Outcome not applicable
JIAN Wanlin 2023	A Stratified Comparative Study on the Meteorological Factors Impacting Respiratory Diseases in the Two Counties of Eastern and Western China	Target the disease, not the pathogen
Paynter S 2014	Respiratory syncytial virus seasonality in tropical Australia	effect estimates not applicable
Michelozzi P 2009	High temperature and hospitalizations for cardiovascular and respiratory causes in 12 european cities	effect estimates not applicable
Shao Lin 2009	Extreme high temperatures and hospital admissions for respiratory and cardiovascular diseases	Outcome not applicable
Habeebullah TM 2021	Impact of outdoor and indoor meteorological conditions on the COVID-19 transmission in the western region of Saudi Arabia	did not have convertible estimates
Donzelli G 2022	Role of meteorological factors on SARS-CoV-2 infection incidence in Italy and Spain before the vaccination campaign. A multi-city time series study	effect estimates not applicable
Kaplin A 2021	Evidence and magnitude of the effects of meteorological changes on SARS-CoV-2 transmission	did not have convertible estimates
Mandal CC 2022	Combinatorial influence of environmental temperature, obesity and cholesterol on SARS-CoV-2 infectivity	did not have convertible estimates
Li Ruiying 2018	Study of the Influence of Meteorological Condition on Children Lower Respiratory Tract Infection and the Prediction Model in Qinhuangdao	Outcome not applicable
Dufloo J 2024	Temperature impacts SARS-CoV-2 spike fusogenicity and evolution	did not have convertible estimates
Thi Khanh HN 2023	The impact of ambient temperature and air pollution on SARS-CoV2 infection and Post COVID-19 condition in Belgium (2021-2022)	Under the combined influence of ambient temperature and air pollution/Not a single temperature effect
Kim J 2023	How Does Climate Change Affect the Upper Airway?	No effect estimation
Grover EN 2024	Does behavior mediate the effect of weather on SARS-CoV-2 transmission? Evidence from cell-phone data	No effect estimation
Lee H 2024	Impact of ambient temperature on respiratory disease: a case-crossover study in Seoul	Target the disease, not the pathogen
Maciorowski D 2021	Environmental factors and their role in the transmission of SARS-CoV-2. Biosaf Health.	No effect estimation
Kaplin A 2021	Evidence and magnitude of the effects of meteorological changes on SARS-CoV-2 transmission	effect estimates not applicable
Nichols GL 2021	Coronavirus seasonality, respiratory infections and weather	Temperature not applicable
Tian Y 2023	Ambient temperature variability and hospital admissions for pneumonia: A nationwide study	Target the disease, not the pathogen
Makrufardi F 2024	Extreme temperatures increase the risk of pediatric pneumonia: a systematic review and meta-analysis	Target the disease, not the pathogen

Classified as ECDC NORMAL

Miyayo SF 2021	Analysis of Pneumonia Occurrence in Relation to Climate Change in Tanga, Tanzania	Outcome not applicable

Classified as ECDC NORMAL

Table S9. Basic characteristics of 137 included studies

Study	Location	Study period	Time sample	Population	Ages	Reporting frequency	Climate	Case source	Exposure (°C)	Pathogen	Income	Overall bias
Raymundo (2023)[1]	Oahu, USA	2016.05-2019.05	37	7143	<6 y	Monthly	A	Reported cases	T_{mean}	EV/HRV	High	PL
Annabel (2023)[2]	Bukit Timah, Singapore	2009-2019	4016	7329	<5 y	Daily	A	Influenza infection	T_{mean}	IVA, IVB	High	DH
Chee (2023)[3]	Kuala Lumpur, Malaysia	2017-2021	60	2950	<12 y	Monthly	A	Hospitalized for ARI	T_{mean}	RSV	Upper-middle	PH
Keita (2023)[4]	47 prefectures in Japan	2014-2019	312	721709	all	Weekly	C	Reported cases	T_{mean}	RSV	High	PL
Ping-Ing (2023)[5]	Taiwan, China	1995-2005, 2000-2005	206	1740	<18 y	Monthly	A/C	Reported bronchiolitis/pneumonia	T_{mean}	RSV	High	PL
Meng (2023)[6]	Singapore	2009-2019	4016	15715	<5 y	Daily	A	Reported cases	T_{mean}, T_{max}, T_{min}	RSV	High	PL
You (2022)[7]	Gwangju, Korea	2016-2019	48	4195	all	Monthly	D	Hospitalized with ARI	T_{mean}	IV, HAdV, PIV, RSV, HCoV, HRV, HBoV, HMPV, EV	High	PH
Zhang (2022)[8]	Jiangyin, China	2015-2020	72	12294	28 d-14 y	Monthly	C	Hospitalized for ARTI	T_{mean}	RSV, IVA, IVB, HPIV 1-3, HAdV	Upper-middle	PH
Yuan (2022)[9]	Chendu, China	2016-2019	48	5127	28 d-14 y	Monthly	C	Hospitalized for ARI	T_{mean}	*M. pneumoniae*	Upper-middle	PH
Na (2022)[10]	Nantong, China	2017-2019	156	51665	<14 y	Weekly	C	Reported pneumonia cases	T_{mean}	*M. pneumoniae*	Upper-middle	PL
Pan (2022)[11]	Shenzhen, China	2009-2015	2555	5744	all	Daily	C	Influenza	T_{mean}, T_{max}, T_{min}	IVA, IVB	Upper-middle	DL
Huang (2022)[12]	Suzhou, China	2016-2019	48	7940	1 m-15 y	Monthly	C	Hospitalized for ARI	T_{mean}	*H. influenzae*	Upper-middle	PH
Ming (2022)[13]	Wenzhou, China	2008-2017	121	89898	1–3 y	Monthly	C	Hospitalized with ARI	T_{mean}	HPIV-3	Upper-middle	PL
You (2022)[14]	13 European countries	2010-2019	521	30965	<5 y	Weekly	Multiple	Hospitalized with ARI	T_{mean}	RSV	High	DH
Sung (2022)[15]	Changwon, Korea	2020.1.20-04.29	100	3234	all	Daily	C	Reported cases	T_{mean}	SARS-CoV-2	High	PL

Classified as ECDC NORMAL

Lisa (2022)[16]	Helsinki, Finland	2020.08.01-2021.05.31	62	48013	all	Daily	C	Reported cases	T_{mean}	SARS-CoV-2	High	PL
Keita (2022)[17]	Six Japanese prefectures	2020.05-2022.03	699	2982262	all	Daily	C	Notified cases	T_{mean}	SARS-CoV-2	High	PH
Cheng (2022)[18]	Macao, China	2014-2017	1460	4880	1 m-14 y	Daily	C	Hospitalized for ARI	T_{mean}	EV/HRV, HAdV, RSV-A, RSV-B, IVA, IVB, HPIV, HMPV, HBoV, HCoV	High	DL
Zhi (2021)[19]	Suzhou, China	2016-2018	36	1157	1-15 y	Monthly	C	Hospitalized with LRTI	T_{mean}	RSV	Upper-middle	DH
Eun (2021)[20]	Cheonan, Korea	2012-2018	2556	9010	1–9 y	Daily	D	Reported RI	T_{mean}	HPIV 1-3	High	PL
Aleix (2021)[21]	52 Spanish provinces	Weeks 10-16 of 2020	7	2418250	all	Weekly	C	Reported cases	T_{mean}, T_{max}, T_{min}	SARS-CoV-2	High	PL
Liu (2021)[22]	Moscow	2020.03.11-06.22	103	Not reported	all	Daily	D	Reported cases	T_{mean}, T_{max}, T_{min}	SARS-CoV	High	PH
Na (2021)[23]	Tianjin, China	2015.06-2019.02	44	63821	0-16 y	Monthly	D	Hospitalized for ARI	T_{mean}	*M. pneumoniae*	Upper-middle	DL
Wu (2021)[24]	Haikou, China	2018-2020	36	309	5.05 m	Monthly	A	Hospitalized for RTI	T_{mean}	RSV, RSV-A, RSV-B	Upper-middle	PL
Chen (2021)[25]	Guangdong, China	2020.01.21-02.26	36	1347	all	Daily	C	Reported cases	T_{mean}	SARS-CoV-2	Upper-middle	PL
Stacy (2021)[26]	Singapore	2009-2019	4016	6393	<5 y	Daily	A	Reported cases	T_{mean}	HPIV	High	PL
Eun (2021)[27]	Cheonan, Korea	2012-2018	85	9010	<10 y	Monthly	D	Reported RTI	T_{mean}	HAdV	High	PL
Can (2021)[28]	China	2004-2017	522	1986536	all	Weekly	C/D	Reported cases	T_{mean}	IVA/H1N1pdm09, IVA/H3N2, IVB	Upper-middle	PH
Chan (2021)[29]	Hong Kong, China	1993-1997	60	9635	<5 y	Monthly	C	Admission with RI	T_{mean}	RSV	High	PL
Dong (2021)[30]	Cheonan, Korea	2012-2018	85	1920	all	Monthly	D	Hospital with RI	T_{mean}	HRV	High	DH
Therese (2021)[31]	Nairobi, Kenya	2007–2013	85	17261	all	Monthly	A	Reported influenza-like illness	T_{mean}	RSV, HPIV 1-3, HAdV	Upper-middle	PL
Lei (2021)[32]	Chengdu, China	2014-2020	85	22882	1 m-17 y	Monthly	C	Hospitalized with RTI	T_{mean}	*M. pneumoniae*	Upper-middle	PL
Mohamed (2021)[33]	Safat, Kuwait	2020.02.24-05.30	96	176	all	Daily	A	Reported cases	T_{mean}	SARS-CoV-2	High	PH

Classified as ECDC NORMAL

Liu (2021)[34]	Chongqing, China	2009.06-2019.06	3681	3107	1 m-18 y	Daily	C	Hospitalized for ARI	T_{mean}, T_{max}, T_{min}	RSV	Upper-middle	PL
Rosalie (2021)[35]	Amsterdam, The Netherlands	2003-2016	170/730	2161	≤ 24 m	Monthly/ Weekly	C	Admissions for bronchiolitis	T_{mean}, T_{max}, T_{min}	RSV	High	PL
Jang (2020)[36]	Cheonan, Korea	2012-2018	2556	9010	<10 y	Daily	D	Reported cases	T_{mean}	HCoV- 229E, HCoV-OC43	High	PL
Ilada (2020)[37]	Bangkok, Thailand	2012-2018	85	8209	≤ 5 y	Monthly	A	Reported ILI cases	T_{mean}	RSV	Upper-middle	PH
Asmaa (2020)[38]	Riyadh, Saudi Arabia	2012.10-2018.12	75	712	all	Monthly	A	Reported cases	T_{mean}	MERS-CoV	High	DL
Sheikh (2020)[39]	Bukit Timah, Singapore	2005-2015	4016	9905	< 30 m	Daily	A	Reported cases	T_{mean}, T_{max}, T_{min}	RSV	High	PL
Shiv (2020)[40]	Fifty U.S. states and Washington D.C.	2020.1.22-2020.3.4	42	974	all	Daily	Multiple	Reported cases	T_{max}	SARS-CoV-2	High	PL
Zhang (2020)[41]	Suzhou, China	2014-2017	48	7525	2.51±15.93 m	Monthly	C	Hospitalized for CAP	T_{mean}	HPIV- 3	Upper-middle	PH
Simone (2020)[42]	Milan; Florence; Trento, Italy	2020.03.08-2020.06.19	103	Not report ed	all	Daily	C/D	Reported cases	T_{mean}, T_{max}, T_{min}	SARS-CoV-2	High	PL
Kong (2020)[43]	Wuhan, China	2020.01.20-02.11	22	Not report ed	all	Daily	C	Reported cases	T_{mean}	SARS-CoV-2	Upper-middle	PH
Giselmo (2020)[44]	Maranhao, Brazil	2018.04-2019.03	12	151	2–12 y	Monthly	A	Hospitalized with RTI	T_{mean}, T_{max}, T_{min}	RSV, HRV	Upper-middle	PL
Li (2020)[45]	Shanghai, China	2017.07-2019.06	104	29135	0-18 y	Weekly	C	Reported cases	T_{mean}, T_{max}, T_{min}	IVA, IVB	Upper-middle	PH
Rory (2019)[46]	Edinburgh, Scotland, UK	2009.04-2015.11	81	52060	all ages	Monthly	C	Hospitalized with RTI	T_{mean}	HPIV 1-3, RSV, HMPV, IVA, IVB, HRV, HAdV	High	PL
Huang (2019)[47]	Zhongshan, China	2014.09-2016.08	24	13705	1 m-14 y	Monthly	C	Hospitalized with CAP	T_{mean}	*M. pneumoniae, C. pneumoniae, M. catarrhalis*, IVA, IVB, HAdV, HPIV, RSV, *H. influenzae, S.pneumoniae*	Upper-middle	PH
Pan (2019)[48]	Panzhihua, China	2006-2015	121	6892	all	Monthly	C	Influenza infection	T_{mean}	IVA, IVB	Upper-middle	DL

Classified as ECDC NORMAL

He (2019)[49]	Suzhou, China	2012-2015	48	8711	＜18 y	Monthly	C	Hospitalized for RTI	T_{mean}	HBoV	Upper-middle	PL
Wu (2019)[50]	Wu zhishan, China	2015-2016	24	1597	1 m-14 y	Monthly	A	Hospitalized for ARI	T_{mean}	*M. pneumoniae*	Upper-middle	PH
Wei (2019)[51]	Jinan, China	2013-2016	208	9170	all	Weekly	D	Reported cases	T_{mean}	IVA/H1N1pdm09, IVA/H3N2, IVB	Upper-middle	PL
Yong (2019)[52]	Seoul, Korea	2007- 2016	521	23694	<18 y	Weekly	D	Hospitalized for ARI	T_{mean}, T_{max}, T_{min}	HMPV	High	PL
Katerina (2019)[53]	Liverpool, UK	2011.11-2017.03	65	374	18-50 y	Monthly	C	Reported cases	T_{mean}, T_{max}, T_{min}	*S. pneumoniae*	High	PL
Magali (2018)[54]	Dijon, France	2011-2016	90	4300	all	Weekly	C	Hospitalized for ARI	T_{mean}	HMPV, RSV	High	PL
Wen-Kuan (2018)[55]	Guangzhou, China	2009.07-2016.06	85	11399	≤14 y	Monthly	C	Hospitalized for ARI	T_{mean}	HBoV-1	Upper-middle	PL
Morley (2018)[56]	Gold Coast, Australia	2007.07-2016.06	469	15387	<5 y	Weekly	C	Hospitalized with ARI	T_{mean}, T_{max}, T_{min}	RSV	High	PL
Hermann (2018)[57]	Garoua, Cameroon	2014-2016	36	1666	0-5 y	Monthly	D	Reported influenza cases	T_{mean}	IVA, IVB	Lower-middle	PL
Ines (2018)[58]	Sousse, Northern Africa	2003–2015	158	5131	≤ 5 y	Monthly	B	Hospitalized for bronchiolitis	T_{mean}	RSV	Lower-middle	PL
Jung (2018)[59]	Seoul, Korea	2005-2012	97	9113	< 3 y	Monthly	D	Hospitalized with bronchiolitis/pneumonia	T_{mean}	RSV	High	PL
Adriana (2018)[60]	Toronto, Canada	2010-2015	312	44362	1 m-108 y	Weekly	D	Reported cases	T_{mean}	IVA, IVB	High	PL
Benjamin (2018)[61]	Nha Trang, central Vietnam	2007.01.29-2012.4.26	63	2998	all	Monthly	A	Hospitalized with ARI	T_{mean}	IVA, IVB, RSV, HMPV, HPIV 1-4, HRV, HCoV-229E, HCoV-OC43, HAdV, HBoV	Lower-middle	PL
Zhou (2018)[62]	Suzhou, China	2013-2015	36	5994	17 d-15 y	Monthly	C	Hospitalized for ARI	T_{mean}	HRV	Upper-middle	DH
Jang (2017)[63]	Cheonan, Korea	2006.12-2014.02	88	6279	<1 y	Monthly	D	Reported cases	T_{mean}	RSV	High	PL
Terezinha (2017)[64]	Sao Paulo, Brazil	1996-2010	182	Not reported	<5 y	Monthly	C	Hospitalized with bronchiolitis/pneumonia	T_{mean}	RSV	Upper-middle	PL

Classified as ECDC NORMAL

Wang (2017)[65]	Suzhou, China	2006-2013	97	15098	1 m-14 y	Monthly	C	Hospitalized with ARI	T_{mean}	*M. pneumoniae*	Upper-middle	PL
Gwladys (2017)[66]	Yaounde´, Cameroon	2009.01-2015.10	360	5216	all	Weekly	A	Reported ILI/ARI cases	T_{mean}	IVA, IVB	Lower-middle	DH
Yan (2017)[67]	Suzhou, China	2013-2015	36	6194	<14 y	Monthly	C	Hospitalized with LRTI	T_{mean}	HRV	Upper-middle	PL
Tian (2017)[68]	Hangzhou, China	2015	365	36500	4.3-52.8 m	Daily	C	Reported pneumonia cases	T_{mean}, T_{max}, T_{min}	RSV, *M. pneumoniae*	Upper-middle	PH
Zhou (2017)[69]	Huangshi, China	2016	12	2326	1 m-14 y	Monthly	C	Hospitalized for ARI	T_{mean}	HRV	Upper-middle	PL
Raffaella (2017)[70]	Rome, Italy	2004-2014	133	723	< 1 y	Monthly	C	Hospitalized for acute bronchiolitis	T_{mean}	RSV	High	PL
Wei (2016)[71]	Shanghai, China	2011.08-2014.12	41	2819	2 m-12 y	Monthly	C	Hospitalized with ARI	T_{mean}	IFV, HPIV 1-4, EV, RSV, HCoV, HAdV, HMPV, HBoV, HRV	Upper-middle	PL
Qing (2016)[72]	Hangzhou, China	2015	365	36500	4.3 m	Daily	C	Hospitalized with ARI	T_{mean}, T_{max}, T_{min}	RSV	Upper-middle	DH
PSOTER (2016)[73]	48 states of USA	2003-2009	2556	3463	<7 y	Daily	A/B/C/D	Reported cases	T_{mean}	*P.aeruginosa*	High	PH
Nicklas (2016)[74]	Gothenburg, Sweden	2010.10-2013.07	147	20062	<18 y	Weekly	C	Hospitalized for RTI	T_{mean}	IVA, IVB, RSV, HRV, EV, HCoV, HMPV, HAdV, HPIV 1–4, HBoV, *C. pneumoniae, M. pneumoniae*	High	DH
Shi (2016)[75]	Zhengzhou, China	2012.01-2015.01	36	2323	18-94 y	Monthly	D	Hospitalized for ARI	T_{mean}	*M. pneumoniae*	Upper-middle	PH
Chen (2016)[76]	Yancheng, China	2014-2015	24	3672	1 m-10 y	Monthly	C	Hospitalized for ARI	T_{mean}	RSV, IVA, IVB, HPIV 1-3, HAdV	Upper-middle	PH
Sobral (2020)[77]	USA	2019.12-2020.03	118	Not reported	all	Daily	Multiple	Reported cases	T_{mean}, T_{max}, T_{min}	SARS-CoV-2	High	PL
Jaqueline (2016)[78]	Curitiba, Brazil	2012-2013	24	755	all	Monthly	C	Hospitalized for ARI	T_{mean}	HRV	Upper-middle	PL
Tiina (2016)[79]	Kajaani, Finland	2004.07-2005.01	214	386	all	Daily	C	Reported cases	T_{mean}	HRV	High	DH

Classified as ECDC NORMAL

Gamba-Sanchez (2016)[80]	Bogota, Colombia	2009-2013	60	13488	<3 y	Monthly	C	Reported cases	T_{mean}	RSV	Upper-middle	DL
Rodriguez-Martinez (2015)[81]	Bogota, Colombian	2010.01-2011.04	16	3931	<3 y	Monthly	C	Hospitalized for ARI	T_{mean}	RSV	Upper-middle	DL
Geng (2015)[82]	Suzhou, China	2001-2011	133	42664	3 d-15 y	Monthly	C	Hospitalized for ARI	T_{mean}	RSV	Upper-middle	PL
Daniel (2015)[83]	Mallorca, Spain	2006-2011	312	60659	1– 15 y	Weekly	C	Reported pharyngitis cases	T_{mean}	*S. pyogenes*	High	PL
Lu (2015)[84]	Suzhou, China	2010-2014	60	1803	≤28 d	Monthly	C	Hospitalized with LRTI	T_{mean}	RSV	Upper-middle	PL
Cui (2015)[85]	Chaoshan, China	2010.12-2011.11	364	1074	0–16 y	Daily	C	Hospitalized for ARI	T_{mean}	RSV, IVA, IVB, HCoV, HMPV, HPIV 1-4, HRV, EV, HAdV, HBoV	Upper-middle	DH
Lin (2015)[86]	Quanzhou, China	2013	12	6020	0-15 y	Monthly	C	Hospitalized for ARI	T_{mean}	RSV, HAdV, IVA, IVB, HPIV, *M. pneumoniae, C.pneumoniae, L. pneumophila*	Upper-middle	PL
Yan (2015)[87]	Shanghai, China	2011-2013	36	2526	0-12 y	Monthly	C	Hospitalized for ARI	T_{mean}	HPIV 1-4	Upper-middle	PL
Huang (2015)[88]	Suzhou, China	2013	12	1926	1-140 m	Monthly	C	Hospitalized for ARI	T_{mean}	HRV	Upper-middle	PH
Ni (2014)[89]	Suzhou, China	2006-2011	72	10596	1-13 y	Monthly	C	Hospitalized for ARI	T_{mean}	*M. pneumoniae*	Upper-middle	PL
Sun (2014)[90]	Suzhou, China	2006-2010	60	8143	1-180 m	Monthly	C	Hospitalized with ARI	T_{mean}	*M. catarrhalis*	Upper-middle	PL
Onozuka (2014)[91]	Fukuoka, Japan	2006-2012	365	30215	all	Weekly	C	Reported cases	T_{mean}, T_{max}, T_{min}	RSV	High	PH
Zheng (2014)[92]	Suzhou, China	2001-2011	133	42104	1 m-14 y	Monthly	C	Hospitalized with ARI	T_{mean}	RSV, IVA, IVB, HPIV 1-3, HAdV	Upper-middle	PH
Chen (2014)[93]	Suzhou, China	2009-2012	48	7626	<14 y	Monthly	C	Hospitalized with LRTI	T_{mean}	HBoV	Upper-middle	PL
Chen (2013)[94]	Suzhou, China	2006	12	1598	1 m-13 y	Monthly	C	Hospitalized for ARI	T_{mean}	*M. pneumoniae, C. pneumoniae*	Upper-middle	PL
Tang (2013)[95]	Suzhou, China	2009	12	1883	1 m-10 y	Monthly	C	Hospitalized for RTI	T_{mean}	RSV	Upper-middle	PH

Classified as ECDC NORMAL

Wan (2013)[96]	Suzhou, China	2007-2011	60	28871	3 d-15 y	Monthly	C	Reported cases	T_{mean}	RSV	Upper-middle	PH
Wang (2013)[97]	Guilin, China	2011-2012	24	1342	30 d-14 y	Monthly	C	Hospitalized for ARI	T_{mean}	*M. pneumoniae*	Upper-middle	PL
Zheng (2013)[98]	Luoyang, China	2011	12	256	8-23 m	Monthly	D	Hospitalized for ARI	T_{mean}	*M. pneumoniae*	Upper-middle	PL
Yin (2013)[99]	Suzhou, China	2006-2009	48	8368	1 m-14 y	Monthly	C	Hospitalized for ARI	T_{mean}	*M. pneumoniae*	Upper-middle	PL
Chen (2013)[100]	Suzhou, China	2006-2010	60	8157	1 m-14 y	Monthly	C	Hospitalized with RTI	T_{mean}	*M. pneumoniae*	Upper-middle	DH
Li (2013)[101]	Zhuhai, China	2010	12	924	1 m-78 y	Monthly	C	Reported ILI cases	T_{mean}	IVA/H3N2, IVA/H1N1pdm09, IVB, HPIV 1-3, RSV, HMPV, HAdV	Upper-middle	PL
Wang (2013)[102]	Suzhou, China	2006 - 2009	48	6655	30 d-10 y	Monthly	C	Hospitalized for ARI	T_{mean}	HMPV	Upper-middle	PL
Chen (2012)[103]	Suzhou, China	2009-2010	24	998	1-24 m	Monthly	C	Hospitalized for bronchiolitis	T_{mean}	RSV, HBoV, HPIV-3, IVA, HAdV, HMPV, *M. pneumoniae*	Upper-middle	PH
Chee-Sieng (2012)[104]	Kuala Lumpur, Malaysia	1982-2008	328	10269	≤5 y	Monthly	A	Hospitalized with RTI	T_{mean}	RSV	Upper-middle	PL
Chen (2012)[105]	Suzhou, China	2006-2010	60	8197	1-120 m	Monthly	C	Hospitalized for ARI	T_{mean}	HAdV	Upper-middle	PH
TNS (2012)[106]	Turkey four regions	2008.05-2010.09	29	3464	<2 y	Monthly	C/D	bronchiolitis/pneumonia	T_{mean}	RSV	Upper-middle	PL
Daniel Hervás (2012)[107]	Mallorca, Spain	1995-2006	146/625	2384	≤ 2 y	Monthly/ Weekly	C	Hospitalized for bronchiolitis	T_{mean}, T_{max}, T_{min}	RSV	High	PL
Susana (2012)[108]	Valencia, Spain	2006-2009	48	243	> 18 y	Monthly	C	Hospitalized for CAP	T_{mean}	*S.pneumoniae, L. pneumophila*	High	PH
Ji (2011)[109]	Suzhou, China	2006-2009	48	6655	1 m -10 y	Monthly	C	Hospitalized for ARI	T_{mean}	RSV, IVA, IVB, HPIV 1-3, HAdV, HMPV, HBoV	Upper-middle	PL
Sun (2011)[110]	Suzhou, China	2006-2009	48	6655	1-120 m	Monthly	C	Hospitalized for RI	T_{mean}	HPIV-3	Upper-middle	PH
Wang (2011)[111]	Suzhou, China	2006-2009	48	6599	1-120 m	Monthly	C	Hospitalized for ARI	T_{mean}	HMPV	Upper-middle	PH
Loh (2011)[112]	Bukit Timah, Singapore	2003.08–2008.12	65	44026	0–5 y	Weekly	A	Hospitalized with RTI	T_{mean}, T_{max}, T_{min}	IVA, IVB, RSV, HPIV 1–3, HAdV	High	PL
Cristiana (2010)[113]	Salvador, Brazil	2003.09-2005.05	20	184	<5 y	Monthly	A	Reported pneumonia cases	T_{mean}	IVA, IVB, RSV, HPIV 1-3, HAdV, *H.*	Upper-middle	PL

Classified as ECDC NORMAL

										influenzae, S. pneumoniae, M. pneumoniae, C. pneumoniae, M. catarrhalis		
Cao (2010)[114]	Beijing, China	2009.08.03-11.08	97	Not reported	all	Daily	D	Reported ILI cases	T_{mean}	IVA/H1N1pdm09	Upper-middle	PH
Jean-Baptist (2009)[115]	Rheinland-Pfalz, Mainz	2001-2006	312	3044	≤16 y	Weekly	C	Hospitalized for ARI	T_{mean}	IVA, HAdV, HMPV, HRV, CoV, *M. pneumoniae, C. pneumoniae*	High	PH
Noyola (2009)[116]	San Luis Potosí, Mexico	2002.10-2006.05	191	1393	<18 y	Weekly	D	Hospitalized with LRTI	T_{mean}	RSV	High	PH
Maria (2009)[117]	Kathmandu, Nepal	2004.06-2007.06	37	887	2-35 m	Monthly	A	Reported pneumonia cases	T_{mean}	RSV, IVA, IVB, HPIV 1-3, HMPV	Lower	PL
Onozuka (2009)[118]	Fukuoka,Tokyo, Japan	1999-2007	469	13056	<15 y	Weekly	C	Reported pneumonia cases	T_{mean}	*M. pneumoniae*	High	PL
Tang (2009)[119]	Hong Kong, China	2000.05–2007.12	93	8539	<18 y	Monthly	C	Hospitalized for RTI	T_{mean}	IVA, IVB, RSV	High	PL
Onozuka (2008)[120]	Fukuoka, Japan	1999-2007	432	13056	all	Weekly	C	Reported cases	T_{mean}	*M. pneumoniae*	Upper-middle	PH
Asma (2008)[121]	Doha, Qatar	2002.01-2007.09	69	3121	≤ 2 y	Monthly	A	Reported cases	T_{mean}	RSV	High	PL
Yuan (2006)[122]	Beijing, China	2003.04.03 -06.11	69	2522	all	Daily	C	Reported cases	$T_{mean,}$ T_{max}, T_{min}	SARS-CoV-2	Upper-middle	PL
Bi (2006)[123]	Beijing and Hong Kong, China	2003.04.21-05.20	29	Not reported	all	Daily	C/D	Notified cases	$T_{mean,}$ T_{max}, T_{min}	SARS-CoV	Upper-middle	PL
Wang (2005)[124]	Hangzhou, China	2001-2003	36	13642	1 m-13 y	Monthly	C	Hospitalized for pneumonia	T_{mean}	RSV	Upper-middle	PL
Feng (2005)[125]	Guangzhou, Hong Kong, Toronto, Singapore, Taiwan, Beijing	2003.01.01-05.18	137	581	all	Daily	A/C/D	Reported cases	$T_{mean,}$ T_{max}, T_{min}	SARS-CoV	Upper-middle/High	PH
Mariana (2004)[126]	Buenos Aires, Argentina	1998 –2002	60	18561	<5 y	Monthly	C	Hospitalized with ARI	T_{mean}	RSV, HPIV, IVA, IVB, HAdV	High	PL

Classified as ECDC NORMAL

Huang (2004)[127]	Guangdong, China	2003.01-05	129	1491	all	Daily	C	Reported cases	T_{mean}, T_{max}, T_{min}	SARS-CoV	Upper-middle	PH
Hailin (2019)[128]	Wenzhou, China	2008-2017	121	89898	<18 y	Monthly	C	Hospitalized with RTI	T_{mean}	RSV	Upper-middle	PL
Liu (2019)[129]	Guangzhou, China	2009.07-2016.06	85	11398	≤14 y	Monthly	C	Hospitalized with ARI	T_{mean}	RSV, HPIV, HMPV	Upper-middle	DL
Natalia (2016)[130]	Athens, Greece	2002-2013	146	7516	0-14 y	Monthly	C	Hospitalized with ARI	T_{mean}	RSV	High	PH
Omer (2008)[131]	Lombok, Indonesia	2000-2002	1095	2878	< 2 y	Daily	A	Reported pneumonia cases	T_{mean}, T_{max}, T_{min}	RSV	Upper-middle	DH
Patrick (2015)[132]	Kuala Lumpur, Malaysia	1982-1997	194	5691	< 2 y	Monthly	A	Reported bronchiolitis and pneumonia	T_{mean}	RSV	Upper-middle	DL
Santiago (2004)[133]	Leon, Spain	1995.10-2000.06	247	221	< 18 y	Weekly	C	Hospitalized with bronchitis/bronchiolitis/pneumonia	T_{mean}, T_{max}, T_{min}	RSV	High	PH
Silvia (2013)[134]	Bologna, Italy	2007-2010	208	327	< 2 y	Weekly	C	Reported cases	T_{min}	RSV	High	PL
Virginia (2015)[135]	Nine states (Arizona, California, Colorado, Iowa, Massachusetts, Maryland, New Jersey, Washington, and Wisconsin), United States	1989-2009	255	Not reported	all	Monthly	C	Reported acute bronchiolitis	T_{mean}, T_{max}, T_{min}	RSV	High	PL
Zhang (2013)[136]	Suzhou, China	2001-2011	133	42664	3 d -15 y	Monthly	C	Hospitalized with ARI	T_{mean}	RSV	Upper-middle	PH
Tamara (2009)[137]	Amsterdam, Netherlands	1998-2005	417	10672	< 6 m	Weekly	C	Reported cases	T_{min}	RSV	High	PL

Note: *d: day(s), wk: week(s), m: month(s), y: year(s); $T_{mean:}$ mean temperature; $T_{max:}$ maximum temperature; $T_{min:}$ minimum temperature; TNS: Turkish Neonatal Society; C: Temperate; A: Tropical; B: Arid; D: Continental; ILI: Influenza-like illness; LRTI: lower respiratory tract infection; ARI: acute respiratory infection; CAP: Community acquired pneumonia; VRI: viral respiratory infection; RSV: Respiratory syncytial virus; IAV: influenza A virus; IBV: influenza B virus; HAdV: human adenovirus; HPIV: human parainfluenza virus; HMPV: human metapneumovirus; HBoV: human bocavirus; HRV: human rhinovirus; EV: enterovirus; HCoV: human coronavirus; EV/HRV: enterovirus/human rhinovirus; MERS-CoV: Middle East Respiratory Syndrome Coronavirus; SARS-CoV: Severe Acute Respiratory Syndrome Coronavirus; *M. pneumoniae: Mycoplasma pneumoniae; S. pneumoniae: Streptococcus pneumoniae; C. pneumoniae: Chlamydophila pneumoniae; L. pneumophila: Legionella pneumophila; H. influenzae : Haemophilus influenzae; M. catarrhalis: Moraxella catarrhalis; P. aeruginosa: Pseudomonas aeruginosa; S. pyogenes: Streptococcus pyogenes* pharyngitis

Classified as ECDC NORMAL

References

1. Marcelo RZ, Lustik MB, Jones MU. Seasonality and Climatic Factors Associated With Human Rhinovirus/Enterovirus Nasopharyngeal Sample Positivity on Oahu, Hawai'i, 2016-2019. *Military medicine* 2023.
2. Seah A, Loo LH, Jamali N, Maiwald M, Aik J. The influence of air quality and meteorological variations on influenza A and B virus infections in a paediatric population in Singapore. *Environmental research* 2023; **216**(Pt 1): 114453.
3. Chan CM, Wahab AA, Ali A. Determining the Relationship of Meteorological Factors and Severe Pediatric Respiratory Syncytial Virus (RSV) Infection in Central Peninsular Malaysia. *International journal of environmental research and public health* 2023; **20**(3).
4. Wagatsuma K, Koolhof IS, Saito R. Nonlinear and Multidelayed Effects of Meteorological Drivers on Human Respiratory Syncytial Virus Infection in Japan. *Viruses* 2023; **15**(9).
5. Lee PI, Liu CC, Hu YL, Chen JM. Seasonality and risk factor analysis of respiratory syncytial virus infection in children in Taiwan-A retrospective study from 1995 to 2005. *Journal of medical virology* 2023; **95**(10): e29116.
6. Lee MH, Mailepessov D, Yahya K, Loo LH, Maiwald M, Aik J. Air quality, meteorological variability and pediatric respiratory syncytial virus infections in Singapore. *Scientific reports* 2023; **13**(1): 1001.
7. Joung YH, Jang TS, Kim JK. Association among sentinel surveillance, meteorological factors, and infectious disease in Gwangju, Korea. *Environmental science and pollution research international* 2022; **29**(12): 17561-9.
8. Zhang Ling WJ, Li Hu. Epidemiological analysis of common respiratory viruses in children with acute respiratory infections in Jiangyin area from 2015 to 2020. *Journal of Southeast University (Medical Edition)* 2022; **41**(2): 203-7.
9. Yuan Xinqi JF, Lu Ruige, Zhou Jiaji Correlation analysis between acute mycoplasma pneumonia infection inchildren and climate change. *Chinese Journal of Woman and Child Health Research* 2022; **33**(1).
10. Wang Na GX, Xu Yun, Zhou Shiran, Zhang Fan, Qu Chunyan. Association between Mycoplasma pneumoniae infection and meteorological factors in children in Nantong city. 2022; **23**(3): 215-9.
11. Ma P, Tang X, Zhang L, et al. Influenza A and B outbreaks differed in their associations with climate conditions in Shenzhen, China. *International journal of biometeorology* 2022; **66**(1): 163-73.
12. Huang Feng GW, Jiang Wujun, Sun Huiming, Chen Zhengrong, Yan Yongdong, Hao Chuangli, Zhu Canhong. Association of Haemophilus influenzae infection with environmental and climatic

Classified as ECDC NORMAL

factors in Suzhou, China. *Chinese Journal of Contemporary Pediatrics* 2022; **24**(12): 1351-5.
13. Xu M, Yue W, Song X, et al. Epidemiological Characteristics of Parainfluenza Virus Type 3 and the Effects of Meteorological Factors in Hospitalized Children With Lower Respiratory Tract Infection. *Frontiers in pediatrics* 2022; **10**: 872199.
14. Li Y, Wang X, Broberg EK, Campbell H, Nair H. Seasonality of respiratory syncytial virus and its association with meteorological factors in 13 European countries, week 40 2010 to week 39 2019. *Euro surveillance : bulletin Europeen sur les maladies transmissibles = European communicable disease bulletin* 2022; **27**(16).
15. Shim SR, Kim HJ, Hong M, et al. Effects of meteorological factors and air pollutants on the incidence of COVID-19 in South Korea. *Environmental research* 2022; **212**(Pt C): 113392.
16. Haga L, Ruuhela R, Auranen K, Lakkala K, Heikkilä A, Gregow H. Impact of Selected Meteorological Factors on COVID-19 Incidence in Southern Finland during 2020-2021. *International journal of environmental research and public health* 2022; **19**(20).
17. Wagatsuma K, Koolhof IS, Saito R. The Relative Roles of Ambient Temperature and Mobility Patterns in Shaping the Transmission Heterogeneity of SARS-CoV-2 in Japan. *Viruses* 2022; **14**(10).
18. Lei C, Lou CT, Io K, et al. Viral etiology among children hospitalized for acute respiratory tract infections and its association with meteorological factors and air pollutants: a time-series study (2014-2017) in Macao. *BMC infectious diseases* 2022; **22**(1): 588.
19. Zhi W, Xu Q, Chen Z, et al. Respiratory syncytial virus infection in children and its correlation with climatic and environmental factors. *The Journal of international medical research* 2021; **49**(9): 3000605211044593.
20. Oh EJ, Kim JM, Joung YH, Kim JK. Effects of climatic factors on human parainfluenza 1, 2, and 3 infections in Cheonan, Republic of Korea. *Environmental science and pollution research international* 2021; **28**(8): 10018-26.
21. Solanes A, Laredo C, Guasp M, et al. No Effects of Meteorological Factors on the SARS-CoV-2 Infection Fatality Rate. *Biomedical and environmental sciences : BES* 2021; **34**(11): 871-80.
22. Liu Zhenchao LG, Zhou Zhen, Yu Zhuqin, Wang Guanxi. Climate Factors associated with COVID-19 Activity in Two Different Climate Environments. *Journal of Qingdao University (Medical Edition)* 2021; **57**(6).
23. Naren S, Zhang Jiayi, Zheng Lisheng, Shen Yongming. Prevalence of mycoplasma pneumoniae respiratory infection in children in Tianjin from 2015 to 2019 and its relationship with environmental factors. *Maternal and Child Health Care of China* 2021; **31**(19): 4496-8.

Classified as ECDC NORMAL

24. Wu Shouye LD, Wang Yaya. Clinical epidemiological characteristics and climatic factors of Different Subtypes of respiratory syncytial virus infection in children. *Hebei Medicine* 2021; **27**(10).
25. Chen Chun LQ, Wang Hui, Di Biao, Cai Wenfeng, Li Tiegang, Yang Zhicong. Short-term effects of meteorological factors on the incidence of COVID-19 in Guangdong Province. *J Med Pest Control* 2021; **37**(7): 639-46.
26. Soh S, Loo LH, Jamali N, Maiwald M, Aik J. Climate variability and seasonal patterns of paediatric parainfluenza infections in the tropics: An ecological study in Singapore. *International journal of hygiene and environmental health* 2022; **239**: 113864.
27. Oh EJ, Park, J., & Kim, J. K. Human Mastadenovirus Infections and Meteorological Factors in Cheonan, Korea. . *Microbiology and Biotechnology Letters* 2021; **49**(249-254).
28. Chen C, Zhang X, Jiang D, et al. Associations between Temperature and Influenza Activity: A National Time Series Study in China. *International journal of environmental research and public health* 2021; **18**(20).
29. Chan PK, Sung RY, Fung KS, et al. Epidemiology of respiratory syncytial virus infection among paediatric patients in Hong Kong: seasonality and disease impact. *Epidemiology and infection* 1999; **123**(2): 257-62.
30. Dong Kyu Lim BKJ, Jae Kyung Kim. Climate Factors and Their Effects on the Prevalence of Rhinovirus Infection in Cheonan, Korea. *Microbiology and Biotechnology Letters* 2021; **49**(3): 425–31.
31. Umuhoza T, Oyugi J, Mancuso JD, Ahmed A, Bulimo WD. Morbidity burden, seasonality and factors associated with the human respiratory syncytial virus, human parainfluenza virus, and human adenovirus infections in Kenya. *IJID regions* 2021; **1**: 72-8.
32. Zhang L, Lai M, Ai T, et al. Analysis of mycoplasma pneumoniae infection among children with respiratory tract infections in hospital in Chengdu from 2014 to 2020. *Translational pediatrics* 2021; **10**(4): 990-7.
33. Yassin MF, Aldashti HA. Stochastic analysis of the relationship between atmospheric variables and coronavirus disease (COVID-19) in a hot, arid climate. *Integrated environmental assessment and management* 2022; **18**(2): 500-16.
34. Liu Xiangyu LX, Zhai Hui, Chen Shiyi, Deng Yu, Xie Xiaohong, Zang Na, Xie Jun, Luo Zhengxiu, Luo Jian, Li Qubei, Fuzhou, Ren Luo, Liu Enmei. The correlation study on human respiratory syncytial virus daily incidence and meteorological parameters in the main urban area of Chongqing from 2009 to 2019. *Chinese Medical Journal* 2021; **101**(36): 2878-84.
35. Linssen RS, den Hollander B, Bont L, van Woensel JBM, Bem RA, On Behalf Of The Pice Study G. The Association between Weather Conditions and Admissions to the Paediatric Intensive Care Unit for Respiratory Syncytial Virus Bronchiolitis. *Pathogens (Basel, Switzerland)* 2021; **10**(5).

Classified as ECDC NORMAL

36. Kim JM, Jeon JS, Kim JK. Climate and Human coronaviruses 229E and Human coronaviruses OC43 Infections: Respiratory Viral Infections Prevalence in Hospitalized Children in Cheonan, Korea. *Journal of microbiology and biotechnology* 2020; **30**(10): 1495-9.
37. Thongpan I, Vongpunsawad S, Poovorawan Y. Respiratory syncytial virus infection trend is associated with meteorological factors. *Scientific reports* 2020; **10**(1): 10931.
38. Altamimi A, Ahmed AE. Climate factors and incidence of Middle East respiratory syndrome coronavirus. *Journal of infection and public health* 2020; **13**(5): 704-8.
39. Ali ST, Tam CC, Cowling BJ, Yeo KT, Yung CF. Meteorological drivers of respiratory syncytial virus infections in Singapore. *Scientific reports* 2020; **10**(1): 20469.
40. Sehra ST, Salciccioli JD, Wiebe DJ, Fundin S, Baker JF. Maximum Daily Temperature, Precipitation, Ultraviolet Light, and Rates of Transmission of Severe Acute Respiratory Syndrome Coronavirus 2 in the United States. *Clinical infectious diseases : an official publication of the Infectious Diseases Society of America* 2020; **71**(9): 2482-7.
41. Zhang Baoqin WY. Epidemiology and Clinical Characteristics of Human Parainfluenza 3 Pneumonia in Hospitalized Children in Suzhou. *Journal of Pediatric Pharmacy* 2020; **16**(12): 32-5.
42. Lolli S, Chen YC, Wang SH, Vivone G. Impact of meteorological conditions and air pollution on COVID-19 pandemic transmission in Italy. *Scientific reports* 2020; **10**(1): 16213.
43. Kong Linghao TG, Lu Shu, Kong Lingjing, Peng Jian, Chen Bingjun. Correlation between Meteorological Data and Incidence of Coronavirus Disease 2019 in Wuhan. *World Chinese medicine* 2020; **15**(4): 524-8.
44. Lopes GP, Amorim Í PS, Melo BO, et al. Identification and seasonality of rhinovirus and respiratory syncytial virus in asthmatic children in tropical climate. *Bioscience reports* 2020; **40**(9).
45. Li Y, Ye X, Zhou J, Zhai F, Chen J. The association between the seasonality of pediatric pandemic influenza virus outbreak and ambient meteorological factors in Shanghai. *Environmental health : a global access science source* 2020; **19**(1): 71.
46. Price RHM, Graham C, Ramalingam S. Association between viral seasonality and meteorological factors. *Scientific reports* 2019; **9**(1): 929.
47. Huang Juan WG, Rong Jiayan, Kong Delong, Liu Xiangteng, Huang Jianmei, Lin Hanlian, Lin Jiabao, Wang Bingjie. The etiology of community acquired pneumonia among hospitalized children in Zhongshan and its relation with climate parameters. *Chin Prev Med* 2019; **20**(3): 203-7.
48. Pan M, Yang HP, Jian J, et al. Association of meteorological factors with seasonal activity of influenza A subtypes and B lineages in subtropical western China. *Epidemiology and infection* 2019; **147**: e72.

Classified as ECDC NORMAL

49. He Xiangping WY. Epidemiological characteristics of hBoV with respiratory tract infection in children and the relationship between the infection and meteorological conditions. *Journal of Clinical Pulmonary Medicine* 2019; **24**(11): 1941-3.

50. Wu Qian WQ, Zhang Hailong, Yang Wenwen, Mo Zhuangbin, Bian Xiaofeng, Hong Chaochang, Li Kunjiao. Epidemiological characteristics of childhood Mycoplasma pneumoniae infection and its correlation with meteorological factors in Wuzhishan area, China. *Chinese Journal of Zoonoses* 2019; **35**(7): 667-71.

51. Su W, Liu T, Geng X, Yang G. Seasonal pattern of influenza and the association with meteorological factors based on wavelet analysis in Jinan City, Eastern China, 2013-2016. *PeerJ* 2020; **8**: e8626.

52. Lim YK, Kweon OJ, Kim HR, Kim TH, Lee MK. Clinical Features, Epidemiology, and Climatic Impact of Genotype-specific Human Metapneumovirus Infections: Long-term Surveillance of Hospitalized Patients in South Korea. *Clinical infectious diseases : an official publication of the Infectious Diseases Society of America* 2020; **70**(12): 2683-94.

53. Cheliotis KS, Jewell CP, Solórzano C, et al. Influence of sex, season and environmental air quality on experimental human pneumococcal carriage acquisition: a retrospective cohort analysis. *ERJ open research* 2022; **8**(2).

54. Darniot M, Pitoiset C, Millière L, et al. Different meteorological parameters influence metapneumovirus and respiratory syncytial virus activity. *Journal of clinical virology : the official publication of the Pan American Society for Clinical Virology* 2018; **104**: 77-82.

55. Liu WK, Liu Q, Chen DH, et al. Epidemiology of HBoV1 infection and relationship with meteorological conditions in hospitalized pediatric patients with acute respiratory illness: a 7-year study in a subtropical region. *BMC infectious diseases* 2018; **18**(1): 329.

56. Morley C, Grimwood K, Maloney S, Ware RS. Meteorological factors and respiratory syncytial virus seasonality in subtropical Australia. *Epidemiology and infection* 2018; **146**(6): 757-62.

57. Munshili Njifon HL, Monamele CG, Kengne Nde C, et al. Influence of meteorological parameters in the seasonality of influenza viruses circulating in Northern Cameroon. *Influenza and other respiratory viruses* 2019; **13**(2): 158-65.

58. Brini I, Bhiri S, Ijaz M, et al. Temporal and climate characteristics of respiratory syncytial virus bronchiolitis in neonates and children in Sousse, Tunisia, during a 13-year surveillance. *Environmental science and pollution research international* 2020; **27**(19): 23379-89.

59. Ji-Hyun Jung S-YC, Je-Yeon Kim, Tae-Hee Han, Sang-Hun Park, Ju-Young Chung, Hyo-Bin Kim. Correlation of respiratory syncytial virus infection with climate parameters and air pollution levels in Korean children during 2005–2012. *Allergy Asthma Respir Dis* 2018; **6**(4): 206-10.

60. Peci A, Winter AL, Li Y, et al. Effects of Absolute Humidity, Relative Humidity, Temperature, and Wind Speed on Influenza Activity in Toronto, Ontario, Canada. *Applied and environmental microbiology* 2019; **85**(6).

Classified as ECDC NORMAL

61. Althouse BM, Flasche S, Minh LN, et al. Seasonality of respiratory viruses causing hospitalizations for acute respiratory infections in children in Nha Trang, Vietnam. *International journal of infectious diseases : IJID : official publication of the International Society for Infectious Diseases* 2018; **75**: 18-25.
62. Zhou Dan WY. Epidemiological Characteristics of Human Rhinovirus with Acute Respiratory Tract Infection in Children and Its Correlation with Climate in Suzhou during 2013 – 2015. *Journal of Pediatric Pharmacy* 2018; **24**(8).
63. Kim JM JJ, Kim JK. Weather and its effects on RSV A and B infections in infants and children in Korea. *AMJ* 2017; **10**(12): 997–1002.
64. Paiva TM, Ishida MA, Benega MA, et al. Shift in the timing of respiratory syncytial virus circulation in a subtropical megalopolis: implications for immunoprophylaxis. *Journal of medical virology* 2012; **84**(11): 1825-30.
65. Yuqing Wang WJ, Chuangli Hao, Yongdong Yan , Zhengrong Chen , Xinxing Zhang , Wenjing Gu, Xuejun Shao, Jun Xu. Epidemiological characteristics of mycoplasma pneumoniae respiratory tract infection in hospitalized children from 2006 to 2013. *Int J Clin Exp Pathol* 2017; **10**(5): 5955-63.
66. Monamele GC, Vernet MA, Nsaibirni RFJ, et al. Associations between meteorological parameters and influenza activity in a subtropical country: Case of five sentinel sites in Yaoundé-Cameroon. *PloS one* 2017; **12**(10): e0186914.
67. Yan Y, Huang L, Wang M, et al. Clinical and epidemiological profiles including meteorological factors of low respiratory tract infection due to human rhinovirus in hospitalized children. *Italian journal of pediatrics* 2017; **43**(1): 23.
68. Tian DD, Jiang R, Chen XJ, Ye Q. Meteorological factors on the incidence of MP and RSV pneumonia in children. *PloS one* 2017; **12**(3): e0173409.
69. Zhou Wubin HZ. The study of the epidemic characteristics of human rhinovirus acute low respiratory tract infection in children and the risk factors in Huangshi. *Chinese Journal of Medical Frontiers (Electronic Edition)* 2017; **9**(8): 70-3.
70. Nenna R, Evangelisti M, Frassanito A, et al. Respiratory syncytial virus bronchiolitis, weather conditions and air pollution in an Italian urban area: An observational study. *Environmental research* 2017; **158**: 188-93.
71. Dong W, Chen Q, Hu Y, et al. Epidemiological and clinical characteristics of respiratory viral infections in children in Shanghai, China. *Archives of virology* 2016; **161**(7): 1907-13.
72. Ye Q, Fu JF, Mao JH, Shang SQ. Haze is a risk factor contributing to the rapid spread of respiratory syncytial virus in children. *Environmental science and pollution research international* 2016; **23**(20): 20178-85.
73. Psoter KJ, AJ DER, Wakefield J, Mayer JD, Bryan M, Rosenfeld M. Association of meteorological and geographical factors and risk of initial Pseudomonas aeruginosa acquisition in young children with cystic fibrosis. *Epidemiology and infection* 2016; **144**(5): 1075-83.

Classified as ECDC NORMAL

74. Sundell N, Andersson LM, Brittain-Long R, Lindh M, Westin J. A four year seasonal survey of the relationship between outdoor climate and epidemiology of viral respiratory tract infections in a temperate climate. *Journal of clinical virology : the official publication of the Pan American Society for Clinical Virology* 2016; **84**: 59-63.
75. Shi Yunju LL, Li Xiaojing, Guo Hongxia, Zhang Qiujun. Respiratory tract infection pneumonia mycoplasma survey and its correlation with seasonal factor. *Chin J Nosocomiol* 2016; **26**(14): 3216-8.
76. Chen Ying ZG, Liu Weidong. Relationship between acute viral respiratory tract infection and climate factors in hospitalized children in Yancheng area. *Clinical research* 2016; (16).
77. Sobral MFF, Duarte GB, da Penha Sobral AIG, Marinho MLM, de Souza Melo A. Association between climate variables and global transmission oF SARS-CoV-2. *Science of The Total Environment* 2020; **729**: 138997.
78. Leotte J, Trombetta H, Faggion HZ, et al. Impact and seasonality of human rhinovirus infection in hospitalized patients for two consecutive years. *Jornal de pediatria* 2017; **93**(3): 294-300.
79. Ikäheimo TM, Jaakkola K, Jokelainen J, et al. A Decrease in Temperature and Humidity Precedes Human Rhinovirus Infections in a Cold Climate. *Viruses* 2016; **8**(9).
80. Gamba-Sanchez N, Rodriguez-Martinez CE, Sossa-Briceño MP. Epidemic activity of respiratory syncytial virus is related to temperature and rainfall in equatorial tropical countries. *Epidemiology and infection* 2016; **144**(10): 2057-63.
81. Rodriguez-Martinez CE, Sossa-Briceño MP, Acuña-Cordero R. Relationship between meteorological conditions and respiratory syncytial virus in a tropical country. *Epidemiology and infection* 2015; **143**(12): 2679-86.
82. Geng Jia GW, Zhang Xuelan Prevalence of respiratory syncytial virus infection in hospitalized children at a children's hospital and effects of climate change on the prevalence in Suzhou, China. *Chin J Contemp Pediatr* 2015; **17**(5).
83. Hervás D, Hervás-Masip J, Ferrés L, Ramírez A, Pérez JL, Hervás JA. Effects of meteorologic factors and schooling on the seasonality of group A streptococcal pharyngitis. *International journal of biometeorology* 2016; **60**(5): 763-9.
84. Lu L, Yan Y, Yang B, et al. Epidemiological and clinical profiles of respiratory syncytial virus infection in hospitalized neonates in Suzhou, China. *BMC infectious diseases* 2015; **15**: 431.
85. Cui B, Zhang D, Pan H, et al. Viral aetiology of acute respiratory infections among children and associated meteorological factors in southern China. *BMC infectious diseases* 2015; **15**: 124.

Classified as ECDC NORMAL

86. Lin Jiansheng RZ, Shi Zongming, Huang Anqing, Lin Chunli, and Fu Qingliu. Epidemiological study of respiratory tract infection by different pathogens in Quanzhou during the year of 2013 and their correlation with climate parameters *Clinical Laboratory Journal(Electronic Edition)* 2015; **4**(3): 897-903.
87. Yan Huajie HD, Sheng Jun, Dong Wei, Liu Jia, Shao Jie. Association between parainfluenza virus infection and climatic factors in children. *Chin J Contemp Pediatr* 2015; **17**(12).
88. Huang Li CZ, Wang Meijuan, Ji Wei, Zhang Xinxing, Gu Wenjing, Shao Xuejun, Yan Yongdong. Epidemic characteristics of human rhinovirus acute low respiratory tract infection in children and the relationship between the infection and meteorological conditions in Suzhou. *Acta Univ Med Nanjing* 2015; **35**(10): 1425-8.
89. Ni Huiping JW, Chen Zhengrong. Role of Climate factors on Mycoplasma Pneumoniae Pneumonia in Children. *Chinese Journal of Medicinal Guide* 2014; **16**(11): 1385-6.
90. Sun Huiming ZW, Ji Wei, Yan Yongdong, Chen Zhengrong, Tao Yunzhen. The relationship between meteorological parameters and the prevalence of Moraxella catarrhalis infection in children hospitalized with acute respiratory infection in Suzhou. *J Clin Pediatr* 2014; **32**(6): 524-7.
91. Onozuka D. The influence of diurnal temperature range on the incidence of respiratory syncytial virus in Japan. *Epidemiology and infection* 2015; **143**(4): 813-20.
92. Chen Z, Zhu Y, Wang Y, et al. Association of meteorological factors with childhood viral acute respiratory infections in subtropical China: an analysis over 11 years. *Archives of virology* 2014; **159**(4): 631-9.
93. Chen ZR, Mize M, Wang YQ, et al. Clinical and epidemiological profiles of lower respiratory tract infection in hospitalized children due to human bocavirus in a subtropical area of China. *Journal of medical virology* 2014; **86**(12): 2154-62.
94. Chen Z, Ji W, Wang Y, et al. Epidemiology and associations with climatic conditions of Mycoplasma pneumoniae and Chlamydophila pneumoniae infections among Chinese children hospitalized with acute respiratory infections. *Italian journal of pediatrics* 2013; **39**: 34.
95. Tang Shenghui WY. The Relationship between Meteorological Conditions and Respiratory Syncytial Virus Infection in Hospitalized Children. *Journal of pediatric Pharmacy* 2013; **19**(5).
96. Wan Fengguo ZX, Shao Xuejun, Ji Zhenghua. Epidemiological feature of respiratory syncytial virus in hospitalized children in Suzhou *J Clin Pediatr* 2013; **31**(8): 723-5.
97. Xu YC, Zhu LJ, Xu D, et al. Epidemiological characteristics and meteorological factors of childhood Mycoplasma pneumoniae pneumonia in Hangzhou. *World journal of pediatrics : WJP* 2011; **7**(3): 240-4.

Classified as ECDC NORMAL

98. Hongling Z. Study on the correlation between epidemic activity of mycoplasma and climatic factors in infants in Luoyang area. *Chinese Medical Innovation* 2013; **10**(25).
99. Yin Song CZ. Relationship between epidemiological features of acute respiratory tract infection due to mycoplasma pneumoniae in children and meterological factors. *Journal of Community Medicine* 2013; **11**(23): 7-9.
100. Chen ZR, Yan YD, Wang YQ, et al. Epidemiology of community-acquired Mycoplasma Pneumoniae respiratory tract infections among hospitalized Chinese children, including relationships with meteorological factors. *Hippokratia* 2013; **17**(1): 20-6.
101. Li H, Wei Q, Tan A, Wang L. Epidemiological analysis of respiratory viral etiology for influenza-like illness during 2010 in Zhuhai, China. *Virology journal* 2013; **10**: 143.
102. Wang Y, Chen Z, Yan YD, et al. Seasonal distribution and epidemiological characteristics of human metapneumovirus infections in pediatric inpatients in Southeast China. *Archives of virology* 2013; **158**(2): 417-24.
103. Chen ZR, Ji W, Wang YQ, et al. Etiology of acute bronchiolitis and the relationship with meteorological conditions in hospitalized infants in China. *Journal of the Formosan Medical Association = Taiwan yi zhi* 2014; **113**(7): 463-9.
104. Khor CS, Sam IC, Hooi PS, Quek KF, Chan YF. Epidemiology and seasonality of respiratory viral infections in hospitalized children in Kuala Lumpur, Malaysia: a retrospective study of 27 years. *BMC pediatrics* 2012; **12**: 32.
105. Chen Zhengrong JW, Wang Yuqing, Guo Hongbo, Yan Yongdong, Zhang Xuelan. The relationship of meteorological conditions to the epidemic activity of adenovirus in hospitalized children with acute respiratory infection during 2006-2010. *J Clin Pediatr* 2012; **30**(6): 539-41.
106. Society TN. The seasonal variations of respiratory syncytial virus infections in Turkey: a 2-year epidemiological study. *The Turkish journal of pediatrics* 2012; **54**(3): 216-22.
107. Hervás D, Reina J, Hervás JA. Meteorologic conditions and respiratory syncytial virus activity. *The Pediatric infectious disease journal* 2012; **31**(10): e176-81.
108. Herrera-Lara S, Fernández-Fabrellas E, Cervera-Juan Á, Blanquer-Olivas R. Do seasonal changes and climate influence the etiology of community acquired pneumonia? *Archivos de bronconeumologia* 2013; **49**(4): 140-5.
109. Ji Wei CZ, Guo Hongbo, Wang Guanjuan, Yan Yongdong, Zhang Xuelan, Ding Yunfang. Characteristics and the prevalence of respiratory viruses and the correlation with climatic factors of hospitalized children in Suzhou childrents hospital. *Chinese journal of preventive medicine* 2011; **45**(3): 205-10.
110. Sun Shiwei SX, Chen Zhengrong, Guo Hongbo, Ji Wei. Relationship of meteorological conditions and epidemic activity of parainfluenza virus-3. *Jiangsu Med J* 2011; **37**(8).

Classified as ECDC NORMAL

111. Wang Yuqing JW, Chen Zhengrong, Yan Yongdong, Guo Hongbo, Chu Chu, Liu Jingding, Yun Fang, Shao Xuejun, Xu Jun. Characteristics of humanmetapneumovirus respiratorytract infectionin children and the relationship between the infection and meteorological conditions. *Chin J Pediatr* 2011; **49**(3): 2114-217.
112. Loh TP, Lai FY, Tan ES, et al. Correlations between clinical illness, respiratory virus infections and climate factors in a tropical paediatric population. *Epidemiology and infection* 2011; **139**(12): 1884-94.
113. Nascimento-Carvalho CM, Cardoso MR, Barral A, et al. Seasonal patterns of viral and bacterial infections among children hospitalized with community-acquired pneumonia in a tropical region. *Scandinavian journal of infectious diseases* 2010; **42**(11-12): 839-44.
114. Cao Zhidong ZD, Wang Feiyue, Wang Quanyi, Wang Xiaoli, Wang Jiaojiao, Zheng Xiaolong. Weather Conditions and Spatio-Temporal Spreading Risk of the Beijing 2009 Influenza A(H1N1) Epidemic. *Technology Review* 2010; **28**(8): 26-32.
115. du Prel JB, Puppe W, Gröndahl B, et al. Are meteorological parameters associated with acute respiratory tract infections? *Clinical infectious diseases : an official publication of the Infectious Diseases Society of America* 2009; **49**(6): 861-8.
116. Noyola DE, Mandeville PB. Effect of climatological factors on respiratory syncytial virus epidemics. *Epidemiology and infection* 2008; **136**(10): 1328-32.
117. Mathisen M, Strand TA, Sharma BN, et al. RNA viruses in community-acquired childhood pneumonia in semi-urban Nepal; a cross-sectional study. *BMC medicine* 2009; **7**: 35.
118. Onozuka D, Hashizume M, Hagihara A. Impact of weather factors on Mycoplasma pneumoniae pneumonia. *Thorax* 2009; **64**(6): 507-11.
119. Tang JW, Lai FY, Wong F, Hon KL. Incidence of common respiratory viral infections related to climate factors in hospitalized children in Hong Kong. *Epidemiology and infection* 2010; **138**(2): 226-35.
120. Wang Junhua FC, Liu Sheng, He Jieshu, Liu Bolun, Cheng Minxian. Epidemiological characteristics and meteorological factors in childhood pidemiological characteristics and meteorological factors in childhood Mycoplasma pneumoniae ycoplasma pneumoniaeinfection in Guilin. *J Clin Pediatr* 2013; **31**(13): 1038-41.
121. Asma Al-Thania ME, Mohammed Janahic, Ajayeb Al-Marrib, Huseyin Caksend, Abdulbari Bener. Seasonality and epidemiology of respiratory syncytial virus in Qatar. *Journal of Pediatric Infectious Diseases* 2008; **3**: 41–5.
122. Yuan J, Yun H, Lan W, et al. A climatologic investigation of the SARS-CoV outbreak in Beijing, China. *American journal of infection control* 2006; **34**(4): 234-6.
123. Bi P, Wang J, Hiller JE. Weather: driving force behind the transmission of severe acute respiratory syndrome in China? *Internal medicine journal* 2007; **37**(8): 550-4.

Classified as ECDC NORMAL

124. Wang Tianlin CZ, Tang Hongfeng, Tang Lanfang, Zou Chaochun, Wu Lihong. Study on the relations between epidemics of respiratory syncytialvirus infection in children and climate factorsin Hangzhou. *Chinese Journal of epidemiology* 2005; **26**(8).
125. Feng Yerong ZK, Ji Zhongping, Du Lin, Wang Anyu, Jin Shunying. Study on the relationship between atmospheric environmental factors and short-term changes of SARS epidemic in Guangzhou. *Journal of Tropical Meteorology* 2005; **21**(2): 191-8.
126. Viegas M, Barrero PR, Maffey AF, Mistchenko AS. Respiratory viruses seasonality in children under five years of age in Buenos Aires, Argentina: a five-year analysis. *The Journal of infection* 2004; **49**(3): 222-8.
127. Huang Ping YS, Chen Qing, Zheng Huanying, Hu Jing, Luo Qiuhong, Nie Jun. Study on the relationship between SARS prevalence and climate change in Guangdong Province. *Chinese Journal of Zoonoses* 2004; **24**(8).
128. Zhang H, Wen S, Zheng J, Chen X, Lv F, Liu L. Meteorological factors affecting respiratory syncytial virus infection: A time-series analysis. *Pediatric pulmonology* 2020; **55**(3): 713-8.
129. Liu WK, Chen DH, Tan WP, et al. Paramyxoviruses respiratory syncytial virus, parainfluenza virus, and human metapneumovirus infection in pediatric hospitalized patients and climate correlation in a subtropical region of southern China: a 7-year survey. *European journal of clinical microbiology & infectious diseases : official publication of the European Society of Clinical Microbiology* 2019; **38**(12): 2355-64.
130. Sirimi N, Miligkos M, Koutouzi F, Petridou E, Siahanidou T, Michos A. Respiratory syncytial virus activity and climate parameters during a 12-year period. *Journal of medical virology* 2016; **88**(6): 931-7.
131. Omer SB, Sutanto A, Sarwo H, et al. Climatic, temporal, and geographic characteristics of respiratory syncytial virus disease in a tropical island population. *Epidemiology and infection* 2008; **136**(10): 1319-27.
132. Chan PW, Chew FT, Tan TN, Chua KB, Hooi PS. Seasonal variation in respiratory syncytial virus chest infection in the tropics. *Pediatric pulmonology* 2002; **34**(1): 47-51.
133. Lapeña S, Robles MB, Castañón L, et al. Climatic factors and lower respiratory tract infection due to respiratory syncytial virus in hospitalised infants in northern Spain. *European journal of epidemiology* 2005; **20**(3): 271-6.
134. Vandini S, Corvaglia L, Alessandroni R, et al. Respiratory syncytial virus infection in infants and correlation with meteorological factors and air pollutants. *Italian journal of pediatrics* 2013; **39**(1): 1.
135. Pitzer VE, Viboud C, Alonso WJ, et al. Environmental drivers of the spatiotemporal dynamics of respiratory syncytial virus in the United States. *PLoS pathogens* 2015; **11**(1): e1004591.

Classified as ECDC NORMAL

136. Zhang XL, Shao XJ, Wang J, Guo WL. Temporal characteristics of respiratory syncytial virus infection in children and its correlation with climatic factors at a public pediatric hospital in Suzhou. *Journal of clinical virology : the official publication of the Pan American Society for Clinical Virology* 2013; **58**(4): 666-70.
137. Meerhoff TJ, Paget JW, Kimpen JL, Schellevis F. Variation of respiratory syncytial virus and the relation with meteorological factors in different winter seasons. *The Pediatric infectious disease journal* 2009; **28**(10): 860-6.

Classified as ECDC NORMAL

Table S10. Global distribution of common respiratory tract specific pathogen infections studies and sites (applied to the Figure 2)

Country	Number of studies	Number of sites
Leon, Spain	1	1
Valencia, Spain	1	1
Mallorca, Spain	2	1
52 Spanish provinces	1	1
Yaounde, Cameroon	1	1
Garoua, Cameroon	1	1
Riyadh, Saudi Arabia	1	1
Nha Trang, central Vietnam	1	1
Sousse, Northern Africa	1	1
Turkey four regions: Marmara, Mediterranean and Aegean region; Black Sea region; East Anatolian region; Middle Anatolian region	1	4
Kathmandu, Nepal	1	1
Nairobi, Kenya	1	1
Doha, Qatar	1	1
Safat, Kuwait	1	1
Lombok, Indonesia	1	1
Bangkok, Thailand	1	1
Athens, Greece	1	1
Toronto, Canada	1	1
Gold Coast, Australia	1	1
Buenos Aires, Argentina	1	1
Moscow	1	1
Dijon, France	1	1
Gothenburg, Sweden	1	1
Kajaani, Finland	1	1
Helsinki, Finland	1	1
Bukit Timah, Singapore	3	1
Singapore	2	1
Bologna, Italy	1	1
Milan; Florence; Trento, Italy	1	3
Rome, Italy	1	1
Amsterdam, the Netherlands	2	1
Nine states (Arizona, California, Colorado, Iowa, Massachusetts, Maryland, New Jersey, Washington, and Wisconsin) , United States	1	9
Rhode Island, USA	1	1
Oahu, USA	1	1
48 states of USA	1	1
Kuala Lumpur, Malaysia	3	1
San Luis Potosí, Mexico	1	1
Rheinland-Pfalz, Mainz	1	1
Curitiba, Brazil	1	1
Sao Paulo, Brazil	1	1
Maranhao, Brazil	1	1

Classified as ECDC NORMAL

Salvador, Brazil	1	1
Edinburgh, UK	1	1
Liverpool, UK	1	1
Bogota, Colombian	2	1
Cheonan, Korea	5	1
Changwon, Korea	1	1
Gwangju, Korea	1	1
Seoul, Korea	2	1
Fukuoka,Tokyo, Japan	3	1
Six Japanese prefectures (Hokkaido, Tokyo, Aichi, Osaka, Fukuoka, and Okinawa)	1	6
46 Okinawa prefecture, Japan	1	1
47 prefectures in Japan	1	1
13 European countries (Austria, Denmark, Estonia, France, Germany, Greece, Ireland, Netherlands, Poland, Portugal, Slovenia, Spain, United Kingdom)	1	13
Fifty U.S. states and Washington D.C.	1	1
Guangzhou, Hong Kong, Toronto, Singapore, Taiwan, Beijing	1	6
Guangzhou, China	2	1
Guangdong, China	1	1
Zhongshan, China	1	1
Zhuhai, China	1	1
Chaoshan, China	1	1
Shenzhen, China	1	1
Guangzhou, Jiangmen, Foshan, Shenzhen, Zhaoqing, and Zhongshan in Guangdong Province, China	1	6
Hong Kong, China	2	1
Beijing and Hong Kong, China	1	2
Beijing, China	2	1
Shanghai, China	3	1
Wenzhou, China	2	1
Hangzhou, China	3	1
Chengdu, China	2	1
Macao, China	1	1
Chongqing, China	1	1
Panzhihua, China	1	1
Wu zhishan, China	1	1
Henan, China	1	1
Luoyang, China	1	1
Zhengzhou, China	1	1
Jinan, China	1	1
Nantong, China	1	1
Taiwan, China	1	1
Quanzhou, China	1	1
Guilin, China	1	1
Huangshi, China	1	1
Tianjin, China	1	1

Classified as ECDC NORMAL

Haikou, China	1	1
Yancheng, China	1	1
Jiangyin, China	1	1
Wuhan, China	1	1
Suzhou, China	26	1

Classified as ECDC NORMAL

Table S11. Risk of bias (RoB) Assessment for each study (n=137)

Study	Exposure assessment	Outcome assessment	Confounding bias	Selection bias	Incomplete missing data	Selective outcome reporting	Conflict of interest	Other bias
Santiago (2004)	PL	PL	DL	DL	DL	DL	DL	PH
Zhang (2013)	PL	PL	PH	DL	PL	DL	PL	PL
Liu (2019)	PL	DL	DL	DL	DL	DL	DL	DL
Natalia (2016)	PL	DL	PL	PL	PH	DL	DL	DL
Silvia (2013)	PL	DL	PL	PL	DL	DL	PL	PL
Tamara (2009)	PH	DL	DL	DL	DL	DL	DL	PL
Omer (2008)	PL	DL	PL	PL	DL	DH	PL	PL
Virginia (2015)	PL	PL	PL	DL	PL	PL	DL	DL
Patrick (2015)	PL	DL	DL	DL	DL	DL	DL	DL
Hailin (2019)	PL	PL	DL	PL	PL	PL	PL	DL
Zheng (2014)	PL	PL	DL	DL	DL	PH	DL	DL
Ming (2022)	DL	PL	PL	DL	PL	DL	DL	DL
Eun (2021)	PL	DL	DL	DL	DL	DL	DL	DL
Ilada (2020)	DL	PH	DL	DL	DL	DL	DL	PL
Nicklas (2016)	DL	PL	DH	DL	DH	PH	DL	DL
Jean-Baptist (2009)	PL	PL	PH	PH	DL	DL	DL	DL
Noyola (2009)	DL	PL	PL	PL	PL	PL	PH	DL
Rodriguez-Martinez (2015)	PL	DL	DL	DL	DL	DL	DL	DL
Young (2019)	PH	PL	DL	DL	DL	DL	DL	PL
Zhi (2021)	PL	DH	PL	DH	DL	DL	DL	DL

Classified as ECDC NORMAL

Huang (2022)	PL	PL	PL	PL	PL	PL	PH	DL
Gamba-Sanchez (2016)	PL	DL	DL	DL	DL	DL	DL	DL
Cheng (2022)	DL	DL	DL	DL	DL	DL	DL	DL
Jang (2020)	PL	PL	DL	PL	PL	DL	DL	PL
Chee (2023)	PH	DL	DL	DL	DL	DL	DL	DL
Wen-Kuan (2018)	PL	PL	DL	PL	DL	PL	DL	DL
Li (2020)	PL	PH	DL	PL	DL	PL	PL	DL
Chen (2014)	PL	PL	DL	PL	DL	PL	DL	DL
Chen (2013)	PL	PL	DL	PL	DL	PL	DL	DL
Liu (2021)	PH	DL	DL	DL	DL	DL	DL	DL
Wang (2005)	DL	DL	PL	DL	PL	PL	DL	PL
Chen (2014)	PH	DL	DL	DL	DL	DL	DL	DL
Giselmo (2020)	PL	DL	DL	PL	PL	PL	DL	DL
Chen (2013)	PL	DL	DH	PH	PL	DL	DL	DL
Rory (2019)	PL	DL	DL	PL	DL	PL	DL	DL
Onozuka (2009	PL	DL	DL	PL	PL	DL	DL	DL
Keita (2022)	PL	PH	PH	DL	DL	PL	DL	DL
Mohamed (2021)	PL	PL	PH	DL	PL	PH	DL	DL
Yuan (2006)	PL	DL	PL	PL	PL	PL	DL	DL
Tian (2017)	PL	PH	DL	DL	DL	DL	DL	DL
Tiina (2016)	PL	PL	DL	DL	DH	DL	DL	DL
Cui (2015)	PL	DL	DH	DL	DH	DL	DL	DL
Yan (2017)	PL	DL	PL	DL	PL	DL	DL	DL

Lei (2021)	PL	PL	PL	PL	DL	DL	PL	DL
Jaqueline (2016)	PL	DL	PL	PL	PL	DL	DL	DL
Lu (2015)	PL	PL	PL	DL	PL	DL	DL	DL
Ji (2011)	PL	DL	PL	DL	PL	DL	DL	DL
Geng (2015)	PL	DL	PL	DL	PL	PL	DL	DL
Daniel (2015)	PL	DL	PL	PL	PL	DL	DL	DL
Cristiana (2010)	DL	PL	DL	DL	PL	PL	DL	DL
Susana (2012)	PH	PL	DL	DL	DL	DL	DL	DL
Maria (2009)	DL	DL	PL	PL	PL	PL	PL	DL
Katerina (2019)	PL	PL	DL	DL	DL	PL	PL	DL
Magali (2018)	PL	PL	DL	DL	DL	PL	DL	DL
Loh (2011)	PL	PL	DL	DL	DL	PL	DL	DL
Yong (2019)	PL	PL	DL	DL	PL	PL	DL	DL
Shi (2016)	PH	PL	DL	DL	DL	DL	PL	DL
Therese (2021)	DL	PL	PL	DL	DL	DL	PL	DL
Li (2013)	DL	PL	PL	PL	PL	DL	PL	DL
TNS (2012)	DL	PL	DL	DL	PL	DL	PL	DL
Wang (2013)	PL	PL	PL	DL	PL	PL	PL	DL
Daniel Hervás (2012)	PL	PL	DL	DL	PL	DL	DL	DL
Asma (2008)	PL	PL	DL	PL	PL	DL	PL	PL
Raffaella (2017)	PL	PL	DL	DL	PL	DL	PL	DL
Sheikh (2020)	PL	PL	DL	DL	PL	DL	PL	PL
Ines (2018)	DL	PL	DL	PL	PL	PL	PL	DL

Classified as ECDC NORMAL

Sun (2014)	DL	PL	DL	DL	PL	PL	PL	DL
You (2022)	PH	PL	DL	DL	PL	PL	PL	DL
Jung (2018)	DL	PL	DL	DL	PL	PL	PL	DL
Adriana (2018)	DL	PL	DL	DL	PL	PL	PL	DL
Chee-Sieng (2012)	DL	PL	DL	DL	PL	PL	PL	DL
Huang (2019)	PH	PL	DL	DL	DL	DL	DL	DL
Wei (2019)	PL	PL	DL	DL	PL	DL	PL	PL
Tang (2009)	PL	PL	DL	DL	PL	DL	PL	DL
Morley (2018)	PL	PL	DL	DL	PL	DL	PL	DL
Hermann (2018)	PL	PL	DL	DL	PL	DL	PL	DL
Rosalie (2021)	PL	PL	DL	DL	DL	DL	PL	DL
Wei (2016)	PL	PL	PL	DL	DL	DL	PL	DL
Keita (2023)	PL	PL	DL	DL	DL	DL	PL	DL
Qing (2016)	PL	PL	DL	DH	PL	DL	PL	DL
Jang (2017)	DL	PL	DL	DL	PL	DL	PL	PL
You (2022)	PL	PL	PL	DH	PL	DL	PL	DL
Terezinha (2017)	PL	PL	DL	DL	PL	DL	PL	DL
Raymundo (2023)	PL	PL	DL	DL	PL	DL	PL	DL
Wang (2017)	PL	DL	DL	DL	PL	DL	PL	DL
Gwladys (2017)	PL	PL	DL	DL	DH	DL	PL	DL
Annabel (2023)	PL	PL	PL	DH	DH	DL	PL	DL
Aleix (2021)	PL	PL	DL	DL	PL	DL	PL	DL

Classified as ECDC NORMAL

Bi (2006)	PL	DL	DL	DL	PL	DL	PL	DL
Mariana (2004)	PL	PL	DL	DL	PL	DL	PL	DL
Pan (2022)	PH	PL	DL	PL	DL	DL	PL	DL
Asmaa (2020)	DL	PL	DL	DL	DL	DL	PL	DL
Pan (2019)	DL	PL	DL	DL	DL	DL	PL	DL
Can (2021)	DL	PL	DL	DL	DH	DL	PL	DL
Na (2022)	DL	PL	DL	DL	PL	DL	PL	DL
Stacy (2021)	PL	DL	DL	DL	PL	DL	PL	DL
Eun (2021)	PL	PL	DL	DL	PL	DL	PL	DL
Ping-Ing (2023)	PL	PL	DL	DL	PL	DL	PL	DL
Chan (2021)	PL	PL	DL	DL	PL	DL	PL	DL
Dong (2021)	DL	PL	DL	DL	DH	DL	PL	DL
Onozuka (2014)	DL	PH	DL	DL	DL	DL	PL	DL
Benjamin (2018)	PL	PL	DL	DL	PL	DL	PL	DL
Shiv (2020)	DL	PL	DL	PL	DL	DL	DL	DL
PSOTER (2016)	PH	PL	DL	PL	DL	DL	DL	DL
Meng (2023)	DL	PL	DL	PL	DL	DL	DL	DL
Lin (2015)	DL	PL	DL	DL	DL	PL	DL	DL
Wang (2013)	DL	PL	DL	PL	DL	DL	DL	DL
Chen (2012)	PH	PL	DL	PL	DL	DL	DL	DL
Sun (2011)	PH	PL	DL	PL	PL	DL	DL	DL
Onozuka (2008)	PH	PL	DL	PL	DL	DL	DL	DL

Classified as ECDC NORMAL

Zhou (2018)	PH	DL	OH	DL	DL	DL	DL	DL
Wu (2019)	PH	DL	DL	DL	DL	DL	DL	DL
Zhou (2017)	PL	DL	DL	DL	PL	DL	DL	DL
Na (2021)	PL	DL	DL	DL	DL	DL	DL	DL
Huang (2015)	PH	DL	DL	PH	DL	DL	DL	DL
Wang (2011)	PH	DL	DL	DL	PL	DL	DL	DL
Yuan (2022)	PH	PL	DL	DL	DL	DL	DL	DL
Ni (2014)	PL	PL	DL	DL	DL	DL	DL	DL
Yin (2013)	PL	PL	PL	PL	DL	DL	DL	DL
Zheng (2013)	PL	PL	DL	PL	DL	DL	DL	DL
Sobral (2020)	PL	PL	DL	PL	DL	DL	DL	DL
Wu (2021)	PL	PL	DL	PL	DL	DL	DL	DL
Tang (2013)	PH	DL	DL	PL	DL	DL	DL	DL
Wan (2013)	PH	DL	DL	DL	DL	DL	DL	DL
Chen (2016)	PH	DL	PL	PL	DL	DL	DL	DL
Zhang (2022)	PH	PH	DL	PH	DL	DL	DL	DL
He (2019)	PL	DL	DL	PL	DL	DL	DL	DL
Yan (2015)	PL	DL	DL	PL	DL	DL	DL	DL
Zhang (2020)	PH	DL	DL	PL	DL	DL	DL	DL
Cao (2010)	PL	PH	DL	PL	DL	DL	DL	DL
Chen (2021)	PL	DL	DL	PL	DL	DL	DL	DL
Feng (2005)	PH	DL	DL	PL	DL	DL	DL	DL

Classified as ECDC NORMAL

Liu (2021)	PL	DL	DL	PL	DL	DL	DL	DL
Kong (2020)	PL	PH	DL	PL	DL	DL	DL	DL
Huang (2004)	PH	DL	DL	PL	DL	DL	DL	DL
Sung (2022)	PL	DL	DL	PL	DL	DL	DL	DL
Lisa (2022)	PL	PL	DL	PL	DL	DL	DL	DL
Simone (2020)	PL	DL	DL	PL	DL	DL	DL	DL

Note: PL- Probably Low risk; PH- Probably High risk; DH- Definitely High risk; DL- Definitely Low risk

Classified as ECDC NORMAL

Table S12. Summary of included studies corresponding to each pathogen

Pathogen	Study
**Viral infection**	
**RSV**	
Respiratory syncytial virus -A	Zhi (2021) [19] Cheng (2022)[18] Jang (2017)[63] Wu (2021)[24]
Respiratory syncytial virus -B	Zhi (2021) [19] Cheng (2022)[18] Jang (2017)[63] Wu (2021)[24]
Respiratory syncytial virus - unsubtyped	Santiago (2004)[133] Zhang (2013)[136] Liu (2019)[129] Natalia (2016)[130] Silvia (2013)[134] Omer (2008)[131] Virginia (2015)[135] Patrick (2015)[132] Hailin (2019)[128] Zheng (2014)[92] Ilada (2020)[37] Nicklas (2016)[74] Jean-Baptist (2009)[115] Noyola (2009)[116] Rodriguez-Martinez (2015)[81] Gamba-Sanchez (2016)[80] Chee (2023)[3] Chen (2012)[103] Liu (2021)[34] Giselmo (2020)[44] Rory (2019)[46] Tian (2017)[68] Cui (2015)[85] Lu (2015)[84] Cristiana (2010)[113] Maria (2009)[117] Magali (2018)[54] Therese (2021)[31] Li (2013)[101] Daniel Hervás (2012)[107] Asma (2008)[121] Raffaella (2017)[70] Sheikh (2020)[39] Ines (2018)[58] You (2022)[7] Chee-Sieng (2012)[104] Rosalie (2021)[35] Wei (2016)[71] Keita (2023)[4] Qing (2016)[72] Terezinha (2017)[64] You (2022)[14] Mariana (2004)[126] Ping-Ing (2023)[5] Chan (2021)[29] Onozuka (2014)[91] Benjamin (2018)[61] Meng (2023)[6] TNS (2012)[106] Wang (2005)[124] Geng (2015)[82] Jung (2018)[59] Lin (2015)[86] Tang (2013)[95] Wan (2013)[96] Chen (2016)[76] Zhang (2022)[8] Ji (2011)[109]
Overall respiratory syncytial virus (RSV)	Zhi (2021) [19] Cheng (2022)[18] Jang (2017)[63] Wu (2021)[24] Santiago (2004)[133] Zhang (2013)[136] Liu (2019)[129] Natalia (2016)[130] Silvia (2013)[134] Omer (2008)[131] Virginia (2015)[135] Patrick (2015)[132] Hailin (2019)[128] Zheng (2014)[92] Ilada (2020)[37] Nicklas (2016)[74] Jean-Baptist (2009)[115] Noyola (2009)[116] Rodriguez-Martinez (2015)[81] Gamba-Sanchez (2016)[80] Chee (2023)[3] Chen (2012)[103] Liu (2021)[34] Giselmo (2020)[44] Rory (2019)[46] Tian (2017)[68] Cui (2015)[85] Lu (2015)[84] Cristiana (2010)[113] Maria (2009)[117] Magali (2018)[54] Therese (2021)[31] Li (2013)[101] Daniel Hervás (2012)[107] Asma (2008)[121] Raffaella (2017)[70] Sheikh (2020)[39] Ines (2018)[58] You (2022)[7] Chee-Sieng (2012)[104] Rosalie (2021)[35] Wei (2016)[71] Keita (2023)[4] Qing (2016)[72] Terezinha (2017)[64] You (2022)[14] Mariana (2004)[126] Ping-Ing (2023)[5] Chan (2021)[29] Onozuka (2014)[91] Benjamin (2018)[61] Meng (2023)[6] TNS (2012)[106] Wang (2005)[124] Geng (2015)[82] Jung (2018)[59] Lin (2015)[86] Tang (2013)[95] Wan (2013)[96] Chen (2016)[76] Zhang (2022)[8] Ji (2011)[109]
**IV**	
Influenza virus A	Zheng (2014)[92] Jean-Baptist (2009)[115] Cheng (2022)[18] Li (2020)[45] Chen (2012)[103] Rory (2019)[46] Cui (2015)[85] Maria (2009)[117] Adriana (2018)[60] Gwladys (2017)[66] Annabel (2023)[2] Mariana (2004)[126] Benjamin (2018)[61] Ji (2011)[109] Zhang (2022)[8] Lin (2015)[86] Chen (2016)[76]
Influenza virus A/H1N1pdm09	Li (2013)[101] Wei (2019)[51] Pan (2019)[48] Pan (2022)[11] Can (2021)[28] Cao (2010)[114]
Influenza virus A/H3N2	Li (2013)[101] Adriana (2018)[60] Wei (2019)[51] Pan (2019)[48] Can (2021)[28]
Influenza virus B	Zheng (2014)[92] Nicklas (2016)[74] Jean-Baptist (2009)[115] Cheng (2022)[18] Li (2020)[45] Rory (2019)[46] Maria (2009)[117] Li (2013)[101] Adriana (2018)[60] Gwladys (2017)[66] Mariana (2004)[126] Pan (2022)[11] Ji (2011)[109] Lin (2015)[86] Chen (2016)[76] Zhang (2022)[8] Can (2021)[28] Wei (2019)[51]
Influenza virus B/Victoria	Pan (2019)[48]
Influenza virus B/Yamagata	Pan (2019)[48]
Influenza virus -unsubtyped	You (2022)[7] Adriana (2018)[60] Gwladys (2017)[66] Wei (2016)[71]
Overall influenza virus (IV)	Zheng (2014)[92] Jean-Baptist (2009)[115] Li (2020)[45] Chen (2014)[93] Rory (2019)[46] Cui (2015)[85] Maria (2009)[117] Adriana (2018)[60] Gwladys (2017)[66] Annabel (2023)[2] Mariana (2004)[126] Benjamin (2018)[61] Cao (2010)[114] Ji (2011)[109] Li (2013)[101] Wei (2019)[51] Pan (2022)[11] Can (2021)[28] Nicklas (2016)[74] Cheng (2022)[18] Lin (2015)[86] Chen (2016)[76] Zhang (2022)[8] Pan (2019)[48] You (2022)[7] Wei (2016)[71]
**HPIV**	
Human parainfluenza virus -1	Eun (2021)[20] Jean-Baptist (2009)[115] Ji (2011)[109] Maria (2009)[117] Chen (2016)[76] Zheng (2014)[92]
Human parainfluenza virus -2	Eun (2021)[20] Ji (2011)[109] Maria (2009)[117] Zheng (2014)[92] Chen (2016)[76]
Human parainfluenza virus -3	Chen (2016)[76] Cui (2015)[85] Eun (2021)[20] Jean-Baptist (2009)[115]

Classified as ECDC NORMAL

	Ji (2011)[109] Maria (2009)[117] Ming (2022)[13] Zheng (2014)[92] Sun (2011)[110] Chen (2016)[76] Zhang (2022)[8] Zhang (2020)[41]
Human parainfluenza virus -4	Cui (2015)[85]
Human parainfluenza virus - unsubtyped	Huang (2019)[47] Li (2013)[101] Therese (2021)[31] Stacy (2021)[26] Wei (2016)[71] Yan (2015)[87] Cheng (2022)[18] Liu (2019)[129] Mariana (2004)[126] You (2022)[7] Lin (2015)[86]
Overall human parainfluenza virus (HPIV)	Eun (2021)[20] Jean-Baptist (2009)[115] Ji (2011)[109] Maria (2009)[117] Chen (2016)[76] Zheng (2014)[92] Maria (2009)[117] Ming (2022)[13] Sun (2011)[110] Zhang (2022)[8] Zhang (2020)[41] Cui (2015)[85] Huang (2019)[47] Li (2013)[101] Therese (2021)[31] Stacy (2021)[26] Wei (2016)[71] Yan (2015)[87] Cheng (2022)[18] Liu (2019)[129] Mariana (2004)[126] You (2022)[7] Lin (2015)[86]
**HMPV**	
Human metapneumoviruses (HMPV)	Rory (2019)[46] Nicklas (2016)[74] Liu (2019)[129] Jean-Baptist (2009)[115] Chen (2012)[103] Maria (2009)[117] Magali (2018)[54] Yong (2019)[52] Li (2013)[101] Wang (2013)[102] You (2022)[7] Ji (2011)[109] Cheng (2022)[18] Wang (2011)[111]
**HRV**	
Human rhinovirus (HRV)	Jean-Baptist (2009)[115] Giselmo (2020)[44] Cui (2015)[85] Tiina (2016)[79] Yan (2017)[67] Jaqueline (2016)[78] You (2022)[7] Dong (2021)[30] Benjamin (2018)[61] Zhou (2018)[62] Zhou (2017)[69] Huang Huang (2015)[88]
**HAdV**	
Human adenovirus (HAdV)	Zheng (2014)[92] Nicklas (2016)[74] Jean-Baptist (2009)[115] Cheng (2022)[18] Chen (2014)[93] Rory (2019)[46] Therese (2021)[31] Li (2013)[101] You (2022)[7] Mariana (2004)[126] Eun (2021)[27] Ji (2011)[109] Lin (2015)[86] Chen (2012)[105] Chen (2016)[76] Zhang (2022)[8]
**HBoV**	
Human bocavirus -1	Wen-Kuan (2018)[55]
Human bocavirus -unsubtyped	Cui (2015)[85] Cheng (2022)[18] Chen (2014)[93] You (2022)[7] Nicklas (2016)[74] Chen (2012)[103] Ji (2011)[109] He (2019)[49]
Overall human bocavirus virus (HBoV)	Cui (2015)[85] Cheng (2022)[18] Wen-Kuan (2018)[55] Chen (2014)[103] You (2022)[7] Nicklas (2016)[74] Chen (2014)[103] Ji (2011)[109] He (2019)[49]
**EV**	
Enterovirus (EV)	You (2022)[7] Cui (2015)[85] Jean-Baptist (2009)[115] Wei (2016)[71]
**CoV**	
HCoV -229E	Jang (2020)[36]
HCoV -OC43	Jang (2020)[36]
HCoV -unsubtyped	You (2022)[7] Jean-Baptist (2009)[115] Nicklas (2016)[74] Cui (2015)[85] Wei (2016)[71]
MERS-CoV	Asmaa (2020)[38]
SARS-CoV-2	Shiv (2020)[40] Aleix (2021)[21] Keita (2022)[17] Chen (2021)[25] Sung (2022)[15] Lisa (2022)[16] Kong (2020)[43] Simone (2020)[42]
SARS-CoV	Bi (2006)[123] Feng (2005)[125] Liu (2021)[22] Huang (2004)[127]
**HRV/EV**	
Human rhinovirus/enterovirus (HRV/EV)	Cheng (2022)[18] Raymundo (2023)[1]
**Bacterial infection**	
Streptococcus pneumoniae	Susana (2012)[108] Cristiana (2010)[113] Katerina (2019)[53] Onozuka (2009)[118]
Streptococcus pyogenes pharyngitis	Daniel (2015)[83]
Moraxella catarrhalis	Huang (2019)[47] Sun (2014)[90]
Pseudomonas aeruginosa	PSOTER (2016)[73]
Legionella pneumophila	Susana (2012)[108] Lin (2015)[86]
Haemophilus influenzae	Huang (2022)[12]
Mycoplasma pneumoniae	Jean-Baptist (2009)[115] Chen (2012)[103] Chen (2013)[94] Huang (2019)[47] Tian (2017)[68] Na (2022)[10] Wang (2017)[65] Lei (2021)[32] Onozuka (2008)[120] Yuan (2022)[9] Lin (2015)[86] Wang (2013)[97] Wu (2019)[50] Na (2021)[23] Ni (2014)[89] Yin (2013)[99] Zheng (2013)[98] Shi (2016)[75], Chen (2013)[100]

Classified as ECDC NORMAL

Chlamydia pneumoniae	Huang (2019)[47] Chen (2013)[94] Jean-Baptist (2009)[115] Lin (2015)[86]
Note: Studies that were included in systematic reviews but not in meta-analyses: Mohamed (2021)[33] Morley (2018)[56] Hermann (2018)[57] Loh (2011)[112] Yuan (2006)[122] Tang (2009)[119] Sobral (2020)[77] Tamara (2009)[137]	

Classified as ECDC NORMAL

Table S13. Meta-regression results

Outcome	Covariates	Coef.	Std. Err.	Z	P>|z|	Ci.lb	Ci.ub
**RSV**	**National income level**						
	High	.2724197	0.4951057	0.55	0.583	-.7095057	1.254345
	Upper-middle	-.3211209	0.4932868	-0.65	0.517	-1.299439	0.657197
	Lower-middle	-.1973232	1.780681	-0.11	0.912	-3.728884	-3.728884
	Low	1.765932	2.549974	0.69	0.489	-3.231925	6.763789
	**WHO-region**						
	Africa	.8373415	1.45493	0.58	0.565	-2.014268	3.688951
	Americas	.3209991	1.064084	0.30	0.763	-1.764567	2.406565
	Eastern Mediterranean	-1.275594	1.838391	-0.69	0.488	-4.878774	2.327586
	Europe	-.9819557	.5239502	-1.87	0.064	-2.021087	.0571759
	South-East Asia	1.765932	2.549974	0.69	0.489	-3.231925	6.763789
	Western Pacifc	0.7205144	0.4944524	1.46	0.148	-.2601152	1.701144
	**Study Design**						
	cohort	-.8153311	.4878452	-1.67	0.095	-1.77149	.140828
	case-control	.8004486	1.203324	0.67	0.506	-1.558023	3.158921
	cross-sectional	-.0459937	.5319277	-0.09	0.931	-1.088553	.9965654
	case-crossover	-.3570562	1.784618	-0.20	0.841	-3.854844	3.140732
	time-series	1.268819	.6338912	2.00	**0.045**	.0264152	2.511223
	**Age**	1.188387	0.7958024	1.49	0.138	-.3898992	2.766673
**IV**	**National income level**						
	High	-.5391765	.4383816	-1.23	0.219	-1.398389	.3200357
	Low	.3938516	1.213169	0.32	0.745	-1.983917	2.77162
	Upper-middle	.4604176	.426858	1.08	0.281	-.3762086	1.297044
	**WHO-region**						
	Africa	.8025995	.9249863	0.87	0.386	-1.01034	2.615539
	Americas	-.4676699	.5736834	-0.82	0.415	-1.592069	.6567289
	Europe	-.0751581	.0119288	-6.30	**0.000**	-.0985382	-.051778
	South-East Asia	.3938516	1.213169	0.32	0.745	-1.983917	2.77162
	Western Pacifc	.3576141	.4337681	0.82	0.410	-.4925558	1.207784
	**Study Design**						
	cohort	-.078567	.0118534	-6.63	**0.000**	-.1017992	-.0553349
	cross-sectional	-.4525397	.3953467	-1.14	0.252	-1.227405	.3223255
	case-crossover	2.617493	1.729004	1.51	0.130	-.7712925	6.006278
	time-series	-.5498703	.389102	-1.41	0.158	-1.312496	.2127556
	**Age**	.5640363	.3840765	1.47	0.142	-.1887397	1.316812
**PIV**	**National income level**						
	High	-1.278616	.5058212	-2.53	**0.011**	-2.270007	-.2872246
	Low	.9196676	.9207501	1.00	0.318	-.8849693	2.724305
	Upper-middle	.8291939	.4922186	1.68	0.092	-.1355368	1.793925
	**WHO-region**						
	Africa	-.7801076	.9954686	-0.78	0.433	-2.73119	1.170975
	Americas	-2.247165	1.454936	-1.54	0.122	-5.098788	.6044577
	Europe	-1.340657	.9798861	-1.37	0.171	-3.261198	.5798848
	South-East Asia	.9196676	.9207501	1.00	0.318	-.8849693	2.724305
	Western Pacifc	.6947843	.565879	1.23	0.220	-.4143182	1.803887
	**Study Design**						
	case-control	1.068844	.8694856	1.23	0.219	-.6353161	2.773005
	cohort	.7647135	.6160566	1.24	0.214	-.4427353	1.972162
	case-crossover	-1.124318	1.514906	-0.74	0.458	-4.093479	1.844843
	cross-sectional	1.503414	.4132066	3.64	**0.000**	.6935438	2.313284
	time-series	.8719242	.5420643	1.61	0.108	-.1905024	1.934351
	ecological	-.9416197	1.381015	-0.68	0.495	-3.648359	1.76512
	**Age**	-.2108411	.8557342	-0.25	0.805	-1.888049	1.466367
**HMPV**	**National income level**						
	High	.4654326	.4281547	1.09	0.277	-.3737352	1.3046
	Low	.1921004	1.005379	0.19	0.848	-1.778406	2.162607
	Upper-middle	-.5276054	.4393033	-1.20	0.230	-1.388624	.3334133
	**WHO-region**						
	Europe	-.1917908	.4546588	-0.42	0.673	-1.082906	.699324
	Western Pacifc	.1413122	.4406817	0.32	0.748	-.7224082	1.005033
	South-East Asia	.1921004	1.005379	0.19	0.848	-1.778406	2.162607
	**Study Design**						
	cohort	.9778105	.3358525	2.91	**0.004**	.3195517	1.636069
	case-crossover	-.339844	.9568592	-0.36	0.722	-2.215254	1.535565
	cross-sectional	-.6637794	.4392632	-1.51	0.131	-1.524719	.1971607
	time-series	-.681461	.537519	-1.27	0.205	-1.734979	.3720568
	**Age**	.0597434	.0614445	0.97	0.331	-.0606855	.1801724
**HRV**	**National income level** *(High vs Upper-middle)*	-1.528162	.8748558	-1.75	0.081	-3.242848	.1865236
	**WHO-region**	-1.796305	.847199	-2.12	**0.034**	-3.456785	-.1358255

Classified as ECDC NORMAL

	(Europe vs Western Pacifc)					
	**Study Design**					
	case-control	.6886622	1.646415	0.42	0.676	-2.538252	3.915577
	case-crossover	-.941739	1.122595	-0.84	0.402	-3.141984	1.258506
	cross-sectional	.910875	.9192012	0.99	0.322	-.8907263	2.712476
	cohort	.5377858	1.383341	0.39	0.697	-2.173513	3.249084
	time-series	-2.493636	1.643218	-1.52	0.129	-5.714284	.7270108
	**Age**	-1.67809	.9866331	-1.70	0.089	-3.611856	.2556748
**HAdVs**	**National income level** *(High vs Upper-middle)*	1.844764	.5307367	3.48	**0.001**	.8045392	2.884989
	**WHO-region**						
	Africa	-.4373683	1.014488	-0.43	0.666	-2.425729	1.550992
	Americas	-2.15672	1.392943	-1.55	0.122	-4.886838	.5733973
	Europe	-1.442254	.7782993	-1.85	0.064	-2.967693	.0831848
	Western Pacifc	1.647847	.5619552	2.93	**0.003**	.5464347	2.749259
	**Study Design**						
	cohort	-1.211562	.7724662	-1.57	0.117	-2.725568	.3024437
	case-crossover	-.9328839	1.473294	-0.63	0.527	-3.820487	1.954719
	cross-sectional	1.465095	.5827668	2.51	**0.012**	.3228926	2.607296
	time-series	-.4536482	.7224517	-0.63	0.530	-1.869628	.9623311
	**Age**	-.6745164	.7700138	-0.88	0.381	-2.183716	.8346829
**HBoV**	**National income level** *(High vs Upper-middle)*	.5216799	.7176844	0.73	0.467	-.8849556	1.928315
	**WHO-region** *(Europe vs Western Pacifc)*	1.16555	.7904104	1.47	0.140	-.3836257	2.714726
	**Study Design**						
	cohort	-.6837422	.1137644	-6.01	**0.000**	-.9067163	-.4607682
	case-crossover	.5538944	1.034743	0.54	0.592	-1.474165	2.581954
	cross-sectional	1.15892	.5094783	2.27	**0.023**	.160361	2.157479
	time-series	-.4482961	.8900741	-0.50	0.614	-2.192809	1.296217
	**Age**	.5538944	1.034743	0.54	0.592	-1.474165	2.581954
**EV**	**National income level** *(High vs Upper-middle)*	.9369969	.2057731	4.55	**0.000**	.5336889	1.340305
	**WHO-region** *(Europe vs Western Pacifc)*	1.132873	.2167625	5.23	**0.000**	.7080263	1.557719
	**Study Design**						
	cohort	.9369969	.2057731	4.55	**0.000**	.5336889	1.340305
	case-crossover	1.101775	.9500641	1.16	0.246	-.7603166	2.963866
	time-series	-1.132873	.2167625	-5.23	**0.000**	-1.557719	-.7080263
	**Age**	1.101775	.9500641	1.16	0.246	-.7603166	2.963866
**SARS-CoV-2**	**National income level** *(High vs Upper-middle)*	-.0344442	.0104926	-3.28	**0.001**	-.0550093	-.0138792
	**WHO-region**						
	Americas	.0116355	.0093826	1.24	0.215	-.0067541	.0300251
	Europe	-.2472766	.1011741	-2.44	**0.015**	-.4455742	-.048979
	Western Pacifc	-.0093421	.009325	-1.00	0.316	-.0276188	.0089345
	**Study Design**						
	cohort	.0255756	.0072157	3.54	**0.000**	.0114331	.039718
	case-crossover	-.275535	.0114296	-24.11	**0.000**	-.2979366	-.2531335
	time-series	.9746894	.3969559	2.46	**0.014**	.1966701	1.752709
**HCoV**	**National income level** *(High vs Upper-middle)*	2.015627	1.031223	1.95	0.051	-.0055333	4.036786
	**WHO-region** *(Europe vs Western Pacifc)*	2.057392	.9107748	2.26	**0.024**	.2723064	3.842478
	**Study Design**						
	cohort	2.125852	1.004709	2.12	**0.034**	.1566589	4.095045
	case-crossover	-2.200029	1.600112	-1.37	0.169	-5.33619	.9361322
	time-series	-1.442606	1.580981	-0.91	0.362	-4.541272	1.656059
	**Age**	-2.200029	1.600112	-1.37	0.169	-5.33619	.9361322
**SARS-CoV**	**National income level** *(High vs Upper-middle)*	-.3785387	.7851233	-0.48	0.630	-1.917352	1.160275
	**WHO-region** *(Europe vs Western Pacifc)*	-1.766721	.613975	-2.88	**0.004**	-2.97009	-.5633517
	**Study Design** *(cross-sectional vs time-series analysis)*	-.2922677	.9553481	-0.31	0.760	-2.164716	1.58018

**S. pneumoniae**	**National income level** *(High vs Upper-middle)*	-1.34663	.6411379	-2.10	**0.036**	-2.603237	-.0900225
	**WHO-region**						
	Americas	-.7572833	1.182572	-0.64	0.522	-3.075082	1.560515
	Europe	-.531455	.1121689	-4.74	**0.000**	-.751302	-.311608
	Western Pacifc	.564659	.1103552	5.12	**0.000**	.3483667	.7809513
	**Study Design** *(cohort vs time-series analysis)*	.564659	.1103552	5.12	**0.000**	.3483667	.7809513
**M. pneumoniae**	**National income level** *(High vs Upper-middle)*	1.390195	2.402839	0.58	0.563	-3.319282	6.099673
	**WHO-region** *(Europe vs Western Pacifc)*	1.770413	3.320054	0.53	0.594	-4.736772	8.277599
	**Study Design**						
	cohort	-1.225525	1.43057	-0.86	0.392	-4.02939	1.57834
	cross-sectional	1.900281	1.456086	1.31	0.192	-.9535947	4.754157
	time-series	-1.390195	2.402839	-0.58	0.563	-6.099673	3.319282
	**Age**	-2.350963	2.385202	-0.99	0.324	-7.025874	2.323947
**C. pneumoniae**	**National income level** *(High vs Upper-middle)*	5.287783	.2139942	24.71	**0.000**	4.868363	5.707204
	**WHO-region** *(Europe vs Western Pacifc)*	5.287783	.2139942	24.71	**0.000**	4.868363	5.707204
	**Study Design**						
	cohort	5.296347	.2115122	25.04	**0.000**	4.881791	5.710903
	cross-sectional	-2.004481	3.697434	-0.54	0.588	-9.251319	5.242357
	time-series	-5.287783	.2139942	-24.71	**0.000**	-5.707204	-4.868363

Crl = credible interval. RSV: Respiratory syncytial virus; IV: influenza virus; HAdV: human adenovirus; HPIV: human parainfluenza virus; HMPV: human metapneumovirus; HBoV: human bocavirus; HRV: human rhinovirus; EV: enterovirus; HCoV: human coronavirus; SARS-CoV: Severe Acute Respiratory Syndrome Coronavirus; *S. pneumoniae*: *Streptococcus pneumoniae*; *M. pneumoniae: Mycoplasma pneumoniae; S. pneumoniae: C. pneumoniae: Chlamydophila pneumoniae*

Classified as ECDC NORMAL

Table S14: Summary of the assessment of the quality and strength of the evidence on ambient temperature as a risk factor for incidence of viral, mycoplasma and chlamydia respiratory infections

Reference	Ambient temperature	Respiratory syncytial virus (RSV) (n = 62)
Quality factor	Rating	Basis
**i. Downgrade**		
Risk of bias across studies	-1	Most studies carry a substantial risk of bias.
Indirectness	0	The studies assessed population, exposure, and outcome of interest morbidity was appropriate outcome, studies conducted in the population of interest, mostly direct measures of exposure.
Inconsistency	0	The magnitude of effect estimates likely to differ because of differences in study methods (study design, statistical methods, lag structure considered, model) and not be driven by unexpected heterogeneity.
Imprecision	0	We judged that the incidence CI of meta-analyses was narrow enough.
Publication bias	0	We found no reason to suspect publication bias. The search was comprehensive, and the studies were generally consistent among their findings, regardless of size or funding source.
**ii. Upgrade**		
Large magnitude of effect	0	We did not consider the estimated effects large.
Dose response	1	Most studies report dose-response patterns that are broadly similar, with incidence increasing or decreasing with ambient temperature above a certain threshold.
Confounding minimizes effect	0	No evidence found to suggest that possible residual confounders would reduce effect estimates.
**iii. Summary of the quality assessment**		
Overall quality of evidence (initial rating is ”moderate”)	*Moderate*	Moderate+(1)+(-1) = Moderate. Downgrading/upgrading resulted in moderate rating for the quality of evidence.
Summary of findings	n/a	Overall moderate quality of the evidence of the reduction in the incidence of RSV infection associated with ambient temperature exposure (see results from meta-analyses in Figure S2).
**Strength considerations**		
Quality of body of evidence	n/a	*Moderate*
Direction of effect estimate	n/a	Direction largely as expected: increased temperature reduced incidence.
Confidence in effect estimate	n/a	Ambient temperature studies directly measure outcomes of interest, influence directions are generally consistent, and most studies have a low risk of bias, especially studies with large sample sizes, but several have large confidence intervals. It is unlikely that a new study would have an effect estimate that would make the results of the meta-analysis null or insignificant.
Other compelling attributes of the data that may influence certainty	n/a	Differences in study design, exposure measures, statistical methods, consideration of lagged effects, and background factors, including completeness of incidence statistics, population exposure levels and vulnerability, and differences in physical and physiological adaptations of study populations, make interpretation and comparison difficult.
Overall strength of evidence	Sufficient	Based on our analysis and interpretation of the evidence, we conclude that there is an association between temperature exposure and RSV incidence, and we are reasonably confident that opportunity, bias, and confounding factors can be excluded as explanations for this association. The available evidence includes the results of one or more well-designed, well-conducted studies, and we believe that our conclusions are unlikely to strongly influence the outcomes of future studies.
**Reference**	**Ambient temperature**	**Influenza viruses (IV) (n = 27)**
Quality factor	Rating	Basis
**i. Downgrade**		
Risk of bias across studies	-1	Some studies are at risk of bias.

Classified as ECDC NORMAL

Indirectness	0	The studies assessed population, exposure, and outcome of interest
Inconsistency	0	The magnitude of effect estimates likely to differ because of differences in study methods (study design, statistical methods, lag structure considered, model) and not be driven by unexpected heterogeneity. The results of the meta-analysis did not appear to be strongly influenced by the individual studies. measures are consistent in the direction of overall effect estimates.
Imprecision	0	We judged that the incidence CI of meta-analyses was narrow enough.
Publication bias	0	We found no reason to suspect publication bias. The search was comprehensive, and the studies were generally consistent among their findings, regardless of size or funding source.
**ii. Upgrade**		
Large magnitude of effect	0	We did not consider the estimated effects large.
Dose response	1	Most studies report dose-response patterns that are broadly similar, with incidence increasing or decreasing with ambient temperature above a certain threshold.
Confounding minimizes effect	0	No evidence found to suggest that possible residual confounders would reduce effect estimates.
**iii. Summary of the quality assessment**		
Overall quality of evidence (initial rating is "moderate")	*Moderate*	Moderate+(1)+(-1) = Moderate. Downgrading/upgrading resulted in moderate rating for the quality of evidence.
Summary of findings from meta-analysis	n/a	We found that most of the reduction in the incidence of IFV infection associated with ambient temperature exposure (see results from meta-analyses in Figure S3).
**Strength considerations**		
Quality of body of evidence	n/a	*Moderate*
Direction of effect estimate	n/a	Direction largely as expected: increased temperature reduced incidence.
Confidence in effect estimate	n/a	Ambient temperature studies directly measure outcomes of interest, influence directions are generally consistent, and most studies have a low risk of bias, especially studies with large sample sizes, but several have large confidence intervals. It is unlikely that a new study would have an effect estimate that would make the results of the meta-analysis null or insignificant.
Other compelling attributes of the data that may influence certainty	n/a	Differences in study design, exposure measures, statistical methods, consideration of lagged effects, and background factors, including completeness of incidence statistics, population exposure levels and vulnerability, and differences in physical and physiological adaptations of study populations, make interpretation and comparison difficult.
Overall strength of evidence	Sufficient	Based on our analysis and interpretation of the evidence, we conclude that there is an association between temperature exposure and IFV incidence, and we are reasonably confident that opportunity, bias, and confounding factors can be excluded as explanations for this association. The available evidence includes the results of one or more well-designed, well-conducted studies, and we believe that our conclusions are unlikely to strongly influence the outcomes of future studies.

Reference	Ambient temperature	Human parainfluenza virus (HPIV) (n = 23)
Quality factor	Rating	Basis
**i. Downgrade**		
Risk of bias across studies	-1	There is a risk of bias in some studies.
Indirectness	0	The studies assessed population, exposure, and outcome of interest
Inconsistency	0	The magnitude of effect estimates likely to differ because of differences in study methods (study design, statistical methods, lag structure considered, model) and not be driven by unexpected heterogeneity. The results of the meta-analysis for incidence of

Classified as ECDC NORMAL

		HPIV infection do not appear to be strongly influenced by an individual study. measures are consistent in the direction of overall effect estimates.
Imprecision	0	We judged that the incidence CI of meta-analyses was narrow enough.
Publication bias	0	We found no reason to suspect publication bias. The search was comprehensive, and the studies were generally consistent among their findings, regardless of size or funding source.
**ii. Upgrade**		
Large magnitude of effect	0	We did not consider the estimated effects large.
Dose response	1	Most studies report dose-response patterns that are broadly similar, with incidence increasing or decreasing with ambient temperature above a certain threshold.
Confounding minimizes effect	0	No evidence found to suggest that possible residual confounders would reduce effect estimates.
**iii. Summary of the quality assessment**		
Overall quality of evidence (initial rating is "moderate")	*Moderate*	Moderate+(1)+(-1) = Moderate. Downgrading/upgrading resulted in moderate rating for the quality of evidence.
Summary of findings from meta-analysis	n/a	We found that most of the reduction in the incidence of HPIV infection associated with ambient temperature exposure (see results from meta-analyses in Figure S4).
**Strength considerations**		
Quality of body of evidence	n/a	*Moderate*
Direction of effect estimate	n/a	The direction of effect estimates largely showed an increasing trend in HPIV infection with increasing high temperatures.
Confidence in effect estimate	n/a	Ambient temperature studies directly measure outcomes of interest, influence directions are generally consistent, and most studies have a low risk of bias, especially studies with large sample sizes, but several have large confidence intervals. It is unlikely that a new study would have an effect estimate that would make the results of the meta-analysis null or insignificant.
Other compelling attributes of the data that may influence certainty	n/a	Differences in study design, exposure measures, statistical methods, consideration of lagged effects, and background factors, including completeness of incidence statistics, population exposure levels and vulnerability, and differences in physical and physiological adaptations of study populations, make interpretation and comparison difficult.
Overall strength of evidence	Sufficient	Based on our analysis and interpretation of the evidence, we conclude that there is an positive association between temperature exposure and HPIV incidence, and we are reasonably confident that opportunity, bias, and confounding factors can be excluded as explanations for this association. The available evidence includes the results of one or more well-designed, well-conducted studies, and we believe that our conclusions are unlikely to strongly influence the outcomes of future studies.

Reference	Ambient temperature	Human metapneumovirus (hMPV) (n = 14)
Quality factor	Rating	Basis
**i. Downgrade**		
Risk of bias across studies	-1	Some studies carry a certain risk of bias.
Indirectness	0	The studies assessed population, exposure, and outcome of interest
Inconsistency	0	The magnitude of effect estimates likely to differ because of differences in study methods (study design, statistical methods, lag structure considered, model) and not be driven by unexpected heterogeneity. The results of the meta-analysis for incidence of respiratory infection do not appear to be strongly influenced by an individual study.
Imprecision	-1	Except six studies, the 95% CIs of the studies possessed notably narrow confidence intervals.

Classified as ECDC NORMAL

Publication bias	0	We found no reason to suspect publication bias. The search was comprehensive, and the studies were generally consistent among their findings, regardless of size or funding source.
**ii. Upgrade**		
Large magnitude of effect	0	We did not consider the estimated effects large.
Dose response	1	Most studies report dose-response patterns that are broadly similar, with incidence increasing or decreasing with ambient temperature above a certain threshold.
Confounding minimizes effect	0	No evidence found to suggest that possible residual confounders would reduce effect estimates.
**iii. Summary of the quality assessment**		
Overall quality of evidence (initial rating is ”moderate”)	*Low*	Moderate+(-1)+(-1)+(1) = Low. Downgrading/upgrading resulted in low rating for the quality of evidence.
Summary of findings from meta-analysis	n/a	We found that most of the reduction in the incidence of hMPV infection associated with ambient temperature exposure (see results from meta-analyses in Figure S5).
**Strength considerations**		
Quality of body of evidence	n/a	*Low*
Direction of effect estimate	n/a	The direction of effect estimates largely showed an decreasing trend in hMPV infection with increasing temperatures.
Confidence in effect estimate	n/a	Ambient temperature studies directly measure outcomes of interest, influence directions are generally consistent, and most studies have a low risk of bias, especially studies with large sample sizes, but several have large confidence intervals. It is unlikely that a new study would have an effect estimate that would make the results of the meta-analysis null or insignificant.
Other compelling attributes of the data that may influence certainty	n/a	Differences in study design, exposure measures, statistical methods, consideration of lagged effects, and background factors, including completeness of incidence statistics, population exposure levels and vulnerability, and differences in physical and physiological adaptations of study populations, make interpretation and comparison difficult.
Overall strength of evidence	Sufficient	Based on our analysis and interpretation of the evidence, we conclude that there is an negative association between temperature exposure and hMPV incidence, and we are reasonably confident that opportunity, bias, and confounding factors can be excluded as explanations for this association. The available evidence includes the results of one or more well-designed, well-conducted studies, and we believe that our conclusions are unlikely to strongly influence the outcomes of future studies.

Reference	Ambient temperature	Human rhinovirus (HRV) (n = 12)
Quality factor	Rating	Basis
**i. Downgrade**		
Risk of bias across studies	-1	There is a substantial risk of bias across most studies.
Indirectness	0	The studies assessed population, exposure, and outcome of interest.
Inconsistency	0	The magnitude of effect estimates likely to differ because of differences in study methods (study design, statistical methods, lag structure considered, model) and not be driven by unexpected heterogeneity. The results of the meta-analysis for incidence of respiratory infection do not appear to be strongly influenced by an individual study. The results of all four metaanalyses for change in the respiratory infection. measures are consistent in the direction of overall effect estimates.
Imprecision	-1	We judged that the incidence CI of 3 studies was wide.
Publication bias	0	We found no reason to suspect publication bias. The search was comprehensive, and the studies were generally consistent among their findings, regardless of size or funding source.
**ii. Upgrade**		

Classified as ECDC NORMAL

Large magnitude of effect	0	We did not consider the estimated effects large.
Dose response	0	Random effects contain uniformity and are not statistically significant.
Confounding minimizes effect	0	No evidence found to suggest that possible residual confounders would reduce effect estimates.
**iii. Summary of the quality assessment**		
Overall quality of evidence (initial rating is ”moderate”)	*Very low*	Moderate+(-1)+(-1) = Very low. Downgrading/upgrading resulted in very low rating for the quality of evidence.
Summary of findings from meta-analysis	n/a	The random effect result contains unity and were statistically insignificant. (see results from meta-analyses in Figure S6).
Summary of qualitative findings	n/a	Studies not included in the meta-analyses presented mixed results, mostly insignificant associations.
**Strength considerations**		
Quality of body of evidence	n/a	*Very low*
Direction of effect estimate	n/a	No association was found and the results were inconclusive.
Confidence in effect estimate	n/a	Ambient temperature studies directly measure outcomes of interest, influence directions are often inconsistent, and most studies have a risk of bias, and some have large confidence intervals, which makes the results uncertain for the future.
Other compelling attributes of the data that may influence certainty	n/a	Differences in study design, exposure measures, statistical methods, consideration of lagged effects, and background factors, including completeness of incidence statistics, population exposure levels and vulnerability, and differences in physical and physiological adaptations of study populations, make interpretation and comparison difficult.
Overall strength of evidence	Limited	Based on our analysis and interpretation of the evidence, we conclude that there is no association between temperature exposure and HRV morbidity.

Reference	Ambient temperature	Human adenovirus (HAdV) (n = 16)
Quality factor	Rating	Basis
**i. Downgrade**		
Risk of bias across studies	-1	There is a risk of bias in some studies.
Indirectness	0	The studies assessed population, exposure, and outcome of interest.
Inconsistency	0	The magnitude of effect estimates likely to differ because of differences in study methods (study design, statistical methods, lag structure considered, model) and not be driven by unexpected heterogeneity. The results of the meta-analysis for incidence of respiratory infection do not appear to be strongly influenced by an individual study. The results of all four metaanalyses for change in the respiratory infection. measures are consistent in the direction of overall effect estimates.
Imprecision	-1	We judged that the incidence CI of 4 studies was wide.
Publication bias	0	We found no reason to suspect publication bias. The search was comprehensive, and the studies were generally consistent among their findings, regardless of size or funding source.
**ii. Upgrade**		
Large magnitude of effect	0	We did not consider the estimated effects large.
Dose response	0	Random effects contain uniformity and are not statistically significant.
Confounding minimizes effect	0	No evidence found to suggest that possible residual confounders would reduce effect estimates.
**iii. Summary of the quality assessment**		
Overall quality of evidence (initial rating is ”moderate”)	*Very low*	Moderate+(-1)+(-1) = Very low. Downgrading/upgrading resulted in very low rating for the quality of evidence.

Classified as ECDC NORMAL

Summary of findings from meta-analysis	n/a	Random effect contains unity and were statistically insignificant (see results from meta-analyses in Figure S7).
**Strength considerations**		
Quality of body of evidence	n/a	*Very low*
Direction of effect estimate	n/a	No association was found and the results were inconclusive.
Confidence in effect estimate	n/a	Ambient temperature studies directly measure outcomes of interest, influence directions are often inconsistent, and some studies have a risk of bias and some have large confidence intervals.
Other compelling attributes of the data that may influence certainty	n/a	Differences in study design, exposure measures, statistical methods, consideration of lagged effects, and background factors, including completeness of incidence statistics, population exposure levels and vulnerability, and differences in physical and physiological adaptations of study populations, make interpretation and comparison difficult.
Overall strength of evidence	Limited	Based on our analysis and interpretation of the evidence, we conclude that there is no association between temperature exposure and incidence, and that future studies may change our results.

Reference	Ambient temperature	Human bocavirus (HBoV) (n = 9)
Quality factor	Rating	Basis
**i. Downgrade**		
Risk of bias across studies	0	There is no indication that there is substantial risk of bias across the body of available evidence.
Indirectness	0	The studies assessed population, exposure, and outcome of interest
Inconsistency	0	The magnitude of effect estimates likely to differ because of differences in study methods (study design, statistical methods, lag structure considered, model) and not be driven by unexpected heterogeneity. The results of the meta-analysis for incidence of respiratory infection do not appear to be strongly influenced by an individual study. The results of all four metaanalyses for change in the respiratory infection. measures are consistent in the direction of overall effect estimates.
Imprecision	-1	The number of studies is small, the sample size is insufficient, and the incidence CI of 5 studies was wide.
Publication bias	0	We found no reason to suspect publication bias.The search was comprehensive, and the studies were generally consistent among their findings, regardless of size or funding source.
**ii. Upgrade**		
Large magnitude of effect	0	We did not consider the estimated effects large.
Dose response	1	Most studies report dose-response patterns that are broadly similar, with incidence increasing or decreasing with ambient temperature above a certain threshold.
Confounding minimizes effect	0	No evidence found to suggest that possible residual confounders would reduce effect estimates.
**iii. Summary of the quality assessment**		
Overall quality of evidence (initial rating is ”moderate”)	*Moderate*	Moderate+(1)+(-1) = Moderate. Downgrading/upgrading resulted in moderate rating for the quality of evidence.
Summary of findings from meta-analysis	n/a	We found that most of the increase in the incidence of HBoV infection associated with ambient temperature exposure (see results from meta-analyses in Figure S8).
**Strength considerations**		
Quality of body of evidence	n/a	*Moderate*
Direction of effect estimate	n/a	Direction largely as expected: higher risk of incidence at high ambient temperatures.
Confidence in effect estimate	n/a	Ambient temperature studies directly measure outcomes of interest, influence directions are generally consistent, and most

Classified as ECDC NORMAL

		studies have a low risk of bias, especially studies with large sample sizes, but several have large confidence intervals. It is unlikely that a new study would have an effect estimate that would make the results of the meta-analysis null or insignificant.
Other compelling attributes of the data that may influence certainty	n/a	Differences in study design, exposure measures, statistical methods, consideration of lagged effects, and background factors, including completeness of incidence statistics, population exposure levels and vulnerability, and differences in physical and physiological adaptations of study populations, make interpretation and comparison difficult.
Overall strength of evidence	Limited	Based on our analysis and interpretation of the evidence, we conclude that there is an positive association between temperature exposure and HBoV incidence, and we are reasonably confident that opportunity, bias, and confounding factors can be excluded as explanations for this association. The available evidence includes the results of one or more well-designed, well-conducted studies, and we believe that our conclusions are unlikely to strongly influence the outcomes of future studies.

Reference	Ambient temperature	Enterovirus (EV) (n = 4)
Quality factor	Rating	Basis
**i. Downgrade**		
Risk of bias across studies	0	There is no indication that there is substantial risk of bias across the body of available evidence.
Indirectness	0	The studies assessed population, exposure, and outcome of interest
Inconsistency	0	The magnitude of effect estimates likely to differ because of differences in study methods (study design, statistical methods, lag structure considered, model) and not be driven by unexpected heterogeneity. The results of the meta-analysis for incidence of EVs infection do not appear to be strongly influenced by an individual study. The results of all four meta-analyses for change in the respiratory infection. measures are consistent in the direction of overall effect estimates.
Imprecision	-1	The number of studies is small and the sample size is insufficient.
Publication bias	0	We found no reason to suspect publication bias. The search was comprehensive, and the studies were generally consistent among their findings, regardless of size or funding source.
**ii. Upgrade**		
Large magnitude of effect	0	We did not consider the estimated effects large.
Dose response	0	No dose-response relationship was found.
Confounding minimizes effect	0	No evidence found to suggest that possible residual confounders would reduce effect estimates.
**iii. Summary of the quality assessment**		
Overall quality of evidence (initial rating is ”moderate”)	*Low*	Moderate+(-1)= Low. Downgrading/upgrading resulted in low rating for the quality of evidence.
Summary of findings from meta-analysis	n/a	We found that most of the reduction in the incidence of respiratory infection associated with ambient temperature exposure (see results from meta-analyses in Figure S9).
**Strength considerations**		
Quality of body of evidence	n/a	*Low*
Direction of effect estimate	n/a	The overall low quality evidence showed no association between temperature exposure and EVs morbidity.
Confidence in effect estimate	n/a	Ambient temperature studies directly measure results of interest, influence directions are inconsistent, the number of studies is small, and one study has a large confidence interval.
Other compelling attributes of the data that may influence certainty	n/a	Differences in study design, exposure measures, statistical methods, consideration of lagged effects, and background factors, including completeness of incidence statistics, population exposure levels and vulnerability, and differences in physical and

Classified as ECDC NORMAL

		physiological adaptations of study populations, make interpretation and comparison difficult.
Overall strength of evidence	Sufficient	Based on our analysis and interpretation of the evidence, we conclude that there is no association between temperature exposure and EVs incidence, and that the addition of future studies may change the current results.

Reference	Ambient temperature	Severe acute respiratory syndrome coronavirus 2 (SARS-CoV-2) (n =8)
Quality factor	Rating	Basis
**i. Downgrade**		
Risk of bias across studies	0	There is no indication that there is substantial risk of bias across the body of available evidence.
Indirectness	0	The studies assessed population, exposure, and outcome of interest.
Inconsistency	0	The magnitude of effect estimates likely to differ because of differences in study methods (study design, statistical methods, lag structure considered, model) and not be driven by unexpected heterogeneity. The results of the meta-analysis for incidence of respiratory infection do not appear to be strongly influenced by an individual study. measures are consistent in the direction of overall effect estimates.
Imprecision	-1	We judged that the incidence CI of one study was wide and the remaining CI was narrow enough.
Publication bias	0	We found no reason to suspect publication bias. The search was comprehensive, and the studies were generally consistent among their findings, regardless of size or funding source.
**ii. Upgrade**		
Large magnitude of effect	0	We did not consider the estimated effects large.
Dose response	1	Most studies report dose-response patterns that are broadly similar, with incidence increasing or decreasing with ambient temperature above a certain threshold.
Confounding minimizes effect	0	No evidence found to suggest that possible residual confounders would reduce effect estimates.
**iii. Summary of the quality assessment**		
Overall quality of evidence (initial rating is ”moderate”)	*Moderate*	Moderate+(1)+(-1) = Moderate. Downgrading/upgrading resulted in moderate rating for the quality of evidence.
Summary of findings from meta-analysis	n/a	We found that most of the reduction in the incidence of respiratory infection associated with ambient temperature exposure (see results from meta-analyses in Figure S10).
**Strength considerations**		
Quality of body of evidence	n/a	*Moderate*
Direction of effect estimate	n/a	The direction of effect estimates largely showed an decreasing trend in COVID-19 infection with increasing high temperatures.
Confidence in effect estimate	n/a	Ambient temperature studies directly measure outcomes of interest, influence directions are usually consistent, and studies have a low risk of bias, especially studies with large sample sizes, but some studies have large confidence intervals. A new study is unlikely to have an effect estimate that would render the results of the meta-analysis invalid or not significant.
Other compelling attributes of the data that may influence certainty	n/a	Differences in study design, exposure measures, statistical methods, consideration of lagged effects, and background factors, including completeness of incidence statistics, population exposure levels and vulnerability, and differences in physical and physiological adaptations of study populations, make interpretation and comparison difficult.
Overall strength of evidence	Sufficient	Based on our analysis and interpretation of the evidence, we conclude that there is an negative association between temperature exposure and COVID-19 incidence, and we are reasonably confident that opportunity, bias, and confounding factors can be excluded as explanations for this association. The available evidence includes the results of one or more well-designed, well-conducted studies, and we believe that our

Classified as ECDC NORMAL

		conclusions are unlikely to strongly influence the outcomes of future studies.

Reference	Ambient temperature	Severe acute respiratory syndrome coronavirus (SARS-CoV) (n=4)
Quality factor	Rating	Basis
**i. Downgrade**		
Risk of bias across studies	-1	There is a risk of bias in some studies.
Indirectness	0	The studies assessed population, exposure, and outcome of interest.
Inconsistency	0	The magnitude of effect estimates likely to differ because of differences in study methods (study design, statistical methods, lag structure considered, model) and not be driven by unexpected heterogeneity. The results of the meta-analysis for incidence of respiratory infection do not appear to be strongly influenced by an individual study. The results of all four meta-analyses for change in the respiratory infection. measures are consistent in the direction of overall effect estimates.
Imprecision	-1	The number of studies is small and the sample size is insufficient.
Publication bias	0	We found no reason to suspect publication bias. The search was comprehensive, and the studies were generally consistent among their findings, regardless of size or funding source.
**ii. Upgrade**		
Large magnitude of effect	0	We did not consider the estimated effects large.
Dose response	1	Most studies report dose-response patterns that are broadly similar, with incidence increasing or decreasing with ambient temperature above a certain threshold.
Confounding minimizes effect	0	No evidence found to suggest that possible residual confounders would reduce effect estimates.
**iii. Summary of the quality assessment**		
Overall quality of evidence (initial rating is ”moderate”)	*Low*	Moderate+(1)+(-1)+(-1) = Low. Downgrading/upgrading resulted in low rating for the quality of evidence.
Summary of findings from meta-analysis	n/a	Low quality evidence did not show that the incidence of SARS infection was related to ambient temperature exposure (see results from meta-analyses in Figure S11).
**Strength considerations**		
Quality of body of evidence	n/a	*Low*
Direction of effect estimate	n/a	The direction of effect estimates did not show an association between temperature and SARS infection, and the results were inconclusive.
Confidence in effect estimate	n/a	Ambient temperature studies directly measure outcomes of interest, influence directions are generally consistent, and most studies have a low risk of bias, but several have large confidence intervals. It is unlikely that a new study would have an effect estimate that would make the results of the meta-analysis null or insignificant.
Other compelling attributes of the data that may influence certainty	n/a	Differences in study design, exposure measures, statistical methods, consideration of lagged effects, and background factors, including completeness of incidence statistics, population exposure levels and vulnerability, and differences in physical and physiological adaptations of study populations, make interpretation and comparison difficult.
Overall strength of evidence	Limited	Based on our analysis and interpretation of the evidence, we did not find an association between temperature exposure and SARS morbidity, and the available evidence is insufficient.

Reference	Ambient temperature	Human coronavirus (HCoV) (n=5)
Quality factor	Rating	Basis
**i. Downgrade**		
Risk of bias across studies	0	There is no indication that there is substantial risk of bias across the body of available evidence.

Classified as ECDC NORMAL

Indirectness	0	The studies assessed population, exposure, and outcome of interest.
Inconsistency	0	The magnitude of effect estimates likely to differ because of differences in study methods (study design, statistical methods, lag structure considered, model) and not be driven by unexpected heterogeneity. The results of the meta-analysis for incidence of HCoV infection do not appear to be strongly influenced by an individual study. Measures are consistent in the direction of overall effect estimates.
Imprecision	-1	The number of studies is small and the sample size is insufficient.
Publication bias	0	We found no reason to suspect publication bias. The search was comprehensive, and the studies were generally consistent among their findings, regardless of size or funding source.
**ii. Upgrade**		
Large magnitude of effect	0	We did not consider the estimated effects large.
Dose response	1	Most studies report dose-response patterns that are broadly similar, with incidence increasing or decreasing with ambient temperature above a certain threshold.
Confounding minimizes effect	0	No evidence found to suggest that possible residual confounders would reduce effect estimates.
**iii. Summary of the quality assessment**		
Overall quality of evidence (initial rating is ”moderate”)	*Moderate*	Moderate+(1)+(-1) = Moderate. Downgrading/upgrading resulted in moderate rating for the quality of evidence.
Summary of findings from meta-analysis	n/a	We found that most of the reduction in the incidence of HCoV infection associated with ambient temperature exposure (see results from meta-analyses in Figure S12).
**Strength considerations**		
Quality of body of evidence	n/a	*Moderate*
Direction of effect estimate	n/a	Direction largely as expected: increased temperature reduced incidence.
Confidence in effect estimate	n/a	Ambient temperature studies directly measure outcomes of interest, influence directions are generally consistent, and most studies have a low risk of bias, but several have large confidence intervals. It is unlikely that a new study would have an effect estimate that would make the results of the meta-analysis null or insignificant.
Other compelling attributes of the data that may influence certainty	n/a	Differences in study design, exposure measures, statistical methods, consideration of lagged effects, and background factors, including completeness of incidence statistics, population exposure levels and vulnerability, and differences in physical and physiological adaptations of study populations, make interpretation and comparison difficult.
Overall strength of evidence	Sufficient	Based on our analysis and interpretation of the evidence, we conclude that there is an negative association between temperature exposure and HCoV incidence, and we are reasonably confident that opportunity, bias, and confounding factors can be excluded as explanations for this association. The available evidence includes the results of one or more well-designed, well-conducted studies, and we believe that our conclusions are unlikely to strongly influence the outcomes of future studies.

Reference	Ambient temperature	MERS-CoV (n =1)
Quality factor	Rating	Basis
**i. Downgrade**		
Risk of bias across studies	0	No risk of bias was found.
Indirectness	0	The studies assessed population, exposure, and outcome of interest.
Inconsistency	0	The magnitude of effect estimates likely to differ because of differences in study methods (study design, statistical methods,

Classified as ECDC NORMAL

		lag structure considered, model) and not be driven by unexpected heterogeneity.
Imprecision	-1	The number of studies is small and the sample size is insufficient.
Publication bias	0	We found no reason to suspect publication bias. The search was comprehensive, and the studies were generally consistent among their findings, regardless of size or funding source.
**ii. Upgrade**		
Large magnitude of effect	0	We did not consider the estimated effects large.
Dose response	1	As ambient temperature increases, morbidity increases in dose-response patterns.
Confounding minimizes effect	0	No evidence found to suggest that possible residual confounders would reduce effect estimates.
**iii. Summary of the quality assessment**		
Overall quality of evidence (initial rating is ”moderate”)	*Moderate*	Moderate+(1)+(-1) = Moderate. Downgrading/upgrading resulted in moderate rating for the quality of evidence.
Summary of findings from meta-analysis	n/a	We found that the increased incidence of respiratory infections was associated with ambient temperature exposure (see results from meta-analyses in Figure S9).
**Strength considerations**		
Quality of body of evidence	n/a	*Moderate*
Direction of effect estimate	n/a	The incidence of respiratory pathogens increased with the increase of temperature.
Confidence in effect estimate	n/a	Ambient temperature studies directly measure outcomes of interest, influence directions are usually consistent, studies have low risk of bias, small sample sizes, and large confidence intervals. The emergence of a new study may invalidate or make significant the results of the meta-analysis.
Other compelling attributes of the data that may influence certainty	n/a	Differences in study design, exposure measures, statistical methods, consideration of lagged effects, and background factors, including completeness of incidence statistics, population exposure levels and vulnerability, and differences in physical and physiological adaptations of study populations, make interpretation and comparison difficult.
Overall strength of evidence	Sufficient	Based on our analysis and interpretation of the evidence, we conclude that there is an association between temperature exposure and incidence, and we have reason to believe that chance, bias, and confounding factors can be excluded as explanations for this association. The available evidence includes the results of only one well-designed study, and our conclusions are unlikely to strongly influence the results of future studies.

Reference	Ambient temperature	Human rhinovirus/enterovirus (HRV/EV) (n = 2)
Quality factor	Rating	Basis
**i. Downgrade**		
Risk of bias across studies	0	There is no indication that there is substantial risk of bias across the body of available evidence.
Indirectness	0	The studies assessed population, exposure, and outcome of interest
Inconsistency	0	The magnitude of effect estimates likely to differ because of differences in study methods (study design, statistical methods, lag structure considered, model) and not be driven by unexpected heterogeneity. The results of the meta-analysis for incidence of HRVs/EVs infection do not appear to be strongly influenced by an individual study.
Imprecision	-1	The number of studies is small and the sample size is insufficient.
Publication bias	0	We found no reason to suspect publication bias. The search was comprehensive, and the studies were generally consistent among their findings, regardless of size or funding source.
**ii. Upgrade**		
Large magnitude of effect	0	We did not consider the estimated effects large.

Classified as ECDC NORMAL

Dose response	1	Most studies report dose-response patterns that are broadly similar, with incidence increasing or decreasing with ambient temperature above a certain threshold.
Confounding minimizes effect	0	No evidence found to suggest that possible residual confounders would reduce effect estimates.
**iii. Summary of the quality assessment**		
Overall quality of evidence (initial rating is ”moderate”)	*Moderate*	Moderate+(1)+(-1) = Moderate. Downgrading/upgrading resulted in moderate rating for the quality of evidence.
Summary of findings from meta-analysis	n/a	We found that the increase in the incidence of HRVs/EVs infection associated with ambient temperature rise (see results from meta-analyses in Figure S13).
**Strength considerations**		
Quality of body of evidence	n/a	*Moderate*
Direction of effect estimate	n/a	Direction largely as expected: higher risk of incidence at high ambient temperatures.
Confidence in effect estimate	n/a	Ambient temperature studies directly measure outcomes of interest, influence directions are generally consistent, and most studies have a low risk of bias, especially studies with large sample sizes, but several have large confidence intervals. It is unlikely that a new study would have an effect estimate that would make the results of the meta-analysis null or insignificant.
Other compelling attributes of the data that may influence certainty	n/a	Differences in study design, exposure measures, statistical methods, consideration of lagged effects, and background factors, including completeness of incidence statistics, population exposure levels and vulnerability, and differences in physical and physiological adaptations of study populations, make interpretation and comparison difficult.
Overall strength of evidence	Limited	Based on our analysis and interpretation of the evidence, we conclude that there is a positive association between temperature exposure and the incidence of HRV/EVs, but this contains only a small amount of available evidence.

n/a, not applicable. “0” quality rating indicates there were no upgrades or downgrades for each factor being evaluated across the body of evidence; –1=downgrade; +1=upgrade.

Table S15: Summary of the assessment of the quality and strength of the evidence on ambient temperature as a risk factor for incidence of bacterial respiratory infections

Reference	Ambient temperature					
	Streptococcus pneumoniae	*Streptococcus pyogenes pharyngiti*	*Moraxella catarrhalis*	*Pseudomonas aeruginosa*	*Legionella pneumoniae*	*Haemophilus influenzae*
Initial rating	Moderate	Moderate	Moderate	Moderate	Moderate	Moderate
Quality factor						
**i. Downgrade**						
Risk of bias across studies	-1	-1	0	-1	0	-1
Indirectness	0	0	0	0	0	0
Inconsistency	0	n/a	-1	n/a	-1	n/a
Imprecision	-1	-1	-1	-1	-1	0
Publication bias	0	0	0	0	0	0
**ii. Upgrade**						
Large magnitude of effect	0					
Dose response	0	+1	0	+1	+1	0
Confounding minimizes effect	0	0	0	0	0	0

Classified as ECDC NORMAL

**iii. Summary of the quality assessment**						
Overall quality of evidence (initial rating is ”moderate”)	*Very Low*	*Low*	*Very Low*	*Moderate*	*Low*	*Low*
Summary of findings from meta-analysis	n/a	n/a	n/a	n/a	n/a	n/a
Summary of qualitative findings	n/a	n/a	n/a	n/a	n/a	n/a
**Strength considerations**						
Quality of body of evidence	n/a	n/a	n/a	n/a	n/a	n/a
Direction of effect estimate	n/a	n/a	n/a	n/a	n/a	n/a
Confidence in effect estimate	n/a	n/a	n/a	n/a	n/a	n/a
Other compelling attributes of the data that may influence certainty	n/a	n/a	n/a	n/a	n/a	n/a
Overall strength of evidence	Limited	Limited	Limited	Limited	Limited	Limited

n/a, not applicable. “0” quality rating indicates there were no upgrades or downgrades for each factor being evaluated across the body of evidence; –1=downgrade; +1=upgrade.

Classified as ECDC NORMAL

Table S16: Summary of the assessment of the quality and strength of the evidence on ambient temperature as a risk factor for incidence of mycoplasma and chlamydia respiratory infections

Reference	Ambient temperature	Mycoplasma pneumoniae (n =19)
Quality factor	Rating	Basis
**i. Downgrade**		
Risk of bias across studies	-1	There is a substantial risk of bias across most studies.
Indirectness	0	The studies assessed population, exposure, and outcome of interest
Inconsistency	0	The magnitude of effect estimates likely to differ because of differences in study methods (study design, statistical methods, lag structure considered, model) and not be driven by unexpected heterogeneity. The results of the meta-analysis for incidence of respiratory infection do not appear to be strongly influenced by an individual study. Measures are consistent in the direction of overall effect estimates.
Imprecision	-1	We judged that the incidence CI of one study was wide.
Publication bias	0	We found no reason to suspect publication bias. The search was comprehensive, and the studies were generally consistent among their findings, regardless of size or funding source.
**ii. Upgrade**		
Large magnitude of effect	0	We did not consider the estimated effects large.
Dose response	0	No significant dose-response relationship was found.
Confounding minimizes effect	0	No evidence found to suggest that possible residual confounders would reduce effect estimates.
**iii. Summary of the quality assessment**		
Overall quality of evidence (initial rating is ”moderate”)	*Very low*	Moderate+(-1)+(-1)=Very low. Downgrading/upgrading resulted in very low rating for the quality of evidence.
Summary of findings from meta-analysis	n/a	The quality of evidence was generally low, and the incidence of Mycoplasma pneumoniae infections was not associated with ambient temperature (see results from meta-analyses in Figure S17).
Summary of qualitative findings	n/a	Studies not included in the meta-analyses presented mixed results, mostly insignificant associations.
**Strength considerations**		
Quality of body of evidence	n/a	*Low*
Direction of effect estimate	n/a	The direction of effect estimates did not show an association between temperature and mycoplasma infection, and the results were inconclusive.
Confidence in effect estimate	n/a	Ambient temperature studies directly measure outcomes of interest, influence directions are inconsistent, and due to these methodological deficiencies, it cannot be ruled out that new studies may show different estimates of effects.
Other compelling attributes of the data that may influence certainty	n/a	Differences in study design, exposure measures, statistical methods, consideration of lagged effects, and background factors, including completeness of incidence statistics, population exposure levels and vulnerability, and differences in physical and physiological adaptations of study populations, make interpretation and comparison difficult.
Overall strength of evidence	Limited	Based on our analysis and interpretation of the evidence, we conclude that there is no association between temperature exposure and morbidity.
Reference	Ambient temperature	Chlamydophila pneumoniae (n =4)
Quality factor	Rating	Basis
**i. Downgrade**		
Risk of bias across studies	-1	There is a risk of bias in some studies.
Indirectness	0	The studies assessed population, exposure, and outcome of interest.

Classified as ECDC NORMAL

Inconsistency	0	The magnitude of effect estimates likely to differ because of differences in study methods (study design, statistical methods, lag structure considered, model) and not be driven by unexpected heterogeneity. The results of the meta-analysis for incidence of respiratory infection do not appear to be strongly influenced by an individual study.
Imprecision	-1	The number of studies is small and the sample size is insufficient.
Publication bias	0	We found no reason to suspect publication bias. The search was comprehensive, and the studies were generally consistent among their findings, regardless of size or funding source.
**ii. Upgrade**		
Large magnitude of effect	0	We did not consider the estimated effects large.
Dose response	0	Most studies have found no effect of temperature on chlamydophila pneumoniae morbidity.
Confounding minimizes effect	0	No evidence found to suggest that possible residual confounders would reduce effect estimates.
**iii. Summary of the quality assessment**		
Overall quality of evidence (initial rating is ”moderate”)	*Very low*	Moderate+(1)+(-1)+(-1) = Very low. Downgrading/upgrading resulted in very low rating for the quality of evidence.
Summary of findings from meta-analysis	n/a	Very low-quality evidence suggests that the incidence of chlamydophila pneumoniae infections is not associated with ambient temperature exposure (see meta-analysis results in Figure S18).
Summary of qualitative findings	n/a	Studies not included in the meta-analyses presented mixed results, mostly insignificant associations.
**Strength considerations**		
Quality of body of evidence	n/a	*Very low*
Direction of effect estimate	n/a	Effect estimates showed no significant difference in temperature and respiratory infections, making the results inconclusive.
Confidence in effect estimate	n/a	No trend was observed in overall random effects, and the number of studies included in the meta-analysis was small, making the results uncertain for the future.
Other compelling attributes of the data that may influence certainty	n/a	Differences in study design, exposure measures, statistical methods, consideration of lagged effects, and background factors, including completeness of incidence statistics, population exposure levels and vulnerability, and differences in physical and physiological adaptations of study populations, make interpretation and comparison difficult.
Overall strength of evidence	Limited	No association was observed, the quality of evidence was "very low", and a small number of studies led to "limited" evidence to draw conclusions about the association between respiratory infections and temperature.

n/a, not applicable. “0” quality rating indicates there were no upgrades or downgrades for each factor being evaluated across the body of evidence; –1=downgrade; +1=upgrade.

Classified as ECDC NORMAL

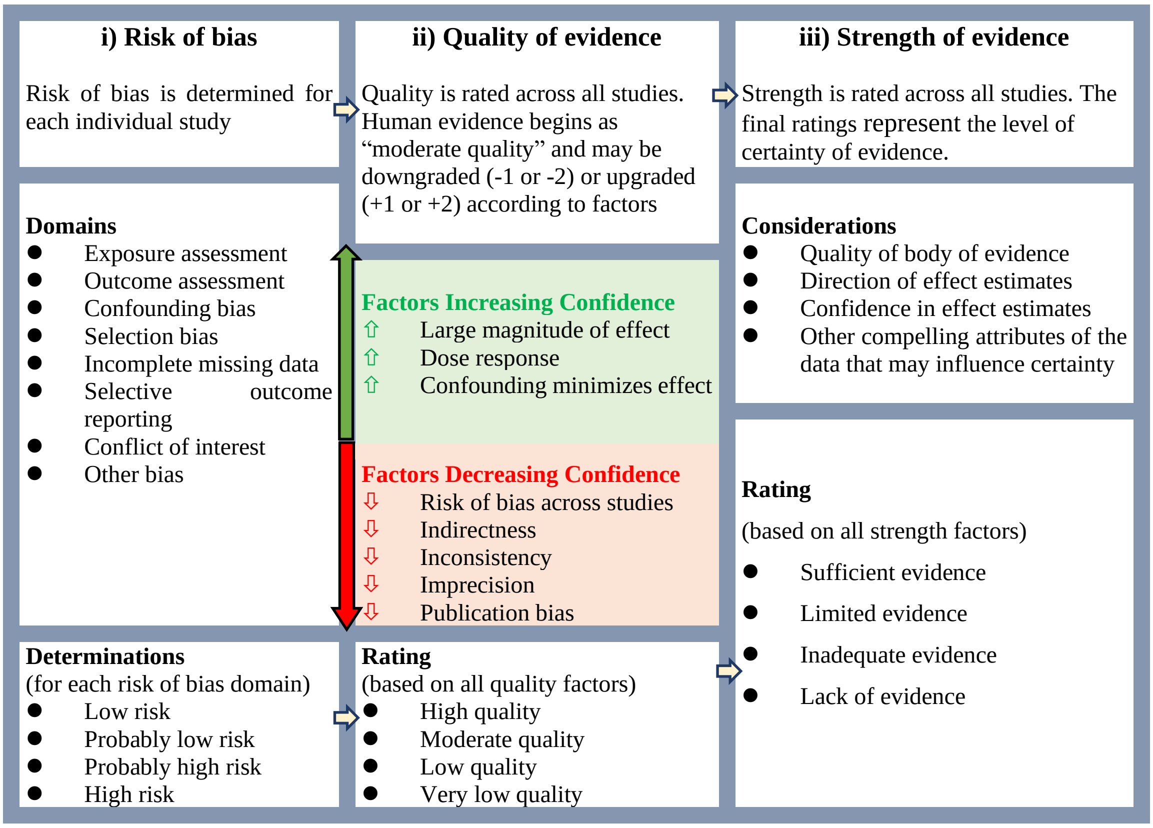

Figure X1 | Overview of Navigation Guide systematic review methodology used for rating the quality and strength of the human evidence.

Picture shows three parts of evidence evaluation, namely risk of bias, quality of evidence, and strength of evidence, respectively showing the subdomains and determinations/ratings of evaluation.

Classified as ECDC NORMAL

Figure S1. Random-effects meta-analysis of respiratory syncytial virus (RSV) estimates (62 studies)

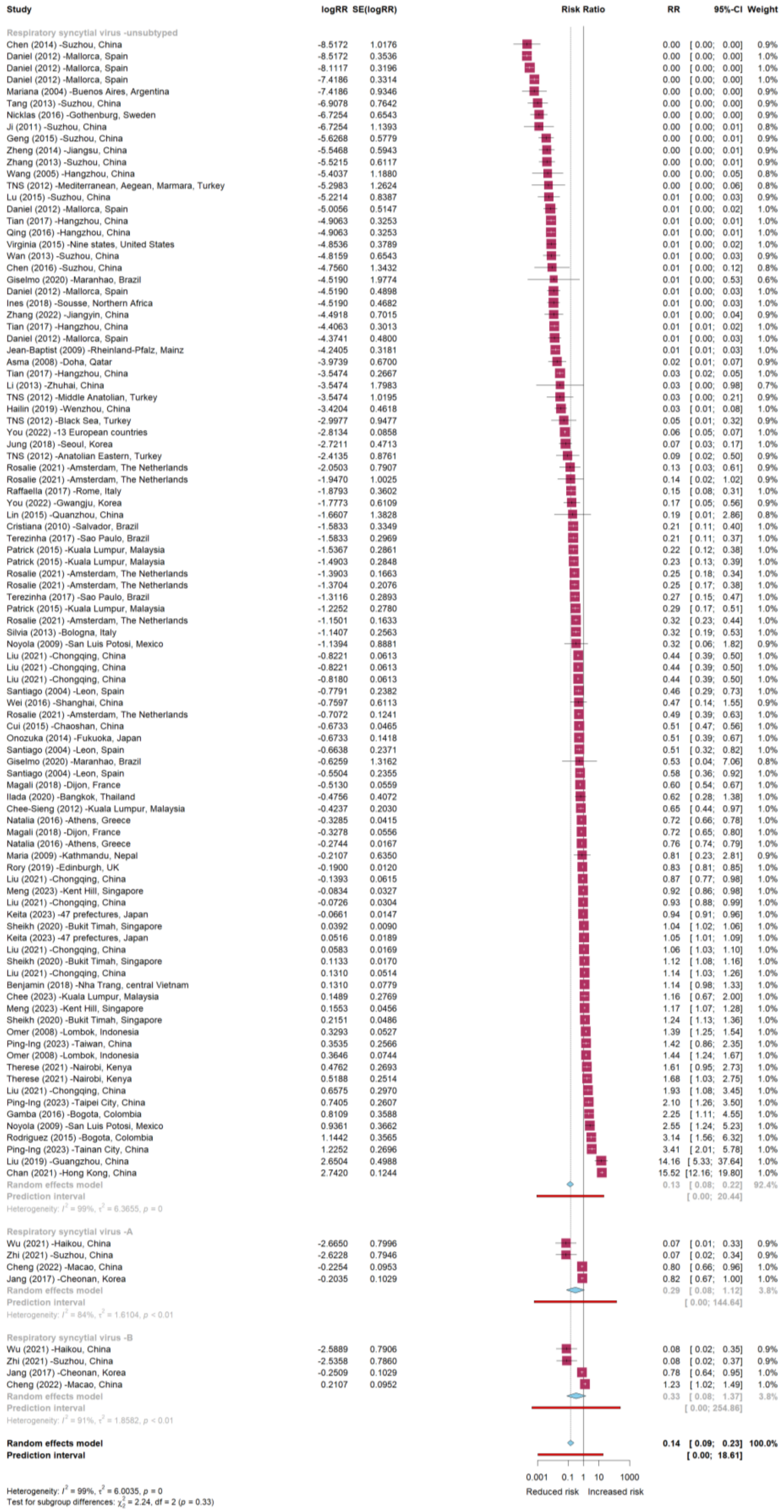

Classified as ECDC NORMAL

Figure S1-1. Subgroup analysis of respiratory syncytial virus (RSV) meta-analysis by Köppen-Geiger climate

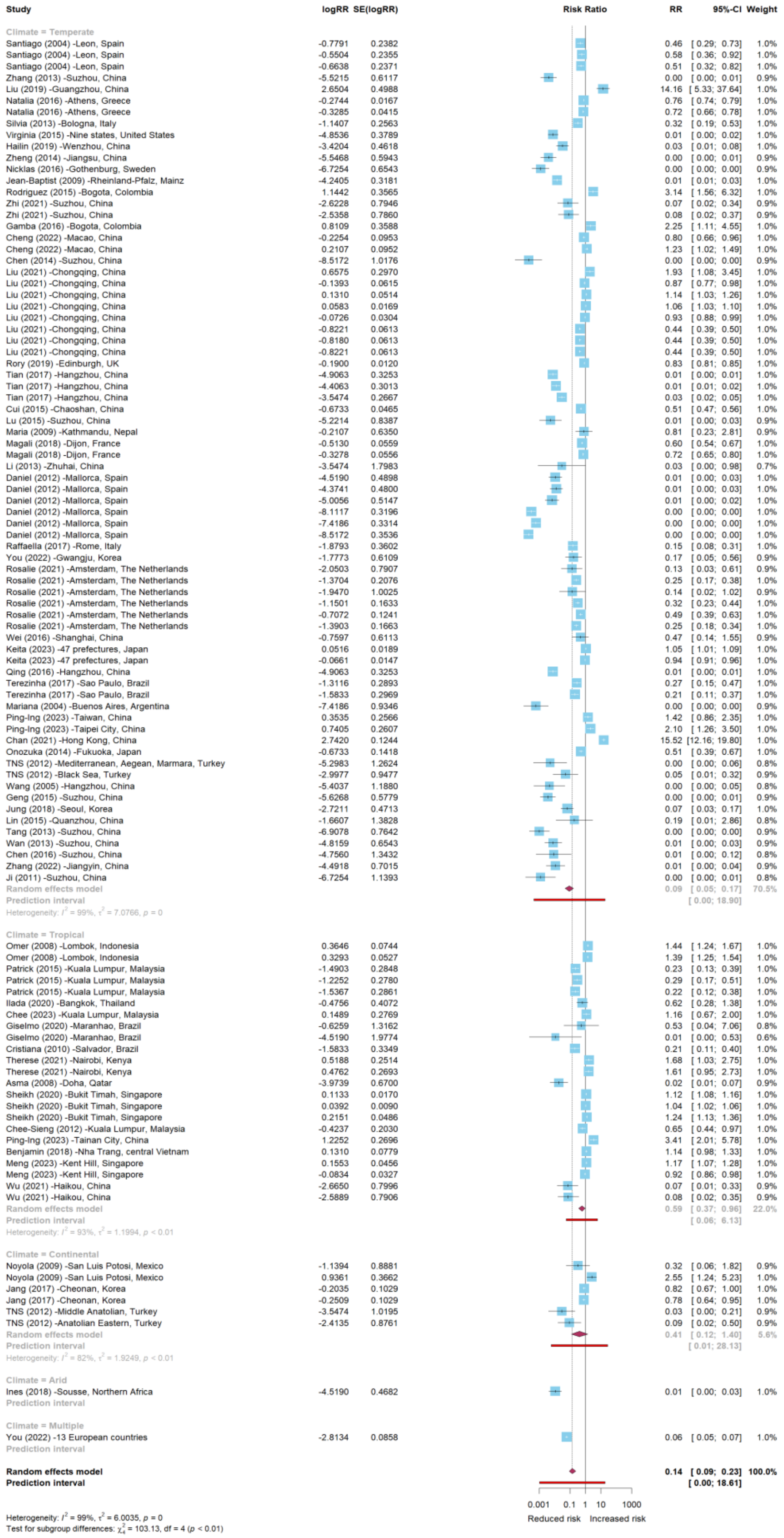

Classified as ECDC NORMAL

Figure S1-2. Subgroup analysis of respiratory syncytial virus (RSV) meta-analysis by World Bank income category

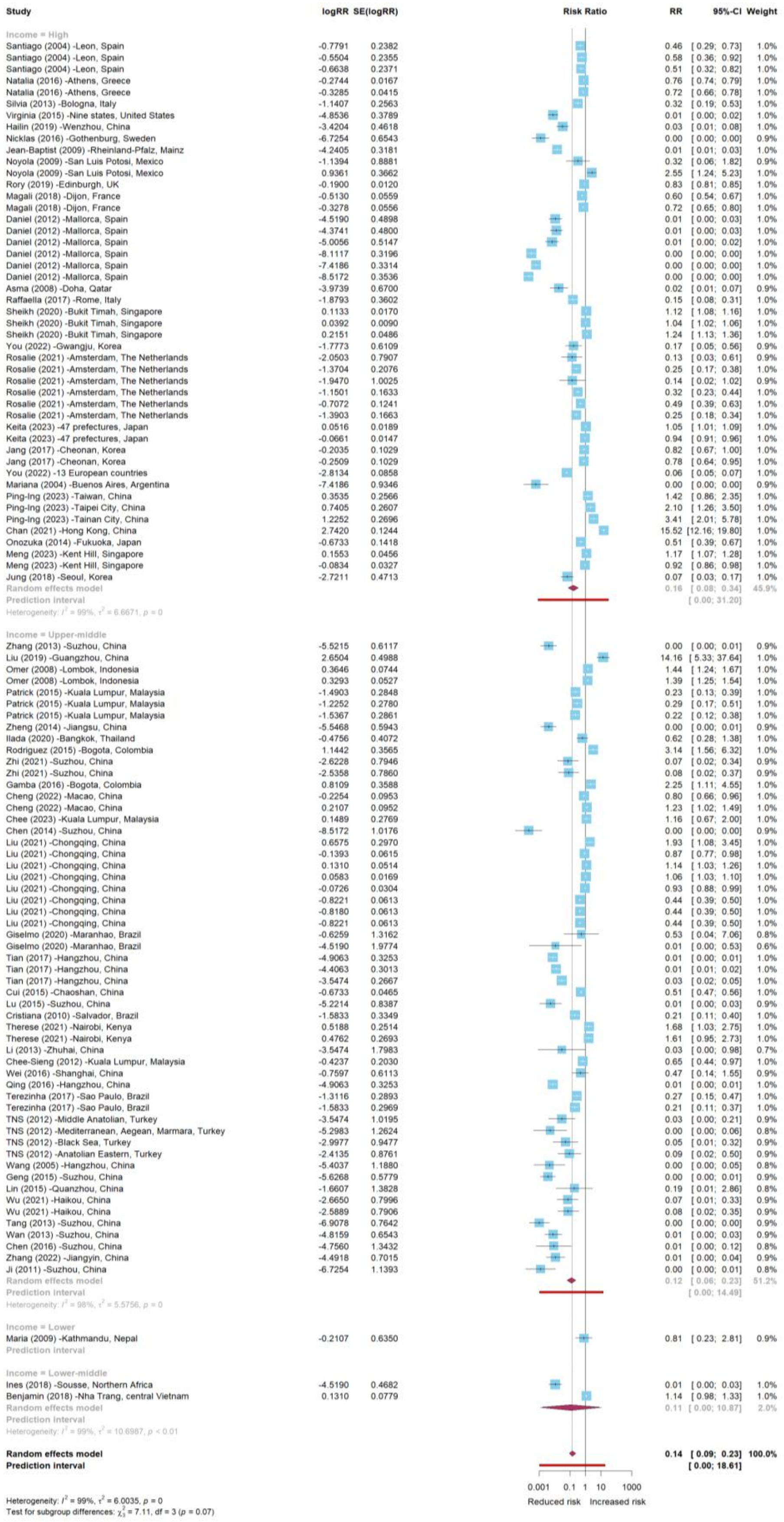

Classified as ECDC NORMAL

Figure S1-3. Subgroup analysis of respiratory syncytial virus (RSV) meta-analysis by temporal resolution

Study	logRR	SE(logRR)	RR	95%-CI	Weight
**Resolution = Weekly**					
Santiago (2004) -Leon, Spain	-0.7791	0.2382	0.46	[0.29; 0.73]	1.0%
Santiago (2004) -Leon, Spain	-0.5504	0.2355	0.58	[0.36; 0.92]	1.0%
Santiago (2004) -Leon, Spain	-0.6638	0.2371	0.51	[0.32; 0.82]	1.0%
Silvia (2013) -Bologna, Italy	-1.1407	0.2563	0.32	[0.19; 0.53]	1.0%
Nicklas (2016) -Gothenburg, Sweden	-6.7254	0.6543	0.00	[0.00; 0.00]	0.9%
Jean-Baptist (2009) -Rheinland-Pfalz, Mainz	-4.2405	0.3181	0.01	[0.01; 0.03]	1.0%
Noyola (2009) -San Luis Potosi, Mexico	-1.1394	0.8881	0.32	[0.06; 1.82]	0.9%
Noyola (2009) -San Luis Potosi, Mexico	0.9361	0.3662	2.55	[1.24; 5.23]	1.0%
Magali (2018) -Dijon, France	-0.5130	0.0559	0.60	[0.54; 0.67]	1.0%
Magali (2018) -Dijon, France	-0.3278	0.0556	0.72	[0.65; 0.80]	1.0%
Daniel (2012) -Mallorca, Spain	-8.1117	0.3196	0.00	[0.00; 0.00]	1.0%
Daniel (2012) -Mallorca, Spain	-7.4186	0.3314	0.00	[0.00; 0.00]	1.0%
Daniel (2012) -Mallorca, Spain	-8.5172	0.3536	0.00	[0.00; 0.00]	1.0%
Rosalie (2021) -Amsterdam, The Netherlands	-1.3704	0.2076	0.25	[0.17; 0.38]	1.0%
Rosalie (2021) -Amsterdam, The Netherlands	-1.1501	0.1633	0.32	[0.23; 0.44]	1.0%
Rosalie (2021) -Amsterdam, The Netherlands	-1.3903	0.1663	0.25	[0.18; 0.34]	1.0%
Keita (2023) -47 prefectures, Japan	0.0516	0.0189	1.05	[1.01; 1.09]	1.0%
Keita (2023) -47 prefectures, Japan	-0.0661	0.0147	0.94	[0.91; 0.96]	1.0%
You (2022) -13 European countries	-2.8134	0.0858	0.06	[0.05; 0.07]	1.0%
Onozuka (2014) -Fukuoka, Japan	-0.6733	0.1418	0.51	[0.39; 0.67]	1.0%
Random effects model			0.10	[0.03; 0.36]	19.7%
Prediction interval				[0.00; 54.59]	
Heterogeneity: $I^2 = 99\%$, $\tau^2 = 8.6074$, $p = 0$					
**Resolution = Monthly**					
Zhang (2013) -Suzhou, China	-5.5215	0.6117	0.00	[0.00; 0.01]	0.9%
Liu (2019) -Guangzhou, China	2.6504	0.4988	14.16	[5.33; 37.64]	1.0%
Natalia (2016) -Athens, Greece	-0.2744	0.0167	0.76	[0.74; 0.79]	1.0%
Natalia (2016) -Athens, Greece	-0.3285	0.0415	0.72	[0.66; 0.78]	1.0%
Virginia (2015) -Nine states, United States	-4.8536	0.3789	0.01	[0.00; 0.02]	1.0%
Patrick (2015) -Kuala Lumpur, Malaysia	-1.4903	0.2848	0.23	[0.13; 0.39]	1.0%
Patrick (2015) -Kuala Lumpur, Malaysia	-1.2252	0.2780	0.29	[0.17; 0.51]	1.0%
Patrick (2015) -Kuala Lumpur, Malaysia	-1.5367	0.2861	0.22	[0.12; 0.38]	1.0%
Hailin (2019) -Wenzhou, China	-3.4204	0.4618	0.03	[0.01; 0.08]	1.0%
Zheng (2014) -Jiangsu, China	-5.5468	0.5943	0.00	[0.00; 0.01]	0.9%
Ilada (2020) -Bangkok, Thailand	-0.4756	0.4072	0.62	[0.28; 1.38]	1.0%
Rodriguez (2015) -Bogota, Colombia	1.1442	0.3565	3.14	[1.56; 6.32]	1.0%
Zhi (2021) -Suzhou, China	-2.6228	0.7946	0.07	[0.02; 0.34]	0.9%
Zhi (2021) -Suzhou, China	-2.5358	0.7860	0.08	[0.02; 0.37]	0.9%
Gamba (2016) -Bogota, Colombia	0.8109	0.3588	2.25	[1.11; 4.55]	1.0%
Chee (2023) -Kuala Lumpur, Malaysia	0.1489	0.2769	1.16	[0.67; 2.00]	1.0%
Chen (2014) -Suzhou, China	-8.5172	1.0176	0.00	[0.00; 0.00]	0.9%
Giselmo (2020) -Maranhao, Brazil	-0.6259	1.3162	0.53	[0.04; 7.06]	0.8%
Giselmo (2020) -Maranhao, Brazil	-4.5190	1.9774	0.01	[0.00; 0.53]	0.6%
Rory (2019) -Edinburgh, UK	-0.1900	0.0120	0.83	[0.81; 0.85]	1.0%
Lu (2015) -Suzhou, China	-5.2214	0.8387	0.01	[0.00; 0.03]	0.9%
Cristiana (2010) -Salvador, Brazil	-1.5833	0.3349	0.21	[0.11; 0.40]	1.0%
Maria (2009) -Kathmandu, Nepal	-0.2107	0.6350	0.81	[0.23; 2.81]	0.9%
Therese (2021) -Nairobi, Kenya	0.5188	0.2514	1.68	[1.03; 2.75]	1.0%
Therese (2021) -Nairobi, Kenya	0.4762	0.2693	1.61	[0.95; 2.73]	1.0%
Li (2013) -Zhuhai, China	-3.5474	1.7983	0.03	[0.00; 0.98]	0.7%
Daniel (2012) -Mallorca, Spain	-4.5190	0.4898	0.01	[0.00; 0.03]	1.0%
Daniel (2012) -Mallorca, Spain	-4.3741	0.4800	0.01	[0.00; 0.03]	1.0%
Daniel (2012) -Mallorca, Spain	-5.0056	0.5147	0.01	[0.00; 0.02]	1.0%
Asma (2008) -Doha, Qatar	-3.9739	0.6700	0.02	[0.01; 0.07]	0.9%
Raffaella (2017) -Rome, Italy	-1.8793	0.3602	0.15	[0.08; 0.31]	1.0%
Ines (2018) -Sousse, Northern Africa	-4.5190	0.4682	0.01	[0.00; 0.03]	1.0%
You (2022) -Gwangju, Korea	-1.7773	0.6109	0.17	[0.05; 0.56]	0.9%
Chee-Sieng (2012) -Kuala Lumpur, Malaysia	-0.4237	0.2030	0.65	[0.44; 0.97]	1.0%
Rosalie (2021) -Amsterdam, The Netherlands	-2.0503	0.7907	0.13	[0.03; 0.61]	0.9%
Rosalie (2021) -Amsterdam, The Netherlands	-1.9470	1.0025	0.14	[0.02; 1.02]	0.9%
Rosalie (2021) -Amsterdam, The Netherlands	-0.7072	0.1241	0.49	[0.39; 0.63]	1.0%
Wei (2016) -Shanghai, China	-0.7597	0.6113	0.47	[0.14; 1.55]	0.9%
Jang (2017) -Cheonan, Korea	-0.2035	0.1029	0.82	[0.67; 1.00]	1.0%
Jang (2017) -Cheonan, Korea	-0.2509	0.1029	0.78	[0.64; 0.95]	1.0%
Terezinha (2017) -Sao Paulo, Brazil	-1.3116	0.2893	0.27	[0.15; 0.47]	1.0%
Terezinha (2017) -Sao Paulo, Brazil	-1.5833	0.2969	0.21	[0.11; 0.37]	1.0%
Mariana (2004) -Buenos Aires, Argentina	-7.4186	0.9346	0.00	[0.00; 0.00]	0.9%
Ping-Ing (2023) -Taiwan, China	0.3535	0.2566	1.42	[0.86; 2.35]	1.0%
Ping-Ing (2023) -Taipei City, China	0.7405	0.2607	2.10	[1.26; 3.50]	1.0%
Ping-Ing (2023) -Tainan City, China	1.2252	0.2696	3.41	[2.01; 5.78]	1.0%
Chan (2021) -Hong Kong, China	2.7420	0.1244	15.52	[12.16; 19.80]	1.0%
Benjamin (2018) -Nha Trang, central Vietnam	0.1310	0.0779	1.14	[0.98; 1.33]	1.0%
TNS (2012) -Middle Anatolian, Turkey	-3.5474	1.0195	0.03	[0.00; 0.21]	0.9%
TNS (2012) -Mediterranean, Aegean, Marmara, Turkey	-5.2983	1.2624	0.00	[0.00; 0.06]	0.8%
TNS (2012) -Black Sea, Turkey	-2.9977	0.9477	0.05	[0.01; 0.32]	0.9%
TNS (2012) -Anatolian Eastern, Turkey	-2.4135	0.8761	0.09	[0.02; 0.50]	0.9%
Wang (2005) -Hangzhou, China	-5.4037	1.1880	0.00	[0.00; 0.05]	0.8%
Geng (2015) -Suzhou, China	-5.6268	0.5779	0.00	[0.00; 0.01]	0.9%
Jung (2018) -Seoul, Korea	-2.7211	0.4713	0.07	[0.03; 0.17]	1.0%
Lin (2015) -Quanzhou, China	-1.6607	1.3828	0.19	[0.01; 2.86]	0.8%
Wu (2021) -Haikou, China	-2.6650	0.7996	0.07	[0.01; 0.33]	0.9%
Wu (2021) -Haikou, China	-2.5889	0.7906	0.08	[0.02; 0.35]	0.9%
Tang (2013) -Suzhou, China	-6.9078	0.7642	0.00	[0.00; 0.00]	0.9%
Wan (2013) -Suzhou, China	-4.8159	0.6543	0.01	[0.00; 0.03]	0.9%
Chen (2016) -Suzhou, China	-4.7560	1.3432	0.01	[0.00; 0.12]	0.8%
Zhang (2022) -Jiangyin, China	-4.4918	0.7015	0.01	[0.00; 0.04]	0.9%
Ji (2011) -Suzhou, China	-6.7254	1.1393	0.00	[0.00; 0.01]	0.8%
Random effects model			0.11	[0.06; 0.20]	58.4%
Prediction interval				[0.00; 14.11]	
Heterogeneity: $I^2 = 97\%$, $\tau^2 = 5.8991$, $p = 0$					
**Resolution = Daily**					
Omer (2008) -Lombok, Indonesia	0.3646	0.0744	1.44	[1.24; 1.67]	1.0%
Omer (2008) -Lombok, Indonesia	0.3293	0.0527	1.39	[1.25; 1.54]	1.0%
Cheng (2022) -Macao, China	-0.2254	0.0953	0.80	[0.66; 0.96]	1.0%
Cheng (2022) -Macao, China	0.2107	0.0952	1.23	[1.02; 1.49]	1.0%
Liu (2021) -Chongqing, China	0.6575	0.2970	1.93	[1.08; 3.45]	1.0%
Liu (2021) -Chongqing, China	-0.1393	0.0615	0.87	[0.77; 0.98]	1.0%
Liu (2021) -Chongqing, China	0.1310	0.0514	1.14	[1.03; 1.26]	1.0%
Liu (2021) -Chongqing, China	0.0583	0.0169	1.06	[1.03; 1.10]	1.0%
Liu (2021) -Chongqing, China	-0.0726	0.0304	0.93	[0.88; 0.99]	1.0%
Liu (2021) -Chongqing, China	-0.8221	0.0613	0.44	[0.39; 0.50]	1.0%
Liu (2021) -Chongqing, China	-0.8180	0.0613	0.44	[0.39; 0.50]	1.0%
Liu (2021) -Chongqing, China	-0.8221	0.0613	0.44	[0.39; 0.50]	1.0%
Tian (2017) -Hangzhou, China	-4.9063	0.3253	0.01	[0.00; 0.01]	1.0%
Tian (2017) -Hangzhou, China	-4.4063	0.3013	0.01	[0.01; 0.02]	1.0%
Tian (2017) -Hangzhou, China	-3.5474	0.2667	0.03	[0.02; 0.05]	1.0%
Cui (2015) -Chaoshan, China	-0.6733	0.0465	0.51	[0.47; 0.56]	1.0%
Sheikh (2020) -Bukit Timah, Singapore	0.1133	0.0170	1.12	[1.08; 1.16]	1.0%
Sheikh (2020) -Bukit Timah, Singapore	0.0392	0.0090	1.04	[1.02; 1.06]	1.0%
Sheikh (2020) -Bukit Timah, Singapore	0.2151	0.0486	1.24	[1.13; 1.36]	1.0%
Qing (2016) -Hangzhou, China	-4.9063	0.3253	0.01	[0.00; 0.01]	1.0%
Meng (2023) -Kent Hill, Singapore	0.1553	0.0456	1.17	[1.07; 1.28]	1.0%
Meng (2023) -Kent Hill, Singapore	-0.0834	0.0327	0.92	[0.86; 0.98]	1.0%
Random effects model			0.43	[0.20; 0.89]	21.9%
Prediction interval				[0.01; 17.64]	
Heterogeneity: $I^2 = 99\%$, $\tau^2 = 3.0465$, $p = 0$					
**Random effects model**			**0.14**	**[0.09; 0.23]**	**100.0%**
**Prediction interval**				**[0.00; 18.61]**	

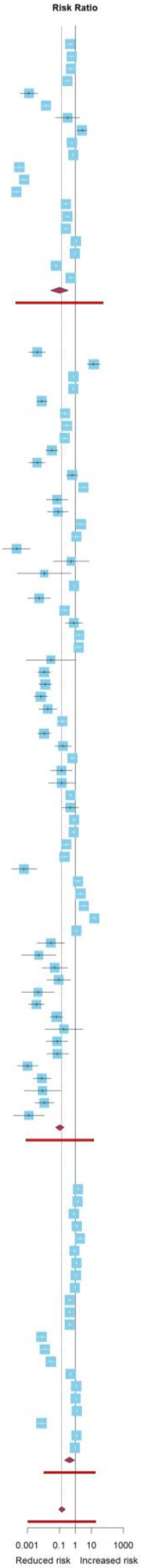

Heterogeneity: $I^2 = 99\%$, $\tau^2 = 6.0035$, $p = 0$
Test for subgroup differences: $\chi^2_2 = 8.99$, df = 2 ($p = 0.01$)

Classified as ECDC NORMAL

Figure S1-4. Subgroup analysis of respiratory syncytial virus (RSV) meta-analysis by exposure measure

Study	logRR	SE(logRR)	RR	95%-CI	Weight
**Temperature = Mean**					
Santiago (2004) -Leon, Spain	-0.7791	0.2382	0.46	[0.29; 0.73]	1.0%
Zhang (2013) -Suzhou, China	-5.5215	0.6117	0.00	[0.00; 0.01]	0.9%
Liu (2019) -Guangzhou, China	2.6504	0.4988	14.16	[5.33; 37.64]	1.0%
Natalia (2016) -Athens, Greece	-0.2744	0.0167	0.76	[0.74; 0.79]	1.0%
Natalia (2016) -Athens, Greece	-0.3285	0.0415	0.72	[0.66; 0.78]	1.0%
Omer (2008) -Lombok, Indonesia	0.3646	0.0744	1.44	[1.24; 1.67]	1.0%
Omer (2008) -Lombok, Indonesia	0.3293	0.0527	1.39	[1.25; 1.54]	1.0%
Patrick (2015) -Kuala Lumpur, Malaysia	-1.4903	0.2848	0.23	[0.13; 0.39]	1.0%
Patrick (2015) -Kuala Lumpur, Malaysia	-1.2252	0.2780	0.29	[0.17; 0.51]	1.0%
Patrick (2015) -Kuala Lumpur, Malaysia	-1.5367	0.2861	0.22	[0.12; 0.38]	1.0%
Hailin (2019) -Wenzhou, China	-3.4204	0.4618	0.03	[0.01; 0.08]	1.0%
Zheng (2014) -Jiangsu, China	-5.5468	0.5943	0.00	[0.00; 0.01]	0.9%
Ilada (2020) -Bangkok, Thailand	-0.4756	0.4072	0.62	[0.28; 1.38]	1.0%
Nicklas (2016) -Gothenburg, Sweden	-6.7254	0.6543	0.00	[0.00; 0.00]	0.9%
Jean-Baptist (2009) -Rheinland-Pfalz, Mainz	-4.2405	0.3181	0.01	[0.01; 0.03]	1.0%
Noyola (2009) -San Luis Potosi, Mexico	-1.1394	0.8881	0.32	[0.06; 1.82]	0.9%
Rodriguez (2015) -Bogota, Colombia	1.1442	0.3565	3.14	[1.56; 6.32]	1.0%
Zhi (2021) -Suzhou, China	-2.6228	0.7946	0.07	[0.02; 0.34]	0.9%
Zhi (2021) -Suzhou, China	-2.5358	0.7860	0.08	[0.02; 0.37]	0.9%
Gamba (2016) -Bogota, Colombia	0.8109	0.3588	2.25	[1.11; 4.55]	1.0%
Cheng (2022) -Macao, China	-0.2254	0.0953	0.80	[0.66; 0.96]	1.0%
Cheng (2022) -Macao, China	0.2107	0.0952	1.23	[1.02; 1.49]	1.0%
Chee (2023) -Kuala Lumpur, Malaysia	0.1489	0.2769	1.16	[0.67; 2.00]	1.0%
Chen (2014) -Suzhou, China	-8.5172	1.0176	0.00	[0.00; 0.00]	0.9%
Liu (2021) -Chongqing, China	-0.8221	0.0613	0.44	[0.39; 0.50]	1.0%
Rory (2019) -Edinburgh, UK	-0.1900	0.0120	0.83	[0.81; 0.85]	1.0%
Tian (2017) -Hangzhou, China	-4.4063	0.3013	0.01	[0.01; 0.02]	1.0%
Cui (2015) -Chaoshan, China	-0.6733	0.0465	0.51	[0.47; 0.56]	1.0%
Lu (2015) -Suzhou, China	-5.2214	0.8387	0.01	[0.00; 0.03]	0.9%
Cristiana (2010) -Salvador, Brazil	-1.5833	0.3349	0.21	[0.11; 0.40]	1.0%
Maria (2009) -Kathmandu, Nepal	-0.2107	0.6350	0.81	[0.23; 2.81]	0.9%
Magali (2018) -Dijon, France	-0.5130	0.0559	0.60	[0.54; 0.67]	1.0%
Magali (2018) -Dijon, France	-0.3278	0.0556	0.72	[0.65; 0.80]	1.0%
Therese (2021) -Nairobi, Kenya	0.5188	0.2514	1.68	[1.03; 2.75]	1.0%
Therese (2021) -Nairobi, Kenya	0.4762	0.2693	1.61	[0.95; 2.73]	1.0%
Li (2013) -Zhuhai, China	-3.5474	1.7983	0.03	[0.00; 0.98]	0.7%
Daniel (2012) -Mallorca, Spain	-4.5190	0.4898	0.01	[0.00; 0.03]	1.0%
Daniel (2012) -Mallorca, Spain	-8.1117	0.3196	0.00	[0.00; 0.00]	1.0%
Asma (2008) -Doha, Qatar	-3.9739	0.6700	0.02	[0.01; 0.07]	0.9%
Raffaella (2017) -Rome, Italy	-1.8793	0.3602	0.15	[0.08; 0.31]	1.0%
Sheikh (2020) -Bukit Timah, Singapore	0.1133	0.0170	1.12	[1.08; 1.16]	1.0%
Ines (2018) -Sousse, Northern Africa	-4.5190	0.4682	0.01	[0.00; 0.03]	1.0%
You (2022) -Gwangju, Korea	-1.7773	0.6109	0.17	[0.05; 0.56]	0.9%
Chee-Sieng (2012) -Kuala Lumpur, Malaysia	-0.4237	0.2030	0.65	[0.44; 0.97]	1.0%
Rosalie (2021) -Amsterdam, The Netherlands	-2.0503	0.7907	0.13	[0.03; 0.61]	0.9%
Rosalie (2021) -Amsterdam, The Netherlands	-1.3704	0.2076	0.25	[0.17; 0.38]	1.0%
Wei (2016) -Shanghai, China	-0.7597	0.6113	0.47	[0.14; 1.55]	0.9%
Keita (2023) -47 prefectures, Japan	0.0516	0.0189	1.05	[1.01; 1.09]	1.0%
Keita (2023) -47 prefectures, Japan	-0.0661	0.0147	0.94	[0.91; 0.96]	1.0%
Qing (2016) -Hangzhou, China	-4.9063	0.3253	0.01	[0.00; 0.01]	1.0%
Jang (2017) -Cheonan, Korea	-0.2035	0.1029	0.82	[0.67; 1.00]	1.0%
Jang (2017) -Cheonan, Korea	-0.2509	0.1029	0.78	[0.64; 0.95]	1.0%
You (2022) -13 European countries	-2.8134	0.0858	0.06	[0.05; 0.07]	1.0%
Mariana (2004) -Buenos Aires, Argentina	-7.4186	0.9346	0.00	[0.00; 0.00]	0.9%
Ping-Ing (2023) -Taiwan, China	0.3535	0.2566	1.42	[0.86; 2.35]	1.0%
Ping-Ing (2023) -Taipei City, China	0.7405	0.2607	2.10	[1.26; 3.50]	1.0%
Ping-Ing (2023) -Tainan City, China	1.2252	0.2696	3.41	[2.01; 5.78]	1.0%
Chan (2021) -Hong Kong, China	2.7420	0.1244	15.52	[12.16; 19.80]	1.0%
Onozuka (2014) -Fukuoka, Japan	-0.6733	0.1418	0.51	[0.39; 0.67]	1.0%
Benjamin (2018) -Nha Trang, central Vietnam	0.1310	0.0779	1.14	[0.98; 1.33]	1.0%
TNS (2012) -Middle Anatolian, Turkey	-3.5474	1.0195	0.03	[0.00; 0.21]	0.9%
TNS (2012) -Mediterranean, Aegean, Marmara, Turkey	-5.2983	1.2624	0.00	[0.00; 0.06]	0.8%
TNS (2012) -Black Sea, Turkey	-2.9977	0.9477	0.05	[0.01; 0.32]	0.9%
TNS (2012) -Anatolian Eastern, Turkey	-2.4135	0.8761	0.09	[0.02; 0.50]	0.9%
Wang (2005) -Hangzhou, China	-5.4037	1.1880	0.00	[0.00; 0.05]	0.8%
Geng (2015) -Suzhou, China	-5.6268	0.5779	0.00	[0.00; 0.01]	0.9%
Jung (2018) -Seoul, Korea	-2.7211	0.4713	0.07	[0.03; 0.17]	1.0%
Lin (2015) -Quanzhou, China	-1.6607	1.3828	0.19	[0.01; 2.86]	0.8%
Wu (2021) -Haikou, China	-2.6650	0.7996	0.07	[0.01; 0.33]	0.9%
Wu (2021) -Haikou, China	-2.5889	0.7906	0.08	[0.02; 0.35]	0.9%
Tang (2013) -Suzhou, China	-6.9078	0.7642	0.00	[0.00; 0.00]	0.9%
Wan (2013) -Suzhou, China	-4.8159	0.6543	0.01	[0.00; 0.03]	0.9%
Chen (2016) -Suzhou, China	-4.7560	1.3432	0.01	[0.00; 0.12]	0.8%
Zhang (2022) -Jiangyin, China	-4.4918	0.7015	0.01	[0.00; 0.04]	0.9%
Ji (2011) -Suzhou, China	-6.7254	1.1393	0.00	[0.00; 0.01]	0.8%
Random effects model			0.13	[0.07; 0.23]	71.0%
Prediction interval				[0.00; 18.97]	
Heterogeneity: $I^2 = 99\%$, $\tau^2 = 6.1799$, $p = 0$					
**Temperature = Min**					
Santiago (2004) -Leon, Spain	-0.5504	0.2355	0.58	[0.36; 0.92]	1.0%
Silvia (2013) -Bologna, Italy	-1.1407	0.2563	0.32	[0.19; 0.53]	1.0%
Virginia (2015) -Nine states, United States	-4.8536	0.3789	0.01	[0.00; 0.02]	1.0%
Liu (2021) -Chongqing, China	0.6575	0.2970	1.93	[1.08; 3.45]	1.0%
Liu (2021) -Chongqing, China	-0.1393	0.0615	0.87	[0.77; 0.98]	1.0%
Liu (2021) -Chongqing, China	0.1310	0.0514	1.14	[1.03; 1.26]	1.0%
Liu (2021) -Chongqing, China	0.0583	0.0169	1.06	[1.03; 1.10]	1.0%
Liu (2021) -Chongqing, China	-0.0726	0.0304	0.93	[0.88; 0.99]	1.0%
Liu (2021) -Chongqing, China	-0.8180	0.0613	0.44	[0.39; 0.50]	1.0%
Giselmo (2020) -Maranhao, Brazil	-0.6259	1.3162	0.53	[0.04; 7.06]	0.8%
Tian (2017) -Hangzhou, China	-4.9063	0.3253	0.01	[0.00; 0.01]	1.0%
Daniel (2012) -Mallorca, Spain	-4.3741	0.4800	0.01	[0.00; 0.03]	1.0%
Daniel (2012) -Mallorca, Spain	-7.4186	0.3314	0.00	[0.00; 0.00]	1.0%
Sheikh (2020) -Bukit Timah, Singapore	0.0392	0.0090	1.04	[1.02; 1.06]	1.0%
Rosalie (2021) -Amsterdam, The Netherlands	-1.9470	1.0025	0.14	[0.02; 1.02]	0.9%
Rosalie (2021) -Amsterdam, The Netherlands	-1.1501	0.1633	0.32	[0.23; 0.44]	1.0%
Terezinha (2017) -Sao Paulo, Brazil	-1.3116	0.2893	0.27	[0.15; 0.47]	1.0%
Random effects model			0.19	[0.06; 0.57]	16.5%
Prediction interval				[0.00; 28.50]	
Heterogeneity: $I^2 = 99\%$, $\tau^2 = 5.2196$, $p < 0.01$					
**Temperature = Max**					
Santiago (2004) -Leon, Spain	-0.6638	0.2371	0.51	[0.32; 0.82]	1.0%
Noyola (2009) -San Luis Potosi, Mexico	0.9361	0.3662	2.55	[1.24; 5.23]	1.0%
Liu (2021) -Chongqing, China	-0.8221	0.0613	0.44	[0.39; 0.50]	1.0%
Giselmo (2020) -Maranhao, Brazil	-4.5190	1.9774	0.01	[0.00; 0.53]	0.6%
Tian (2017) -Hangzhou, China	-3.5474	0.2667	0.03	[0.02; 0.05]	1.0%
Daniel (2012) -Mallorca, Spain	-5.0056	0.5147	0.01	[0.00; 0.02]	1.0%
Daniel (2012) -Mallorca, Spain	-8.5172	0.3536	0.00	[0.00; 0.00]	1.0%
Sheikh (2020) -Bukit Timah, Singapore	0.2151	0.0486	1.24	[1.13; 1.36]	1.0%
Rosalie (2021) -Amsterdam, The Netherlands	-0.7072	0.1241	0.49	[0.39; 0.63]	1.0%
Rosalie (2021) -Amsterdam, The Netherlands	-1.3903	0.1663	0.25	[0.18; 0.34]	1.0%
Terezinha (2017) -Sao Paulo, Brazil	-1.5833	0.2969	0.21	[0.11; 0.37]	1.0%
Meng (2023) -Kent Hill, Singapore	0.1553	0.0456	1.17	[1.07; 1.28]	1.0%
Meng (2023) -Kent Hill, Singapore	-0.0834	0.0327	0.92	[0.86; 0.98]	1.0%
Random effects model			0.15	[0.04; 0.66]	12.5%
Prediction interval				[0.00; 64.75]	
Heterogeneity: $I^2 = 99\%$, $\tau^2 = 6.9935$, $p < 0.01$					
**Random effects model**			**0.14**	**[0.09; 0.23]**	**100.0%**
**Prediction interval**				**[0.00; 18.61]**	

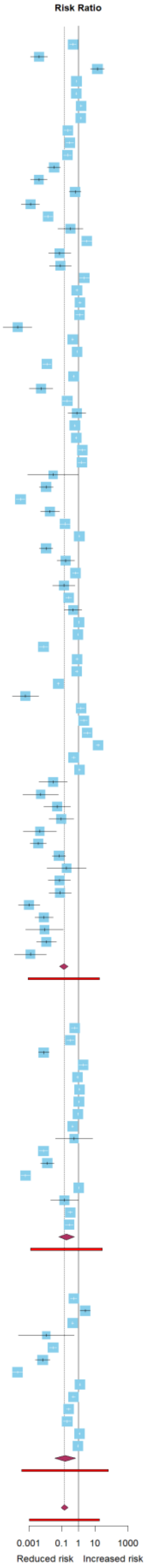

Heterogeneity: $I^2 = 99\%$, $\tau^2 = 6.0035$, $p = 0$
Test for subgroup differences: $\chi^2_2 = 0.37$, df = 2 ($p = 0.83$)

Classified as ECDC NORMAL

Figure S1-5. Subgroup analysis of respiratory syncytial virus (RSV) meta-analysis by modelling approach

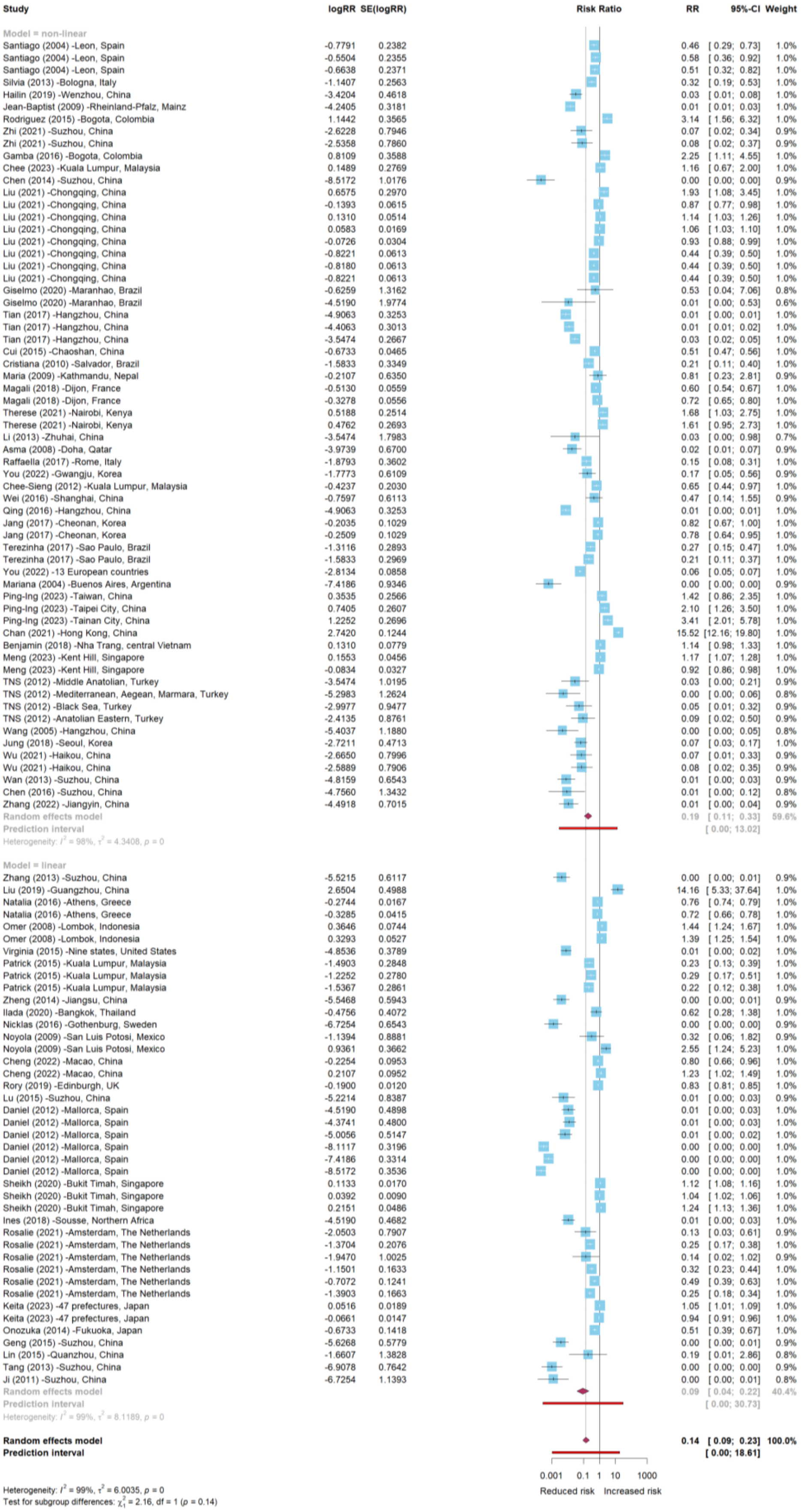

Classified as ECDC NORMAL

Figure S1-6. Subgroup analysis of respiratory syncytial virus (RSV) meta-analysis by lag type

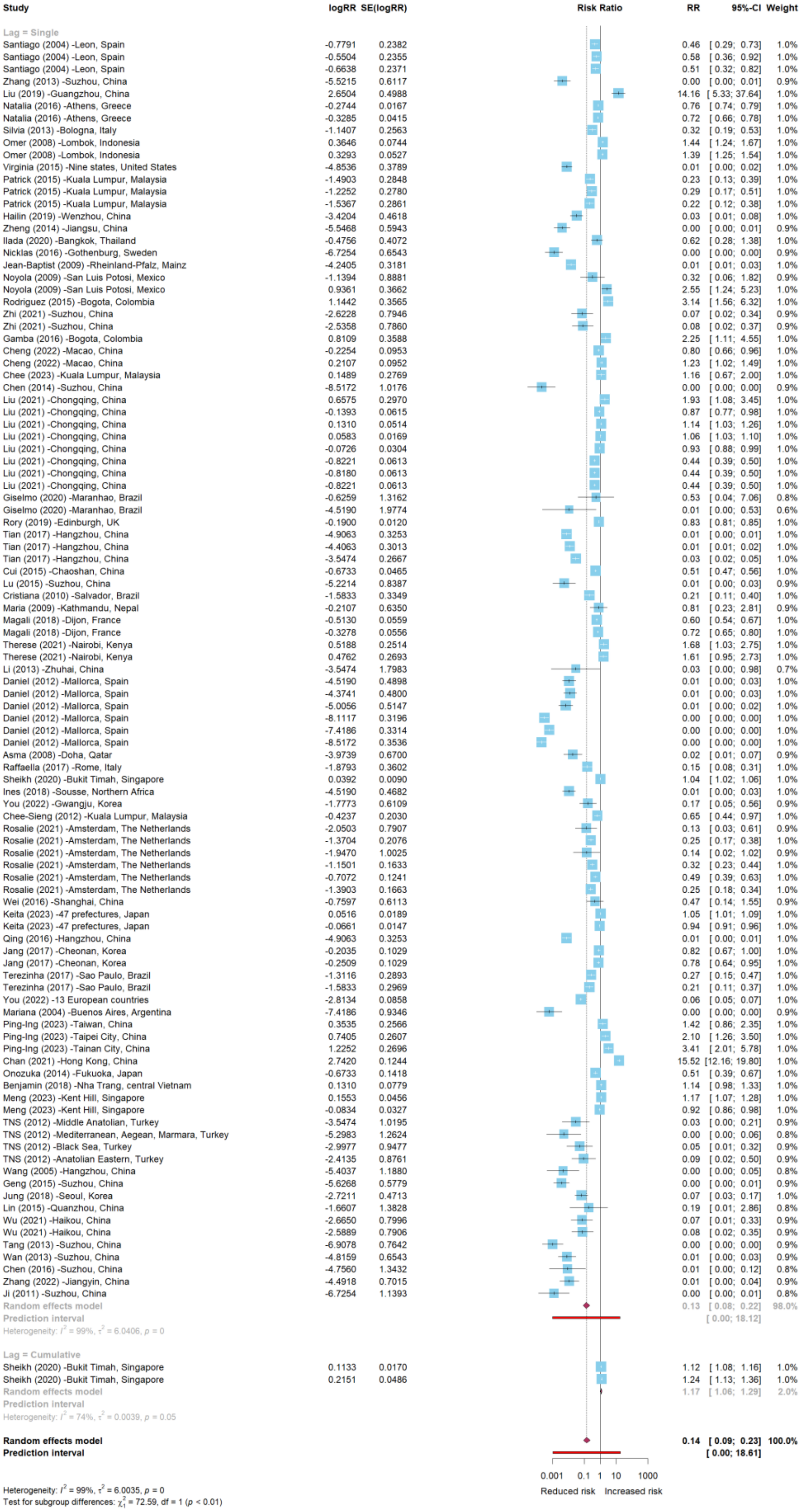

Classified as ECDC NORMAL

Figure S1-7. Leave-one-out analysis of respiratory syncytial virus (RSV) meta-analysis

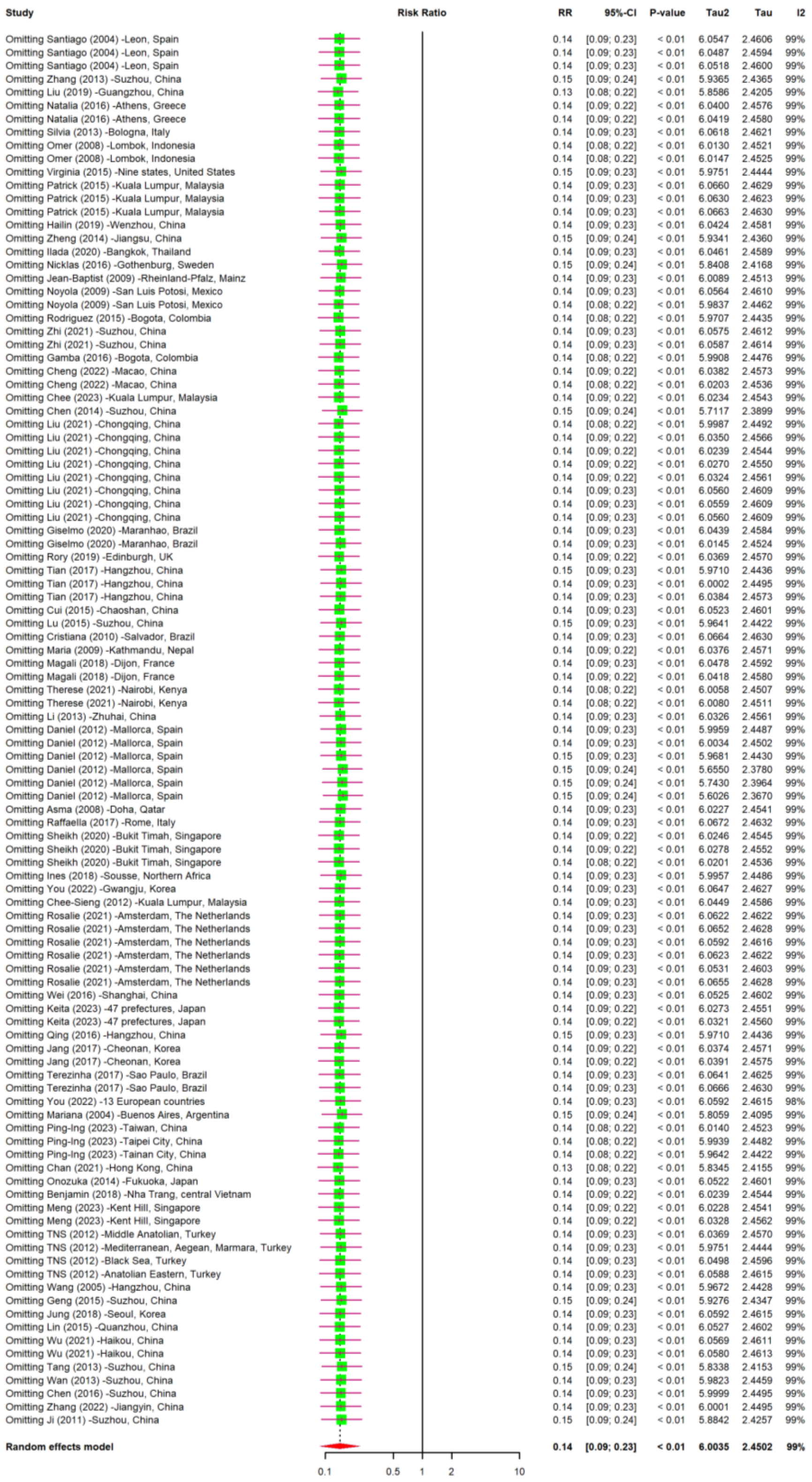

Figure S1-8. Funnel plot of respiratory syncytial virus (RSV) estimates

Classified as ECDC NORMAL

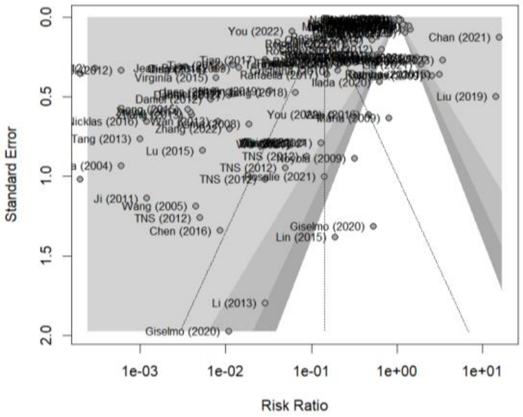

Linear regression test of funnel plot asymmetry

Egger’s test result: t = -6.36, df = 103, p-value < 0.0001

Sample estimates:

bias	se.bias	intercept	se.intercept
-5.1657	0.8258	0.0354	0.0397

Figure S1-9. Trim and fill of respiratory syncytial virus (RSV) meta-analysis

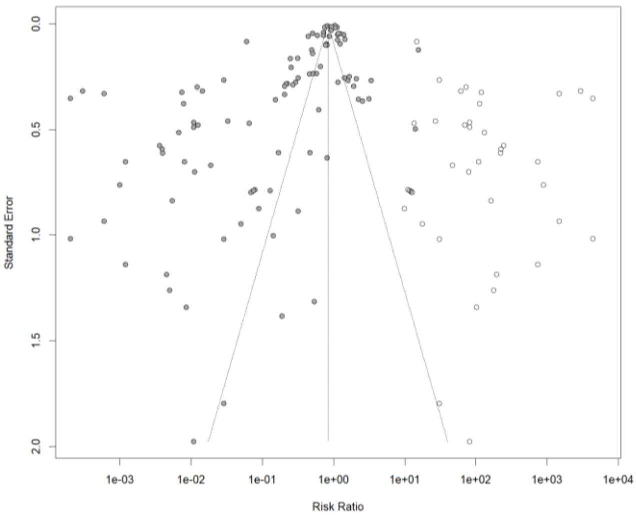

Number of studies: k = 145 (with 40 added studies)

Adjusted estimates: RR= 0.8329 [0.4510; 1.5382], Z= -0.58, *P*= 0.5591

Quantifying heterogeneity: tau^2 = 13.7945 [11.1059; 17.9865]; tau = 3.7141 [3.3326; 4.2411],

I^2 = 98.9% [98.8%; 99.0%]; H = 9.50 [9.21; 9.80]

Classified as ECDC NORMAL

Figure S2. Random-effects meta-analysis of influenza virus (IV) estimates (27 studies)

Study	logRR	SE(logRR)	RR	95%-CI	Weight
Influenza virus A					
Mariana (2004) -Buenos Aires, Argentina	-6.3771	0.9767	0.00	[0.00; 0.01]	1.1%
Li (2020) -Shanghai, China	-2.8665	0.4635	0.06	[0.02; 0.14]	1.4%
Li (2020) -Shanghai, China	-2.8665	0.4635	0.06	[0.02; 0.14]	1.4%
Li (2020) -Shanghai, China	-2.7920	0.4580	0.06	[0.02; 0.15]	1.4%
Chen (2014) -Suzhou, China	-2.3279	0.9684	0.10	[0.01; 0.65]	1.1%
Jean-Baptist (2009) -Rheinland-Pfalz, Mainz	-2.2672	0.2439	0.10	[0.06; 0.17]	1.5%
Zheng (2014) -Jiangsu, China	-1.2040	0.3368	0.30	[0.16; 0.58]	1.5%
Adriana (2018) -Toronto, Canada	-1.0581	0.2154	0.35	[0.23; 0.53]	1.5%
Zhang (2022) -Jiangyin, China	-1.0458	0.4587	0.35	[0.14; 0.86]	1.4%
Chen (2016) -Suzhou, China	-0.6259	0.8280	0.53	[0.11; 2.71]	1.2%
Ji (2011) -Suzhou, China	-0.5504	0.5547	0.58	[0.19; 1.71]	1.4%
Cui (2015) -Chaoshan, China	-0.5108	0.0858	0.60	[0.51; 0.71]	1.5%
Gwladys (2017) -Yaounde, Cameroon	-0.2838	0.1929	0.75	[0.52; 1.10]	1.5%
Benjamin (2018) -Nha Trang, central Vietnam	-0.1625	0.0465	0.85	[0.78; 0.93]	1.5%
Rory (2019) -Edinburgh, UK	-0.1473	0.0112	0.86	[0.84; 0.88]	1.5%
Cheng (2022) -Macao, China	-0.1380	0.0951	0.87	[0.72; 1.05]	1.5%
Maria (2009) -Kathmandu, Nepal	-0.1234	0.6343	0.88	[0.25; 3.06]	1.3%
Adriana (2018) -Toronto, Canada	-0.0943	0.0084	0.91	[0.90; 0.93]	1.5%
Annabel (2023) -Bukit Timah, Singapore	-0.0523	0.0180	0.95	[0.92; 0.98]	1.5%
Lin (2015) -Quanzhou, China	0.1222	1.3027	1.13	[0.09; 14.52]	1.0%
Li (2020) -Shanghai, China	3.1001	0.3334	22.20	[11.55; 42.67]	1.5%
Random effects model			0.37	[0.18; 0.76]	29.3%
Prediction interval				[0.01; 12.10]	
Heterogeneity: $I^2 = 95\%$, $\tau^2 = 2.6400$, $p < 0.01$					
Influenza virus A/H1N1pdm09					
Cao (2010) -Beijing, China	-10.4143	0.7642	0.00	[0.00; 0.00]	1.3%
Wei (2019) -Jinan, China	-2.9584	0.3284	0.05	[0.03; 0.10]	1.5%
Pan (2019) -Panzhihua, China	-1.4494	0.3617	0.23	[0.12; 0.48]	1.4%
Li (2013) -Zhuhai, China	-0.6677	1.3186	0.51	[0.04; 6.80]	1.0%
Can (2021) -Northern China	-0.0726	0.0275	0.93	[0.88; 0.98]	1.5%
Can (2021) -China	-0.0619	0.0243	0.94	[0.90; 0.99]	1.5%
Can (2021) -Southern China	-0.0408	0.0291	0.96	[0.91; 1.02]	1.5%
Pan (2022) -Shenzhen, China	0.0908	0.0718	1.09	[0.95; 1.26]	1.5%
Pan (2022) -Shenzhen, China	0.0979	0.0719	1.10	[0.96; 1.27]	1.5%
Pan (2022) -Shenzhen, China	0.1053	0.0719	1.11	[0.97; 1.28]	1.5%
Random effects model			0.22	[0.03; 1.61]	14.2%
Prediction interval				[0.00; 461.07]	
Heterogeneity: $I^2 = 97\%$, $\tau^2 = 9.9374$, $p < 0.01$					
Influenza virus A/H3N2					
Wei (2019) -Jinan, China	-2.2201	0.2980	0.11	[0.06; 0.19]	1.5%
Adriana (2018) -Toronto, Canada	-1.5833	0.2257	0.21	[0.13; 0.32]	1.5%
Pan (2019) -Panzhihua, China	-0.3698	0.3375	0.69	[0.36; 1.34]	1.5%
Can (2021) -Northern China	-0.0408	0.0266	0.96	[0.91; 1.01]	1.5%
Can (2021) -China	-0.0305	0.0263	0.97	[0.92; 1.02]	1.5%
Can (2021) -Southern China	-0.0101	0.0282	0.99	[0.94; 1.05]	1.5%
Li (2013) -Zhuhai, China	2.6646	1.6075	14.36	[0.62; 335.42]	0.8%
Random effects model			0.59	[0.27; 1.28]	9.8%
Prediction interval				[0.04; 8.97]	
Heterogeneity: $I^2 = 94\%$, $\tau^2 = 0.9660$, $p < 0.01$					
Influenza virus B					
Nicklas (2016) -Gothenburg, Sweden	-6.1193	0.6078	0.00	[0.00; 0.01]	1.3%
Mariana (2004) -Buenos Aires, Argentina	-2.1507	0.5646	0.12	[0.04; 0.35]	1.4%
Li (2020) -Shanghai, China	-1.9317	0.4117	0.14	[0.06; 0.32]	1.4%
Li (2020) -Shanghai, China	-1.8793	0.4090	0.15	[0.07; 0.34]	1.4%
Adriana (2018) -Toronto, Canada	-1.8282	0.2317	0.16	[0.10; 0.25]	1.5%
Wei (2019) -Jinan, China	-1.8282	0.2845	0.16	[0.09; 0.28]	1.5%
Li (2020) -Shanghai, China	-1.7277	0.4025	0.18	[0.08; 0.39]	1.4%
Jean-Baptist (2009) -Rheinland-Pfalz, Mainz	-1.2680	0.2190	0.28	[0.18; 0.43]	1.5%
Zhang (2022) -Jiangyin, China	-1.2595	0.4665	0.28	[0.11; 0.71]	1.4%
Maria (2009) -Kathmandu, Nepal	-1.1829	0.6668	0.31	[0.08; 1.13]	1.3%
Chen (2016) -Suzhou, China	-0.7404	0.8329	0.48	[0.09; 2.44]	1.2%
Pan (2022) -Shenzhen, China	-0.6259	0.0729	0.53	[0.46; 0.62]	1.5%
Pan (2022) -Shenzhen, China	-0.6182	0.0728	0.54	[0.47; 0.62]	1.5%
Pan (2022) -Shenzhen, China	-0.5766	0.0727	0.56	[0.49; 0.65]	1.5%
Zheng (2014) -Jiangsu, China	-0.2801	0.3207	0.76	[0.40; 1.42]	1.5%
Gwladys (2017) -Yaounde, Cameroon	-0.1744	0.1926	0.84	[0.58; 1.23]	1.5%
Rory (2019) -Edinburgh, UK	-0.1393	0.0138	0.87	[0.85; 0.89]	1.5%
Adriana (2018) -Toronto, Canada	-0.0619	0.0081	0.94	[0.93; 0.96]	1.5%
Cheng (2022) -Macao, China	-0.0617	0.0951	0.94	[0.78; 1.13]	1.5%
Can (2021) -Northern China	-0.0408	0.0187	0.96	[0.93; 1.00]	1.5%
Can (2021) -China	-0.0305	0.0183	0.97	[0.94; 1.01]	1.5%
Can (2021) -Southern China	-0.0305	0.0211	0.97	[0.93; 1.01]	1.5%
Li (2013) -Zhuhai, China	0.0508	1.2972	1.05	[0.08; 13.37]	1.0%
Ji (2011) -Suzhou, China	0.4756	0.5532	1.61	[0.54; 4.76]	1.4%
Lin (2015) -Quanzhou, China	0.8879	1.3427	2.43	[0.17; 33.77]	0.9%
Li (2020) -Shanghai, China	3.3925	0.2460	29.74	[18.36; 48.16]	1.5%
Random effects model			0.47	[0.26; 0.85]	36.7%
Prediction interval				[0.02; 10.65]	
Heterogeneity: $I^2 = 96\%$, $\tau^2 = 2.2022$, $p < 0.01$					
Influenza virus B/Victoria					
Pan (2019) -Panzhihua, China	0.2764	0.3367	1.32	[0.68; 2.55]	1.5%
Prediction interval					
Influenza virus B/Yamagata					
Pan (2019) -Panzhihua, China	-0.4008	0.3378	0.67	[0.35; 1.30]	1.5%
Prediction interval					
Influenza virus -unsubtyped					
You (2022) -Gwangju, Korea	-3.6156	0.7767	0.03	[0.01; 0.12]	1.3%
Wei (2016) -Shanghai, China	-1.9421	0.6791	0.14	[0.04; 0.54]	1.3%
Adriana (2018) -Toronto, Canada	-1.1829	0.2175	0.31	[0.20; 0.47]	1.5%
Gwladys (2017) -Yaounde, Cameroon	-0.3425	0.1932	0.71	[0.49; 1.04]	1.5%
Adriana (2018) -Toronto, Canada	-0.0726	0.0055	0.93	[0.92; 0.94]	1.5%
Random effects model			0.28	[0.09; 0.88]	7.1%
Prediction interval				[0.00; 21.45]	
Heterogeneity: $I^2 = 93\%$, $\tau^2 = 1.5151$, $p < 0.01$					
**Random effects model**			**0.40**	**[0.27; 0.61]**	**100.0%**
**Prediction interval**				**[0.01; 12.69]**	

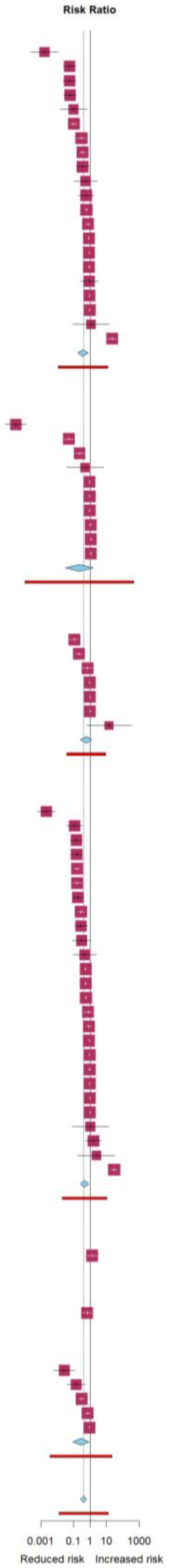

Heterogeneity: $I^2 = 96\%$, $\tau^2 = 2.9513$, $p < 0.01$
Test for subgroup differences: $\chi^2_6 = 10.55$, df = 6 ($p = 0.10$)

Classified as ECDC NORMAL

Figure S2-1. Subgroup analysis of influenza virus (IV) meta-analysis by Köppen-Geiger climate

Study	logRR	SE(logRR)	RR	95%-CI	Weight
Climate = Temperate					
Zheng (2014) -Jiangsu, China	-1.2040	0.3368	0.30	[0.16; 0.58]	1.5%
Jean-Baptist (2009) -Rheinland-Pfalz, Mainz	-2.2672	0.2439	0.10	[0.06; 0.17]	1.5%
Cheng (2022) -Macao, China	-0.1380	0.0951	0.87	[0.72; 1.05]	1.5%
Li (2020) -Shanghai, China	-2.8665	0.4635	0.06	[0.02; 0.14]	1.4%
Li (2020) -Shanghai, China	-2.7920	0.4580	0.06	[0.02; 0.15]	1.4%
Li (2020) -Shanghai, China	-2.8665	0.4635	0.06	[0.02; 0.14]	1.4%
Chen (2014) -Suzhou, China	-2.3279	0.9684	0.10	[0.01; 0.65]	1.1%
Rory (2019) -Edinburgh, UK	-0.1473	0.0112	0.86	[0.84; 0.88]	1.5%
Cui (2015) -Chaoshan, China	-0.5108	0.0858	0.60	[0.51; 0.71]	1.5%
Maria (2009) -Kathmandu, Nepal	-0.1234	0.6343	0.88	[0.25; 3.06]	1.3%
Li (2013) -Zhuhai, China	-0.6677	1.3186	0.51	[0.04; 6.80]	1.0%
Li (2013) -Zhuhai, China	2.6646	1.6075	14.36	[0.62; 335.42]	0.8%
Mariana (2004) -Buenos Aires, Argentina	-6.3771	0.9767	0.00	[0.00; 0.01]	1.1%
Pan (2022) -Shenzhen, China	0.1053	0.0719	1.11	[0.97; 1.28]	1.5%
Pan (2022) -Shenzhen, China	0.0979	0.0719	1.10	[0.96; 1.27]	1.5%
Pan (2022) -Shenzhen, China	0.0908	0.0718	1.09	[0.95; 1.26]	1.5%
Ji (2011) -Suzhou, China	-0.5504	0.5547	0.58	[0.19; 1.71]	1.4%
Pan (2019) -Panzhihua, China	-0.3698	0.3375	0.69	[0.36; 1.34]	1.5%
Pan (2019) -Panzhihua, China	-1.4494	0.3617	0.23	[0.12; 0.48]	1.4%
Zheng (2014) -Jiangsu, China	-0.2801	0.3207	0.76	[0.40; 1.42]	1.5%
Nicklas (2016) -Gothenburg, Sweden	-6.1193	0.6078	0.00	[0.00; 0.01]	1.3%
Jean-Baptist (2009) -Rheinland-Pfalz, Mainz	-1.2680	0.2190	0.28	[0.18; 0.43]	1.5%
Cheng (2022) -Macao, China	-0.0617	0.0951	0.94	[0.78; 1.13]	1.5%
Li (2020) -Shanghai, China	-1.8793	0.4090	0.15	[0.07; 0.34]	1.4%
Li (2020) -Shanghai, China	-1.9317	0.4117	0.14	[0.06; 0.32]	1.4%
Li (2020) -Shanghai, China	-1.7277	0.4025	0.18	[0.08; 0.39]	1.4%
Rory (2019) -Edinburgh, UK	-0.1393	0.0138	0.87	[0.85; 0.89]	1.5%
Maria (2009) -Kathmandu, Nepal	-1.1829	0.6668	0.31	[0.08; 1.13]	1.3%
Li (2013) -Zhuhai, China	0.0508	1.2972	1.05	[0.08; 13.37]	1.0%
Mariana (2004) -Buenos Aires, Argentina	-2.1507	0.5646	0.12	[0.04; 0.35]	1.4%
Pan (2022) -Shenzhen, China	-0.6182	0.0728	0.54	[0.47; 0.62]	1.5%
Pan (2022) -Shenzhen, China	-0.6259	0.0729	0.53	[0.46; 0.62]	1.5%
Pan (2022) -Shenzhen, China	-0.5766	0.0727	0.56	[0.49; 0.65]	1.5%
Pan (2019) -Panzhihua, China	0.2764	0.3367	1.32	[0.68; 2.55]	1.5%
Pan (2019) -Panzhihua, China	-0.4008	0.3378	0.67	[0.35; 1.30]	1.5%
Ji (2011) -Suzhou, China	0.4756	0.5532	1.61	[0.54; 4.76]	1.4%
Wei (2016) -Shanghai, China	-1.9421	0.6791	0.14	[0.04; 0.54]	1.3%
Lin (2015) -Quanzhou, China	0.1222	1.3027	1.13	[0.09; 14.52]	1.0%
Lin (2015) -Quanzhou, China	0.8879	1.3427	2.43	[0.17; 33.77]	0.9%
Chen (2016) -Suzhou, China	-0.6259	0.8280	0.53	[0.11; 2.71]	1.2%
Chen (2016) -Suzhou, China	-0.7404	0.8329	0.48	[0.09; 2.44]	1.2%
Zhang (2022) -Jiangyin, China	-1.0458	0.4587	0.35	[0.14; 0.86]	1.4%
Zhang (2022) -Jiangyin, China	-1.2595	0.4665	0.28	[0.11; 0.71]	1.4%
Can (2021) -China	-0.0619	0.0243	0.94	[0.90; 0.99]	1.5%
Can (2021) -Southern China	-0.0408	0.0291	0.96	[0.91; 1.02]	1.5%
Can (2021) -China	-0.0305	0.0263	0.97	[0.92; 1.02]	1.5%
Can (2021) -Southern China	-0.0101	0.0282	0.99	[0.94; 1.05]	1.5%
Can (2021) -China	-0.0305	0.0183	0.97	[0.94; 1.01]	1.5%
Can (2021) -Southern China	-0.0305	0.0211	0.97	[0.93; 1.01]	1.5%
Li (2020) -Shanghai, China	3.3925	0.2460	29.74	[18.36; 48.16]	1.5%
Li (2020) -Shanghai, China	3.1001	0.3334	22.20	[11.55; 42.67]	1.5%
Random effects model			0.48	[0.31; 0.74]	70.5%
Prediction interval				[0.02; 10.20]	
Heterogeneity: $I^2 = 95\%$, $\tau^2 = 2.2768$, $p < 0.01$					
Climate = Continental					
Adriana (2018) -Toronto, Canada	-0.0943	0.0084	0.91	[0.90; 0.93]	1.5%
Adriana (2018) -Toronto, Canada	-1.0581	0.2154	0.35	[0.23; 0.53]	1.5%
Adriana (2018) -Toronto, Canada	-1.5833	0.2257	0.21	[0.13; 0.32]	1.5%
Wei (2019) -Jinan, China	-2.9584	0.3284	0.05	[0.03; 0.10]	1.5%
Wei (2019) -Jinan, China	-2.2201	0.2980	0.11	[0.06; 0.19]	1.5%
Adriana (2018) -Toronto, Canada	-0.0619	0.0081	0.94	[0.93; 0.96]	1.5%
Adriana (2018) -Toronto, Canada	-1.8282	0.2317	0.16	[0.10; 0.25]	1.5%
Wei (2019) -Jinan, China	-1.8282	0.2845	0.16	[0.09; 0.28]	1.5%
You (2022) -Gwangju, Korea	-3.6156	0.7767	0.03	[0.01; 0.12]	1.3%
Adriana (2018) -Toronto, Canada	-0.0726	0.0055	0.93	[0.92; 0.94]	1.5%
Adriana (2018) -Toronto, Canada	-1.1829	0.2175	0.31	[0.20; 0.47]	1.5%
Cao (2010) -Beijing, China	-10.4143	0.7642	0.00	[0.00; 0.00]	1.3%
Can (2021) -Northern China	-0.0726	0.0275	0.93	[0.88; 0.98]	1.5%
Can (2021) -Northern China	-0.0408	0.0266	0.96	[0.91; 1.01]	1.5%
Can (2021) -Northern China	-0.0408	0.0187	0.96	[0.93; 1.00]	1.5%
Random effects model			0.18	[0.05; 0.62]	22.0%
Prediction interval				[0.00; 45.01]	
Heterogeneity: $I^2 = 97\%$, $\tau^2 = 6.1793$, $p < 0.01$					
Climate = Tropical					
Gwladys (2017) -Yaounde, Cameroon	-0.2838	0.1929	0.75	[0.52; 1.10]	1.5%
Annabel (2023) -Bukit Timah, Singapore	-0.0523	0.0180	0.95	[0.92; 0.98]	1.5%
Benjamin (2018) -Nha Trang, central Vietnam	-0.1625	0.0465	0.85	[0.78; 0.93]	1.5%
Gwladys (2017) -Yaounde, Cameroon	-0.1744	0.1926	0.84	[0.58; 1.23]	1.5%
Gwladys (2017) -Yaounde, Cameroon	-0.3425	0.1932	0.71	[0.49; 1.04]	1.5%
Random effects model			0.88	[0.79; 0.97]	7.5%
Prediction interval				[0.66; 1.17]	
Heterogeneity: $I^2 = 53\%$, $\tau^2 = 0.0054$, $p = 0.08$					
**Random effects model**			**0.40**	**[0.27; 0.61]**	**100.0%**
**Prediction interval**				**[0.01; 12.69]**	

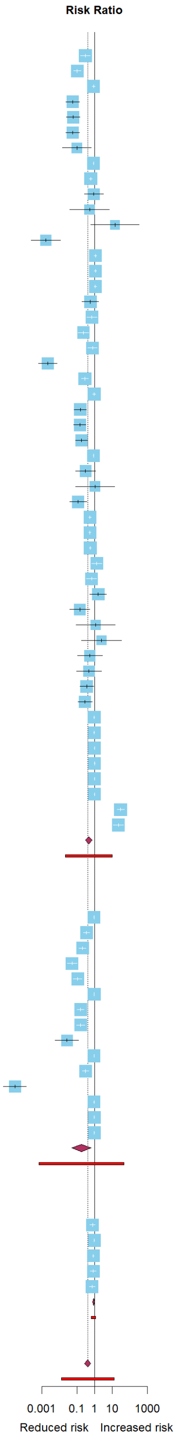

Heterogeneity: $I^2 = 96\%$, $\tau^2 = 2.9513$, $p < 0.01$
Test for subgroup differences: $\chi^2_2 = 13.05$, df = 2 ($p < 0.01$)

Classified as ECDC NORMAL

Figure S2-2. Subgroup analysis of influenza virus (IV) meta-analysis by World Bank income category

Study	logRR	SE(logRR)	RR	95%-CI	Weight
**Income = Upper-middle**					
Zheng (2014) -Jiangsu, China	-1.2040	0.3368	0.30	[0.16; 0.58]	1.5%
Li (2020) -Shanghai, China	-2.8665	0.4635	0.06	[0.02; 0.14]	1.4%
Li (2020) -Shanghai, China	-2.7920	0.4580	0.06	[0.02; 0.15]	1.4%
Li (2020) -Shanghai, China	-2.8665	0.4635	0.06	[0.02; 0.14]	1.4%
Chen (2014) -Suzhou, China	-2.3279	0.9684	0.10	[0.01; 0.65]	1.1%
Cui (2015) -Chaoshan, China	-0.5108	0.0858	0.60	[0.51; 0.71]	1.5%
Li (2013) -Zhuhai, China	-0.6677	1.3186	0.51	[0.04; 6.80]	1.0%
Li (2013) -Zhuhai, China	2.6646	1.6075	14.36	[0.62; 335.42]	0.8%
Wei (2019) -Jinan, China	-2.9584	0.3284	0.05	[0.03; 0.10]	1.5%
Wei (2019) -Jinan, China	-2.2201	0.2980	0.11	[0.06; 0.19]	1.5%
Gwladys (2017) -Yaounde, Cameroon	-0.2838	0.1929	0.75	[0.52; 1.10]	1.5%
Pan (2022) -Shenzhen, China	0.1053	0.0719	1.11	[0.97; 1.28]	1.5%
Pan (2022) -Shenzhen, China	0.0979	0.0719	1.10	[0.96; 1.27]	1.5%
Pan (2022) -Shenzhen, China	0.0908	0.0718	1.09	[0.95; 1.26]	1.5%
Benjamin (2018) -Nha Trang, central Vietnam	-0.1625	0.0465	0.85	[0.78; 0.93]	1.5%
Ji (2011) -Suzhou, China	-0.5504	0.5547	0.58	[0.19; 1.71]	1.4%
Pan (2019) -Panzhihua, China	-0.3698	0.3375	0.69	[0.36; 1.34]	1.5%
Pan (2019) -Panzhihua, China	-1.4494	0.3617	0.23	[0.12; 0.48]	1.4%
Zheng (2014) -Jiangsu, China	-0.2801	0.3207	0.76	[0.40; 1.42]	1.5%
Li (2020) -Shanghai, China	-1.8793	0.4090	0.15	[0.07; 0.34]	1.4%
Li (2020) -Shanghai, China	-1.9317	0.4117	0.14	[0.06; 0.32]	1.4%
Li (2020) -Shanghai, China	-1.7277	0.4025	0.18	[0.08; 0.39]	1.4%
Li (2013) -Zhuhai, China	0.0508	1.2972	1.05	[0.08; 13.37]	1.0%
Wei (2019) -Jinan, China	-1.8282	0.2845	0.16	[0.09; 0.28]	1.5%
Gwladys (2017) -Yaounde, Cameroon	-0.1744	0.1926	0.84	[0.58; 1.23]	1.5%
Pan (2022) -Shenzhen, China	-0.6182	0.0728	0.54	[0.47; 0.62]	1.5%
Pan (2022) -Shenzhen, China	-0.6259	0.0729	0.53	[0.46; 0.62]	1.5%
Pan (2022) -Shenzhen, China	-0.5766	0.0727	0.56	[0.49; 0.65]	1.5%
Pan (2019) -Panzhihua, China	0.2764	0.3367	1.32	[0.68; 2.55]	1.5%
Pan (2019) -Panzhihua, China	-0.4008	0.3378	0.67	[0.35; 1.30]	1.5%
Ji (2011) -Suzhou, China	0.4756	0.5532	1.61	[0.54; 4.76]	1.4%
Wei (2016) -Shanghai, China	-1.9421	0.6791	0.14	[0.04; 0.54]	1.3%
Gwladys (2017) -Yaounde, Cameroon	-0.3425	0.1932	0.71	[0.49; 1.04]	1.5%
Lin (2015) -Quanzhou, China	0.1222	1.3027	1.13	[0.09; 14.52]	1.0%
Lin (2015) -Quanzhou, China	0.8879	1.3427	2.43	[0.17; 33.77]	0.9%
Chen (2016) -Suzhou, China	-0.6259	0.8280	0.53	[0.11; 2.71]	1.2%
Chen (2016) -Suzhou, China	-0.7404	0.8329	0.48	[0.09; 2.44]	1.2%
Zhang (2022) -Jiangyin, China	-1.0458	0.4587	0.35	[0.14; 0.86]	1.4%
Zhang (2022) -Jiangyin, China	-1.2595	0.4665	0.28	[0.11; 0.71]	1.4%
Cao (2010) -Beijing, China	-10.4143	0.7642	0.00	[0.00; 0.00]	1.3%
Can (2021) -China	-0.0619	0.0243	0.94	[0.90; 0.99]	1.5%
Can (2021) -Northern China	-0.0726	0.0275	0.93	[0.88; 0.98]	1.5%
Can (2021) -Southern China	-0.0408	0.0291	0.96	[0.91; 1.02]	1.5%
Can (2021) -China	-0.0305	0.0263	0.97	[0.92; 1.02]	1.5%
Can (2021) -Northern China	-0.0408	0.0266	0.96	[0.91; 1.01]	1.5%
Can (2021) -Southern China	-0.0101	0.0282	0.99	[0.94; 1.05]	1.5%
Can (2021) -China	-0.0305	0.0183	0.97	[0.94; 1.01]	1.5%
Can (2021) -Northern China	-0.0408	0.0187	0.96	[0.93; 1.00]	1.5%
Can (2021) -Southern China	-0.0305	0.0211	0.97	[0.93; 1.01]	1.5%
Li (2020) -Shanghai, China	3.3925	0.2460	29.74	[18.36; 48.16]	1.5%
Li (2020) -Shanghai, China	3.1001	0.3334	22.20	[11.55; 42.67]	1.5%
**Random effects model**			0.48	[0.30; 0.79]	71.2%
**Prediction interval**				[0.01; 15.98]	
Heterogeneity: $I^2 = 95\%$, $\tau^2 = 2.9649$, $p < 0.01$					
**Income = High**					
Jean-Baptist (2009) -Rheinland-Pfalz, Mainz	-2.2672	0.2439	0.10	[0.06; 0.17]	1.5%
Cheng (2022) -Macao, China	-0.1380	0.0951	0.87	[0.72; 1.05]	1.5%
Rory (2019) -Edinburgh, UK	-0.1473	0.0112	0.86	[0.84; 0.88]	1.5%
Adriana (2018) -Toronto, Canada	-0.0943	0.0084	0.91	[0.90; 0.93]	1.5%
Adriana (2018) -Toronto, Canada	-1.0581	0.2154	0.35	[0.23; 0.53]	1.5%
Adriana (2018) -Toronto, Canada	-1.5833	0.2257	0.21	[0.13; 0.32]	1.5%
Annabel (2023) -Bukit Timah, Singapore	-0.0523	0.0180	0.95	[0.92; 0.98]	1.5%
Mariana (2004) -Buenos Aires, Argentina	-6.3771	0.9767	0.00	[0.00; 0.01]	1.1%
Nicklas (2016) -Gothenburg, Sweden	-6.1193	0.6078	0.00	[0.00; 0.01]	1.3%
Jean-Baptist (2009) -Rheinland-Pfalz, Mainz	-1.2680	0.2190	0.28	[0.18; 0.43]	1.5%
Cheng (2022) -Macao, China	-0.0617	0.0951	0.94	[0.78; 1.13]	1.5%
Rory (2019) -Edinburgh, UK	-0.1393	0.0138	0.87	[0.85; 0.89]	1.5%
Adriana (2018) -Toronto, Canada	-0.0619	0.0081	0.94	[0.93; 0.96]	1.5%
Adriana (2018) -Toronto, Canada	-1.8282	0.2317	0.16	[0.10; 0.25]	1.5%
Mariana (2004) -Buenos Aires, Argentina	-2.1507	0.5646	0.12	[0.04; 0.35]	1.4%
You (2022) -Gwangju, Korea	-3.6156	0.7767	0.03	[0.01; 0.12]	1.3%
Adriana (2018) -Toronto, Canada	-0.0726	0.0055	0.93	[0.92; 0.94]	1.5%
Adriana (2018) -Toronto, Canada	-1.1829	0.2175	0.31	[0.20; 0.47]	1.5%
**Random effects model**			0.23	[0.10; 0.54]	26.1%
**Prediction interval**				[0.00; 11.06]	
Heterogeneity: $I^2 = 97\%$, $\tau^2 = 3.1284$, $p < 0.01$					
**Income = Lower**					
Maria (2009) -Kathmandu, Nepal	-0.1234	0.6343	0.88	[0.25; 3.06]	1.3%
Maria (2009) -Kathmandu, Nepal	-1.1829	0.6668	0.31	[0.08; 1.13]	1.3%
**Random effects model**			0.53	[0.19; 1.50]	2.6%
**Prediction interval**					
Heterogeneity: $I^2 = 25\%$, $\tau^2 = 0.1377$, $p = 0.25$					
**Random effects model**			**0.40**	**[0.27; 0.61]**	**100.0%**
**Prediction interval**				**[0.01; 12.69]**	

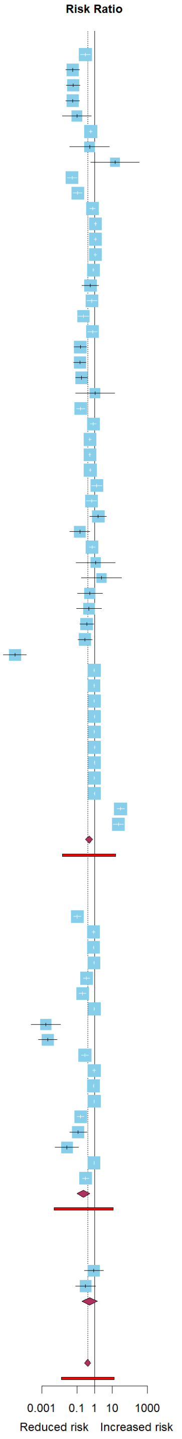

Heterogeneity: $I^2 = 96\%$, $\tau^2 = 2.9513$, $p < 0.01$
Test for subgroup differences: $\chi^2_2 = 2.41$, df = 2 ($p = 0.30$)

Classified as ECDC NORMAL

Figure S2-3. Subgroup analysis of influenza virus (IV) meta-analysis by temporal resolution

Study	logRR	SE(logRR)	RR	95%-CI	Weight
Resolution = Monthly					
Zheng (2014) -Jiangsu, China	-1.2040	0.3368	0.30	[0.16; 0.58]	1.5%
Chen (2014) -Suzhou, China	-2.3279	0.9684	0.10	[0.01; 0.65]	1.1%
Rory (2019) -Edinburgh, UK	-0.1473	0.0112	0.86	[0.84; 0.88]	1.5%
Maria (2009) -Kathmandu, Nepal	-0.1234	0.6343	0.88	[0.25; 3.06]	1.3%
Li (2013) -Zhuhai, China	-0.6677	1.3186	0.51	[0.04; 6.80]	1.0%
Li (2013) -Zhuhai, China	2.6646	1.6075	14.36	[0.62; 335.42]	0.8%
Mariana (2004) -Buenos Aires, Argentina	-6.3771	0.9767	0.00	[0.00; 0.01]	1.1%
Benjamin (2018) -Nha Trang, central Vietnam	-0.1625	0.0465	0.85	[0.78; 0.93]	1.5%
Ji (2011) -Suzhou, China	-0.5504	0.5547	0.58	[0.19; 1.71]	1.4%
Pan (2019) -Panzhihua, China	-0.3698	0.3375	0.69	[0.36; 1.34]	1.5%
Pan (2019) -Panzhihua, China	-1.4494	0.3617	0.23	[0.12; 0.48]	1.4%
Zheng (2014) -Jiangsu, China	-0.2801	0.3207	0.76	[0.40; 1.42]	1.5%
Rory (2019) -Edinburgh, UK	-0.1393	0.0138	0.87	[0.85; 0.89]	1.5%
Maria (2009) -Kathmandu, Nepal	-1.1829	0.6668	0.31	[0.08; 1.13]	1.3%
Li (2013) -Zhuhai, China	0.0508	1.2972	1.05	[0.08; 13.37]	1.0%
Mariana (2004) -Buenos Aires, Argentina	-2.1507	0.5646	0.12	[0.04; 0.35]	1.4%
Pan (2019) -Panzhihua, China	0.2764	0.3367	1.32	[0.68; 2.55]	1.5%
Pan (2019) -Panzhihua, China	-0.4008	0.3378	0.67	[0.35; 1.30]	1.5%
Ji (2011) -Suzhou, China	0.4756	0.5532	1.61	[0.54; 4.76]	1.4%
You (2022) -Gwangju, Korea	-3.6156	0.7767	0.03	[0.01; 0.12]	1.3%
Wei (2016) -Shanghai, China	-1.9421	0.6791	0.14	[0.04; 0.54]	1.3%
Lin (2015) -Quanzhou, China	0.1222	1.3027	1.13	[0.09; 14.52]	1.0%
Lin (2015) -Quanzhou, China	0.8879	1.3427	2.43	[0.17; 33.77]	0.9%
Chen (2016) -Suzhou, China	-0.6259	0.8280	0.53	[0.11; 2.71]	1.2%
Chen (2016) -Suzhou, China	-0.7404	0.8329	0.48	[0.09; 2.44]	1.2%
Zhang (2022) -Jiangyin, China	-1.0458	0.4587	0.35	[0.14; 0.86]	1.4%
Zhang (2022) -Jiangyin, China	-1.2595	0.4665	0.28	[0.11; 0.71]	1.4%
Random effects model			0.43	[0.27; 0.69]	34.8%
Prediction interval				[0.04; 4.22]	
Heterogeneity: $I^2 = 80\%$, $\tau^2 = 1.1751$, $p < 0.01$					
Resolution = Weekly					
Jean-Baptist (2009) -Rheinland-Pfalz, Mainz	-2.2672	0.2439	0.10	[0.06; 0.17]	1.5%
Li (2020) -Shanghai, China	-2.8665	0.4635	0.06	[0.02; 0.14]	1.4%
Li (2020) -Shanghai, China	-2.7920	0.4580	0.06	[0.02; 0.15]	1.4%
Li (2020) -Shanghai, China	-2.8665	0.4635	0.06	[0.02; 0.14]	1.4%
Adriana (2018) -Toronto, Canada	-0.0943	0.0084	0.91	[0.90; 0.93]	1.5%
Adriana (2018) -Toronto, Canada	-1.0581	0.2154	0.35	[0.23; 0.53]	1.5%
Adriana (2018) -Toronto, Canada	-1.5833	0.2257	0.21	[0.13; 0.32]	1.5%
Wei (2019) -Jinan, China	-2.9584	0.3284	0.05	[0.03; 0.10]	1.5%
Wei (2019) -Jinan, China	-2.2201	0.2980	0.11	[0.06; 0.19]	1.5%
Gwladys (2017) -Yaounde, Cameroon	-0.2838	0.1929	0.75	[0.52; 1.10]	1.5%
Nicklas (2016) -Gothenburg, Sweden	-6.1193	0.6078	0.00	[0.00; 0.01]	1.3%
Jean-Baptist (2009) -Rheinland-Pfalz, Mainz	-1.2680	0.2190	0.28	[0.18; 0.43]	1.5%
Li (2020) -Shanghai, China	-1.8793	0.4090	0.15	[0.07; 0.34]	1.4%
Li (2020) -Shanghai, China	-1.9317	0.4117	0.14	[0.06; 0.32]	1.4%
Li (2020) -Shanghai, China	-1.7277	0.4025	0.18	[0.08; 0.39]	1.4%
Adriana (2018) -Toronto, Canada	-0.0619	0.0081	0.94	[0.93; 0.96]	1.5%
Adriana (2018) -Toronto, Canada	-1.8282	0.2317	0.16	[0.10; 0.25]	1.5%
Wei (2019) -Jinan, China	-1.8282	0.2845	0.16	[0.09; 0.28]	1.5%
Gwladys (2017) -Yaounde, Cameroon	-0.1744	0.1926	0.84	[0.58; 1.23]	1.5%
Adriana (2018) -Toronto, Canada	-0.0726	0.0055	0.93	[0.92; 0.94]	1.5%
Adriana (2018) -Toronto, Canada	-1.1829	0.2175	0.31	[0.20; 0.47]	1.5%
Gwladys (2017) -Yaounde, Cameroon	-0.3425	0.1932	0.71	[0.49; 1.04]	1.5%
Can (2021) -China	-0.0619	0.0243	0.94	[0.90; 0.99]	1.5%
Can (2021) -Northern China	-0.0726	0.0275	0.93	[0.88; 0.98]	1.5%
Can (2021) -Southern China	-0.0408	0.0291	0.96	[0.91; 1.02]	1.5%
Can (2021) -China	-0.0305	0.0263	0.97	[0.92; 1.02]	1.5%
Can (2021) -Northern China	-0.0408	0.0266	0.96	[0.91; 1.01]	1.5%
Can (2021) -Southern China	-0.0101	0.0282	0.99	[0.94; 1.05]	1.5%
Can (2021) -China	-0.0305	0.0183	0.97	[0.94; 1.01]	1.5%
Can (2021) -Northern China	-0.0408	0.0187	0.96	[0.93; 1.00]	1.5%
Can (2021) -Southern China	-0.0305	0.0211	0.97	[0.93; 1.01]	1.5%
Li (2020) -Shanghai, China	3.3925	0.2460	29.74	[18.36; 48.16]	1.5%
Li (2020) -Shanghai, China	3.1001	0.3334	22.20	[11.55; 42.67]	1.5%
Random effects model			0.40	[0.23; 0.71]	48.8%
Prediction interval				[0.01; 12.31]	
Heterogeneity: $I^2 = 97\%$, $\tau^2 = 2.7323$, $p < 0.01$					
Resolution = Daily					
Cheng (2022) -Macao, China	-0.1380	0.0951	0.87	[0.72; 1.05]	1.5%
Cui (2015) -Chaoshan, China	-0.5108	0.0858	0.60	[0.51; 0.71]	1.5%
Annabel (2023) -Bukit Timah, Singapore	-0.0523	0.0180	0.95	[0.92; 0.98]	1.5%
Pan (2022) -Shenzhen, China	0.1053	0.0719	1.11	[0.97; 1.28]	1.5%
Pan (2022) -Shenzhen, China	0.0979	0.0719	1.10	[0.96; 1.27]	1.5%
Pan (2022) -Shenzhen, China	0.0908	0.0718	1.09	[0.95; 1.26]	1.5%
Cheng (2022) -Macao, China	-0.0617	0.0951	0.94	[0.78; 1.13]	1.5%
Pan (2022) -Shenzhen, China	-0.6182	0.0728	0.54	[0.47; 0.62]	1.5%
Pan (2022) -Shenzhen, China	-0.6259	0.0729	0.53	[0.46; 0.62]	1.5%
Pan (2022) -Shenzhen, China	-0.5766	0.0727	0.56	[0.49; 0.65]	1.5%
Cao (2010) -Beijing, China	-10.4143	0.7642	0.00	[0.00; 0.00]	1.3%
Random effects model			0.33	[0.06; 1.87]	16.4%
Prediction interval				[0.00; 326.61]	
Heterogeneity: $I^2 = 97\%$, $\tau^2 = 8.4984$, $p < 0.01$					
**Random effects model**			**0.40**	**[0.27; 0.61]**	**100.0%**
**Prediction interval**				**[0.01; 12.69]**	

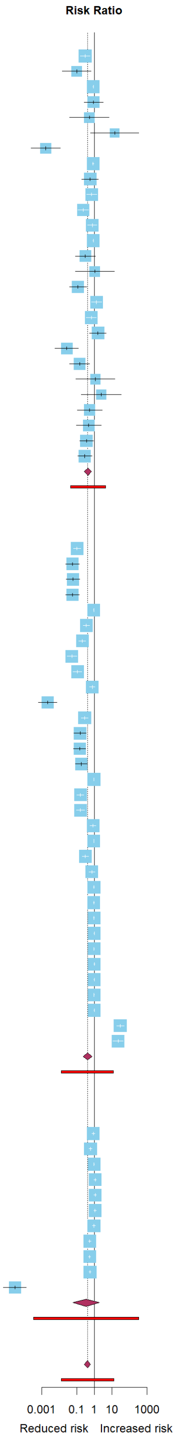

Heterogeneity: $I^2 = 96\%$, $\tau^2 = 2.9513$, $p < 0.01$
Test for subgroup differences: $\chi^2_2 = 0.09$, df = 2 ($p = 0.95$)

Classified as ECDC NORMAL

Figure S2-4. Subgroup analysis of influenza virus (IV) meta-analysis exposure measure

Study	logRR	SE(logRR)	RR	95%-CI	Weight
**Temperature = Mean**					
Zheng (2014) -Jiangsu, China	-1.2040	0.3368	0.30	[0.16; 0.58]	1.5%
Jean-Baptist (2009) -Rheinland-Pfalz, Mainz	-2.2672	0.2439	0.10	[0.06; 0.17]	1.5%
Cheng (2022) -Macao, China	-0.1380	0.0951	0.87	[0.72; 1.05]	1.5%
Li (2020) -Shanghai, China	-2.8665	0.4635	0.06	[0.02; 0.14]	1.4%
Chen (2014) -Suzhou, China	-2.3279	0.9684	0.10	[0.01; 0.65]	1.1%
Rory (2019) -Edinburgh, UK	-0.1473	0.0112	0.86	[0.84; 0.88]	1.5%
Cui (2015) -Chaoshan, China	-0.5108	0.0858	0.60	[0.51; 0.71]	1.5%
Maria (2009) -Kathmandu, Nepal	-0.1234	0.6343	0.88	[0.25; 3.06]	1.3%
Li (2013) -Zhuhai, China	-0.6677	1.3186	0.51	[0.04; 6.80]	1.0%
Li (2013) -Zhuhai, China	2.6646	1.6075	14.36	[0.62; 335.42]	0.8%
Adriana (2018) -Toronto, Canada	-0.0943	0.0084	0.91	[0.90; 0.93]	1.5%
Adriana (2018) -Toronto, Canada	-1.0581	0.2154	0.35	[0.23; 0.53]	1.5%
Adriana (2018) -Toronto, Canada	-1.5833	0.2257	0.21	[0.13; 0.32]	1.5%
Wei (2019) -Jinan, China	-2.9584	0.3284	0.05	[0.03; 0.10]	1.5%
Wei (2019) -Jinan, China	-2.2201	0.2980	0.11	[0.06; 0.19]	1.5%
Gwladys (2017) -Yaounde, Cameroon	-0.2838	0.1929	0.75	[0.52; 1.10]	1.5%
Annabel (2023) -Bukit Timah, Singapore	-0.0523	0.0180	0.95	[0.92; 0.98]	1.5%
Mariana (2004) -Buenos Aires, Argentina	-6.3771	0.9767	0.00	[0.00; 0.01]	1.1%
Pan (2022) -Shenzhen, China	0.1053	0.0719	1.11	[0.97; 1.28]	1.5%
Benjamin (2018) -Nha Trang, central Vietnam	-0.1625	0.0465	0.85	[0.78; 0.93]	1.5%
Ji (2011) -Suzhou, China	-0.5504	0.5547	0.58	[0.19; 1.71]	1.4%
Pan (2019) -Panzhihua, China	-0.3698	0.3375	0.69	[0.36; 1.34]	1.5%
Pan (2019) -Panzhihua, China	-1.4494	0.3617	0.23	[0.12; 0.48]	1.4%
Zheng (2014) -Jiangsu, China	-0.2801	0.3207	0.76	[0.40; 1.42]	1.5%
Nicklas (2016) -Gothenburg, Sweden	-6.1193	0.6078	0.00	[0.00; 0.01]	1.3%
Jean-Baptist (2009) -Rheinland-Pfalz, Mainz	-1.2680	0.2190	0.28	[0.18; 0.43]	1.5%
Cheng (2022) -Macao, China	-0.0617	0.0951	0.94	[0.78; 1.13]	1.5%
Li (2020) -Shanghai, China	-1.8793	0.4090	0.15	[0.07; 0.34]	1.4%
Rory (2019) -Edinburgh, UK	-0.1393	0.0138	0.87	[0.85; 0.89]	1.5%
Maria (2009) -Kathmandu, Nepal	-1.1829	0.6668	0.31	[0.08; 1.13]	1.3%
Li (2013) -Zhuhai, China	0.0508	1.2972	1.05	[0.08; 13.37]	1.0%
Adriana (2018) -Toronto, Canada	-0.0619	0.0081	0.94	[0.93; 0.96]	1.5%
Adriana (2018) -Toronto, Canada	-1.8282	0.2317	0.16	[0.10; 0.25]	1.5%
Wei (2019) -Jinan, China	-1.8282	0.2845	0.16	[0.09; 0.28]	1.5%
Gwladys (2017) -Yaounde, Cameroon	-0.1744	0.1926	0.84	[0.58; 1.23]	1.5%
Mariana (2004) -Buenos Aires, Argentina	-2.1507	0.5646	0.12	[0.04; 0.35]	1.4%
Pan (2022) -Shenzhen, China	-0.6182	0.0728	0.54	[0.47; 0.62]	1.5%
Pan (2019) -Panzhihua, China	0.2764	0.3367	1.32	[0.68; 2.55]	1.5%
Pan (2019) -Panzhihua, China	-0.4008	0.3378	0.67	[0.35; 1.30]	1.5%
Ji (2011) -Suzhou, China	0.4756	0.5532	1.61	[0.54; 4.76]	1.4%
You (2022) -Gwangju, Korea	-3.6156	0.7767	0.03	[0.01; 0.12]	1.3%
Adriana (2018) -Toronto, Canada	-0.0726	0.0055	0.93	[0.92; 0.94]	1.5%
Adriana (2018) -Toronto, Canada	-1.1829	0.2175	0.31	[0.20; 0.47]	1.5%
Wei (2016) -Shanghai, China	-1.9421	0.6791	0.14	[0.04; 0.54]	1.3%
Gwladys (2017) -Yaounde, Cameroon	-0.3425	0.1932	0.71	[0.49; 1.04]	1.5%
Lin (2015) -Quanzhou, China	0.1222	1.3027	1.13	[0.09; 14.52]	1.0%
Lin (2015) -Quanzhou, China	0.8879	1.3427	2.43	[0.17; 33.77]	0.9%
Chen (2016) -Suzhou, China	-0.6259	0.8280	0.53	[0.11; 2.71]	1.2%
Chen (2016) -Suzhou, China	-0.7404	0.8329	0.48	[0.09; 2.44]	1.2%
Zhang (2022) -Jiangyin, China	-1.0458	0.4587	0.35	[0.14; 0.86]	1.4%
Zhang (2022) -Jiangyin, China	-1.2595	0.4665	0.28	[0.11; 0.71]	1.4%
Cao (2010) -Beijing, China	-10.4143	0.7642	0.00	[0.00; 0.00]	1.3%
Can (2021) -China	-0.0619	0.0243	0.94	[0.90; 0.99]	1.5%
Can (2021) -Northern China	-0.0726	0.0275	0.93	[0.88; 0.98]	1.5%
Can (2021) -Southern China	-0.0408	0.0291	0.96	[0.91; 1.02]	1.5%
Can (2021) -China	-0.0305	0.0263	0.97	[0.92; 1.02]	1.5%
Can (2021) -Northern China	-0.0408	0.0266	0.96	[0.91; 1.01]	1.5%
Can (2021) -Southern China	-0.0101	0.0282	0.99	[0.94; 1.05]	1.5%
Can (2021) -China	-0.0305	0.0183	0.97	[0.94; 1.01]	1.5%
Can (2021) -Northern China	-0.0408	0.0187	0.96	[0.93; 1.00]	1.5%
Can (2021) -Southern China	-0.0305	0.0211	0.97	[0.93; 1.01]	1.5%
Li (2020) -Shanghai, China	3.3925	0.2460	29.74	[18.36; 48.16]	1.5%
Li (2020) -Shanghai, China	3.1001	0.3334	22.20	[11.55; 42.67]	1.5%
Random effects model			0.42	[0.26; 0.66]	88.3%
Prediction interval				[0.01; 15.64]	
Heterogeneity: $I^2 = 96\%$, $\tau^2 = 3.2193$, $p < 0.01$					
**Temperature = Min**					
Li (2020) -Shanghai, China	-2.7920	0.4580	0.06	[0.02; 0.15]	1.4%
Pan (2022) -Shenzhen, China	0.0979	0.0719	1.10	[0.96; 1.27]	1.5%
Li (2020) -Shanghai, China	-1.9317	0.4117	0.14	[0.06; 0.32]	1.4%
Pan (2022) -Shenzhen, China	-0.6259	0.0729	0.53	[0.46; 0.62]	1.5%
Random effects model			0.29	[0.08; 1.01]	5.9%
Prediction interval				[0.00; 120.84]	
Heterogeneity: $I^2 = 97\%$, $\tau^2 = 1.5616$, $p < 0.01$					
**Temperature = Max**					
Li (2020) -Shanghai, China	-2.8665	0.4635	0.06	[0.02; 0.14]	1.4%
Pan (2022) -Shenzhen, China	0.0908	0.0718	1.09	[0.95; 1.26]	1.5%
Li (2020) -Shanghai, China	-1.7277	0.4025	0.18	[0.08; 0.39]	1.4%
Pan (2022) -Shenzhen, China	-0.5766	0.0727	0.56	[0.49; 0.65]	1.5%
Random effects model			0.30	[0.08; 1.05]	5.9%
Prediction interval				[0.00; 126.00]	
Heterogeneity: $I^2 = 97\%$, $\tau^2 = 1.5611$, $p < 0.01$					
**Random effects model**			**0.40**	**[0.27; 0.61]**	**100.0%**
**Prediction interval**				**[0.01; 12.69]**	

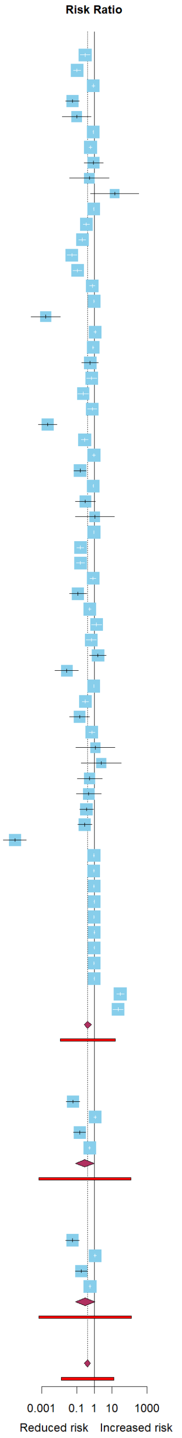

Heterogeneity: $I^2 = 96\%$, $\tau^2 = 2.9513$, $p < 0.01$
Test for subgroup differences: $\chi^2_2 = 0.50$, df = 2 ($p = 0.78$)

Classified as ECDC NORMAL

Figure S2-5. Subgroup analysis of influenza virus (IV) meta-analysis by modelling approach

Study	logRR	SE(logRR)	RR	95%-CI	Weight
Model = linear					
Zheng (2014) -Jiangsu, China	-1.2040	0.3368	0.30	[0.16; 0.58]	1.5%
Cheng (2022) -Macao, China	-0.1380	0.0951	0.87	[0.72; 1.05]	1.5%
Rory (2019) -Edinburgh, UK	-0.1473	0.0112	0.86	[0.84; 0.88]	1.5%
Adriana (2018) -Toronto, Canada	-0.0943	0.0084	0.91	[0.90; 0.93]	1.5%
Gwladys (2017) -Yaounde, Cameroon	-0.2838	0.1929	0.75	[0.52; 1.10]	1.5%
Annabel (2023) -Bukit Timah, Singapore	-0.0523	0.0180	0.95	[0.92; 0.98]	1.5%
Ji (2011) -Suzhou, China	-0.5504	0.5547	0.58	[0.19; 1.71]	1.4%
Pan (2019) -Panzhihua, China	-0.3698	0.3375	0.69	[0.36; 1.34]	1.5%
Pan (2019) -Panzhihua, China	-1.4494	0.3617	0.23	[0.12; 0.48]	1.4%
Zheng (2014) -Jiangsu, China	-0.2801	0.3207	0.76	[0.40; 1.42]	1.5%
Nicklas (2016) -Gothenburg, Sweden	-6.1193	0.6078	0.00	[0.00; 0.01]	1.3%
Cheng (2022) -Macao, China	-0.0617	0.0951	0.94	[0.78; 1.13]	1.5%
Rory (2019) -Edinburgh, UK	-0.1393	0.0138	0.87	[0.85; 0.89]	1.5%
Adriana (2018) -Toronto, Canada	-0.0619	0.0081	0.94	[0.93; 0.96]	1.5%
Gwladys (2017) -Yaounde, Cameroon	-0.1744	0.1926	0.84	[0.58; 1.23]	1.5%
Pan (2019) -Panzhihua, China	0.2764	0.3367	1.32	[0.68; 2.55]	1.5%
Pan (2019) -Panzhihua, China	-0.4008	0.3378	0.67	[0.35; 1.30]	1.5%
Ji (2011) -Suzhou, China	0.4756	0.5532	1.61	[0.54; 4.76]	1.4%
Adriana (2018) -Toronto, Canada	-0.0726	0.0055	0.93	[0.92; 0.94]	1.5%
Gwladys (2017) -Yaounde, Cameroon	-0.3425	0.1932	0.71	[0.49; 1.04]	1.5%
Lin (2015) -Quanzhou, China	0.1222	1.3027	1.13	[0.09; 14.52]	1.0%
Lin (2015) -Quanzhou, China	0.8879	1.3427	2.43	[0.17; 33.77]	0.9%
Random effects model			0.63	[0.39; 1.03]	31.3%
Prediction interval				[0.06; 6.69]	
Heterogeneity: I^2 = 89%, τ^2 = 1.2183, p < 0.01					
Model = non-linear					
Jean-Baptist (2009) -Rheinland-Pfalz, Mainz	-2.2672	0.2439	0.10	[0.06; 0.17]	1.5%
Li (2020) -Shanghai, China	-2.8665	0.4635	0.06	[0.02; 0.14]	1.4%
Li (2020) -Shanghai, China	-2.7920	0.4580	0.06	[0.02; 0.15]	1.4%
Li (2020) -Shanghai, China	-2.8665	0.4635	0.06	[0.02; 0.14]	1.4%
Chen (2014) -Suzhou, China	-2.3279	0.9684	0.10	[0.01; 0.65]	1.1%
Cui (2015) -Chaoshan, China	-0.5108	0.0858	0.60	[0.51; 0.71]	1.5%
Maria (2009) -Kathmandu, Nepal	-0.1234	0.6343	0.88	[0.25; 3.06]	1.3%
Li (2013) -Zhuhai, China	-0.6677	1.3186	0.51	[0.04; 6.80]	1.0%
Li (2013) -Zhuhai, China	2.6646	1.6075	14.36	[0.62; 335.42]	0.8%
Adriana (2018) -Toronto, Canada	-1.0581	0.2154	0.35	[0.23; 0.53]	1.5%
Adriana (2018) -Toronto, Canada	-1.5833	0.2257	0.21	[0.13; 0.32]	1.5%
Wei (2019) -Jinan, China	-2.9584	0.3284	0.05	[0.03; 0.10]	1.5%
Wei (2019) -Jinan, China	-2.2201	0.2980	0.11	[0.06; 0.19]	1.5%
Mariana (2004) -Buenos Aires, Argentina	-6.3771	0.9767	0.00	[0.00; 0.01]	1.1%
Pan (2022) -Shenzhen, China	0.1053	0.0719	1.11	[0.97; 1.28]	1.5%
Pan (2022) -Shenzhen, China	0.0979	0.0719	1.10	[0.96; 1.27]	1.5%
Pan (2022) -Shenzhen, China	0.0908	0.0718	1.09	[0.95; 1.26]	1.5%
Benjamin (2018) -Nha Trang, central Vietnam	-0.1625	0.0465	0.85	[0.78; 0.93]	1.5%
Jean-Baptist (2009) -Rheinland-Pfalz, Mainz	-1.2680	0.2190	0.28	[0.18; 0.43]	1.5%
Li (2020) -Shanghai, China	-1.8793	0.4090	0.15	[0.07; 0.34]	1.4%
Li (2020) -Shanghai, China	-1.9317	0.4117	0.14	[0.06; 0.32]	1.4%
Li (2020) -Shanghai, China	-1.7277	0.4025	0.18	[0.08; 0.39]	1.4%
Maria (2009) -Kathmandu, Nepal	-1.1829	0.6668	0.31	[0.08; 1.13]	1.3%
Li (2013) -Zhuhai, China	0.0508	1.2972	1.05	[0.08; 13.37]	1.0%
Adriana (2018) -Toronto, Canada	-1.8282	0.2317	0.16	[0.10; 0.25]	1.5%
Wei (2019) -Jinan, China	-1.8282	0.2845	0.16	[0.09; 0.28]	1.5%
Mariana (2004) -Buenos Aires, Argentina	-2.1507	0.5646	0.12	[0.04; 0.35]	1.4%
Pan (2022) -Shenzhen, China	-0.6182	0.0728	0.54	[0.47; 0.62]	1.5%
Pan (2022) -Shenzhen, China	-0.6259	0.0729	0.53	[0.46; 0.62]	1.5%
Pan (2022) -Shenzhen, China	-0.5766	0.0727	0.56	[0.49; 0.65]	1.5%
You (2022) -Gwangju, Korea	-3.6156	0.7767	0.03	[0.01; 0.12]	1.3%
Adriana (2018) -Toronto, Canada	-1.1829	0.2175	0.31	[0.20; 0.47]	1.5%
Wei (2016) -Shanghai, China	-1.9421	0.6791	0.14	[0.04; 0.54]	1.3%
Chen (2016) -Suzhou, China	-0.6259	0.8280	0.53	[0.11; 2.71]	1.2%
Chen (2016) -Suzhou, China	-0.7404	0.8329	0.48	[0.09; 2.44]	1.2%
Zhang (2022) -Jiangyin, China	-1.0458	0.4587	0.35	[0.14; 0.86]	1.4%
Zhang (2022) -Jiangyin, China	-1.2595	0.4665	0.28	[0.11; 0.71]	1.4%
Cao (2010) -Beijing, China	-10.4143	0.7642	0.00	[0.00; 0.00]	1.3%
Can (2021) -China	-0.0619	0.0243	0.94	[0.90; 0.99]	1.5%
Can (2021) -Northern China	-0.0726	0.0275	0.93	[0.88; 0.98]	1.5%
Can (2021) -Southern China	-0.0408	0.0291	0.96	[0.91; 1.02]	1.5%
Can (2021) -China	-0.0305	0.0263	0.97	[0.92; 1.02]	1.5%
Can (2021) -Northern China	-0.0408	0.0266	0.96	[0.91; 1.01]	1.5%
Can (2021) -Southern China	-0.0101	0.0282	0.99	[0.94; 1.05]	1.5%
Can (2021) -China	-0.0305	0.0183	0.97	[0.94; 1.01]	1.5%
Can (2021) -Northern China	-0.0408	0.0187	0.96	[0.93; 1.00]	1.5%
Can (2021) -Southern China	-0.0305	0.0211	0.97	[0.93; 1.01]	1.5%
Li (2020) -Shanghai, China	3.3925	0.2460	29.74	[18.36; 48.16]	1.5%
Li (2020) -Shanghai, China	3.1001	0.3334	22.20	[11.55; 42.67]	1.5%
Random effects model			0.33	[0.19; 0.57]	68.7%
Prediction interval				[0.01; 16.04]	
Heterogeneity: I^2 = 97%, τ^2 = 3.6723, p < 0.01					
**Random effects model**			**0.40**	**[0.27; 0.61]**	**100.0%**
**Prediction interval**				**[0.01; 12.69]**	

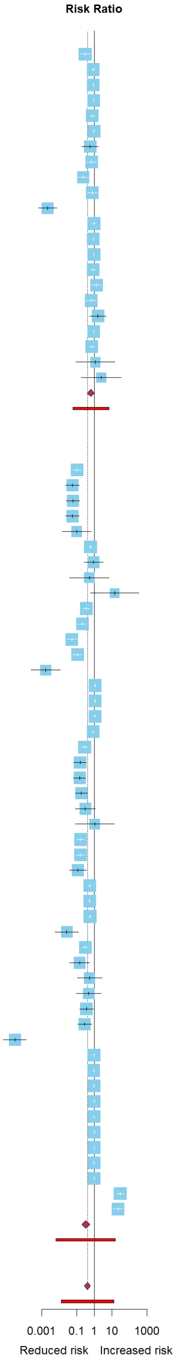

Heterogeneity: I^2 = 96%, τ^2 = 2.9513, p < 0.01
Test for subgroup differences: χ^2_1 = 3.07, df = 1 (p = 0.08)

Classified as ECDC NORMAL

Figure S2-6. Subgroup analysis of influenza virus (IV) meta-analysis by lag type

Study	logRR	SE(logRR)	RR	95%-CI	Weight
**Lag = 0**					
Zheng (2014) -Jiangsu, China	-1.2040	0.3368	0.30	[0.16; 0.58]	1.5%
Jean-Baptist (2009) -Rheinland-Pfalz, Mainz	-2.2672	0.2439	0.10	[0.06; 0.17]	1.5%
Cheng (2022) -Macao, China	-0.1380	0.0951	0.87	[0.72; 1.05]	1.5%
Li (2020) -Shanghai, China	-2.8665	0.4635	0.06	[0.02; 0.14]	1.4%
Li (2020) -Shanghai, China	-2.7920	0.4580	0.06	[0.02; 0.15]	1.4%
Li (2020) -Shanghai, China	-2.8665	0.4635	0.06	[0.02; 0.14]	1.4%
Chen (2014) -Suzhou, China	-2.3279	0.9684	0.10	[0.01; 0.65]	1.1%
Rory (2019) -Edinburgh, UK	-0.1473	0.0112	0.86	[0.84; 0.88]	1.5%
Cui (2015) -Chaoshan, China	-0.5108	0.0858	0.60	[0.51; 0.71]	1.5%
Maria (2009) -Kathmandu, Nepal	-0.1234	0.6343	0.88	[0.25; 3.06]	1.3%
Li (2013) -Zhuhai, China	-0.6677	1.3186	0.51	[0.04; 6.80]	1.0%
Li (2013) -Zhuhai, China	2.6646	1.6075	14.36	[0.62; 335.42]	0.8%
Adriana (2018) -Toronto, Canada	-0.0943	0.0084	0.91	[0.90; 0.93]	1.5%
Adriana (2018) -Toronto, Canada	-1.0581	0.2154	0.35	[0.23; 0.53]	1.5%
Adriana (2018) -Toronto, Canada	-1.5833	0.2257	0.21	[0.13; 0.32]	1.5%
Wei (2019) -Jinan, China	-2.9584	0.3284	0.05	[0.03; 0.10]	1.5%
Wei (2019) -Jinan, China	-2.2201	0.2980	0.11	[0.06; 0.19]	1.5%
Gwladys (2017) -Yaounde, Cameroon	-0.2838	0.1929	0.75	[0.52; 1.10]	1.5%
Mariana (2004) -Buenos Aires, Argentina	-6.3771	0.9767	0.00	[0.00; 0.01]	1.1%
Pan (2022) -Shenzhen, China	0.1053	0.0719	1.11	[0.97; 1.28]	1.5%
Pan (2022) -Shenzhen, China	0.0979	0.0719	1.10	[0.96; 1.27]	1.5%
Pan (2022) -Shenzhen, China	0.0908	0.0718	1.09	[0.95; 1.26]	1.5%
Benjamin (2018) -Nha Trang, central Vietnam	-0.1625	0.0465	0.85	[0.78; 0.93]	1.5%
Ji (2011) -Suzhou, China	-0.5504	0.5547	0.58	[0.19; 1.71]	1.4%
Pan (2019) -Panzhihua, China	-0.3698	0.3375	0.69	[0.36; 1.34]	1.5%
Pan (2019) -Panzhihua, China	-1.4494	0.3617	0.23	[0.12; 0.48]	1.4%
Zheng (2014) -Jiangsu, China	-0.2801	0.3207	0.76	[0.40; 1.42]	1.5%
Nicklas (2016) -Gothenburg, Sweden	-6.1193	0.6078	0.00	[0.00; 0.01]	1.3%
Jean-Baptist (2009) -Rheinland-Pfalz, Mainz	-1.2680	0.2190	0.28	[0.18; 0.43]	1.5%
Cheng (2022) -Macao, China	-0.0617	0.0951	0.94	[0.78; 1.13]	1.5%
Li (2020) -Shanghai, China	-1.8793	0.4090	0.15	[0.07; 0.34]	1.4%
Li (2020) -Shanghai, China	-1.9317	0.4117	0.14	[0.06; 0.32]	1.4%
Li (2020) -Shanghai, China	-1.7277	0.4025	0.18	[0.08; 0.39]	1.4%
Rory (2019) -Edinburgh, UK	-0.1393	0.0138	0.87	[0.85; 0.89]	1.5%
Maria (2009) -Kathmandu, Nepal	-1.1829	0.6668	0.31	[0.08; 1.13]	1.3%
Li (2013) -Zhuhai, China	0.0508	1.2972	1.05	[0.08; 13.37]	1.0%
Adriana (2018) -Toronto, Canada	-0.0619	0.0081	0.94	[0.93; 0.96]	1.5%
Adriana (2018) -Toronto, Canada	-1.8282	0.2317	0.16	[0.10; 0.25]	1.5%
Wei (2019) -Jinan, China	-1.8282	0.2845	0.16	[0.09; 0.28]	1.5%
Gwladys (2017) -Yaounde, Cameroon	-0.1744	0.1926	0.84	[0.58; 1.23]	1.5%
Mariana (2004) -Buenos Aires, Argentina	-2.1507	0.5646	0.12	[0.04; 0.35]	1.4%
Pan (2022) -Shenzhen, China	-0.6182	0.0728	0.54	[0.47; 0.62]	1.5%
Pan (2022) -Shenzhen, China	-0.6259	0.0729	0.53	[0.46; 0.62]	1.5%
Pan (2022) -Shenzhen, China	-0.5766	0.0727	0.56	[0.49; 0.65]	1.5%
Pan (2019) -Panzhihua, China	0.2764	0.3367	1.32	[0.68; 2.55]	1.5%
Pan (2019) -Panzhihua, China	-0.4008	0.3378	0.67	[0.35; 1.30]	1.5%
Ji (2011) -Suzhou, China	0.4756	0.5532	1.61	[0.54; 4.76]	1.4%
You (2022) -Gwangju, Korea	-3.6156	0.7767	0.03	[0.01; 0.12]	1.3%
Adriana (2018) -Toronto, Canada	-0.0726	0.0055	0.93	[0.92; 0.94]	1.5%
Adriana (2018) -Toronto, Canada	-1.1829	0.2175	0.31	[0.20; 0.47]	1.5%
Wei (2016) -Shanghai, China	-1.9421	0.6791	0.14	[0.04; 0.54]	1.3%
Gwladys (2017) -Yaounde, Cameroon	-0.3425	0.1932	0.71	[0.49; 1.04]	1.5%
Lin (2015) -Quanzhou, China	0.1222	1.3027	1.13	[0.09; 14.52]	1.0%
Lin (2015) -Quanzhou, China	0.8879	1.3427	2.43	[0.17; 33.77]	0.9%
Chen (2016) -Suzhou, China	-0.6259	0.8280	0.53	[0.11; 2.71]	1.2%
Chen (2016) -Suzhou, China	-0.7404	0.8329	0.48	[0.09; 2.44]	1.2%
Zhang (2022) -Jiangyin, China	-1.0458	0.4587	0.35	[0.14; 0.86]	1.4%
Zhang (2022) -Jiangyin, China	-1.2595	0.4665	0.28	[0.11; 0.71]	1.4%
Cao (2010) -Beijing, China	-10.4143	0.7642	0.00	[0.00; 0.00]	1.3%
Can (2021) -China	-0.0619	0.0243	0.94	[0.90; 0.99]	1.5%
Can (2021) -Northern China	-0.0726	0.0275	0.93	[0.88; 0.98]	1.5%
Can (2021) -Southern China	-0.0408	0.0291	0.96	[0.91; 1.02]	1.5%
Can (2021) -China	-0.0305	0.0263	0.97	[0.92; 1.02]	1.5%
Can (2021) -Northern China	-0.0408	0.0266	0.96	[0.91; 1.01]	1.5%
Can (2021) -Southern China	-0.0101	0.0282	0.99	[0.94; 1.05]	1.5%
Can (2021) -China	-0.0305	0.0183	0.97	[0.94; 1.01]	1.5%
Can (2021) -Northern China	-0.0408	0.0187	0.96	[0.93; 1.00]	1.5%
Can (2021) -Southern China	-0.0305	0.0211	0.97	[0.93; 1.01]	1.5%
Random effects model			0.35	[0.24; 0.52]	95.5%
Prediction interval				[0.02; 8.08]	
Heterogeneity: $I^2 = 95\%$, $\tau^2 = 2.4304$, $p < 0.01$					
**Lag = >1**					
Annabel (2023) -Bukit Timah, Singapore	-0.0523	0.0180	0.95	[0.92; 0.98]	1.5%
Li (2020) -Shanghai, China	3.3925	0.2460	29.74	[18.36; 48.16]	1.5%
Li (2020) -Shanghai, China	3.1001	0.3334	22.20	[11.55; 42.67]	1.5%
Random effects model			8.42	[0.95; 74.40]	4.5%
Prediction interval				[0.00; 13327175986419.18]	
Heterogeneity: $I^2 = 99\%$, $\tau^2 = 3.6514$, $p < 0.01$					
**Random effects model**			**0.40**	**[0.27; 0.61]**	**100.0%**
**Prediction interval**				**[0.01; 12.69]**	

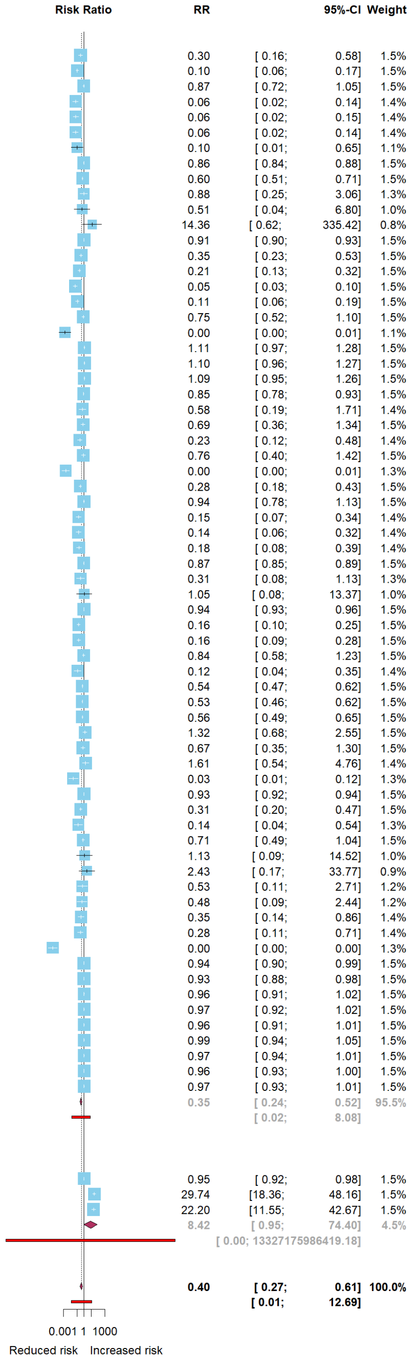

Heterogeneity: $I^2 = 96\%$, $\tau^2 = 2.9513$, $p < 0.01$
Test for subgroup differences: $\chi^2_1 = 7.92$, df = 1 ($p < 0.01$)

Classified as ECDC NORMAL

Figure S2-7. Leave-one-out analysis of influenza virus (IV) meta-analysis

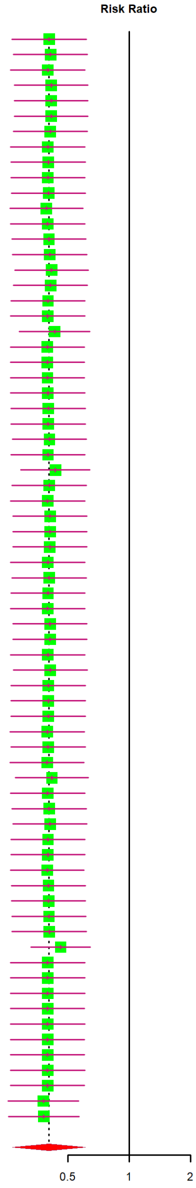

Study	RR	95%-CI	P-value	Tau2	Tau	I2
Omitting Zheng (2014) -Jiangsu, China	0.40	[0.26; 0.61]	< 0.01	3.0037	1.7331	96%
Omitting Jean-Baptist (2009) -Rheinland-Pfalz, Mainz	0.41	[0.27; 0.62]	< 0.01	2.9708	1.7236	95%
Omitting Cheng (2022) -Macao, China	0.40	[0.26; 0.60]	< 0.01	2.9968	1.7311	96%
Omitting Li (2020) -Shanghai, China	0.41	[0.27; 0.63]	< 0.01	2.9374	1.7139	96%
Omitting Li (2020) -Shanghai, China	0.41	[0.27; 0.63]	< 0.01	2.9422	1.7153	96%
Omitting Li (2020) -Shanghai, China	0.41	[0.27; 0.63]	< 0.01	2.9374	1.7139	96%
Omitting Chen (2014) -Suzhou, China	0.41	[0.27; 0.62]	< 0.01	2.9707	1.7236	96%
Omitting Rory (2019) -Edinburgh, UK	0.40	[0.26; 0.60]	< 0.01	2.9971	1.7312	96%
Omitting Cui (2015) -Chaoshan, China	0.40	[0.26; 0.61]	< 0.01	3.0048	1.7334	96%
Omitting Maria (2009) -Kathmandu, Nepal	0.40	[0.26; 0.60]	< 0.01	2.9924	1.7299	96%
Omitting Li (2013) -Zhuhai, China	0.40	[0.26; 0.61]	< 0.01	2.9866	1.7282	96%
Omitting Li (2013) -Zhuhai, China	0.39	[0.26; 0.59]	< 0.01	2.9150	1.7073	96%
Omitting Adriana (2018) -Toronto, Canada	0.40	[0.26; 0.60]	< 0.01	2.9956	1.7308	96%
Omitting Adriana (2018) -Toronto, Canada	0.40	[0.26; 0.61]	< 0.01	3.0062	1.7338	96%
Omitting Adriana (2018) -Toronto, Canada	0.41	[0.27; 0.62]	< 0.01	2.9975	1.7313	96%
Omitting Wei (2019) -Jinan, China	0.41	[0.27; 0.63]	< 0.01	2.9277	1.7110	95%
Omitting Wei (2019) -Jinan, China	0.41	[0.27; 0.62]	< 0.01	2.9733	1.7243	96%
Omitting Gwladys (2017) -Yaounde, Cameroon	0.40	[0.26; 0.61]	< 0.01	3.0002	1.7321	96%
Omitting Annabel (2023) -Bukit Timah, Singapore	0.40	[0.26; 0.60]	< 0.01	2.9943	1.7304	96%
Omitting Mariana (2004) -Buenos Aires, Argentina	0.43	[0.29; 0.64]	< 0.01	2.6690	1.6337	96%
Omitting Pan (2022) -Shenzhen, China	0.40	[0.26; 0.60]	< 0.01	2.9887	1.7288	96%
Omitting Pan (2022) -Shenzhen, China	0.40	[0.26; 0.60]	< 0.01	2.9890	1.7289	96%
Omitting Pan (2022) -Shenzhen, China	0.40	[0.26; 0.60]	< 0.01	2.9892	1.7289	96%
Omitting Benjamin (2018) -Nha Trang, central Vietnam	0.40	[0.26; 0.60]	< 0.01	2.9975	1.7313	96%
Omitting Ji (2011) -Suzhou, China	0.40	[0.26; 0.61]	< 0.01	3.0007	1.7322	96%
Omitting Pan (2019) -Panzhihua, China	0.40	[0.26; 0.61]	< 0.01	3.0009	1.7323	96%
Omitting Pan (2019) -Panzhihua, China	0.40	[0.27; 0.62]	< 0.01	2.9995	1.7319	96%
Omitting Zheng (2014) -Jiangsu, China	0.40	[0.26; 0.61]	< 0.01	2.9992	1.7318	96%
Omitting Nicklas (2016) -Gothenburg, Sweden	0.43	[0.29; 0.64]	< 0.01	2.5804	1.6063	95%
Omitting Jean-Baptist (2009) -Rheinland-Pfalz, Mainz	0.40	[0.26; 0.61]	< 0.01	3.0039	1.7332	96%
Omitting Cheng (2022) -Macao, China	0.40	[0.26; 0.60]	< 0.01	2.9945	1.7305	96%
Omitting Li (2020) -Shanghai, China	0.41	[0.27; 0.62]	< 0.01	2.9874	1.7284	96%
Omitting Li (2020) -Shanghai, China	0.41	[0.27; 0.62]	< 0.01	2.9855	1.7279	96%
Omitting Li (2020) -Shanghai, China	0.41	[0.27; 0.62]	< 0.01	2.9923	1.7298	96%
Omitting Rory (2019) -Edinburgh, UK	0.40	[0.26; 0.60]	< 0.01	2.9969	1.7312	96%
Omitting Maria (2009) -Kathmandu, Nepal	0.40	[0.26; 0.61]	< 0.01	2.9990	1.7318	96%
Omitting Li (2013) -Zhuhai, China	0.40	[0.26; 0.60]	< 0.01	2.9808	1.7265	96%
Omitting Adriana (2018) -Toronto, Canada	0.40	[0.26; 0.60]	< 0.01	2.9946	1.7305	96%
Omitting Adriana (2018) -Toronto, Canada	0.41	[0.27; 0.62]	< 0.01	2.9899	1.7291	96%
Omitting Wei (2019) -Jinan, China	0.41	[0.27; 0.62]	< 0.01	2.9897	1.7291	96%
Omitting Gwladys (2017) -Yaounde, Cameroon	0.40	[0.26; 0.60]	< 0.01	2.9975	1.7313	96%
Omitting Mariana (2004) -Buenos Aires, Argentina	0.41	[0.27; 0.62]	< 0.01	2.9768	1.7253	96%
Omitting Pan (2022) -Shenzhen, China	0.40	[0.26; 0.61]	< 0.01	3.0062	1.7338	96%
Omitting Pan (2022) -Shenzhen, China	0.40	[0.26; 0.61]	< 0.01	3.0062	1.7338	96%
Omitting Pan (2022) -Shenzhen, China	0.40	[0.26; 0.61]	< 0.01	3.0057	1.7337	96%
Omitting Pan (2019) -Panzhihua, China	0.39	[0.26; 0.60]	< 0.01	2.9815	1.7267	96%
Omitting Pan (2019) -Panzhihua, China	0.40	[0.26; 0.61]	< 0.01	3.0014	1.7325	96%
Omitting Ji (2011) -Suzhou, China	0.39	[0.26; 0.60]	< 0.01	2.9733	1.7243	96%
Omitting You (2022) -Gwangju, Korea	0.42	[0.28; 0.63]	< 0.01	2.8984	1.7025	96%
Omitting Adriana (2018) -Toronto, Canada	0.40	[0.26; 0.60]	< 0.01	2.9949	1.7306	96%
Omitting Adriana (2018) -Toronto, Canada	0.40	[0.26; 0.61]	< 0.01	3.0050	1.7335	96%
Omitting Wei (2016) -Shanghai, China	0.41	[0.27; 0.62]	< 0.01	2.9837	1.7274	96%
Omitting Gwladys (2017) -Yaounde, Cameroon	0.40	[0.26; 0.61]	< 0.01	3.0014	1.7325	96%
Omitting Lin (2015) -Quanzhou, China	0.40	[0.26; 0.60]	< 0.01	2.9797	1.7262	96%
Omitting Lin (2015) -Quanzhou, China	0.39	[0.26; 0.60]	< 0.01	2.9637	1.7216	96%
Omitting Chen (2016) -Suzhou, China	0.40	[0.26; 0.61]	< 0.01	2.9963	1.7310	96%
Omitting Chen (2016) -Suzhou, China	0.40	[0.26; 0.61]	< 0.01	2.9968	1.7311	96%
Omitting Zhang (2022) -Jiangyin, China	0.40	[0.26; 0.61]	< 0.01	3.0035	1.7331	96%
Omitting Zhang (2022) -Jiangyin, China	0.40	[0.26; 0.61]	< 0.01	3.0014	1.7325	96%
Omitting Cao (2010) -Beijing, China	0.46	[0.33; 0.64]	< 0.01	1.8413	1.3570	95%
Omitting Can (2021) -China	0.40	[0.26; 0.60]	< 0.01	2.9946	1.7305	96%
Omitting Can (2021) -Northern China	0.40	[0.26; 0.60]	< 0.01	2.9949	1.7306	96%
Omitting Can (2021) -Southern China	0.40	[0.26; 0.60]	< 0.01	2.9939	1.7303	96%
Omitting Can (2021) -China	0.40	[0.26; 0.60]	< 0.01	2.9936	1.7302	96%
Omitting Can (2021) -Northern China	0.40	[0.26; 0.60]	< 0.01	2.9939	1.7303	96%
Omitting Can (2021) -Southern China	0.40	[0.26; 0.60]	< 0.01	2.9929	1.7300	96%
Omitting Can (2021) -China	0.40	[0.26; 0.60]	< 0.01	2.9936	1.7302	96%
Omitting Can (2021) -Northern China	0.40	[0.26; 0.60]	< 0.01	2.9939	1.7303	96%
Omitting Can (2021) -Southern China	0.40	[0.26; 0.60]	< 0.01	2.9936	1.7302	96%
Omitting Li (2020) -Shanghai, China	0.38	[0.25; 0.56]	< 0.01	2.6609	1.6312	95%
Omitting Li (2020) -Shanghai, China	0.38	[0.25; 0.57]	< 0.01	2.7176	1.6485	95%
**Random effects model**	**0.40**	**[0.27; 0.61]**	**< 0.01**	**2.9513**	**1.7179**	**96%**

Classified as ECDC NORMAL

Figure S2-8. Sensitivity analysis by switching the relative risk for cold effects for Li et al 2020

Study	logRR	SE(logRR)	RR	95%-CI	Weight
**Influenza virus A**					
Mariana (2004) -Buenos Aires, Argentina	-6.3771	0.9767	0.00	[0.00; 0.01]	1.1%
Li (2020) -Shanghai, China	-2.8665	0.4635	0.06	[0.02; 0.14]	1.4%
Li (2020) -Shanghai, China	-2.8665	0.4635	0.06	[0.02; 0.14]	1.4%
Li (2020) -Shanghai, China	-2.7920	0.4580	0.06	[0.02; 0.15]	1.4%
Chen (2014) -Suzhou, China	-2.3279	0.9684	0.10	[0.01; 0.65]	1.1%
Jean-Baptist (2009) -Rheinland-Pfalz, Mainz	-2.2672	0.2439	0.10	[0.06; 0.17]	1.5%
Zheng (2014) -Jiangsu, China	-1.2040	0.3368	0.30	[0.16; 0.58]	1.5%
Adriana (2018) -Toronto, Canada	-1.0581	0.2154	0.35	[0.23; 0.53]	1.5%
Zhang (2022) -Jiangyin, China	-1.0458	0.4587	0.35	[0.14; 0.86]	1.4%
Chen (2016) -Suzhou, China	-0.6259	0.8280	0.53	[0.11; 2.71]	1.2%
Ji (2011) -Suzhou, China	-0.5504	0.5547	0.58	[0.19; 1.71]	1.4%
Cui (2015) -Chaoshan, China	-0.5108	0.0858	0.60	[0.51; 0.71]	1.5%
Gwladys (2017) -Yaounde, Cameroon	-0.2838	0.1929	0.75	[0.52; 1.10]	1.5%
Benjamin (2018) -Nha Trang, central Vietnam	-0.1625	0.0465	0.85	[0.78; 0.93]	1.5%
Rory (2019) -Edinburgh, UK	-0.1473	0.0112	0.86	[0.84; 0.88]	1.5%
Cheng (2022) -Macao, China	-0.1380	0.0951	0.87	[0.72; 1.05]	1.5%
Maria (2009) -Kathmandu, Nepal	-0.1234	0.6343	0.88	[0.25; 3.06]	1.3%
Adriana (2018) -Toronto, Canada	-0.0943	0.0084	0.91	[0.90; 0.93]	1.5%
Annabel (2023) -Bukit Timah, Singapore	-0.0523	0.0180	0.95	[0.92; 0.98]	1.5%
Lin (2015) -Quanzhou, China	0.1222	1.3027	1.13	[0.09; 14.52]	1.0%
Li (2020) -Shanghai, China	2.3561	0.1538	10.55	[7.80; 14.26]	1.5%
Random effects model			0.36	[0.18; 0.72]	29.3%
Prediction interval				[0.01; 9.68]	
Heterogeneity: $I^2 = 96\%$, $\tau^2 = 2.3466$, $p < 0.01$					
**Influenza virus A/H1N1pdm09**					
Cao (2010) -Beijing, China	-10.4143	0.7642	0.00	[0.00; 0.00]	1.3%
Wei (2019) -Jinan, China	-2.9584	0.3284	0.05	[0.03; 0.10]	1.5%
Pan (2019) -Panzhihua, China	-1.4494	0.3617	0.23	[0.12; 0.48]	1.4%
Li (2013) -Zhuhai, China	-0.6677	1.3186	0.51	[0.04; 6.80]	0.9%
Can (2021) -Northern China	-0.0726	0.0275	0.93	[0.88; 0.98]	1.5%
Can (2021) -China	-0.0619	0.0243	0.94	[0.90; 0.99]	1.5%
Can (2021) -Southern China	-0.0408	0.0291	0.96	[0.91; 1.02]	1.5%
Pan (2022) -Shenzhen, China	0.0908	0.0718	1.09	[0.95; 1.26]	1.5%
Pan (2022) -Shenzhen, China	0.0979	0.0719	1.10	[0.96; 1.27]	1.5%
Pan (2022) -Shenzhen, China	0.1053	0.0719	1.11	[0.97; 1.28]	1.5%
Random effects model			0.22	[0.03; 1.61]	14.2%
Prediction interval				[0.00; 461.07]	
Heterogeneity: $I^2 = 97\%$, $\tau^2 = 9.9374$, $p < 0.01$					
**Influenza virus A/H3N2**					
Wei (2019) -Jinan, China	-2.2201	0.2980	0.11	[0.06; 0.19]	1.5%
Adriana (2018) -Toronto, Canada	-1.5833	0.2257	0.21	[0.13; 0.32]	1.5%
Pan (2019) -Panzhihua, China	-0.3698	0.3375	0.69	[0.36; 1.34]	1.5%
Can (2021) -Northern China	-0.0408	0.0266	0.96	[0.91; 1.01]	1.5%
Can (2021) -China	-0.0305	0.0263	0.97	[0.92; 1.02]	1.5%
Can (2021) -Southern China	-0.0101	0.0282	0.99	[0.94; 1.05]	1.5%
Li (2013) -Zhuhai, China	2.6646	1.6075	14.36	[0.62; 335.42]	0.8%
Random effects model			0.59	[0.27; 1.28]	9.8%
Prediction interval				[0.04; 8.97]	
Heterogeneity: $I^2 = 94\%$, $\tau^2 = 0.9660$, $p < 0.01$					
**Influenza virus B**					
Nicklas (2016) -Gothenburg, Sweden	-6.1193	0.6078	0.00	[0.00; 0.01]	1.3%
Mariana (2004) -Buenos Aires, Argentina	-2.1507	0.5646	0.12	[0.04; 0.35]	1.4%
Li (2020) -Shanghai, China	-1.9317	0.4117	0.14	[0.06; 0.32]	1.4%
Li (2020) -Shanghai, China	-1.8793	0.4090	0.15	[0.07; 0.34]	1.4%
Adriana (2018) -Toronto, Canada	-1.8282	0.2317	0.16	[0.10; 0.25]	1.5%
Wei (2019) -Jinan, China	-1.8282	0.2845	0.16	[0.09; 0.28]	1.5%
Li (2020) -Shanghai, China	-1.7277	0.4025	0.18	[0.08; 0.39]	1.4%
Jean-Baptist (2009) -Rheinland-Pfalz, Mainz	-1.2680	0.2190	0.28	[0.18; 0.43]	1.5%
Zhang (2022) -Jiangyin, China	-1.2595	0.4665	0.28	[0.11; 0.71]	1.4%
Maria (2009) -Kathmandu, Nepal	-1.1829	0.6668	0.31	[0.08; 1.13]	1.3%
Chen (2016) -Suzhou, China	-0.7404	0.8329	0.48	[0.09; 2.44]	1.2%
Pan (2022) -Shenzhen, China	-0.6259	0.0729	0.53	[0.46; 0.62]	1.5%
Pan (2022) -Shenzhen, China	-0.6182	0.0728	0.54	[0.47; 0.62]	1.5%
Pan (2022) -Shenzhen, China	-0.5766	0.0727	0.56	[0.49; 0.65]	1.5%
Zheng (2014) -Jiangsu, China	-0.2801	0.3207	0.76	[0.40; 1.42]	1.5%
Gwladys (2017) -Yaounde, Cameroon	-0.1744	0.1926	0.84	[0.58; 1.23]	1.5%
Rory (2019) -Edinburgh, UK	-0.1393	0.0138	0.87	[0.85; 0.89]	1.5%
Adriana (2018) -Toronto, Canada	-0.0619	0.0081	0.94	[0.93; 0.96]	1.5%
Cheng (2022) -Macao, China	-0.0617	0.0951	0.94	[0.78; 1.13]	1.5%
Can (2021) -Northern China	-0.0408	0.0187	0.96	[0.93; 1.00]	1.5%
Can (2021) -China	-0.0305	0.0183	0.97	[0.94; 1.01]	1.5%
Can (2021) -Southern China	-0.0305	0.0211	0.97	[0.93; 1.01]	1.5%
Li (2013) -Zhuhai, China	0.0508	1.2972	1.05	[0.08; 13.37]	1.0%
Ji (2011) -Suzhou, China	0.4756	0.5532	1.61	[0.54; 4.76]	1.4%
Lin (2015) -Quanzhou, China	0.8879	1.3427	2.43	[0.17; 33.77]	0.9%
Li (2020) -Shanghai, China	3.3925	0.2460	29.74	[18.36; 48.16]	1.5%
Random effects model			0.47	[0.26; 0.85]	36.7%
Prediction interval				[0.02; 10.65]	
Heterogeneity: $I^2 = 96\%$, $\tau^2 = 2.2022$, $p < 0.01$					
**Influenza virus B/Victoria**					
Pan (2019) -Panzhihua, China	0.2764	0.3367	1.32	[0.68; 2.55]	1.5%
Prediction interval					
**Influenza virus B/Yamagata**					
Pan (2019) -Panzhihua, China	-0.4008	0.3378	0.67	[0.35; 1.30]	1.5%
Prediction interval					
**Influenza virus -unsubtyped**					
You (2022) -Gwangju, Korea	-3.6156	0.7767	0.03	[0.01; 0.12]	1.3%
Wei (2016) -Shanghai, China	-1.9421	0.6791	0.14	[0.04; 0.54]	1.3%
Adriana (2018) -Toronto, Canada	-1.1829	0.2175	0.31	[0.20; 0.47]	1.5%
Gwladys (2017) -Yaounde, Cameroon	-0.3425	0.1932	0.71	[0.49; 1.04]	1.5%
Adriana (2018) -Toronto, Canada	-0.0726	0.0055	0.93	[0.92; 0.94]	1.5%
Random effects model			0.28	[0.09; 0.88]	7.1%
Prediction interval				[0.00; 21.45]	
Heterogeneity: $I^2 = 93\%$, $\tau^2 = 1.5151$, $p < 0.01$					
**Random effects model**			**0.40**	**[0.26; 0.60]**	**100.0%**
**Prediction interval**				**[0.01; 11.97]**	

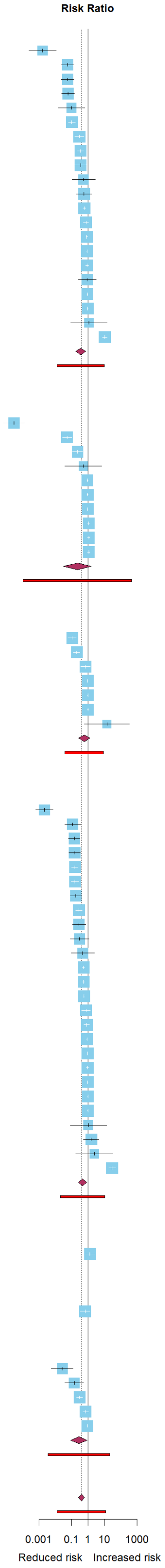

Heterogeneity: $I^2 = 96\%$, $\tau^2 = 2.8670$, $p < 0.01$
Test for subgroup differences: $\chi^2_6 = 10.89$, df = 6 ($p = 0.09$)

Classified as ECDC NORMAL

Figure S2-9. Funnel plot of influenza virus (IV) estimates

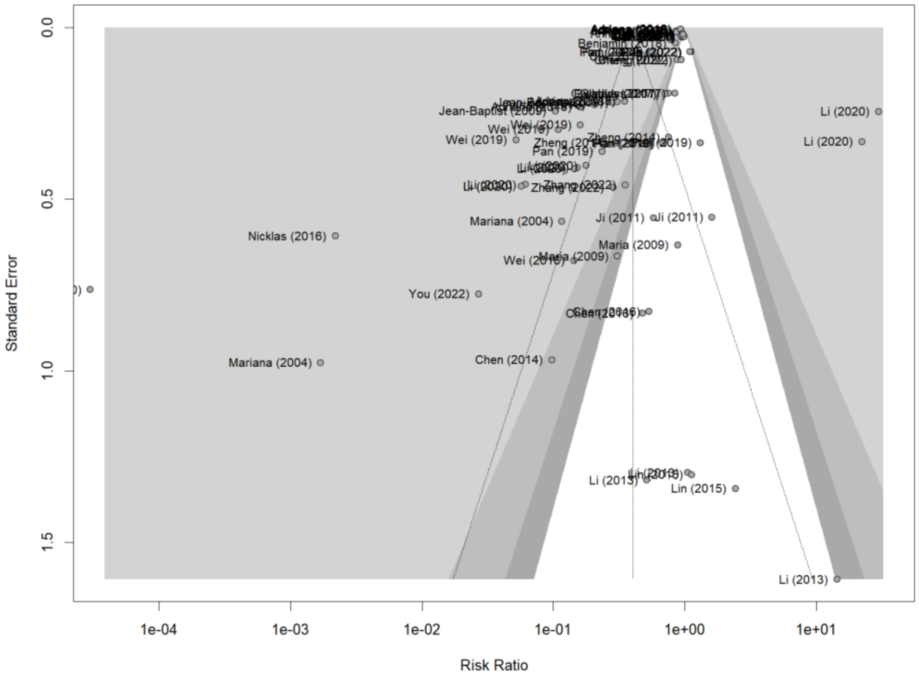

Linear regression test of funnel plot asymmetry

Egger's test result: t = -4.29, df = 69, p-value < 0.0001

Sample estimates:

bias	se.bias	intercept	se.intercept
-2.4900	0.5804	-0.0516	0.0156

Figure S2-10. Trim and fill of influenza virus (IV) meta-analysis

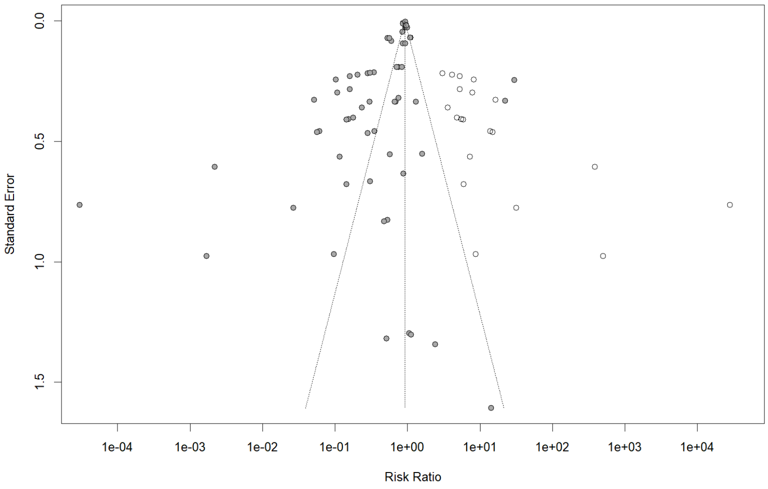

Number of studies: k = 92 (with 21 added studies)
Random effects model: RR= 0.9162 [0.5580; 1.5041], Z= -0.35, P=0.7292
Quantifying heterogeneity:
tau^2 = 5.6364 [4.3703; 8.2222]; tau = 2.3741 [2.0905; 2.8674]
I^2 = 96.4% [96.0%; 96.8%]; H = 5.27 [4.98; 5.58]

Classified as ECDC NORMAL

Figure S3. Random-effects meta-analysis of human parainfluenza virus (HPIV) estimates (23 studies)

Study	logRR	SE(logRR)	RR	95%-CI	Weight
**Parainfluenza virus -1**					
Jean-Baptist (2009) -Rheinland-Pfalz, Mainz	-0.1816	0.2071	0.83	[0.56; 1.25]	3.1%
Ji (2011) -Suzhou, China	-0.0362	0.5485	0.96	[0.33; 2.83]	2.7%
Zheng (2014) -Jiangsu, China	0.2290	0.3204	1.26	[0.67; 2.36]	3.0%
Eun (2021) -Cheonan, Korea	0.2624	0.3732	1.30	[0.63; 2.70]	3.0%
Chen (2016) -Suzhou, China	0.5504	0.8253	1.73	[0.34; 8.74]	2.3%
Maria (2009) -Kathmandu, Nepal	0.6258	0.6433	1.87	[0.53; 6.60]	2.6%
Random effects model			1.06	[0.78; 1.42]	16.8%
Prediction interval				[0.66; 1.69]	
Heterogeneity: $I^2 = 0\%$, $\tau^2 = 0.0056$, $p = 0.69$					
**Parainfluenza virus -2**					
Eun (2021) -Cheonan, Korea	-1.3280	0.6948	0.26	[0.07; 1.03]	2.5%
Zheng (2014) -Jiangsu, China	0.0798	0.3198	1.08	[0.58; 2.03]	3.0%
Chen (2016) -Suzhou, China	0.8181	0.8364	2.27	[0.44; 11.68]	2.3%
Ji (2011) -Suzhou, China	1.0373	0.5518	2.82	[0.96; 8.32]	2.7%
Maria (2009) -Kathmandu, Nepal	1.5347	0.6883	4.64	[1.20; 17.88]	2.5%
Random effects model			1.50	[0.60; 3.75]	13.2%
Prediction interval				[0.07; 32.31]	
Heterogeneity: $I^2 = 65\%$, $\tau^2 = 0.7113$, $p = 0.02$					
**Parainfluenza virus -3**					
Cui (2015) -Chaoshan, China	-1.2040	0.1034	0.30	[0.24; 0.37]	3.2%
Jean-Baptist (2009) -Rheinland-Pfalz, Mainz	-0.6259	0.2098	0.53	[0.35; 0.81]	3.1%
Eun (2021) -Cheonan, Korea	0.5400	0.5017	1.72	[0.64; 4.59]	2.8%
Zheng (2014) -Jiangsu, China	1.9158	0.3616	6.79	[3.34; 13.80]	3.0%
Chen (2014) -Suzhou, China	2.2084	0.9553	9.10	[1.40; 59.19]	2.1%
Zhang (2022) -Jiangyin, China	2.2495	0.0385	9.48	[8.79; 10.23]	3.2%
Ji (2011) -Suzhou, China	2.2673	0.6468	9.65	[2.72; 34.30]	2.6%
Sun (2011) -Suzhou, China	2.2673	0.0525	9.65	[8.71; 10.70]	3.2%
Chen (2016) -Suzhou, China	2.7926	0.0756	16.32	[14.08; 18.93]	3.2%
Ming (2022) -Wenzhou, China	2.9508	0.4328	19.12	[8.19; 44.66]	2.9%
Maria (2009) -Kathmandu, Nepal	3.1028	0.8342	22.26	[4.34; 114.17]	2.3%
Zhang (2020) -Suzhou, China	3.3278	0.0568	27.88	[24.94; 31.16]	3.2%
Random effects model			5.92	[2.48; 14.13]	34.8%
Prediction interval				[0.19; 182.55]	
Heterogeneity: $I^2 = 99\%$, $\tau^2 = 2.1701$, $p = 0$					
**Parainfluenza virus -4**					
Cui (2015) -Chaoshan, China	0.0953	0.0669	1.10	[0.96; 1.25]	3.2%
Prediction interval					
**Parainfluenza viruses -unsubtyped**					
Wei (2016) -Shanghai, China	-2.3581	0.7139	0.09	[0.02; 0.38]	2.5%
Mariana (2004) -Buenos Aires, Argentina	-1.3116	0.5167	0.27	[0.10; 0.74]	2.8%
Li (2013) -Zhuhai, China	-0.7520	1.3243	0.47	[0.04; 6.32]	1.6%
Stacy (2021) -Singapore	-0.0587	0.0219	0.94	[0.90; 0.98]	3.2%
Cheng (2022) -Macao, China	0.0108	0.0951	1.01	[0.84; 1.22]	3.2%
Therese (2021) -Nairobi, Kenya	0.1044	0.1493	1.11	[0.83; 1.49]	3.2%
Therese (2021) -Nairobi, Kenya	0.1398	0.1377	1.15	[0.88; 1.51]	3.2%
Lin (2015) -Quanzhou, China	0.6152	1.3179	1.85	[0.14; 24.49]	1.6%
Liu (2019) -Guangzhou, China	1.2595	0.4263	3.52	[1.53; 8.13]	2.9%
You (2022) -Gwangju, Korea	1.9475	0.6225	7.01	[2.07; 23.75]	2.6%
Yan (2015) -Shanghai, China	2.7066	0.0901	14.98	[12.55; 17.87]	3.2%
Huang (2019) -Zhongshan, China	2.8293	1.0348	16.93	[2.23; 128.69]	2.0%
Random effects model			1.55	[0.65; 3.66]	32.0%
Prediction interval				[0.06; 40.24]	
Heterogeneity: $I^2 = 99\%$, $\tau^2 = 1.9466$, $p < 0.01$					
**Random effects model**			**2.35**	**[1.46; 3.77]**	**100.0%**
**Prediction interval**				**[0.14; 38.03]**	

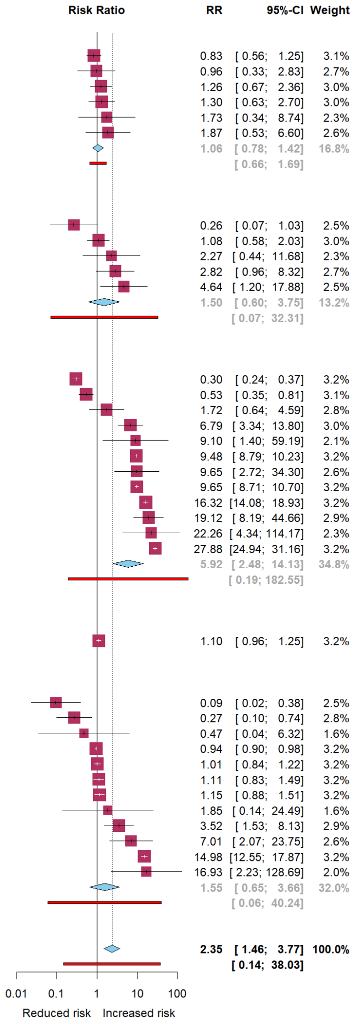

Heterogeneity: $I^2 = 100\%$, $\tau^2 = 1.8204$, $p = 0$
Test for subgroup differences: $\chi^2_4 = 15.19$, df = 4 ($p < 0.01$)

Classified as ECDC NORMAL

Figure S3-1. Subgroup analysis of human parainfluenza virus (HPIV) meta-analysis by Köppen-Geiger climate

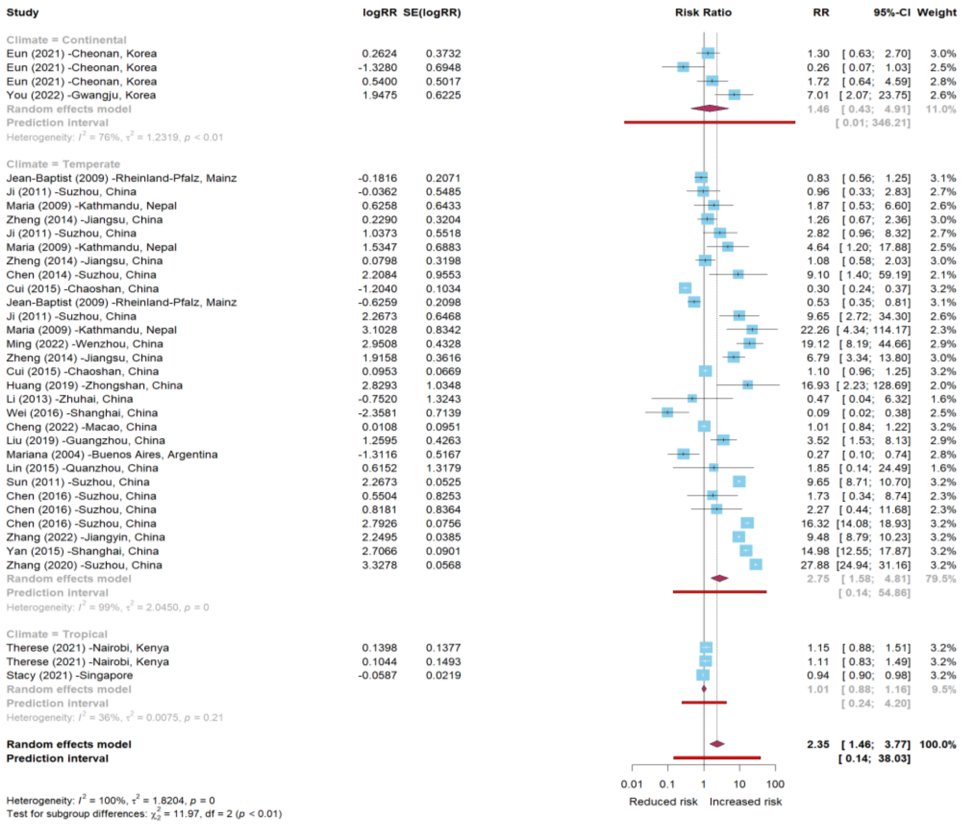

Figure S3-2. Subgroup analysis of human parainfluenza virus (HPIV) meta-analysis by World Bank income category

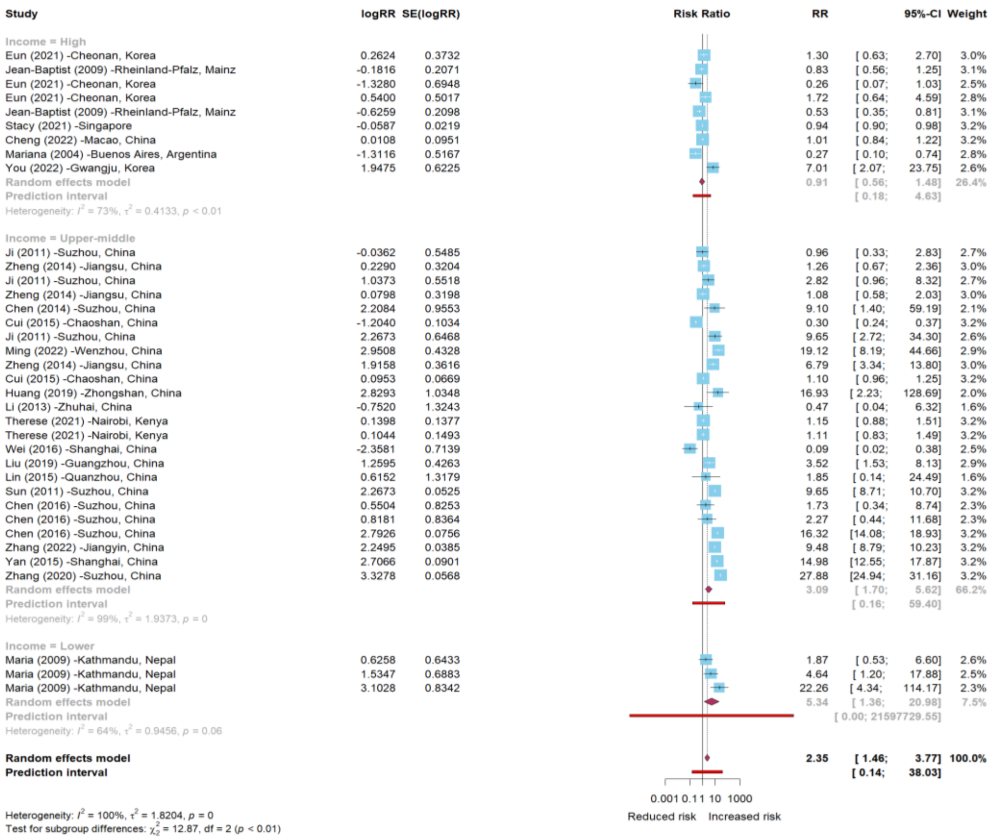

Classified as ECDC NORMAL

Figure S3-3. Subgroup analysis of human parainfluenza virus (HPIV) meta-analysis by temporal resolution

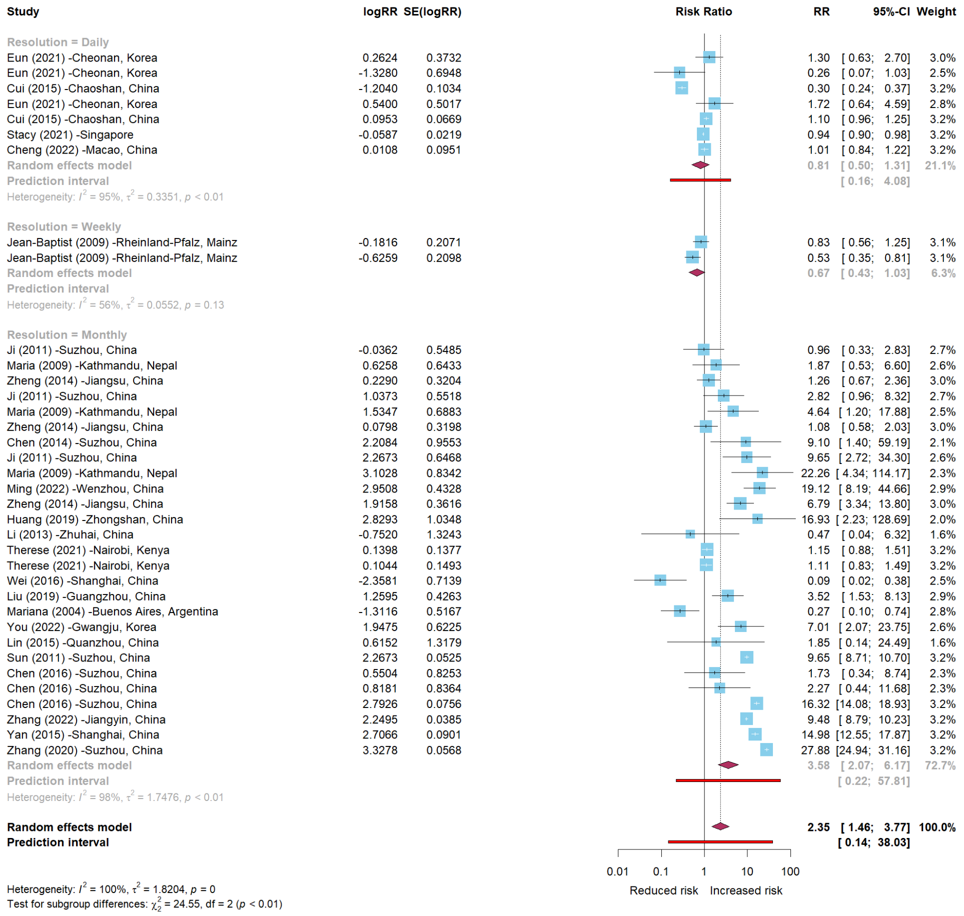

Classified as ECDC NORMAL

Figure S3-4. Subgroup analysis of human parainfluenza virus (HPIV) meta-analysis by exposure measure

Study	logRR	SE(logRR)	RR	95%-CI	Weight
**Temperature = Mean**					
Eun (2021) -Cheonan, Korea	0.2624	0.3732	1.30	[0.63; 2.70]	3.0%
Jean-Baptist (2009) -Rheinland-Pfalz, Mainz	-0.1816	0.2071	0.83	[0.56; 1.25]	3.1%
Ji (2011) -Suzhou, China	-0.0362	0.5485	0.96	[0.33; 2.83]	2.7%
Maria (2009) -Kathmandu, Nepal	0.6258	0.6433	1.87	[0.53; 6.60]	2.6%
Zheng (2014) -Jiangsu, China	0.2290	0.3204	1.26	[0.67; 2.36]	3.0%
Eun (2021) -Cheonan, Korea	-1.3280	0.6948	0.26	[0.07; 1.03]	2.5%
Ji (2011) -Suzhou, China	1.0373	0.5518	2.82	[0.96; 8.32]	2.7%
Maria (2009) -Kathmandu, Nepal	1.5347	0.6883	4.64	[1.20; 17.88]	2.5%
Zheng (2014) -Jiangsu, China	0.0798	0.3198	1.08	[0.58; 2.03]	3.0%
Chen (2014) -Suzhou, China	2.2084	0.9553	9.10	[1.40; 59.19]	2.1%
Cui (2015) -Chaoshan, China	-1.2040	0.1034	0.30	[0.24; 0.37]	3.2%
Eun (2021) -Cheonan, Korea	0.5400	0.5017	1.72	[0.64; 4.59]	2.8%
Jean-Baptist (2009) -Rheinland-Pfalz, Mainz	-0.6259	0.2098	0.53	[0.35; 0.81]	3.1%
Ji (2011) -Suzhou, China	2.2673	0.6468	9.65	[2.72; 34.30]	2.6%
Maria (2009) -Kathmandu, Nepal	3.1028	0.8342	22.26	[4.34; 114.17]	2.3%
Ming (2022) -Wenzhou, China	2.9508	0.4328	19.12	[8.19; 44.66]	2.9%
Zheng (2014) -Jiangsu, China	1.9158	0.3616	6.79	[3.34; 13.80]	3.0%
Cui (2015) -Chaoshan, China	0.0953	0.0669	1.10	[0.96; 1.25]	3.2%
Huang (2019) -Zhongshan, China	2.8293	1.0348	16.93	[2.23; 128.69]	2.0%
Li (2013) -Zhuhai, China	-0.7520	1.3243	0.47	[0.04; 6.32]	1.6%
Therese (2021) -Nairobi, Kenya	0.1398	0.1377	1.15	[0.88; 1.51]	3.2%
Therese (2021) -Nairobi, Kenya	0.1044	0.1493	1.11	[0.83; 1.49]	3.2%
Stacy (2021) -Singapore	-0.0587	0.0219	0.94	[0.90; 0.98]	3.2%
Wei (2016) -Shanghai, China	-2.3581	0.7139	0.09	[0.02; 0.38]	2.5%
Cheng (2022) -Macao, China	0.0108	0.0951	1.01	[0.84; 1.22]	3.2%
Liu (2019) -Guangzhou, China	1.2595	0.4263	3.52	[1.53; 8.13]	2.9%
Mariana (2004) -Buenos Aires, Argentina	-1.3116	0.5167	0.27	[0.10; 0.74]	2.8%
You (2022) -Gwangju, Korea	1.9475	0.6225	7.01	[2.07; 23.75]	2.6%
Lin (2015) -Quanzhou, China	0.6152	1.3179	1.85	[0.14; 24.49]	1.6%
Sun (2011) -Suzhou, China	2.2673	0.0525	9.65	[8.71; 10.70]	3.2%
Chen (2016) -Suzhou, China	0.5504	0.8253	1.73	[0.34; 8.74]	2.3%
Chen (2016) -Suzhou, China	0.8181	0.8364	2.27	[0.44; 11.68]	2.3%
Chen (2016) -Suzhou, China	2.7926	0.0756	16.32	[14.08; 18.93]	3.2%
Zhang (2022) -Jiangyin, China	2.2495	0.0385	9.48	[8.79; 10.23]	3.2%
Yan (2015) -Shanghai, China	2.7066	0.0901	14.98	[12.55; 17.87]	3.2%
Zhang (2020) -Suzhou, China	3.3278	0.0568	27.88	[24.94; 31.16]	3.2%
Random effects model			2.35	[1.46; 3.77]	100.0%
Prediction interval				[0.14; 38.03]	
Heterogeneity: I^2 = 100%, τ^2 = 1.8204, p = 0					
**Random effects model**			**2.35**	**[1.46; 3.77]**	**100.0%**
**Prediction interval**				**[0.14; 38.03]**	

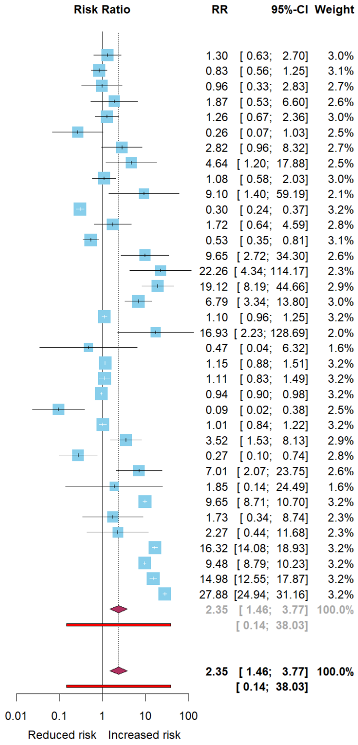

Heterogeneity: I^2 = 100%, τ^2 = 1.8204, p = 0
Test for subgroup differences: χ^2_0 = 0.00, df = 0 (p = NA)

Classified as ECDC NORMAL

Figure S3-5. Subgroup analysis of human parainfluenza virus (HPIV) meta-analysis by modelling approach

Study	logRR	SE(logRR)	RR	95%-CI	Weight
**Model = non-linear**					
Eun (2021) -Cheonan, Korea	0.2624	0.3732	1.30	[0.63; 2.70]	3.0%
Jean-Baptist (2009) -Rheinland-Pfalz, Mainz	-0.1816	0.2071	0.83	[0.56; 1.25]	3.1%
Maria (2009) -Kathmandu, Nepal	0.6258	0.6433	1.87	[0.53; 6.60]	2.6%
Eun (2021) -Cheonan, Korea	-1.3280	0.6948	0.26	[0.07; 1.03]	2.5%
Maria (2009) -Kathmandu, Nepal	1.5347	0.6883	4.64	[1.20; 17.88]	2.5%
Chen (2014) -Suzhou, China	2.2084	0.9553	9.10	[1.40; 59.19]	2.1%
Cui (2015) -Chaoshan, China	-1.2040	0.1034	0.30	[0.24; 0.37]	3.2%
Eun (2021) -Cheonan, Korea	0.5400	0.5017	1.72	[0.64; 4.59]	2.8%
Jean-Baptist (2009) -Rheinland-Pfalz, Mainz	-0.6259	0.2098	0.53	[0.35; 0.81]	3.1%
Maria (2009) -Kathmandu, Nepal	3.1028	0.8342	22.26	[4.34; 114.17]	2.3%
Cui (2015) -Chaoshan, China	0.0953	0.0669	1.10	[0.96; 1.25]	3.2%
Li (2013) -Zhuhai, China	-0.7520	1.3243	0.47	[0.04; 6.32]	1.6%
Therese (2021) -Nairobi, Kenya	0.1398	0.1377	1.15	[0.88; 1.51]	3.2%
Therese (2021) -Nairobi, Kenya	0.1044	0.1493	1.11	[0.83; 1.49]	3.2%
Stacy (2021) -Singapore	-0.0587	0.0219	0.94	[0.90; 0.98]	3.2%
Wei (2016) -Shanghai, China	-2.3581	0.7139	0.09	[0.02; 0.38]	2.5%
Mariana (2004) -Buenos Aires, Argentina	-1.3116	0.5167	0.27	[0.10; 0.74]	2.8%
You (2022) -Gwangju, Korea	1.9475	0.6225	7.01	[2.07; 23.75]	2.6%
Sun (2011) -Suzhou, China	2.2673	0.0525	9.65	[8.71; 10.70]	3.2%
Chen (2016) -Suzhou, China	0.5504	0.8253	1.73	[0.34; 8.74]	2.3%
Chen (2016) -Suzhou, China	0.8181	0.8364	2.27	[0.44; 11.68]	2.3%
Chen (2016) -Suzhou, China	2.7926	0.0756	16.32	[14.08; 18.93]	3.2%
Zhang (2022) -Jiangyin, China	2.2495	0.0385	9.48	[8.79; 10.23]	3.2%
Yan (2015) -Shanghai, China	2.7066	0.0901	14.98	[12.55; 17.87]	3.2%
Zhang (2020) -Suzhou, China	3.3278	0.0568	27.88	[24.94; 31.16]	3.2%
Random effects model			2.06	[1.12; 3.80]	70.2%
Prediction interval				[0.09; 46.74]	
Heterogeneity: I^2 = 100%, τ^2 = 2.1798, p = 0					
**Model = linear**					
Ji (2011) -Suzhou, China	-0.0362	0.5485	0.96	[0.33; 2.83]	2.7%
Zheng (2014) -Jiangsu, China	0.2290	0.3204	1.26	[0.67; 2.36]	3.0%
Ji (2011) -Suzhou, China	1.0373	0.5518	2.82	[0.96; 8.32]	2.7%
Zheng (2014) -Jiangsu, China	0.0798	0.3198	1.08	[0.58; 2.03]	3.0%
Ji (2011) -Suzhou, China	2.2673	0.6468	9.65	[2.72; 34.30]	2.6%
Ming (2022) -Wenzhou, China	2.9508	0.4328	19.12	[8.19; 44.66]	2.9%
Zheng (2014) -Jiangsu, China	1.9158	0.3616	6.79	[3.34; 13.80]	3.0%
Huang (2019) -Zhongshan, China	2.8293	1.0348	16.93	[2.23; 128.69]	2.0%
Cheng (2022) -Macao, China	0.0108	0.0951	1.01	[0.84; 1.22]	3.2%
Liu (2019) -Guangzhou, China	1.2595	0.4263	3.52	[1.53; 8.13]	2.9%
Lin (2015) -Quanzhou, China	0.6152	1.3179	1.85	[0.14; 24.49]	1.6%
Random effects model			3.07	[1.56; 6.06]	29.8%
Prediction interval				[0.27; 34.52]	
Heterogeneity: I^2 = 89%, τ^2 = 1.0231, p < 0.01					
**Random effects model**			**2.35**	**[1.46; 3.77]**	**100.0%**
**Prediction interval**				**[0.14; 38.03]**	

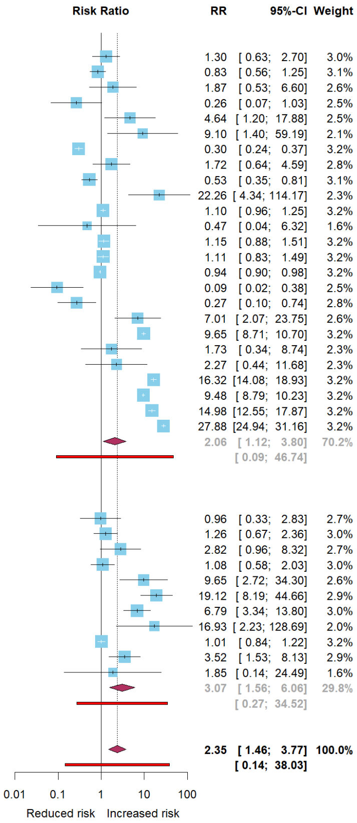

Heterogeneity: I^2 = 100%, τ^2 = 1.8204, p = 0
Test for subgroup differences: χ^2_1 = 0.73, df = 1 (p = 0.39)

Classified as ECDC NORMAL

Figure S3-6. Subgroup analysis of human parainfluenza virus (HPIV) meta-analysis by lag type

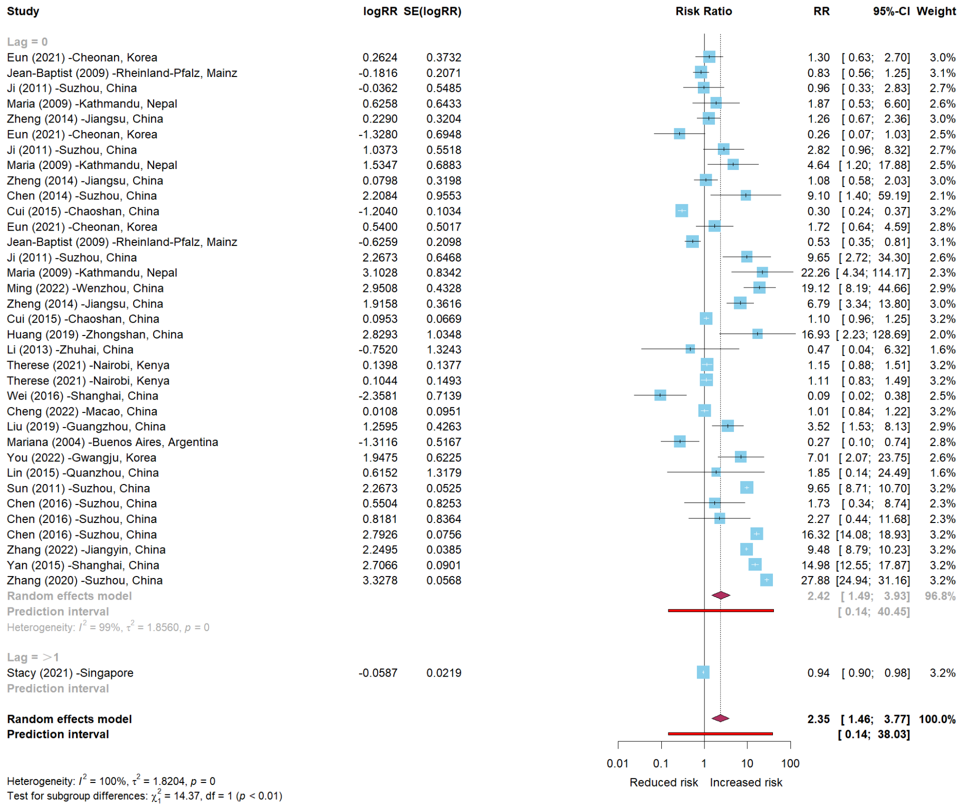

Classified as ECDC NORMAL

Figure S3-7. Leave-one-out analysis of human parainfluenza virus (HPIV) meta-analysis

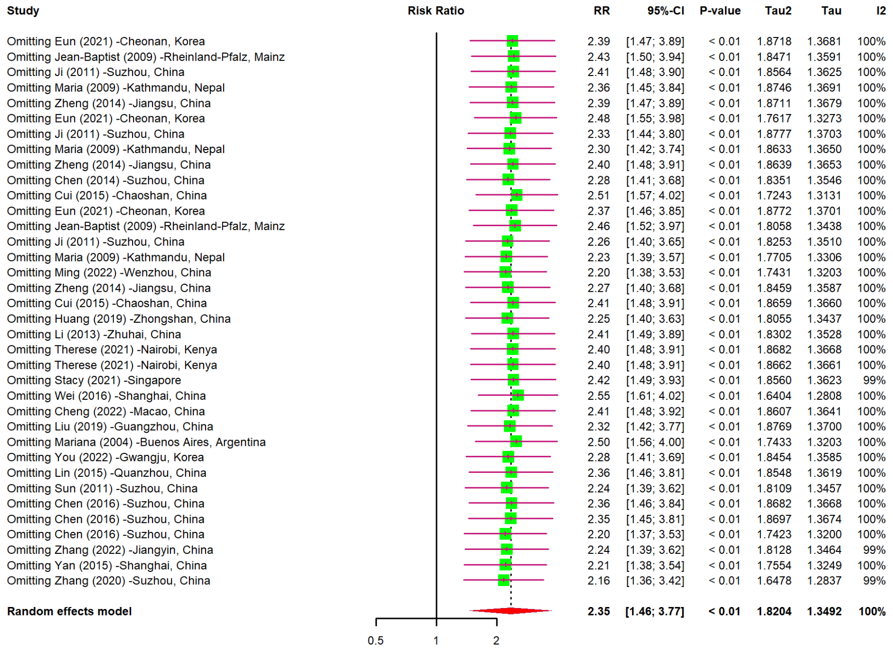

Figure S3-8. Funnel plot of human parainfluenza virus (HPIV) estimates

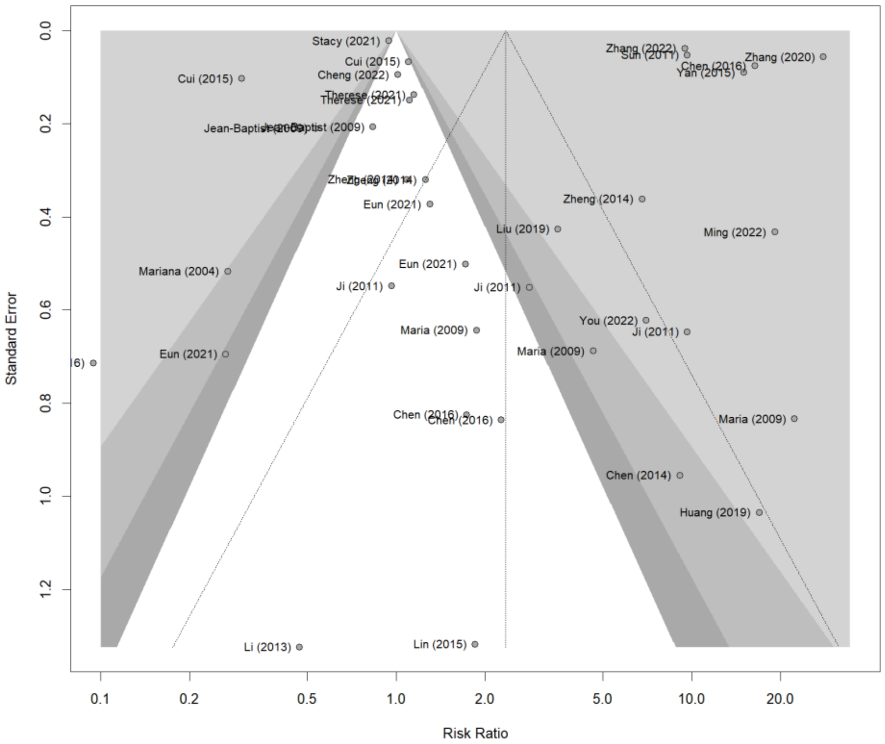

Linear regression test of funnel plot asymmetry

Egger's test result: t = 0.43, df = 34, p = 0.6721

Sample estimates:

bias	se.bias	intercept	se.intercept
1.3038	3.0541	0.8533	0.2769

Classified as ECDC NORMAL

Figure S3-9 Trim and fill of human parainfluenza virus (HPIV) meta-analysis

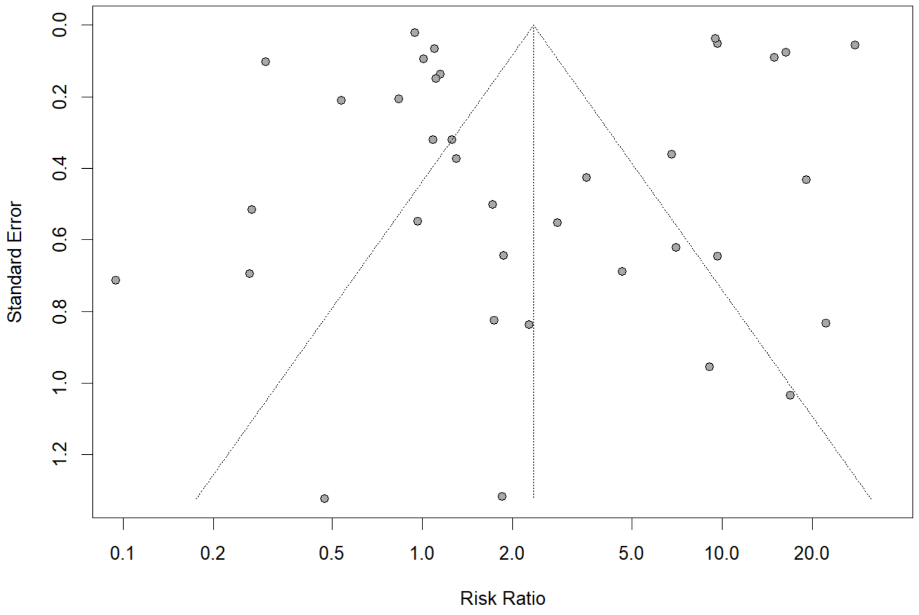

Number of studies: k = 36 (with 0 added studies)

Random effects model: RR=2.3464 [1.4620-3.7656], z= 3.53, p=0.0004

Quantifying heterogeneity:

tau^2 = 1.8204 [1.0997; 3.2263]; tau = 1.3492 [1.0487; 1.7962]

I^2 = 99.5% [99.5%; 99.6%]; H = 14.72 [14.06; 15.41]

Figure S4. Random-effects meta-analysis of human metapneumoviruses (HMPV) estimates (14 studies)

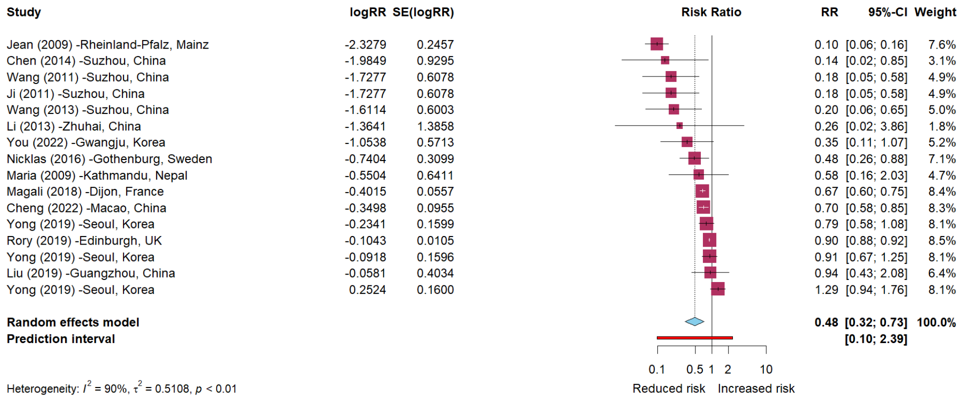

Classified as ECDC NORMAL

Figure S4-1. Subgroup analysis of human metapneumoviruses (HMPV) meta-analysis by Köppen-Geiger climate

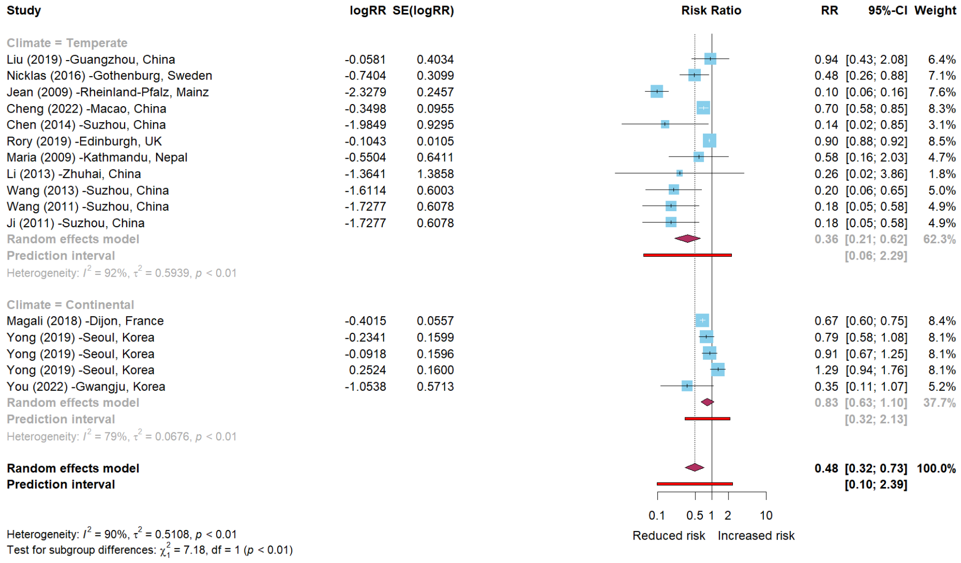

Figure S4-2. Subgroup analysis of human metapneumoviruses (HMPV) meta-analysis by World Bank income category

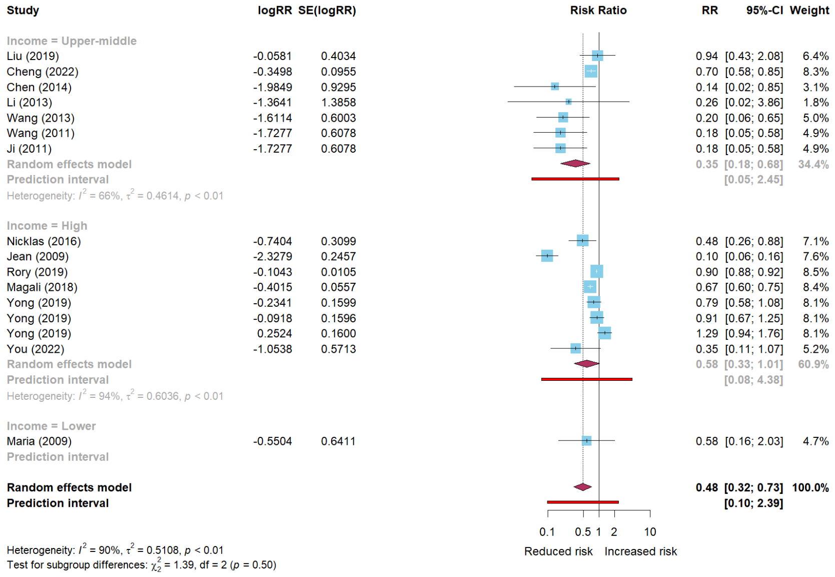

Classified as ECDC NORMAL

Figure S4-3. Subgroup analysis of human metapneumoviruses (HMPV) meta-analysis by temporal resolution

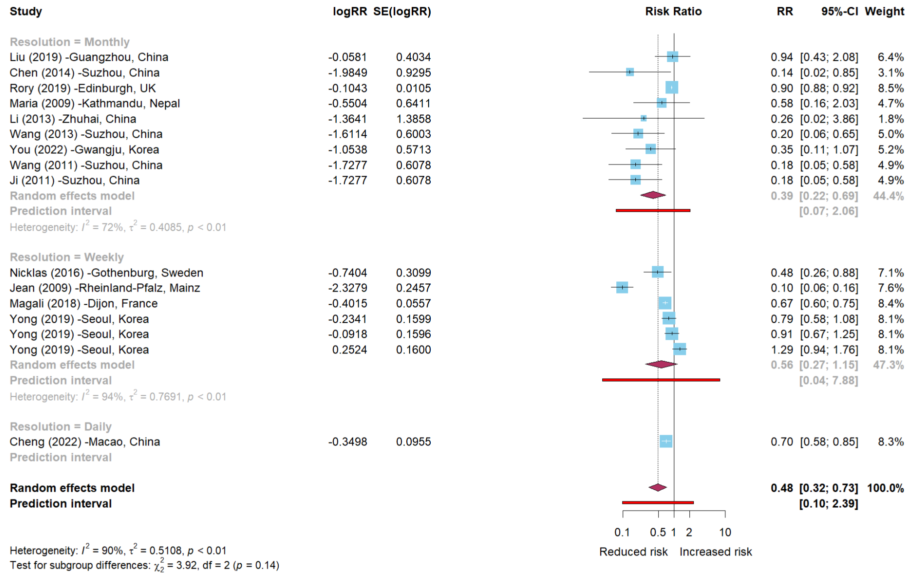

Figure S4-4. Subgroup analysis of human metapneumoviruses (HMPV) meta-analysis by exposure measure

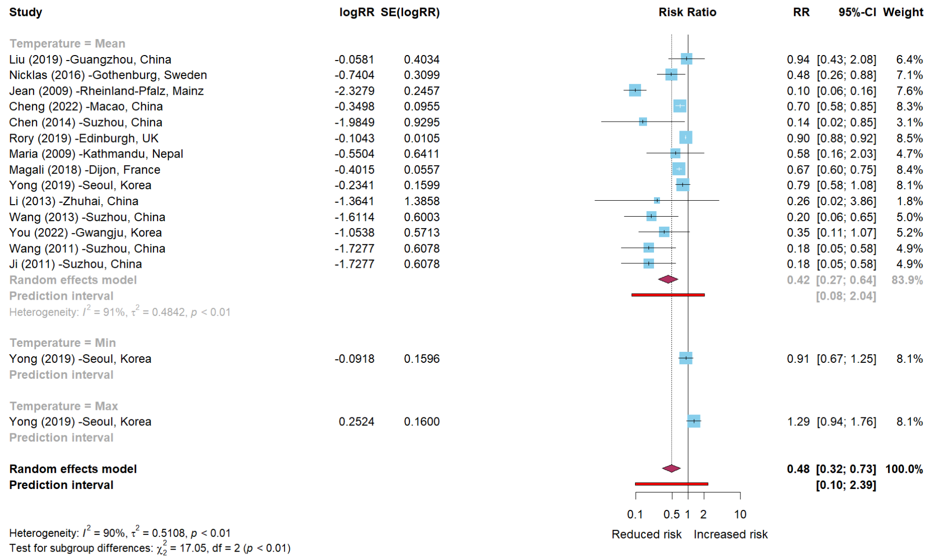

Classified as ECDC NORMAL

Figure S4-5. Subgroup analysis of human metapneumoviruses (HMPV) meta-analysis by modelling approach

Study	logRR	SE(logRR)	RR	95%-CI	Weight
**Model = linear**					
Liu (2019) -Guangzhou, China	-0.0581	0.4034	0.94	[0.43; 2.08]	6.4%
Nicklas (2016) -Gothenburg, Sweden	-0.7404	0.3099	0.48	[0.26; 0.88]	7.1%
Cheng (2022) -Macao, China	-0.3498	0.0955	0.70	[0.58; 0.85]	8.3%
Rory (2019) -Edinburgh, UK	-0.1043	0.0105	0.90	[0.88; 0.92]	8.5%
Yong (2019) -Seoul, Korea	-0.2341	0.1599	0.79	[0.58; 1.08]	8.1%
Yong (2019) -Seoul, Korea	-0.0918	0.1596	0.91	[0.67; 1.25]	8.1%
Yong (2019) -Seoul, Korea	0.2524	0.1600	1.29	[0.94; 1.76]	8.1%
Wang (2013) -Suzhou, China	-1.6114	0.6003	0.20	[0.06; 0.65]	5.0%
Wang (2011) -Suzhou, China	-1.7277	0.6078	0.18	[0.05; 0.58]	4.9%
Ji (2011) -Suzhou, China	-1.7277	0.6078	0.18	[0.05; 0.58]	4.9%
Random effects model			0.66	[0.47; 0.93]	69.3%
Prediction interval				[0.21; 2.05]	
Heterogeneity: I^2 = 76%, τ^2 = 0.2101, p < 0.01					
**Model = non-linear**					
Jean (2009) -Rheinland-Pfalz, Mainz	-2.3279	0.2457	0.10	[0.06; 0.16]	7.6%
Chen (2014) -Suzhou, China	-1.9849	0.9295	0.14	[0.02; 0.85]	3.1%
Maria (2009) -Kathmandu, Nepal	-0.5504	0.6411	0.58	[0.16; 2.03]	4.7%
Magali (2018) -Dijon, France	-0.4015	0.0557	0.67	[0.60; 0.75]	8.4%
Li (2013) -Zhuhai, China	-1.3641	1.3858	0.26	[0.02; 3.86]	1.8%
You (2022) -Gwangju, Korea	-1.0538	0.5713	0.35	[0.11; 1.07]	5.2%
Random effects model			0.29	[0.13; 0.65]	30.7%
Prediction interval				[0.02; 3.50]	
Heterogeneity: I^2 = 92%, τ^2 = 0.6357, p < 0.01					
**Random effects model**			**0.48**	**[0.32; 0.73]**	**100.0%**
**Prediction interval**				**[0.10; 2.39]**	

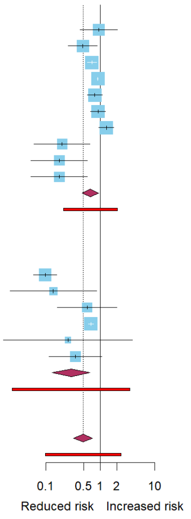

Heterogeneity: I^2 = 90%, τ^2 = 0.5108, p < 0.01
Test for subgroup differences: χ^2_1 = 3.41, df = 1 (p = 0.06)

Figure S4-6. Leave-one-out analysis of human metapneumoviruses (HMPV) meta-analysis

Study	RR	95%-CI	P-value	Tau2	Tau	I2
Omitting Liu (2019) -Guangzhou, China	0.46	[0.30; 0.71]	< 0.01	0.5352	0.7316	91%
Omitting Nicklas (2016) -Gothenburg, Sweden	0.48	[0.31; 0.74]	< 0.01	0.5613	0.7492	91%
Omitting Jean (2009) -Rheinland-Pfalz, Mainz	0.63	[0.48; 0.83]	< 0.01	0.1674	0.4092	80%
Omitting Cheng (2022) -Macao, China	0.46	[0.30; 0.72]	< 0.01	0.5591	0.7477	90%
Omitting Chen (2014) -Suzhou, China	0.50	[0.33; 0.76]	< 0.01	0.4989	0.7063	91%
Omitting Rory (2019) -Edinburgh, UK	0.45	[0.29; 0.70]	< 0.01	0.5335	0.7304	86%
Omitting Maria (2009) -Kathmandu, Nepal	0.48	[0.31; 0.73]	< 0.01	0.5457	0.7387	91%
Omitting Magali (2018) -Dijon, France	0.47	[0.30; 0.72]	< 0.01	0.5629	0.7503	89%
Omitting Yong (2019) -Seoul, Korea	0.46	[0.30; 0.71]	< 0.01	0.5491	0.7410	91%
Omitting Yong (2019) -Seoul, Korea	0.46	[0.30; 0.70]	< 0.01	0.5335	0.7304	91%
Omitting Yong (2019) -Seoul, Korea	0.45	[0.30; 0.67]	< 0.01	0.4763	0.6901	90%
Omitting Li (2013) -Zhuhai, China	0.49	[0.32; 0.74]	< 0.01	0.5188	0.7203	91%
Omitting Wang (2013) -Suzhou, China	0.51	[0.33; 0.77]	< 0.01	0.5027	0.7090	90%
Omitting You (2022) -Gwangju, Korea	0.49	[0.32; 0.75]	< 0.01	0.5389	0.7341	91%
Omitting Wang (2011) -Suzhou, China	0.51	[0.34; 0.77]	< 0.01	0.4930	0.7021	90%
Omitting Ji (2011) -Suzhou, China	0.51	[0.34; 0.77]	< 0.01	0.4930	0.7021	90%
**Random effects model**	**0.48**	**[0.32; 0.73]**	**< 0.01**	**0.5108**	**0.7147**	**90%**

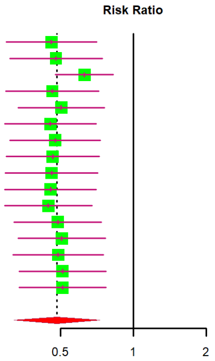

Classified as ECDC NORMAL

Figure S4-7. Funnel plot of human metapneumoviruses (HMPV) estimates

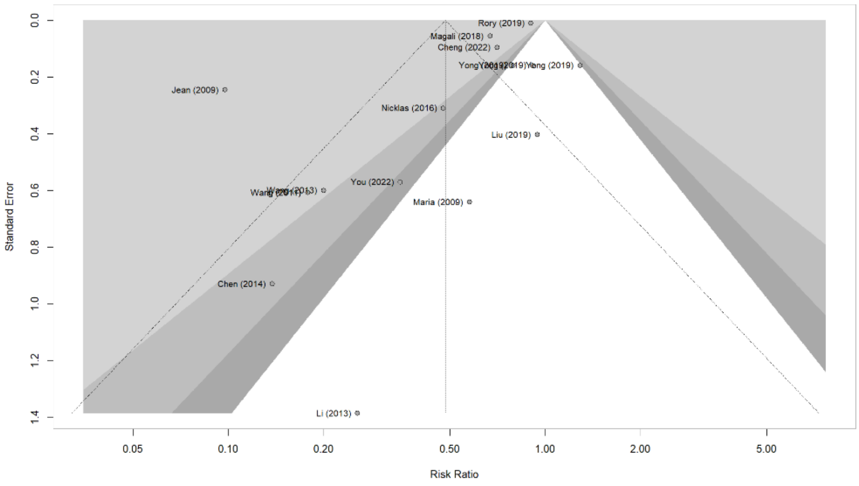

Linear regression test of funnel plot asymmetry

Egger's test result: t = -2.91, df = 14, p-value = 0.0115

Sample estimates:

bias	se.bias	intercept	se.intercept
-2.0778	0.7151	-0.0876	0.0290

Figure S4-8. Trim and fill of human metapneumoviruses (HMPV) meta-analysis

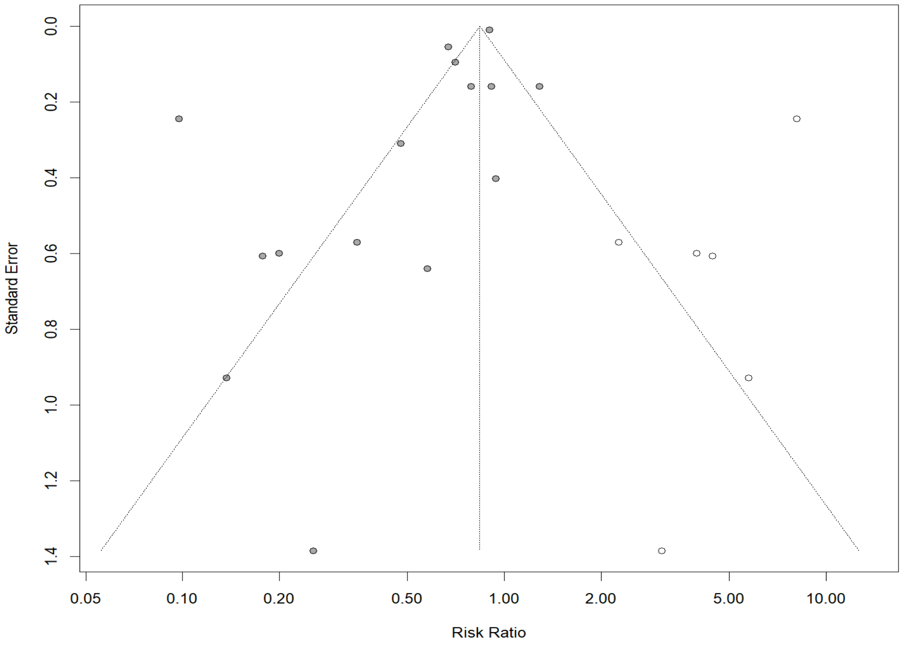

Number of studies: k = 23 (with 7 added studies)

Random effects model: RR= 0.8394 [0.5034; 1.3998] , Z =-0.67, *P*= 0.5023

Quantifying heterogeneity:

tau^2 = 1.2694 [0.6562, 2.9522]; tau = 1.1267 [0.8101, 1.7182]

I^2 = 91.6% [88.7%, 93.8%]; H = 3.45 [2.97, 4.00]

Figure S5. Random-effects meta-analysis of human rhinovirus (HRV) estimates (12 studies)

Classified as ECDC NORMAL

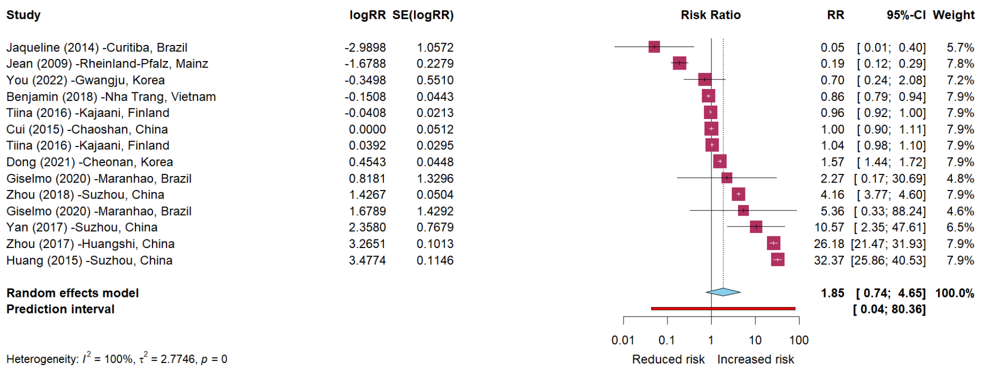

Figure S5-1. Subgroup analysis of human rhinovirus (HRV) meta-analysis by Köppen-Geiger climate

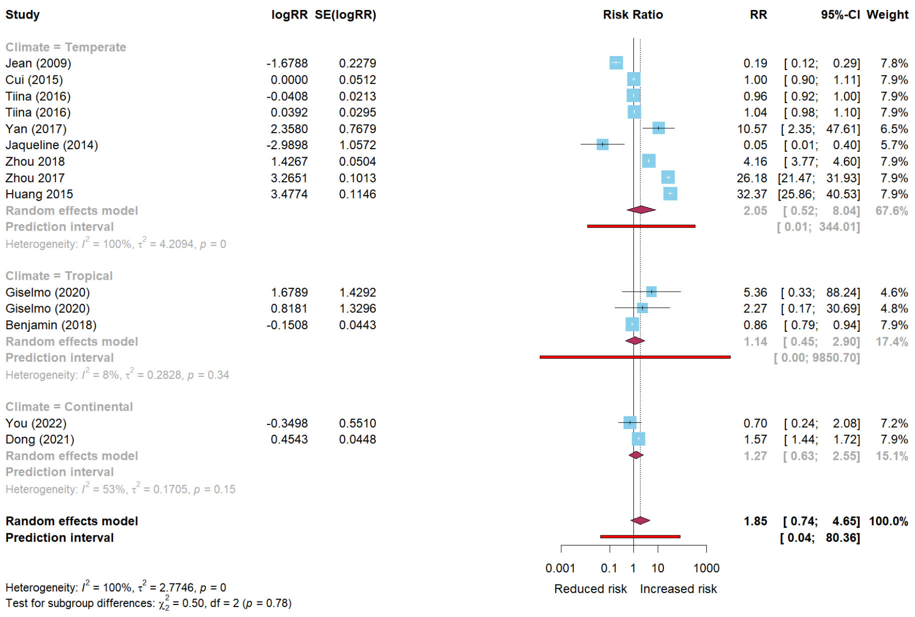

Classified as ECDC NORMAL

Figure S5-2. Subgroup analysis of human rhinovirus (HRV) meta-analysis by World Bank income category

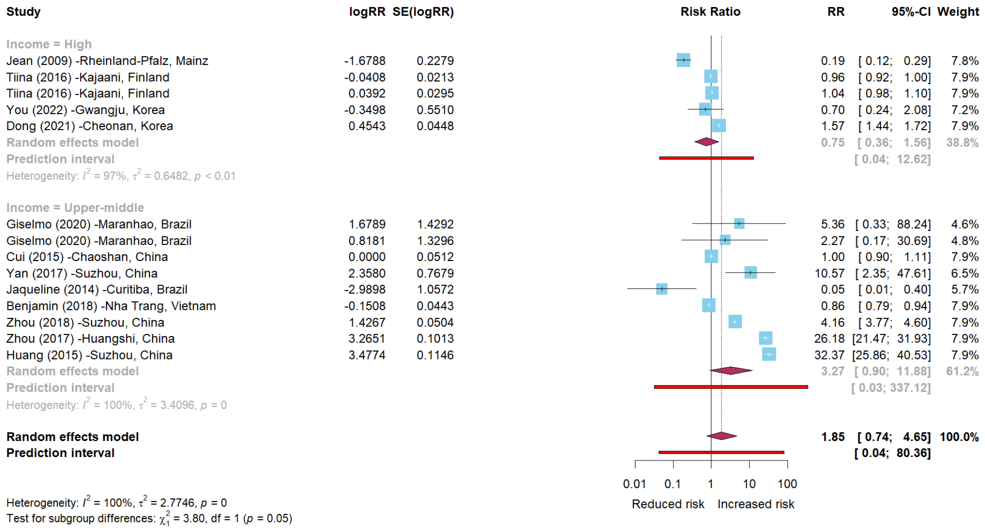

Figure S5-3. Subgroup analysis of human rhinovirus (HRV) meta-analysis by temporal resolution

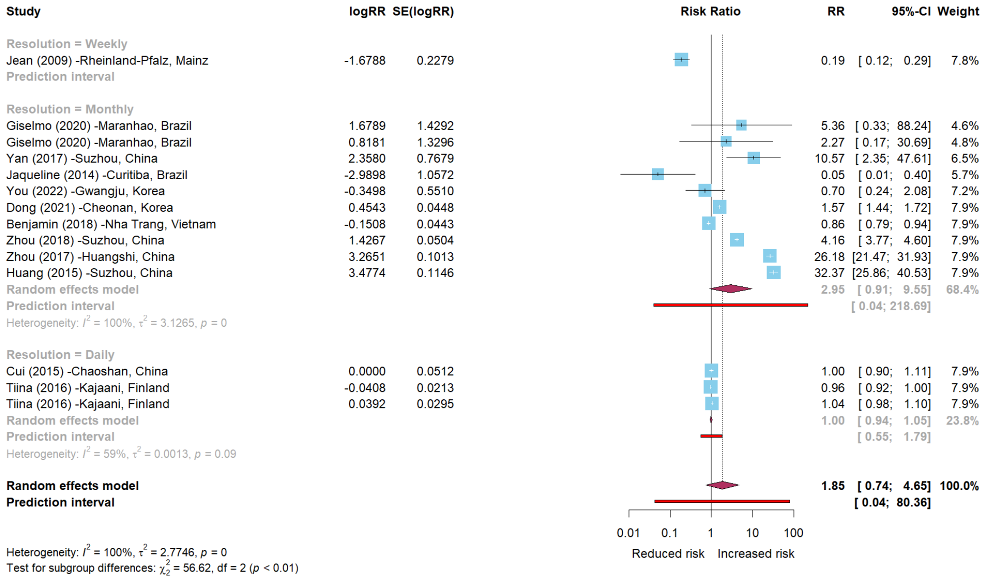

Figure S5-4. Subgroup analysis of human rhinovirus (HRV) meta-analysis by exposure measure

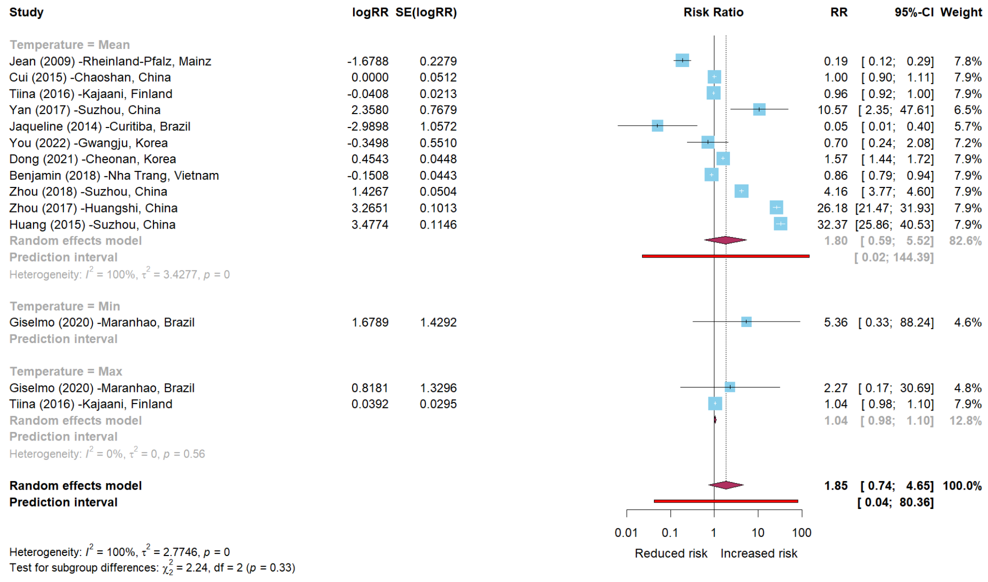

Figure S5-5. Subgroup analysis of human rhinovirus (HRV) meta-analysis by modelling approach

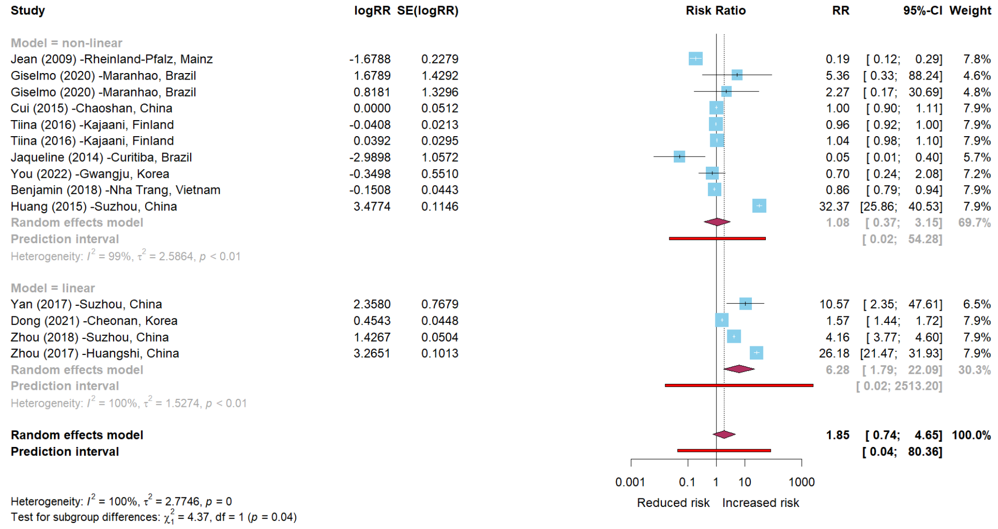

Classified as ECDC NORMAL

Figure S5-6. Leave-one-out analysis of human rhinovirus (HRV) meta-analysis

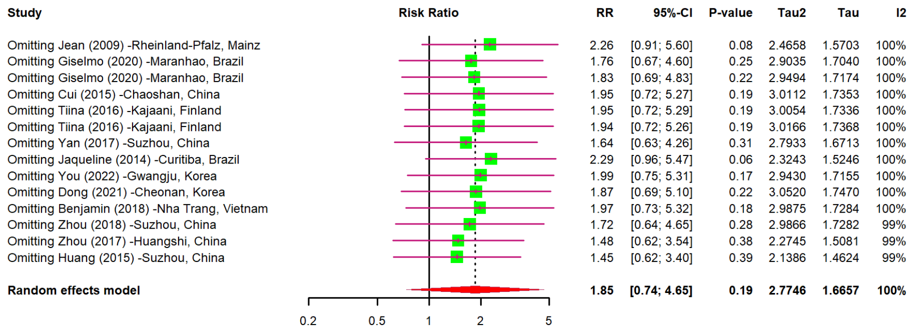

Figure S5-7. Funnel plot of human rhinovirus (HRV) estimates

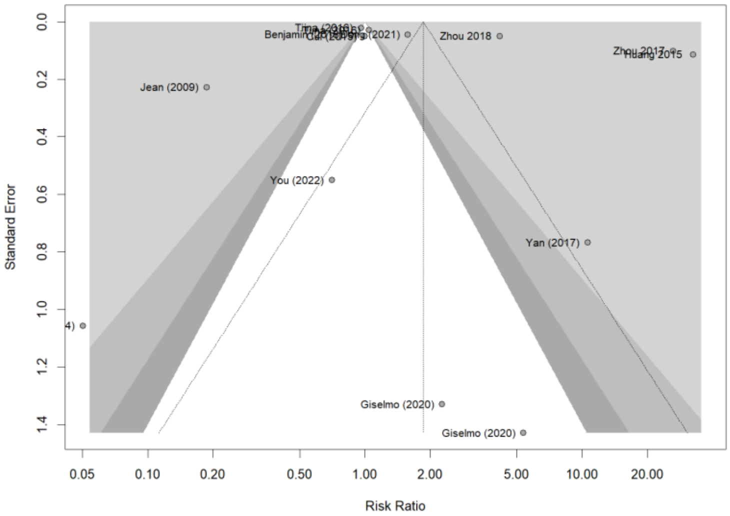

Linear regression test of funnel plot asymmetry

Egger’s test result: t = 1.32, df = 13, p-value = 0.2086

Figure S5-8. Trim and fill of human rhinovirus (HRV) meta-analysis

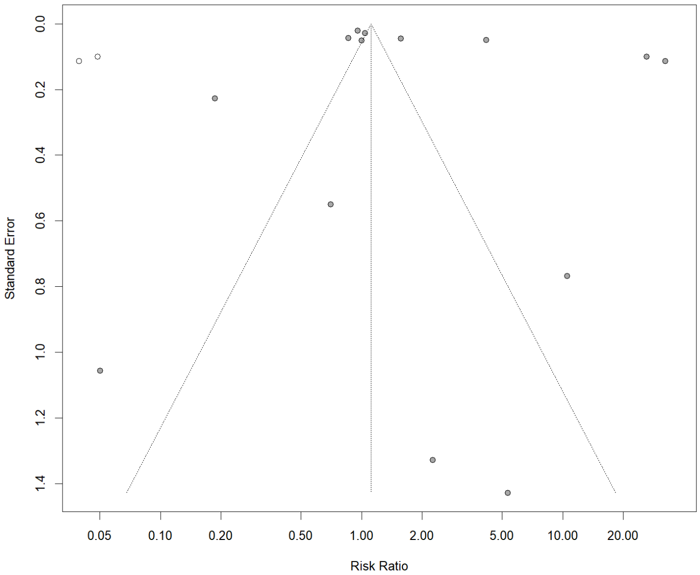

Number of studies: k = 16 (with 2 added studies)

Classified as ECDC NORMAL

Random effects model: RR 1.1163 [0.3960; 3.1466], Z=0.21, *P*= 0.8352

Quantifying heterogeneity: tau^2 = 4.1785 [2.1504; 10.1327]; tau = 2.0441 [1.4664; 3.1832]; I^2 = 99.7% [99.6%; 99.7%]; H = 17.43 [16.37; 18.54]

Figure S6. Random-effects meta-analysis of human adenoviruses (HAdVs) estimates (16 studies)

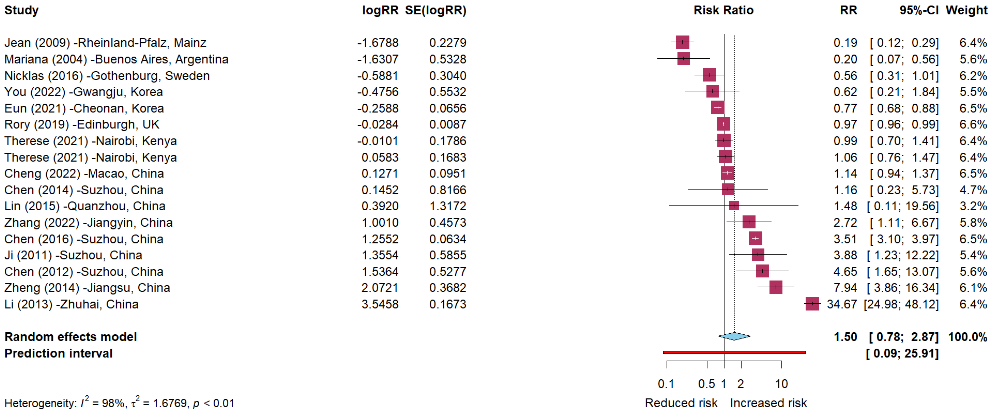

Figure S6-1. Subgroup analysis of human adenoviruses (HAdVs) meta-analysis by Köppen-Geiger climate

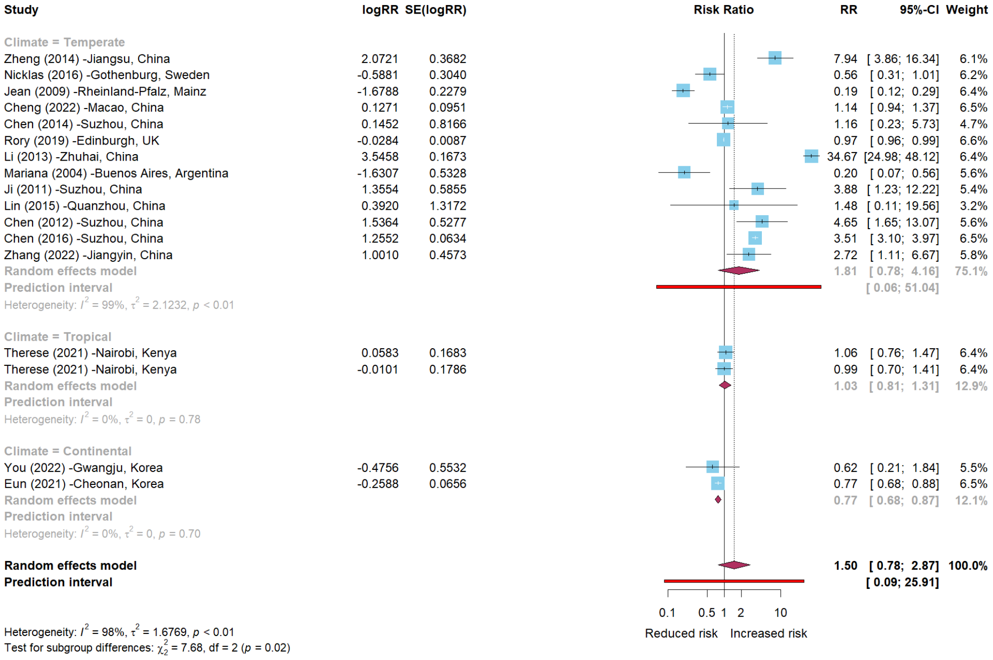

Classified as ECDC NORMAL

Figure S6-2. Subgroup analysis of human adenoviruses (HAdVs) meta-analysis by World Bank income category

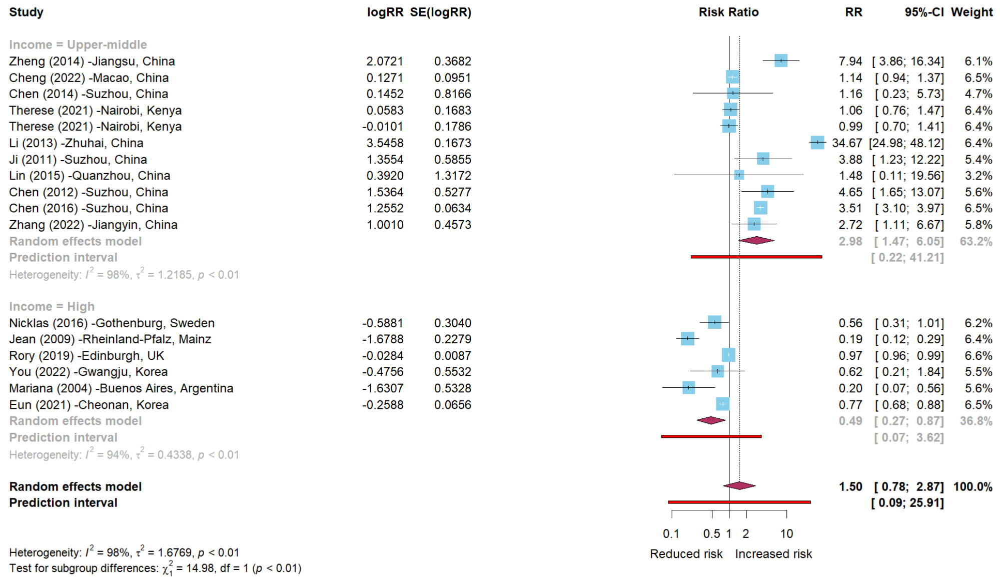

Figure S6-3. Subgroup analysis of human adenoviruses (HAdVs) meta-analysis by temporal resolution

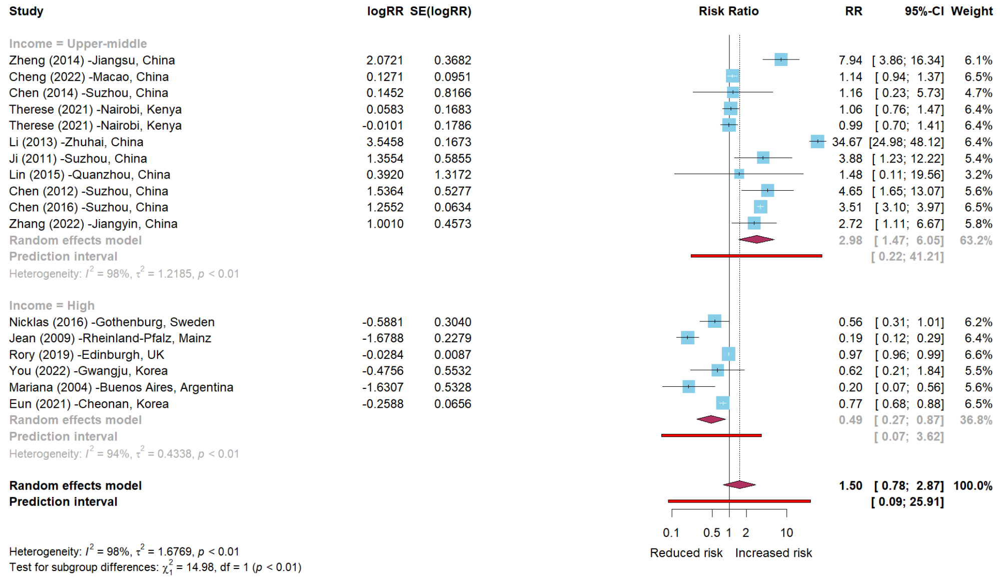

Classified as ECDC NORMAL

Figure S6-4. Subgroup analysis of human adenoviruses (HAdVs) meta-analysis by exposure measure

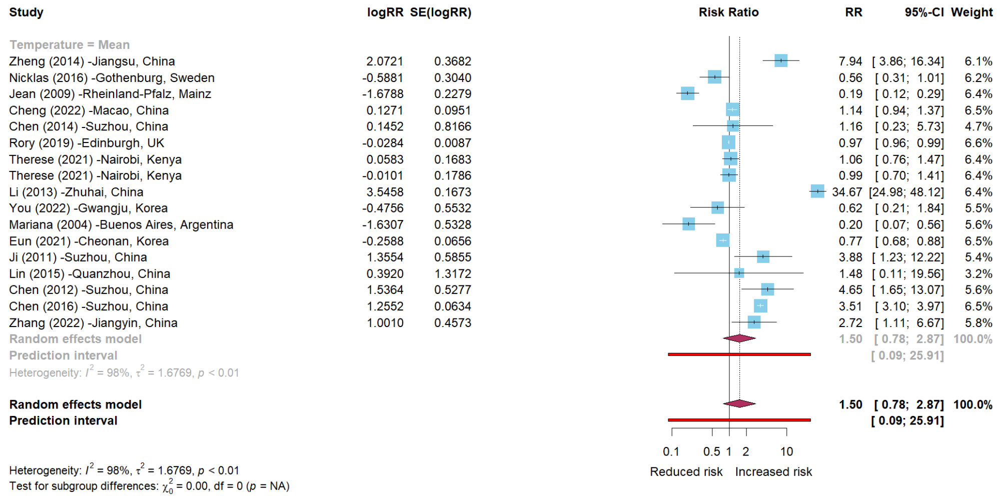

Figure S6-5. Subgroup analysis of human adenoviruses (HAdVs) meta-analysis by modelling approach

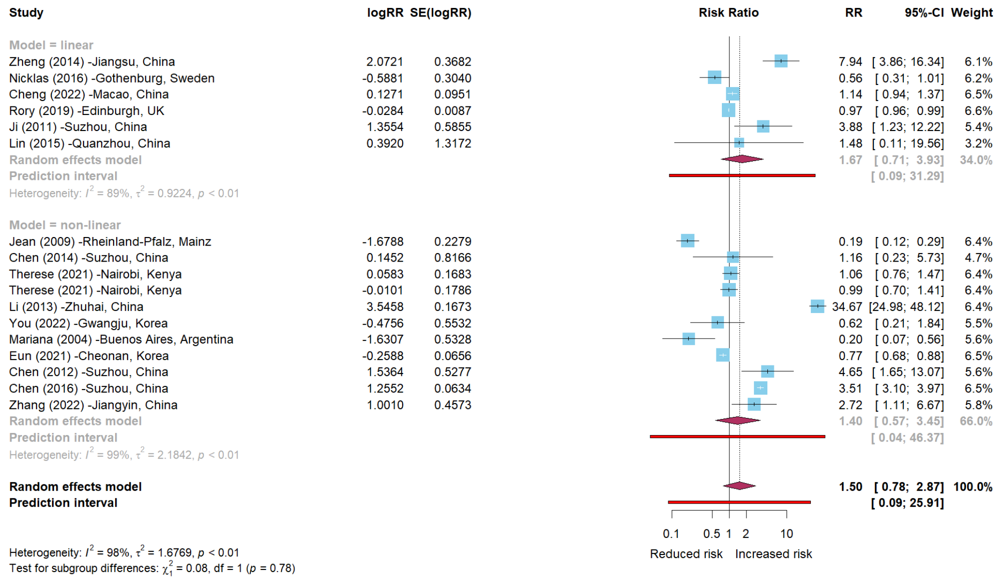

Classified as ECDC NORMAL

Figure S6-6. Leave-one-out analysis of human adenoviruses (HAdVs) meta-analysis

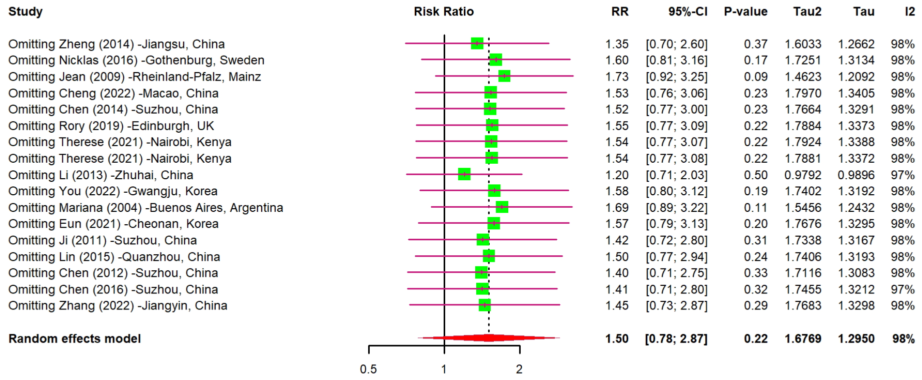

Figure S6-7. Funnel plot of human adenoviruses (HAdVs) estimates

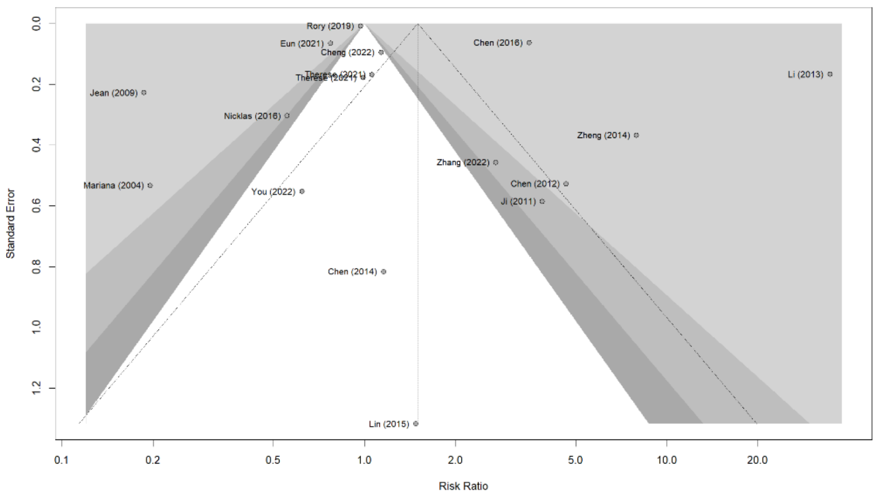

Linear regression test of funnel plot asymmetry

Egger's test result: t = 1.22, df = 15, p-value = 0.2409

Sample estimates:

bias	se.bias	intercept	se.intercept
2.5030	2.0500	-0.0349	0.0713

Figure S6-8. Trim and fill of human adenoviruses (HAdVs) meta-analysis

Classified as ECDC NORMAL

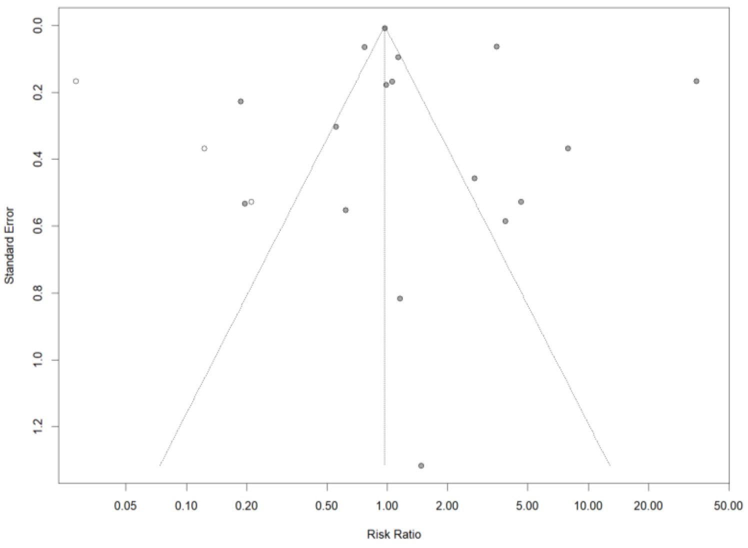

Number of studies: k = 20 (with 3 added studies)

Random effects model : RR=0.9707 [0.4667; 2.0189] , Z= -0.08 , P=0.9365

Quantifying heterogeneity:

tau^2 = 2.5968 [1.4069; 5.4493]; tau = 1.6114 [1.1861; 2.3344]

I^2 = 98.7% [98.5%; 98.9%]; H = 8.83 [8.09; 9.65]

Figure S7. Random-effects meta-analysis of human bocavirus (HBoV) estimates (nine studies)

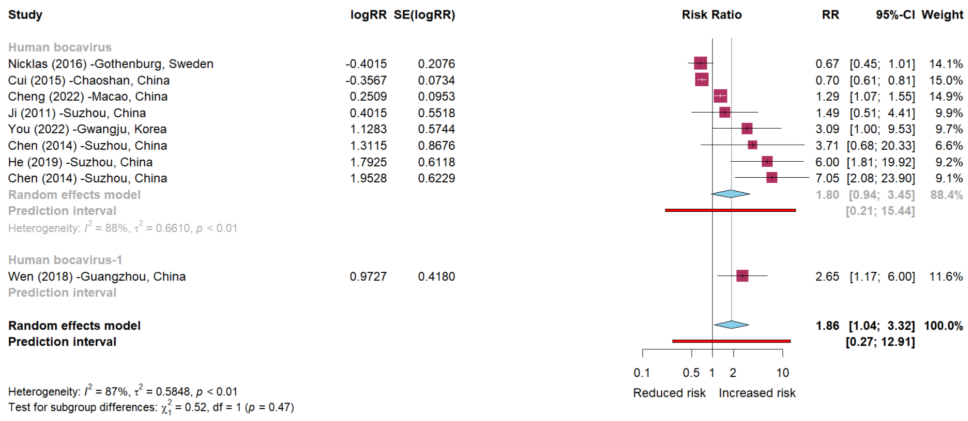

Classified as ECDC NORMAL

Figure S7-1. Subgroup analysis of human bocavirus (HBoV) meta-analysis by Köppen-Geiger climate

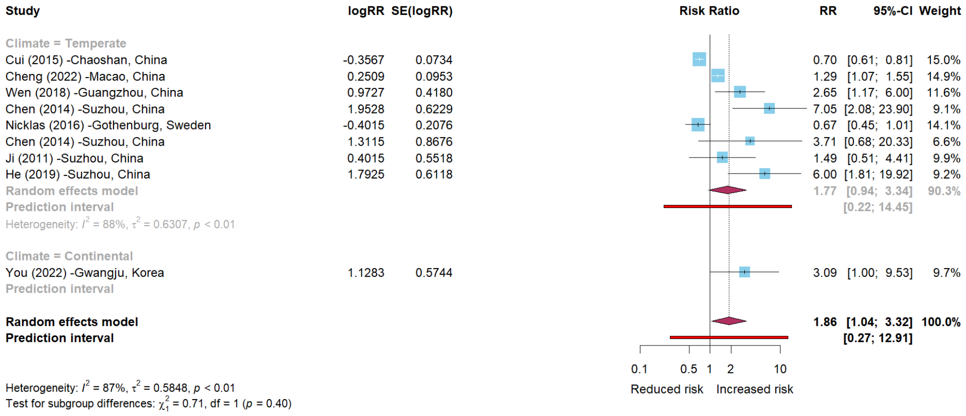

Figure S7-2. Subgroup analysis of human bocavirus (HBoV) meta-analysis by World Bank income category

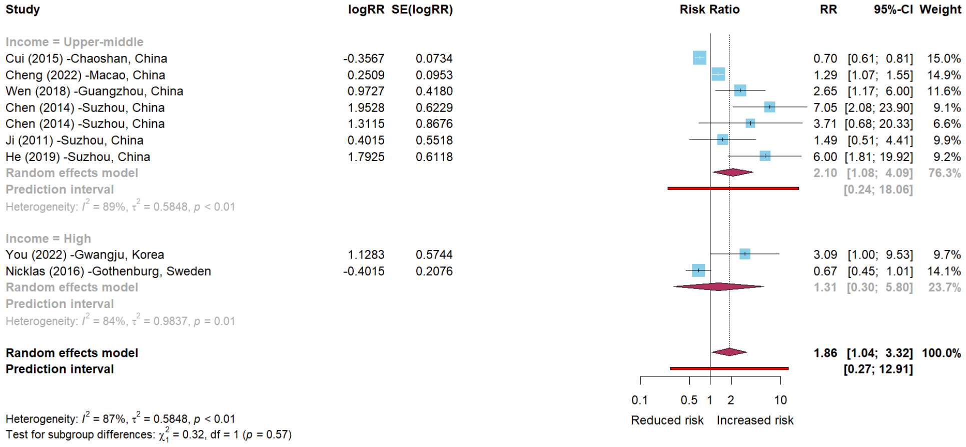

Figure S7-3. Subgroup analysis of human bocavirus (HBoV) meta-analysis by temporal resolution

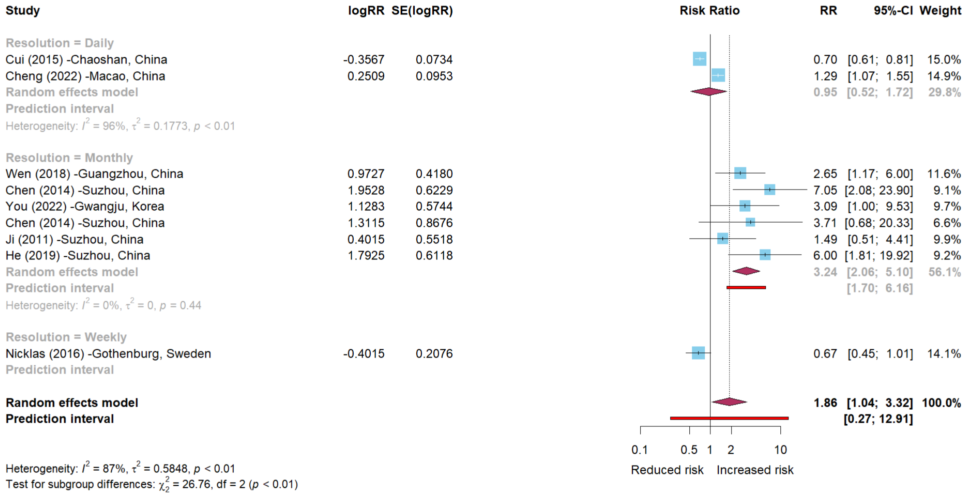

Figure S7-4. Subgroup analysis of human bocavirus (HBoV) meta-analysis by exposure measure

Classified as ECDC NORMAL

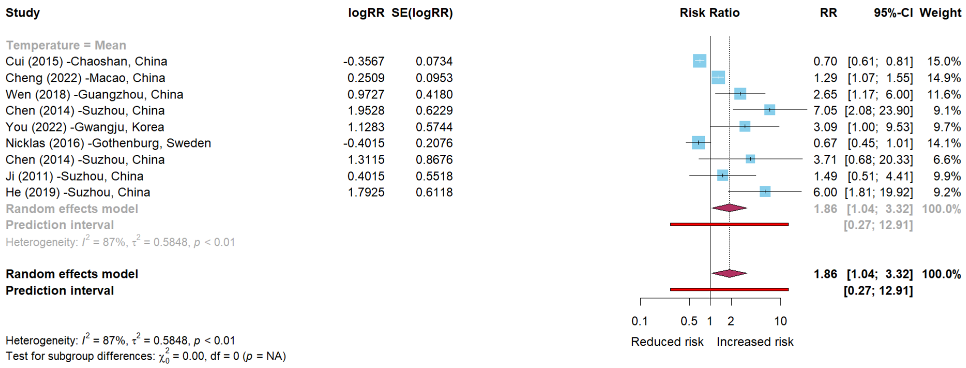

Figure S7-5. Subgroup analysis of human bocavirus (HBoV) meta-analysis by modelling approach

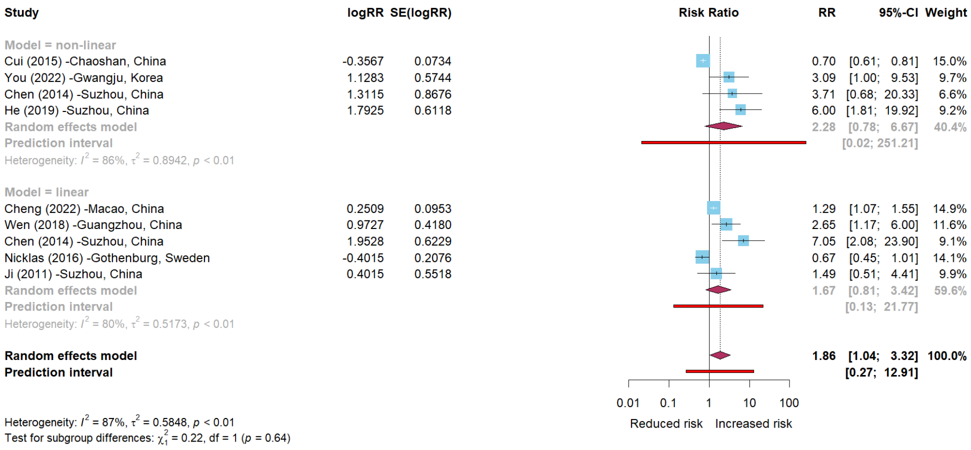

Figure S7-6. Leave-one-out analysis of human bocavirus (HBoV) meta-analysis

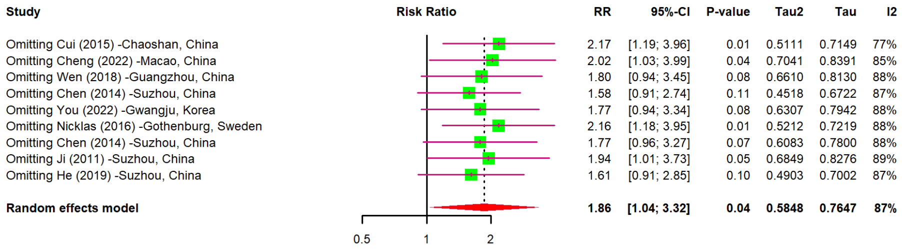

Figure S8. Random-effects meta-analysis of enterovirus(EV) estimates (four studies)

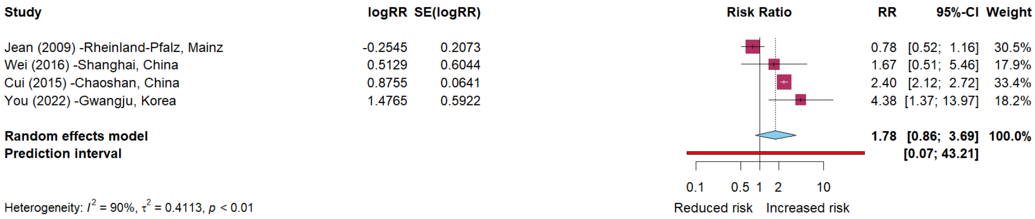

Figure S8-1. Subgroup analysis of enterovirus (EV) meta-analysis by Köppen-Geiger climate

Classified as ECDC NORMAL

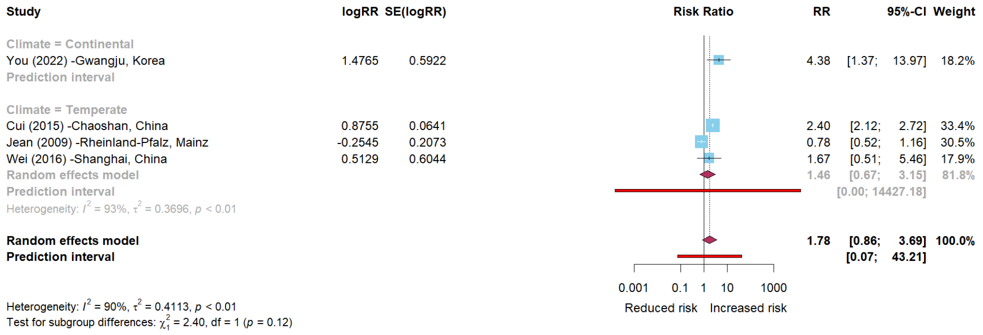

Figure S8-2. Subgroup analysis of enterovirus (EV) meta-analysis by World Bank income category

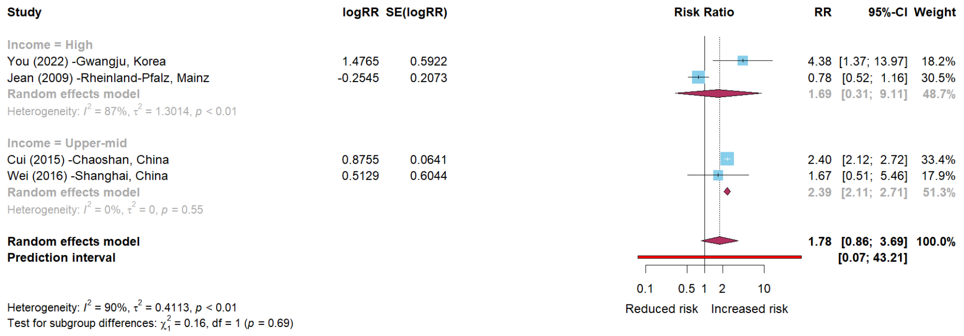

Figure S8-3. Subgroup analysis of enterovirus (EV) meta-analysis by temporal resolution

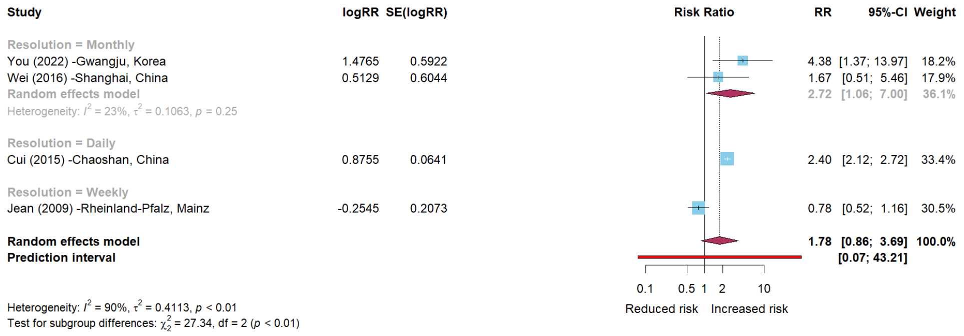

Figure S8-4. Subgroup analysis of enterovirus (EV) meta-analysis by exposure measure

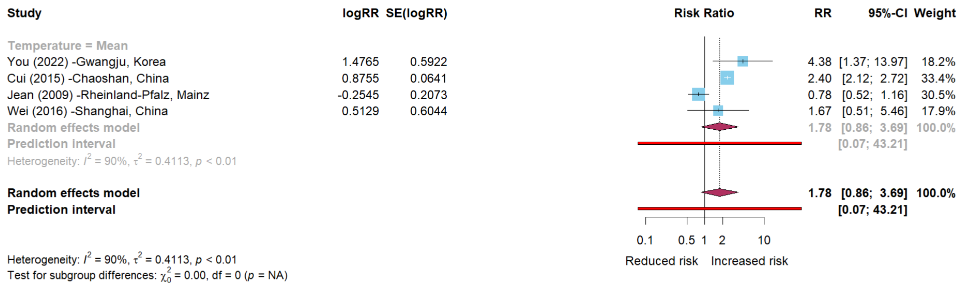

Figure S8-5. Subgroup analysis of enterovirus (EV) meta-analysis by modelling approach

Classified as ECDC NORMAL

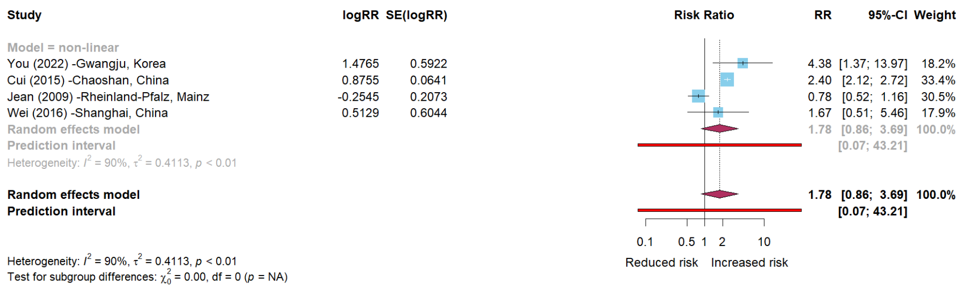

Figure S8-6. Leave-one-out analysis of enterovirus (EV) meta-analysis

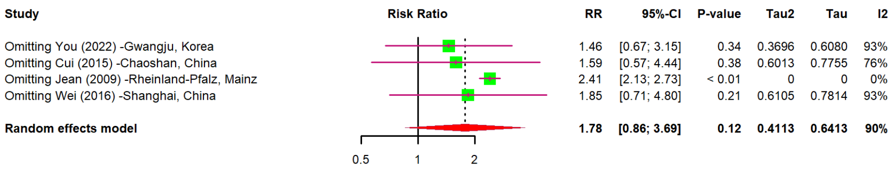

Figure S8-7. Sensitivity analysis by excluding study with high risk of bias for Jean et al 2009

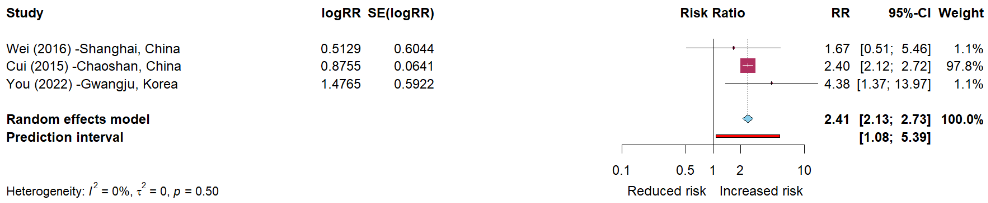

Figure S9. Random-effects meta-analysis of severe acute respiratory syndrome coronavirus 2 (SARS-CoV-2) estimates (nine studies)

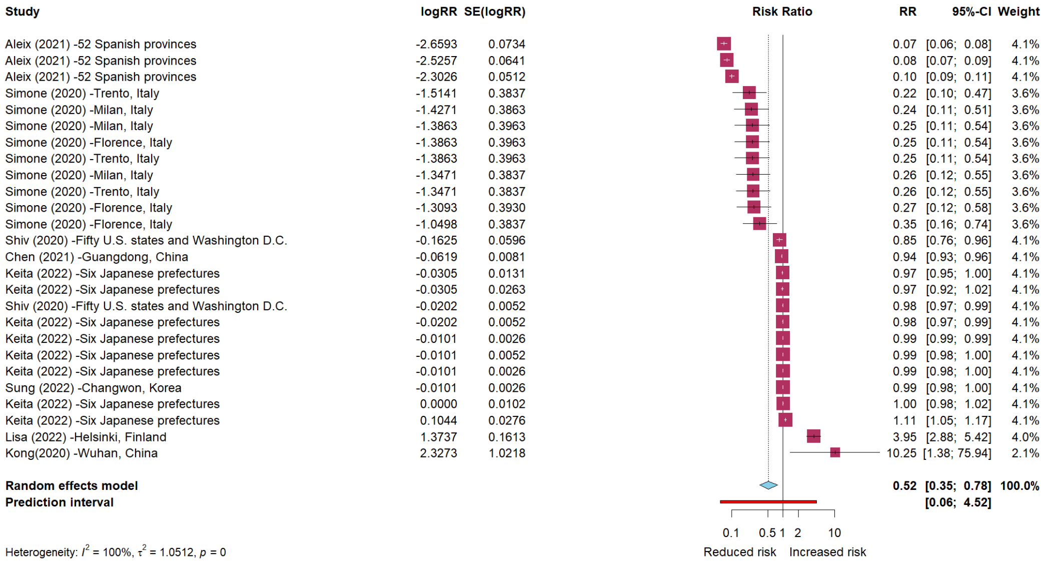

Figure S9-1. Subgroup analysis of severe acute respiratory syndrome coronavirus 2 (SARS-CoV-2) meta-analysis by Köppen-Geiger climate

Classified as ECDC NORMAL

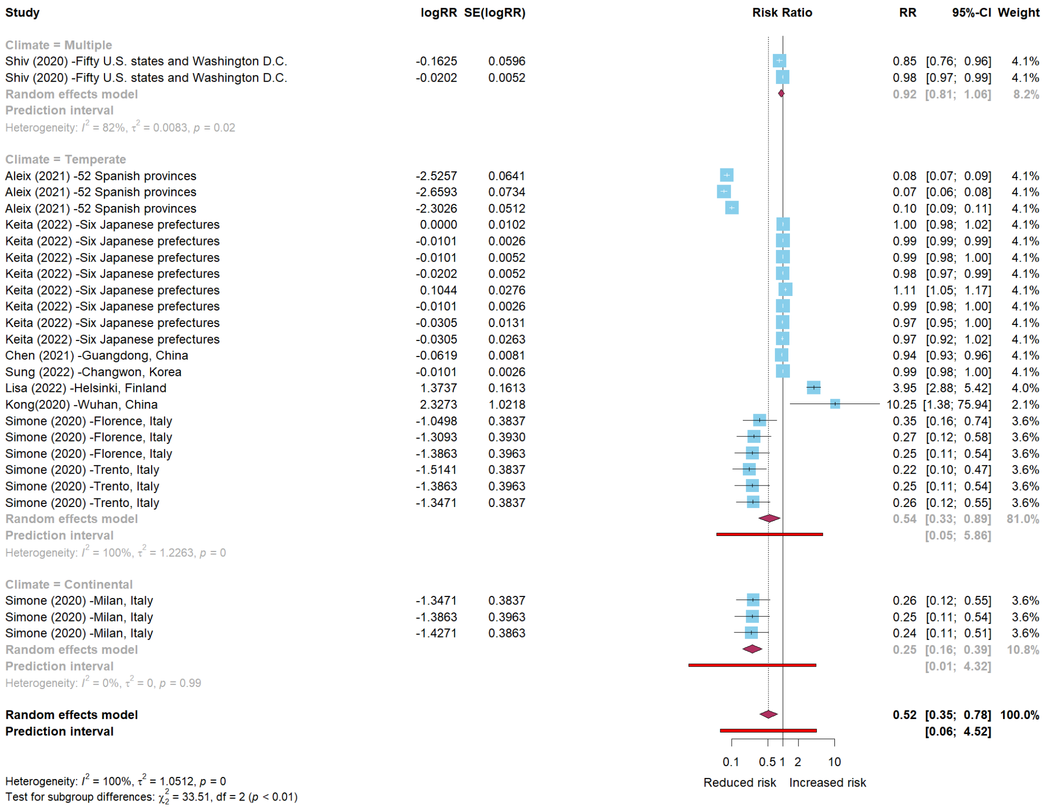
Study logRR SE(logRR) Risk Ratio RR 95%-CI Weight
Climate = Multiple
Shiv (2020) -Fifty U.S. states and Washington D.C. -0.1625 0.0596 0.85 [0.76; 0.96] 4.1%
Shiv (2020) -Fifty U.S. states and Washington D.C. -0.0202 0.0052 0.98 [0.97; 0.99] 4.1%
Random effects model 0.92 [0.81; 1.06] 8.2%
Prediction interval
Heterogeneity: I^2 = 82%, τ^2 = 0.0083, p = 0.02
Climate = Temperate
Aleix (2021) -52 Spanish provinces -2.5257 0.0641 0.08 [0.07; 0.09] 4.1%
Aleix (2021) -52 Spanish provinces -2.6593 0.0734 0.07 [0.06; 0.08] 4.1%
Aleix (2021) -52 Spanish provinces -2.3026 0.0512 0.10 [0.09; 0.11] 4.1%
Keita (2022) -Six Japanese prefectures 0.0000 0.0102 1.00 [0.98; 1.02] 4.1%
Keita (2022) -Six Japanese prefectures -0.0101 0.0026 0.99 [0.99; 0.99] 4.1%
Keita (2022) -Six Japanese prefectures -0.0101 0.0052 0.99 [0.98; 1.00] 4.1%
Keita (2022) -Six Japanese prefectures -0.0202 0.0052 0.98 [0.97; 0.99] 4.1%
Keita (2022) -Six Japanese prefectures 0.1044 0.0276 1.11 [1.05; 1.17] 4.1%
Keita (2022) -Six Japanese prefectures -0.0101 0.0026 0.99 [0.98; 1.00] 4.1%
Keita (2022) -Six Japanese prefectures -0.0305 0.0131 0.97 [0.95; 1.00] 4.1%
Keita (2022) -Six Japanese prefectures -0.0305 0.0263 0.97 [0.92; 1.02] 4.1%
Chen (2021) -Guangdong, China -0.0619 0.0081 0.94 [0.93; 0.96] 4.1%
Sung (2022) -Changwon, Korea -0.0101 0.0026 0.99 [0.98; 1.00] 4.1%
Lisa (2022) -Helsinki, Finland 1.3737 0.1613 3.95 [2.88; 5.42] 4.0%
Kong(2020) -Wuhan, China 2.3273 1.0218 10.25 [1.38; 75.94] 2.1%
Simone (2020) -Florence, Italy -1.0498 0.3837 0.35 [0.16; 0.74] 3.6%
Simone (2020) -Florence, Italy -1.3093 0.3930 0.27 [0.12; 0.58] 3.6%
Simone (2020) -Florence, Italy -1.3863 0.3963 0.25 [0.11; 0.54] 3.6%
Simone (2020) -Trento, Italy -1.5141 0.3837 0.22 [0.10; 0.47] 3.6%
Simone (2020) -Trento, Italy -1.3863 0.3963 0.25 [0.11; 0.54] 3.6%
Simone (2020) -Trento, Italy -1.3471 0.3837 0.26 [0.12; 0.55] 3.6%
Random effects model 0.54 [0.33; 0.89] 81.0%
Prediction interval [0.05; 5.86]
Heterogeneity: I^2 = 100%, τ^2 = 1.2263, p = 0
Climate = Continental
Simone (2020) -Milan, Italy -1.3471 0.3837 0.26 [0.12; 0.55] 3.6%
Simone (2020) -Milan, Italy -1.3863 0.3963 0.25 [0.11; 0.54] 3.6%
Simone (2020) -Milan, Italy -1.4271 0.3863 0.24 [0.11; 0.51] 3.6%
Random effects model 0.25 [0.16; 0.39] 10.8%
Prediction interval [0.01; 4.32]
Heterogeneity: I^2 = 0%, τ^2 = 0, p = 0.99
Random effects model 0.52 [0.35; 0.78] 100.0%
Prediction interval [0.06; 4.52]
0.1 0.5 1 2 10
Reduced risk Increased risk
Heterogeneity: I^2 = 100%, τ^2 = 1,0512, p = 0
Test for subgroup differences: χ^2_2 = 33.51, df = 2 (p < 0.01)

Figure S9-2. Subgroup analysis of severe acute respiratory syndrome coronavirus 2 (SARS-CoV-2) meta-analysis by World Bank income category

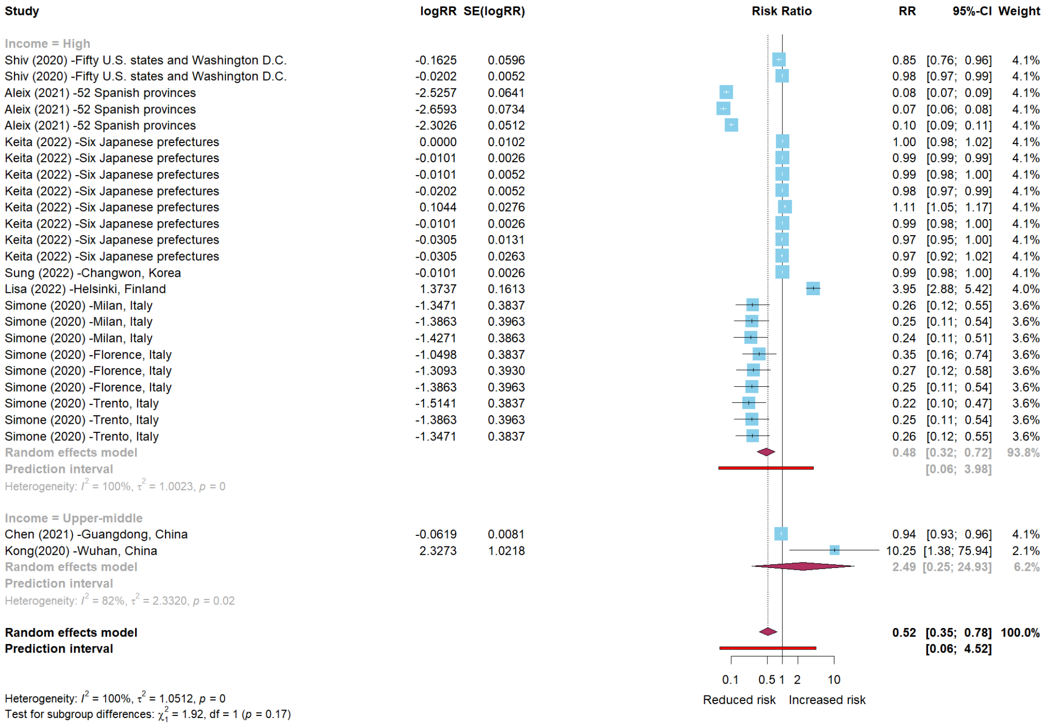

Figure S9-3. Subgroup analysis of severe acute respiratory syndrome coronavirus 2 (SARS-CoV-2) meta-analysis by temporal resolution

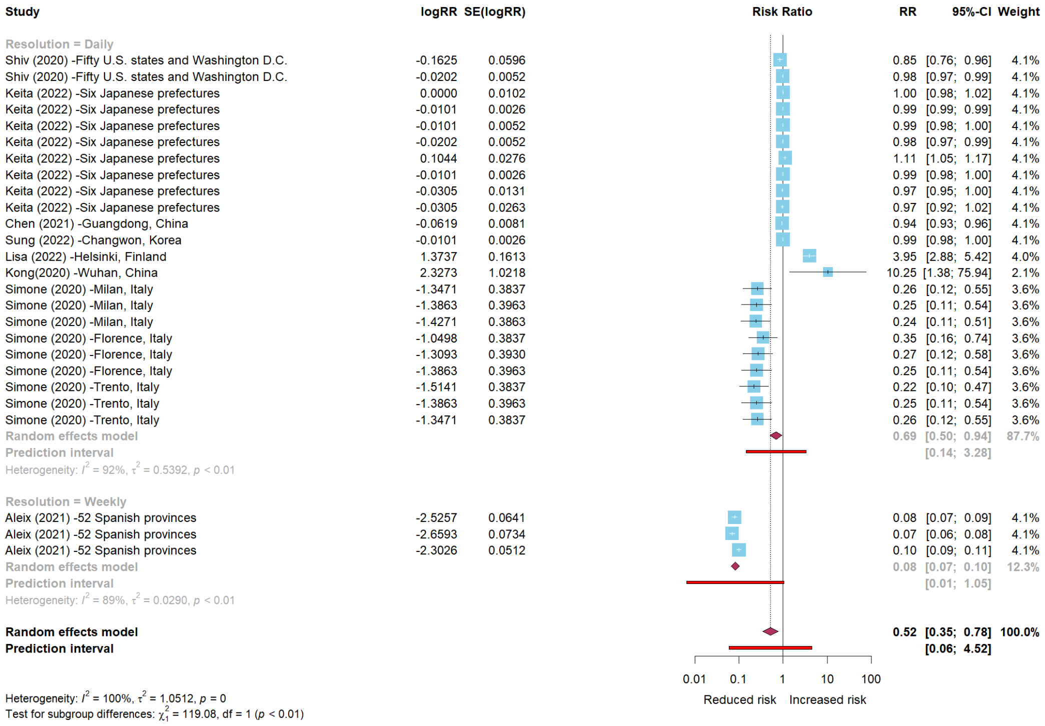

Classified as ECDC NORMAL

Figure S9-4. Subgroup analysis of severe acute respiratory syndrome coronavirus 2 (SARS-CoV-2) meta-analysis by exposure measure

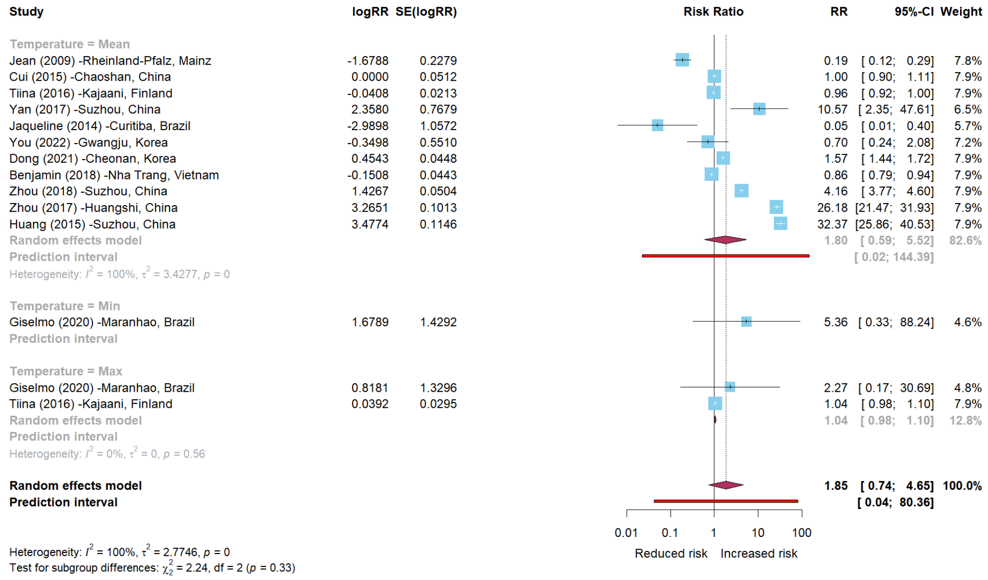

Figure S9-5. Subgroup analysis of severe acute respiratory syndrome coronavirus 2 (SARS-CoV-2) meta-analysis by modelling approach

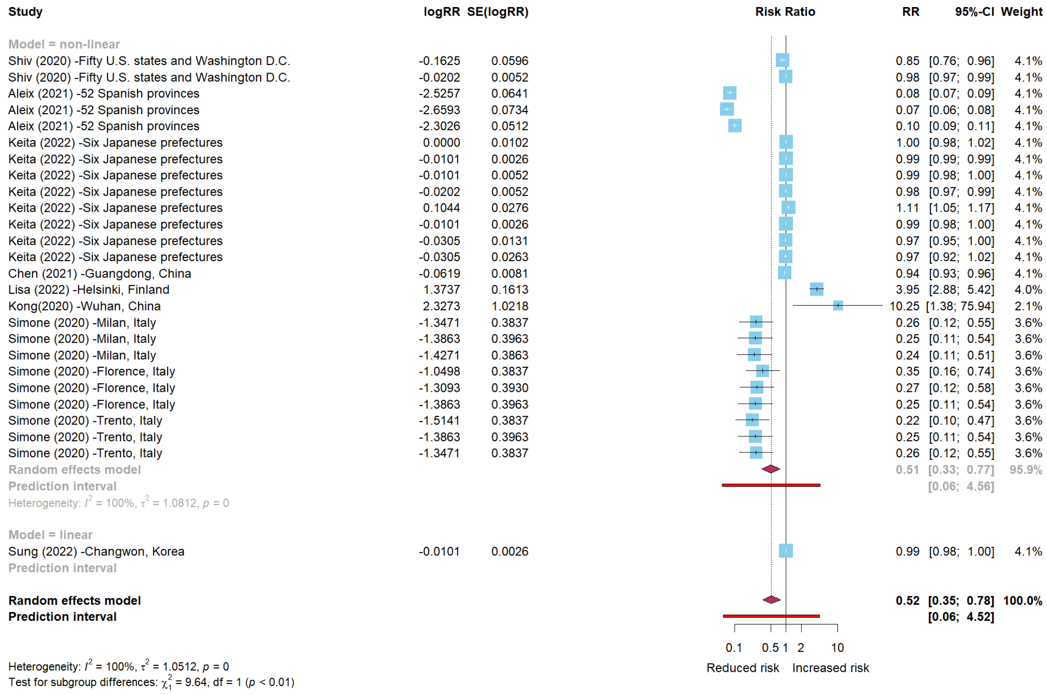

Classified as ECDC NORMAL

Figure S9-6. Subgroup analysis of severe acute respiratory syndrome coronavirus 2 (SARS-CoV-2) meta-analysis by lag type

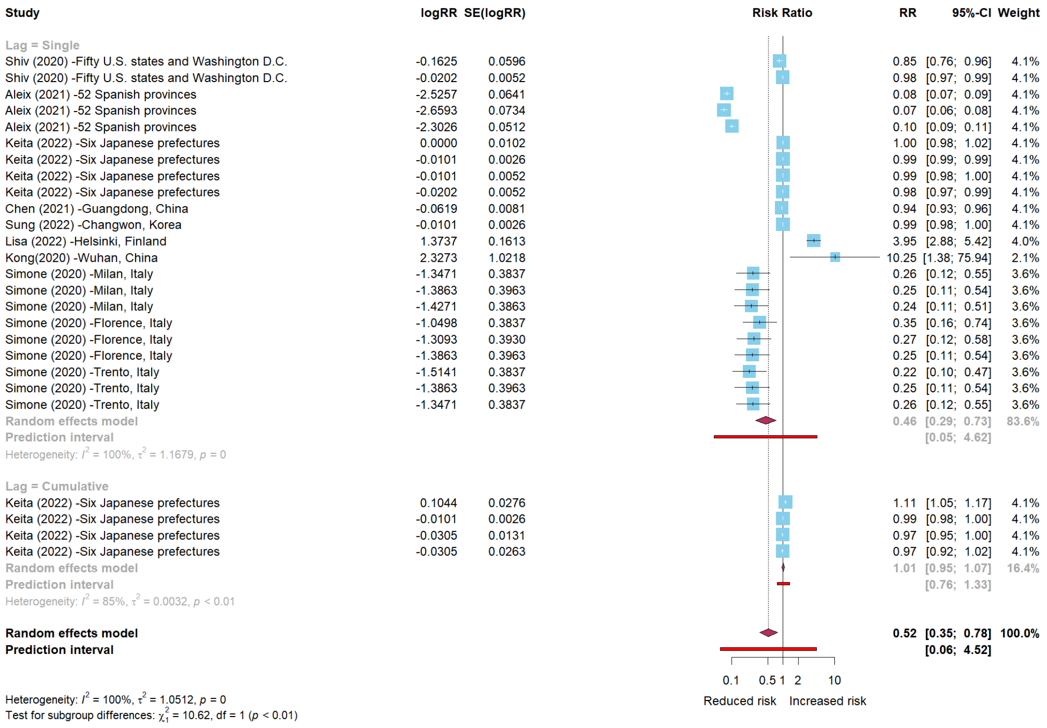

Figure S9-7. Leave-one-out analysis of severe acute respiratory syndrome coronavirus 2 (SARS-CoV-2) meta-analysis

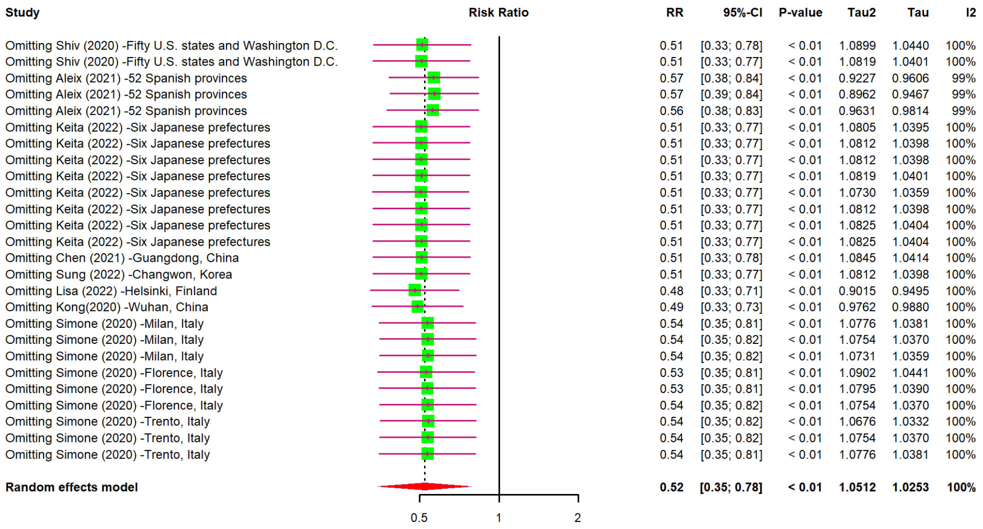

Classified as ECDC NORMAL

Figure S10. Random-effects meta-analysis of severe acute respiratory syndrome coronavirus (SARS-CoV) estimates (four studies)

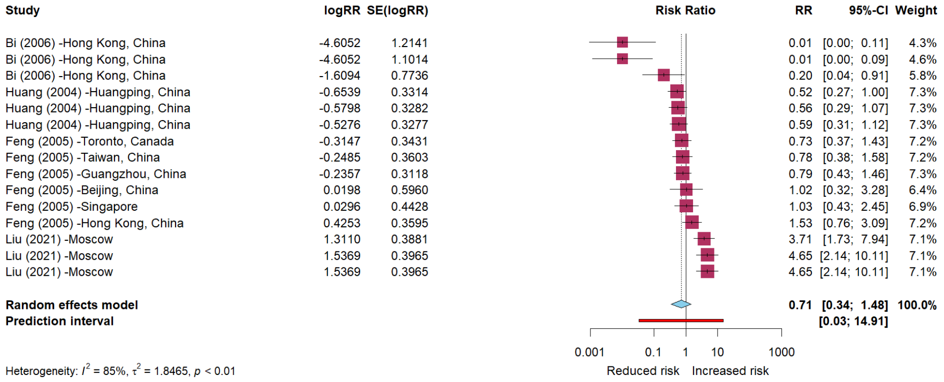

Figure S10-1. Subgroup analysis of severe acute respiratory syndrome (SARS) meta-analysis by Köppen-Geiger climate

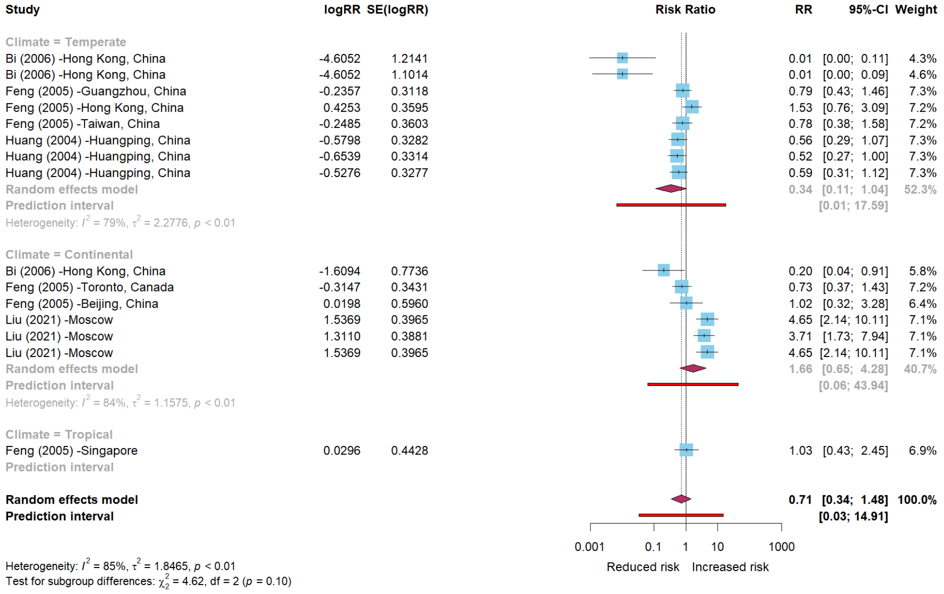

Classified as ECDC NORMAL

Figure S10-2. Subgroup analysis of severe acute respiratory syndrome coronavirus (SARS-CoV) meta-analysis by World Bank income category

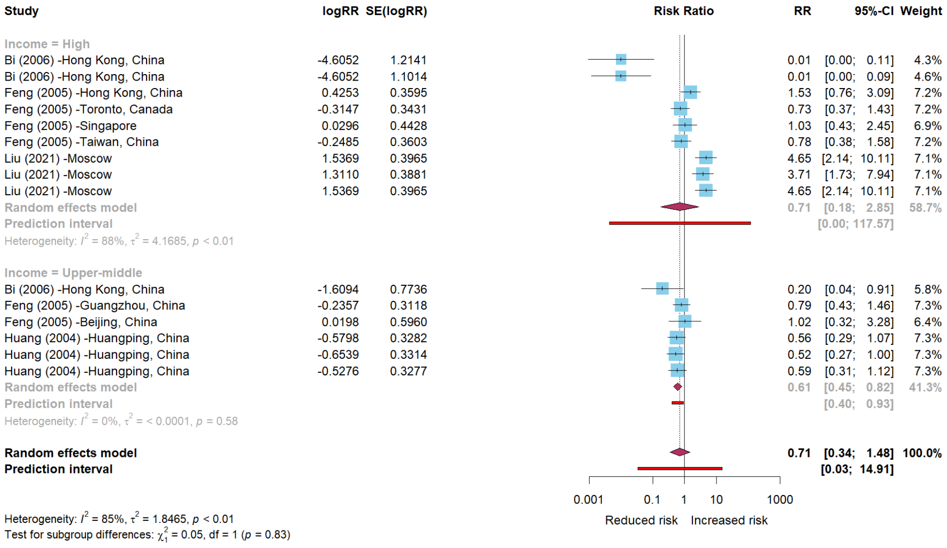

Figure S10-3. Subgroup analysis of severe acute respiratory syndrome coronavirus (SARS-CoV) meta-analysis by temporal resolution

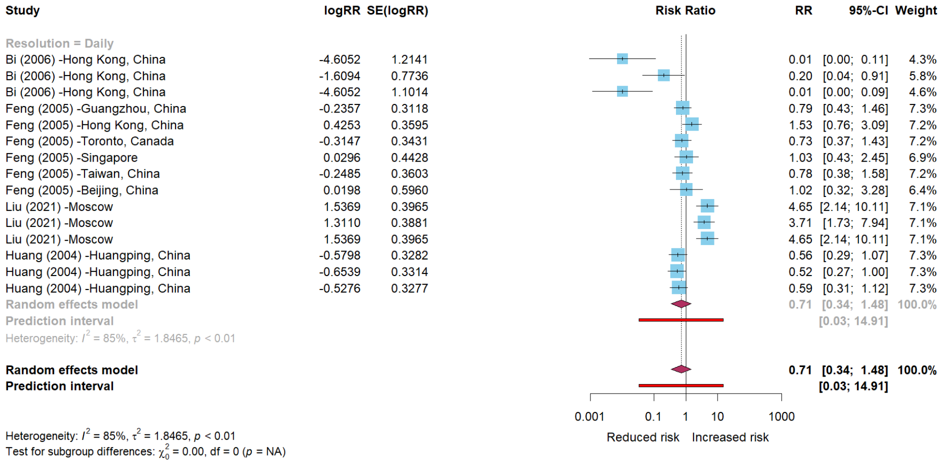

Figure S10-4. Subgroup analysis of severe acute respiratory syndrome coronavirus (SARS-CoV) meta-analysis by exposure measure

Classified as ECDC NORMAL

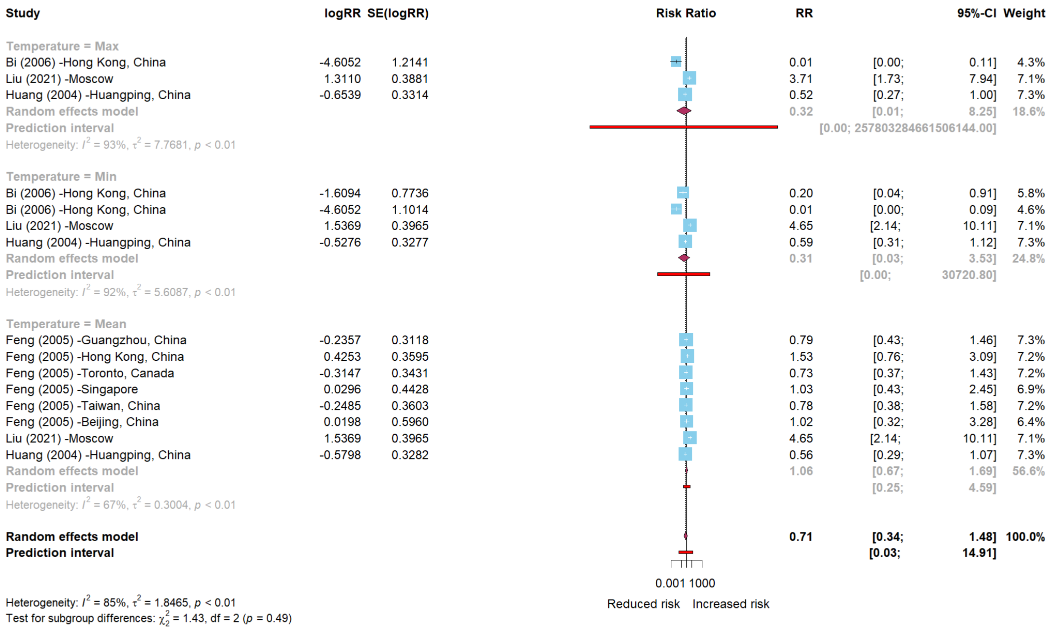

Figure S10-5. Subgroup analysis of severe acute respiratory syndrome coronavirus (SARS-CoV) meta-analysis by modelling approach

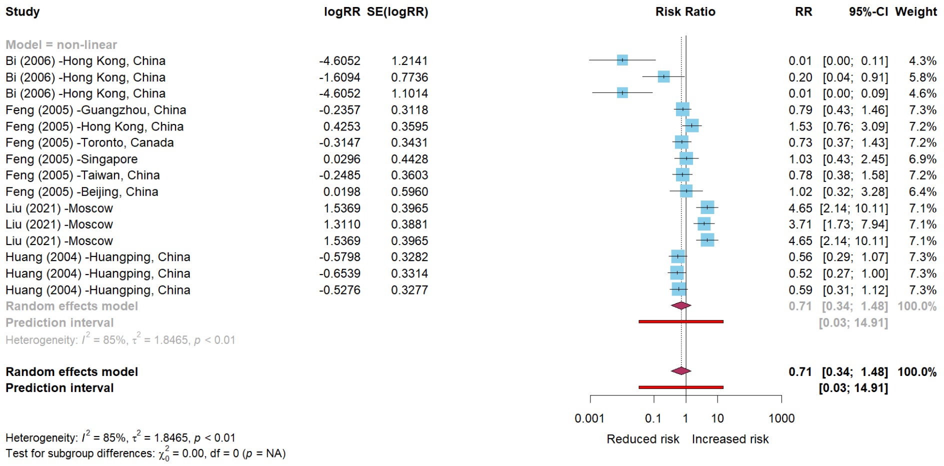

Figure S10-6. Leave-one-out analysis of severe acute respiratory syndrome coronavirus (SARS-CoV) meta-analysis

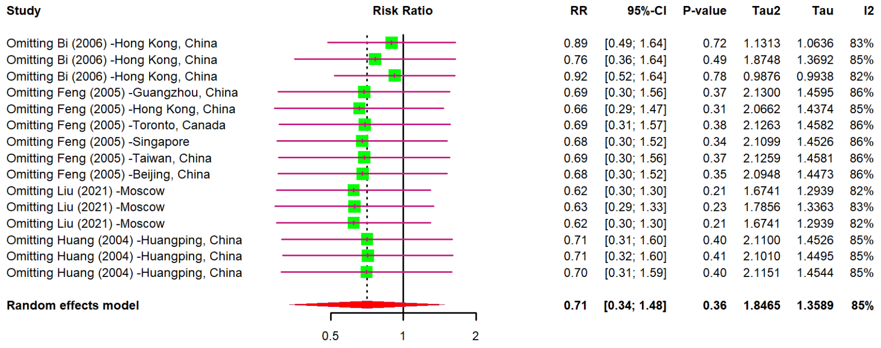

Figure S11. Random-effects meta-analysis of human coronavirus (HCoV) estimates (five studies)

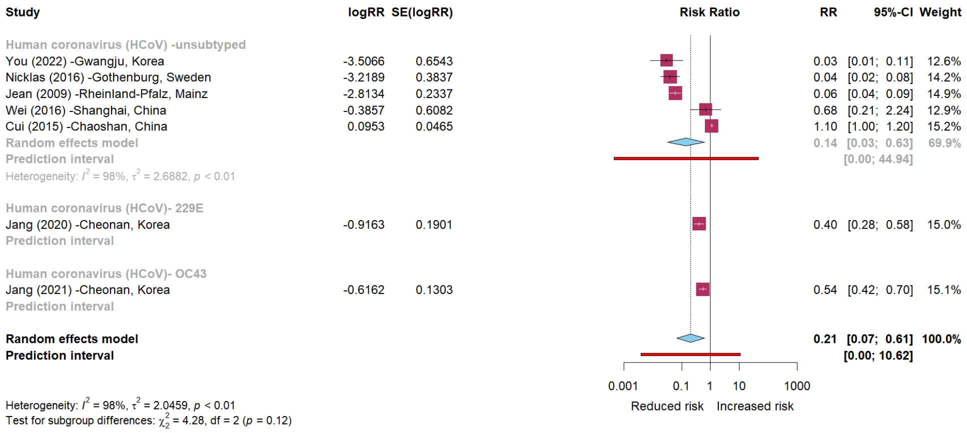

Figure S11-1. Subgroup analysis of human coronavirus (HCoV) meta-analysis by Köppen-Geiger climate

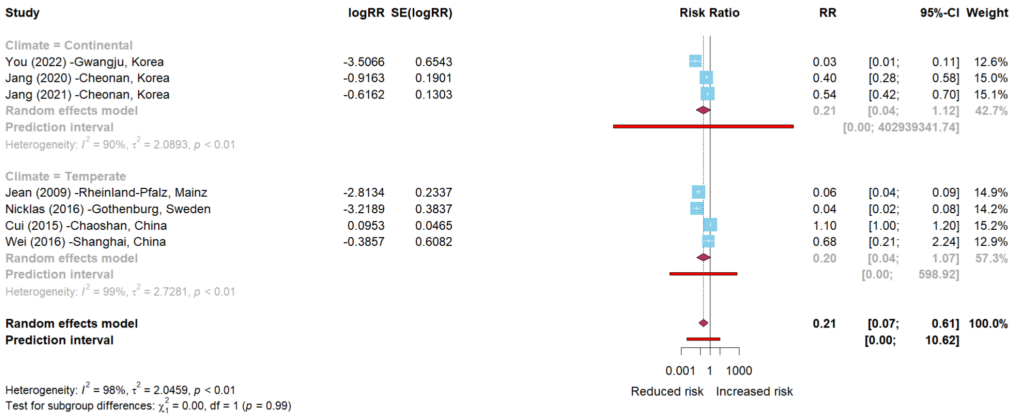

Figure S11-2. Subgroup analysis of human coronavirus (HCoV) meta-analysis by World Bank income category

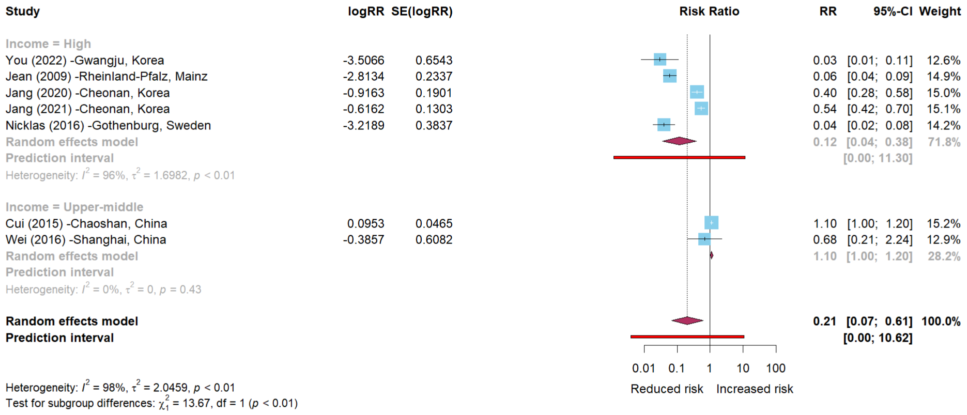

Figure S11-3. Subgroup analysis of human coronavirus (HCoV) meta-analysis by temporal resolution

Classified as ECDC NORMAL

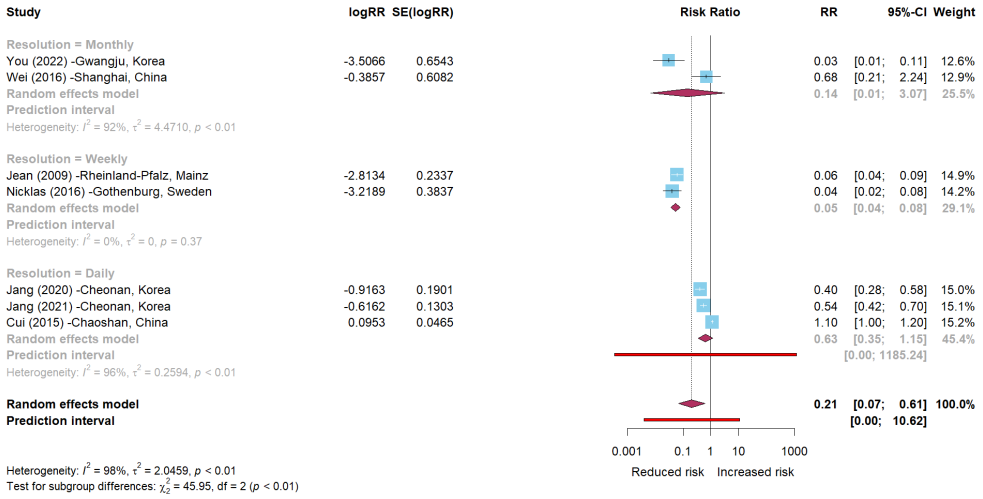

Figure S11-4. Subgroup analysis of human coronavirus (HCoV) meta-analysis by exposure measure

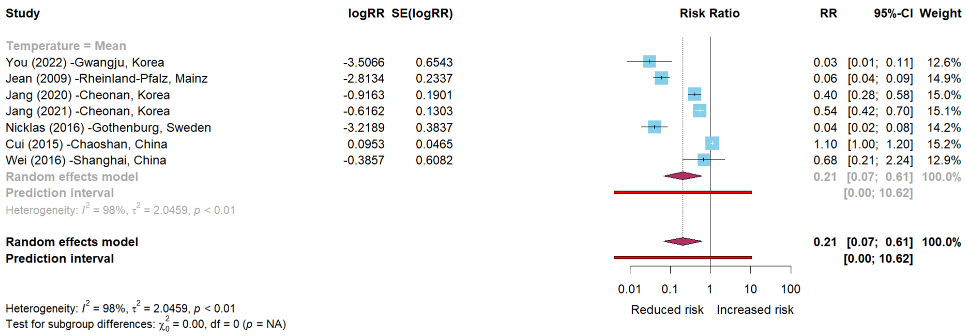

Figure S11-5. Subgroup analysis of human coronavirus (HCoV) meta-analysis by modelling approach

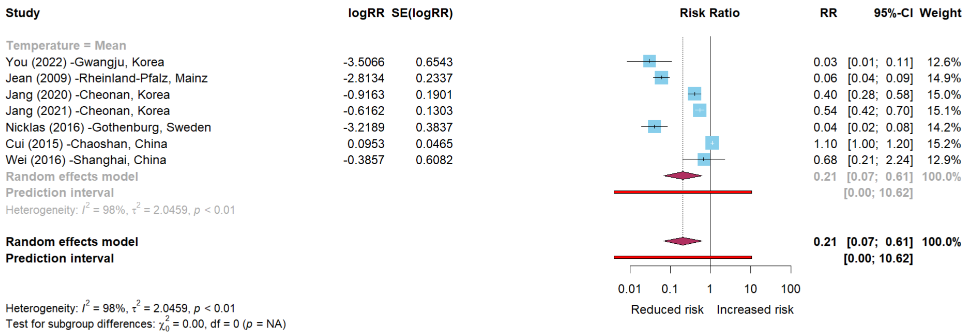

Figure S11-6. Leave-one-out analysis of human coronavirus (HCoV) meta-analysis

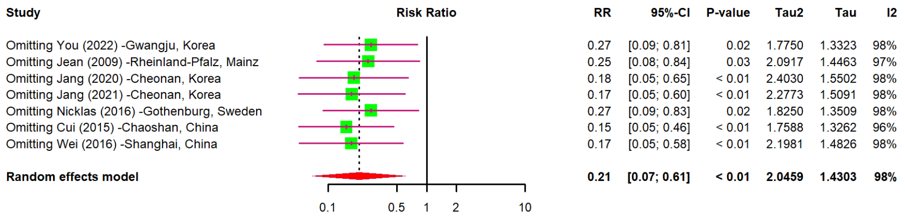

Figure S12. Random-effects meta-analysis of Human rhinoviruses/enteroviruses (HRVs/EVs) estimates (two studies)

Classified as ECDC NORMAL

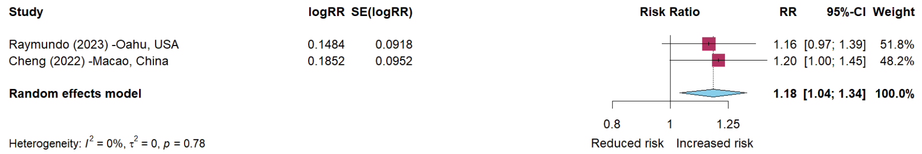

Figure S12-1. Subgroup analysis of Human rhinoviruses/enteroviruses (HRVs/EVs) meta-analysis by Köppen-Geiger climate

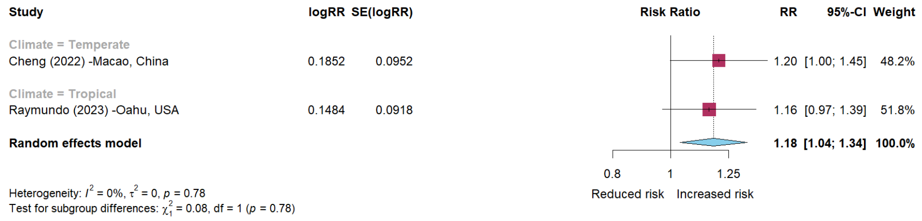

Figure S12-2. Subgroup analysis of Human rhinoviruses/enteroviruses (HRVs/EVs) meta-analysis by World Bank income category

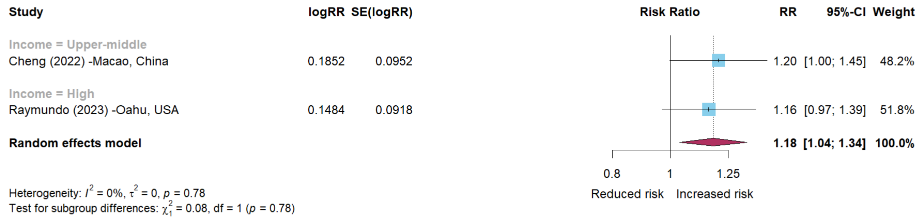

Figure S12-3. Subgroup analysis of Human rhinoviruses/enteroviruses (HRVs/EVs) meta-analysis by temporal resolution

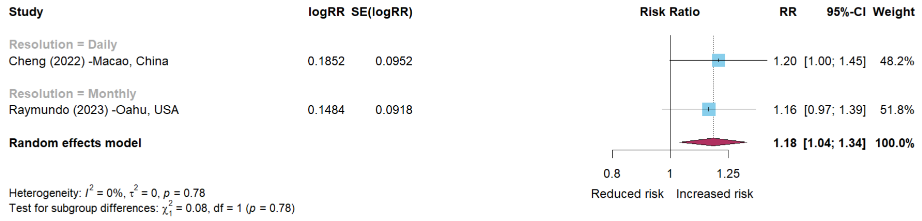

Figure S12-4. Subgroup analysis of Human rhinoviruses/enteroviruses (HRVs/EVs) meta-analysis by exposure measure

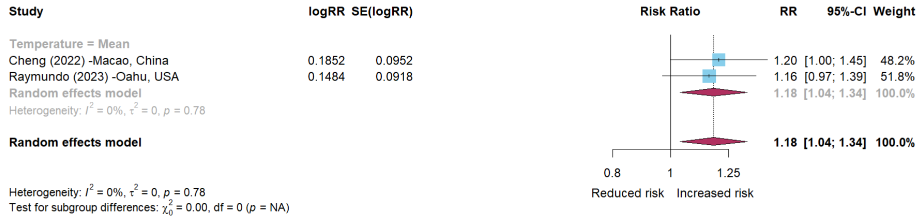

Figure S12-5. Subgroup analysis of Human rhinoviruses/enteroviruses (HRVs/EVs) meta-analysis by modelling approach

Classified as ECDC NORMAL

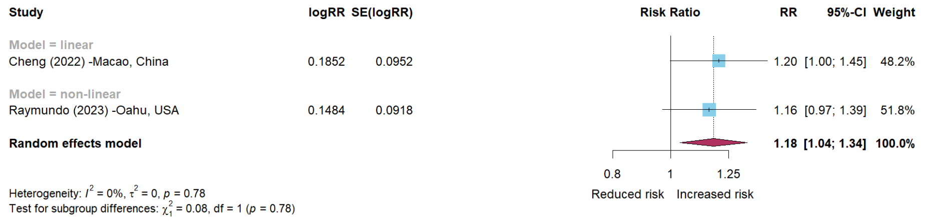

Figure S13. Subgroup analysis of bacterial respiratory infections meta-analysis by pathogen

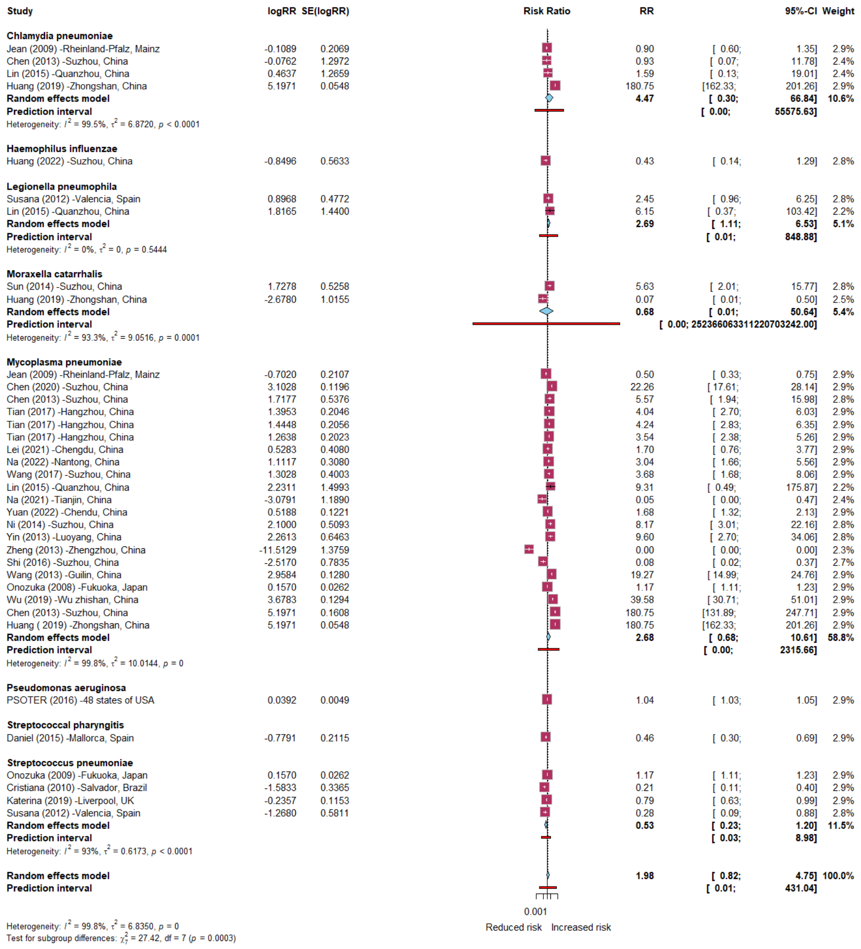

Figure S14. Random-effects meta-analysis of *Streptococcus pneumoniae* estimates (four studies)

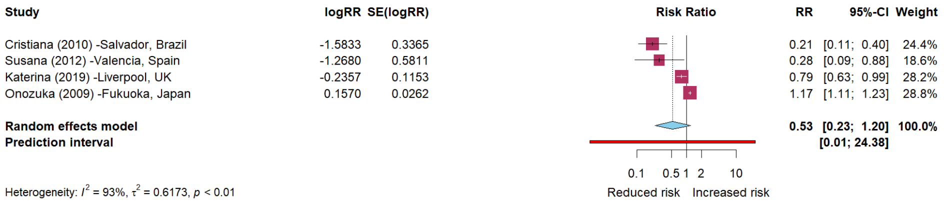

Figure S14-1. Subgroup analysis of *Streptococcus pneumoniae* meta-analysis by Köppen-Geiger climate

Classified as ECDC NORMAL

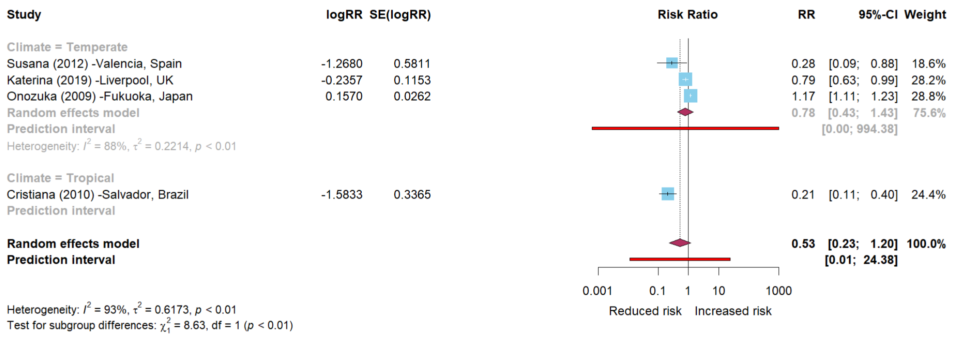

Figure S14-2. Subgroup analysis of *Streptococcus pneumoniae* meta-analysis by World Bank income category

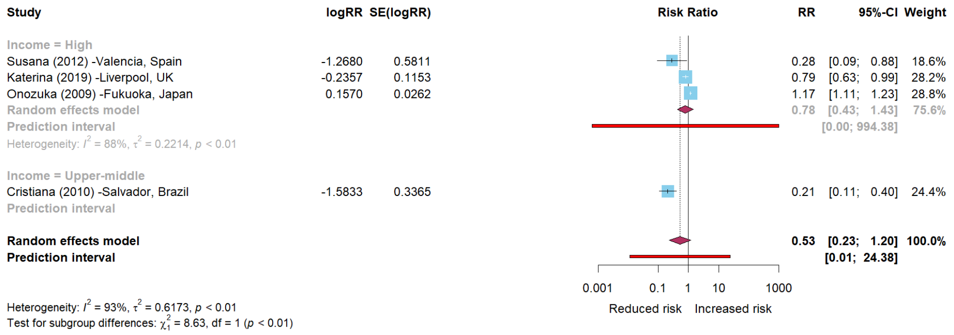

Figure S14-3. Subgroup analysis of *Streptococcus pneumoniae* meta-analysis by temporal resolution

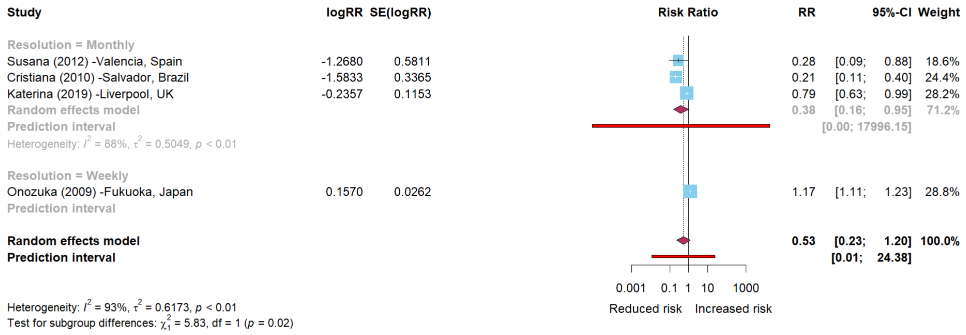

Figure S14-4. Subgroup analysis of *Streptococcus pneumoniae* meta-analysis by exposure measure

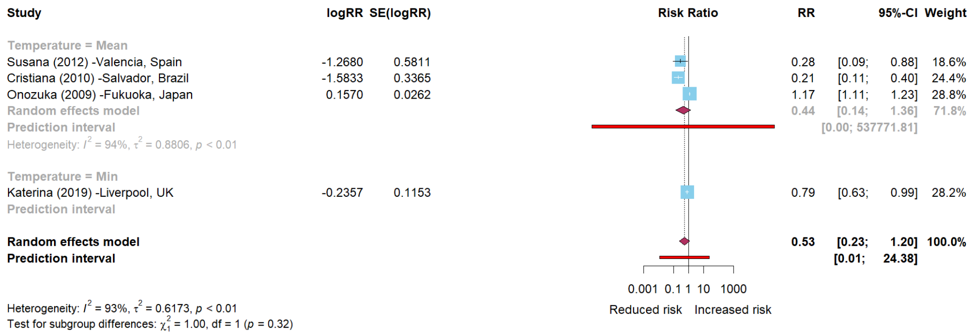

Figure S14-5. Subgroup analysis of *Streptococcus pneumoniae* meta-analysis by modelling approach

Classified as ECDC NORMAL

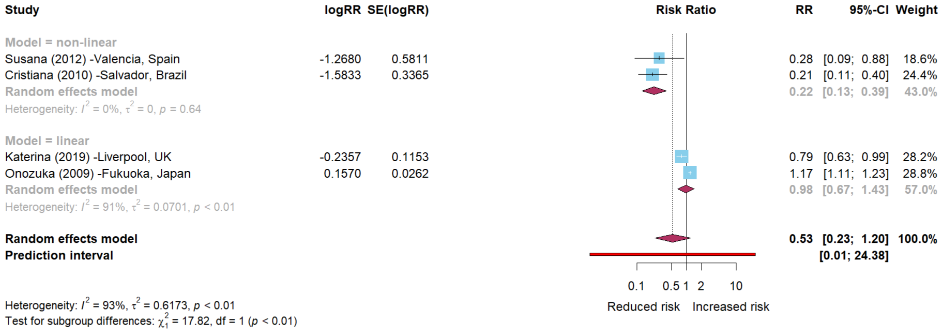

Figure S14-6. Leave-one-out analysis of *Streptococcus pneumoniae* meta-analysis

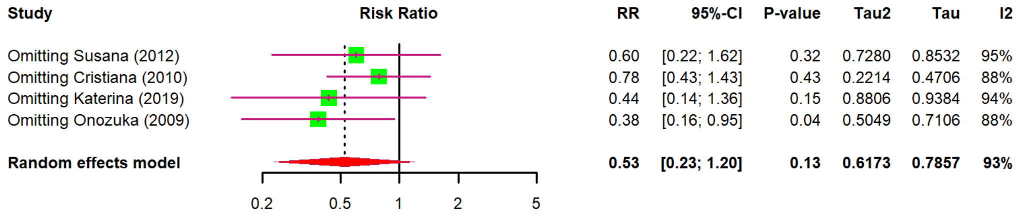

Figure S15. Random-effects meta-analysis of *Mycoplasma pneumoniae* estimates (19 studies)

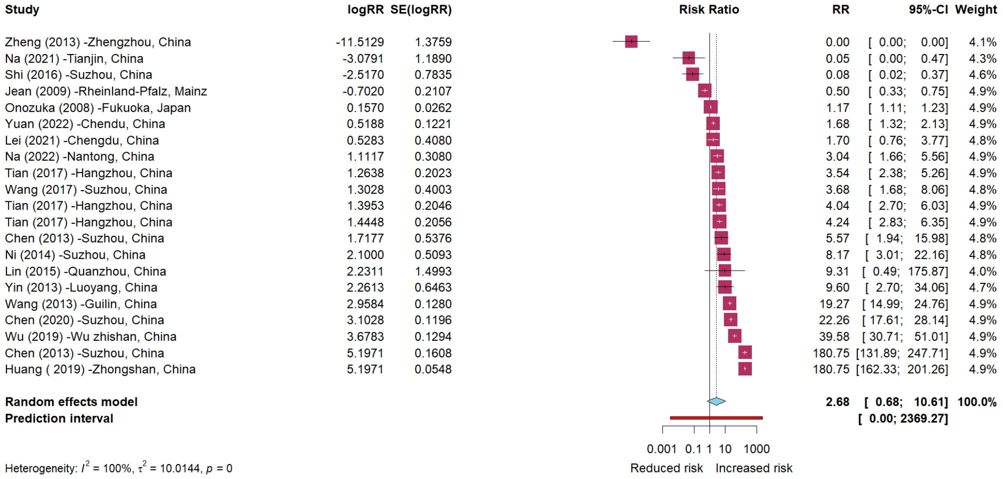

Figure S15-1. Subgroup analysis of *Mycoplasma pneumoniae* meta-analysis by Köppen-Geiger climate

Classified as ECDC NORMAL

Study	logRR	SE(logRR)	RR	95%-CI	Weight
**Climate = Temperate**					
Jean (2009) -Rheinland-Pfalz, Mainz	-0.7020	0.2107	0.50	[0.33; 0.75]	4.9%
Chen (2020) -Suzhou, China	3.1028	0.1196	22.26	[17.61; 28.14]	4.9%
Chen (2013) -Suzhou, China	5.1971	0.1608	180.75	[131.89; 247.71]	4.9%
Chen (2013) -Suzhou, China	1.7177	0.5376	5.57	[1.94; 15.98]	4.8%
Huang (2019) -Zhongshan, China	5.1971	0.0548	180.75	[162.33; 201.26]	4.9%
Tian (2017) -Hangzhou, China	1.3953	0.2046	4.04	[2.70; 6.03]	4.9%
Tian (2017) -Hangzhou, China	1.4448	0.2056	4.24	[2.83; 6.35]	4.9%
Tian (2017) -Hangzhou, China	1.2638	0.2023	3.54	[2.38; 5.26]	4.9%
Na (2022) -Nantong, China	1.1117	0.3080	3.04	[1.66; 5.56]	4.9%
Wang (2017) -Suzhou, China	1.3028	0.4003	3.68	[1.68; 8.06]	4.8%
Lei (2021) -Chengdu, China	0.5283	0.4080	1.70	[0.76; 3.77]	4.8%
Onozuka (2008) -Fukuoka, Japan	0.1570	0.0262	1.17	[1.11; 1.23]	4.9%
Yuan (2022) -Chendu, China	0.5188	0.1221	1.68	[1.32; 2.13]	4.9%
Lin (2015) -Quanzhou, China	2.2311	1.4993	9.31	[0.49; 175.87]	4.0%
Wang (2013) -Guilin, China	2.9584	0.1280	19.27	[14.99; 24.76]	4.9%
Ni (2014) -Suzhou, China	2.1000	0.5093	8.17	[3.01; 22.16]	4.8%
Random effects model			6.32	[2.74; 14.57]	77.3%
Prediction interval				[0.16; 248.64]	
Heterogeneity: I^2 = 100%, τ^2 = 2.7508, p = 0					
**Climate = Tropical**					
Wu (2019) -Wu zhishan, China	3.6783	0.1294	39.58	[30.71; 51.01]	4.9%
Prediction interval					
**Climate = Continental**					
Na (2021) -Tianjin, China	-3.0791	1.1890	0.05	[0.00; 0.47]	4.3%
Yin (2013) -Luoyang, China	2.2613	0.6463	9.60	[2.70; 34.06]	4.7%
Zheng (2013) -Zhengzhou, China	-11.5129	1.3759	0.00	[0.00; 0.00]	4.1%
Shi (2016) -Suzhou, China	-2.5170	0.7835	0.08	[0.02; 0.37]	4.6%
Random effects model			0.03	[0.00; 6.94]	17.8%
Prediction interval				[0.00; 13636514406.88]	
Heterogeneity: I^2 = 97%, τ^2 = 31.2086, p < 0.01					
**Random effects model**			**2.68**	**[0.68; 10.61]**	**100.0%**
**Prediction interval**				**[0.00; 2369.27]**	

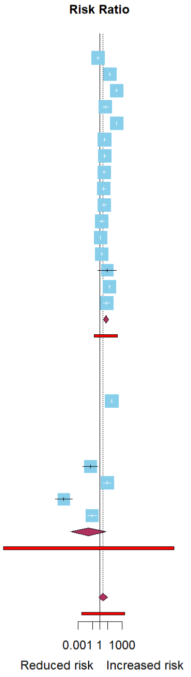

Heterogeneity: I^2 = 100%, τ^2 = 10.0144, p = 0
Test for subgroup differences: χ^2_2 = 23.30, df = 2 (p < 0.01)

Classified as ECDC NORMAL

Figure S15-2. Subgroup analysis of *Mycoplasma pneumoniae* meta-analysis by World Bank income category

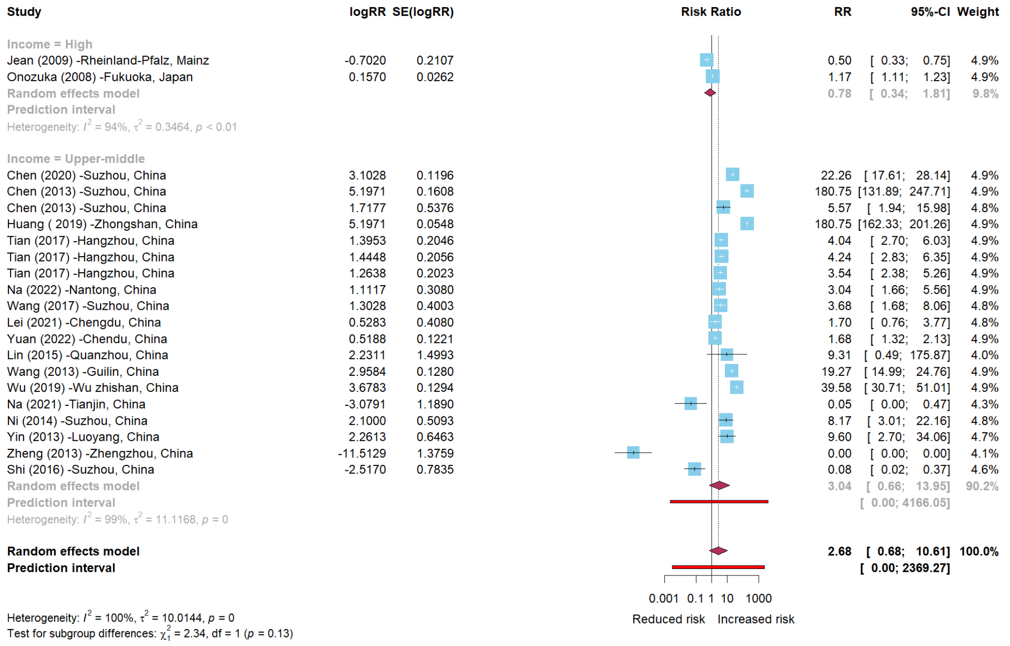

Figure S15-3. Subgroup analysis of *Mycoplasma pneumoniae* meta-analysis by temporal resolution

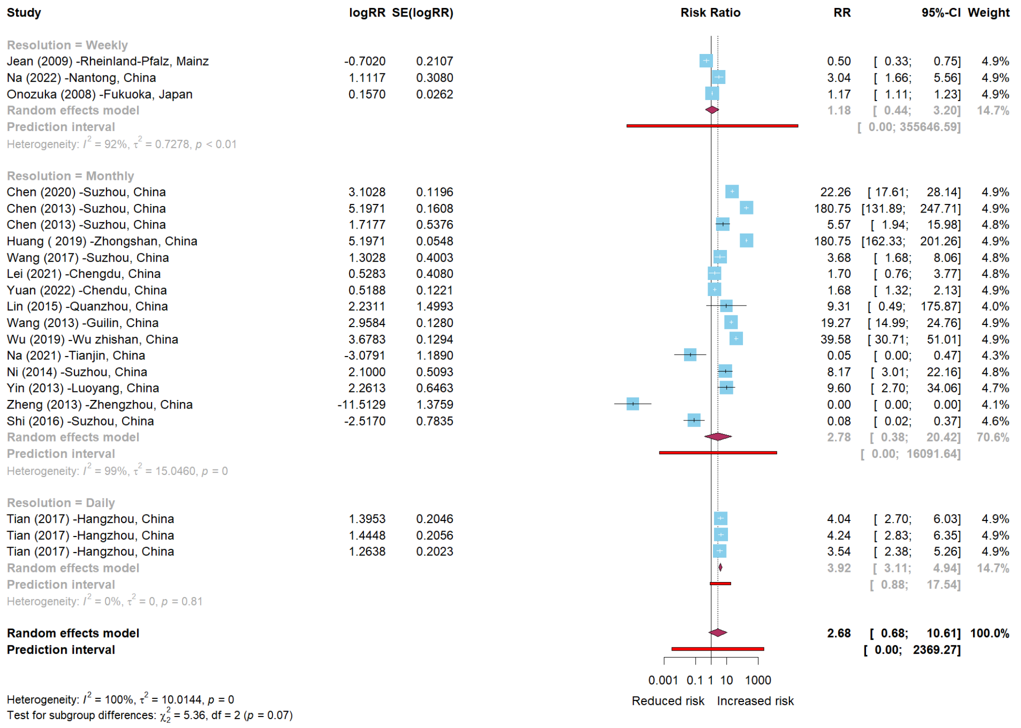

Classified as ECDC NORMAL

Figure S15-4. Subgroup analysis of *Mycoplasma pneumoniae* meta-analysis by exposure measure

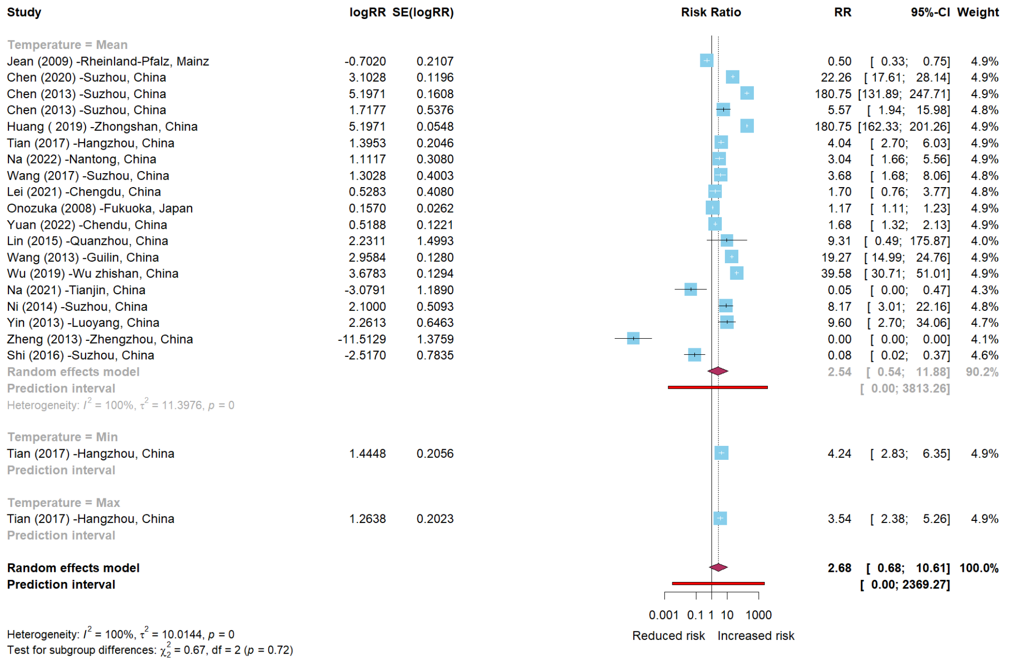

Figure S15-5. Subgroup analysis of *Mycoplasma pneumoniae* meta-analysis by modelling approach

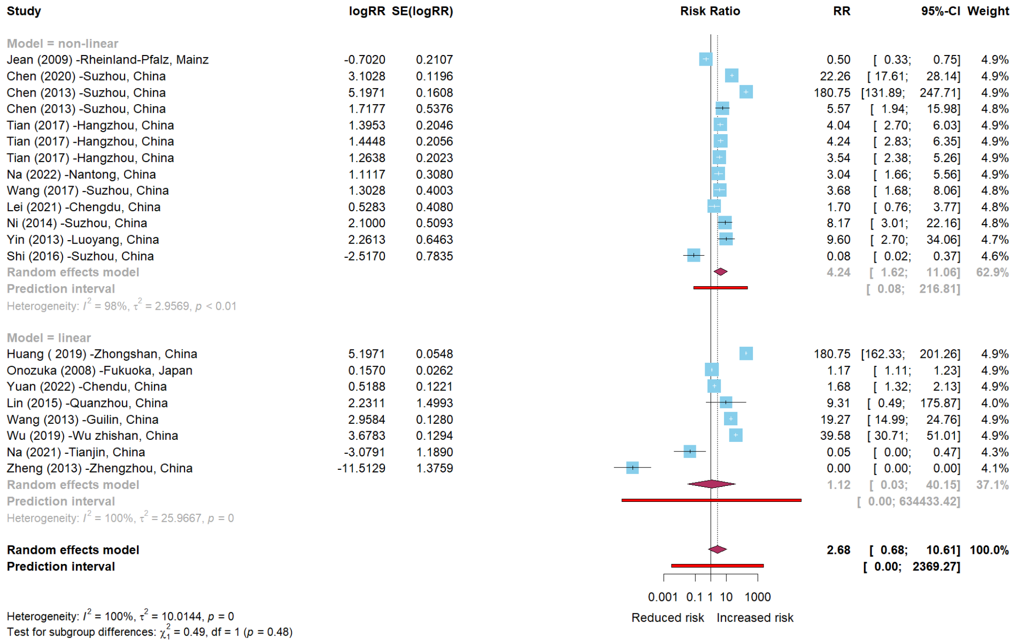

Classified as ECDC NORMAL

Figure S15-6. Leave-one-out analysis of *Mycoplasma pneumoniae* meta-analysis

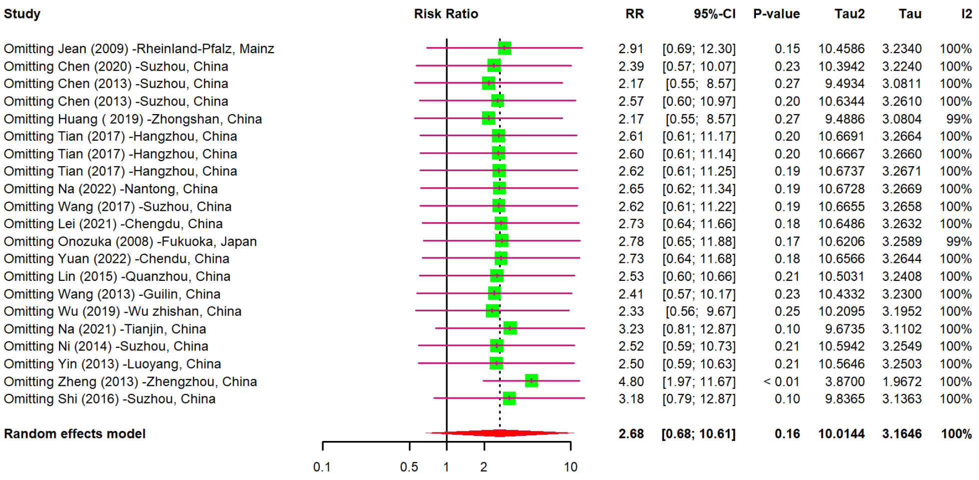

Figure S15-7. Funnel plot of *Mycoplasma pneumoniae* estimates

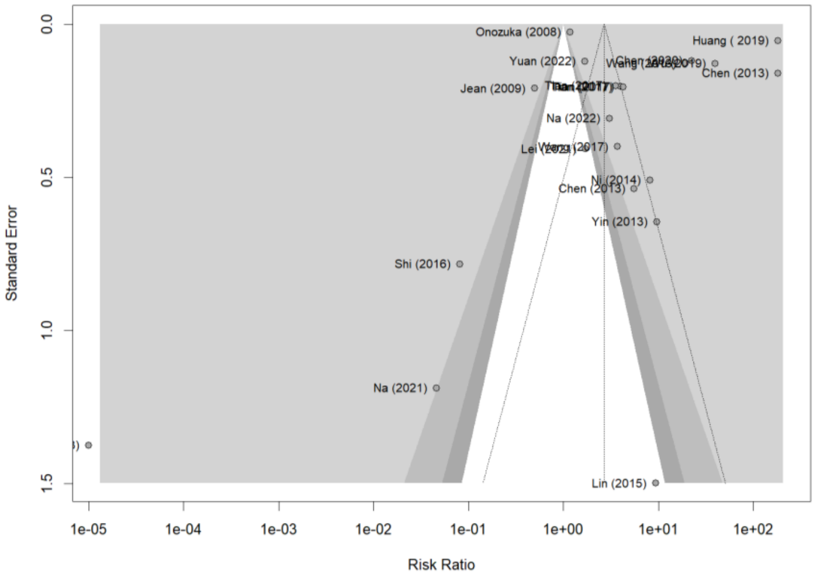

Linear regression test of funnel plot asymmetry

Test result: t = 0.86, df = 19, p-value = 0.4024

Sample estimates:

bias	se.bias	intercept	se.intercept
4.8989	5.7192	1.0285	0.5566

Figure S15-8. Trim and fill of *Mycoplasma pneumoniae* meta-analysis

Classified as ECDC NORMAL

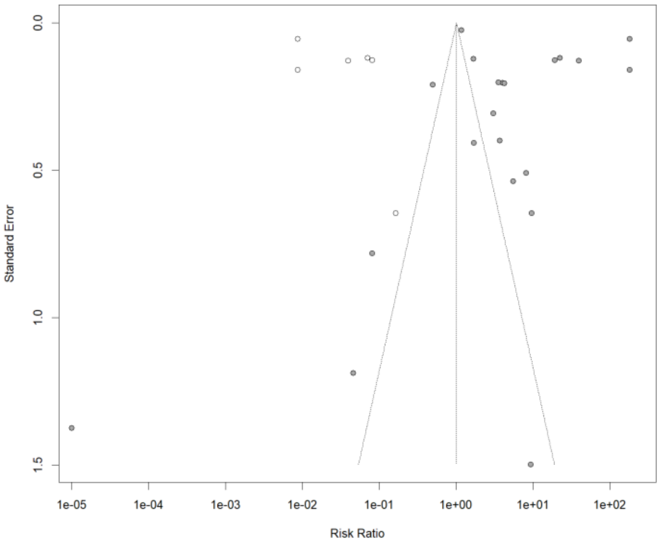

Number of studies: k = 27 (with 6 added studies)

Random effects model: RR= 1.0086 [0.2755; 3.6929], Z=0.01, P=0.9896

Quantifying heterogeneity: tau^2 = 11.5561 [7.1899; 23.2580]; tau = 3.3994 [2.6814; 4.8227], I^2 = 99.9%; H = 29.17

Figure S16. Random-effects meta-analysis of *Chlamydia pneumoniae* estimates (four studies)

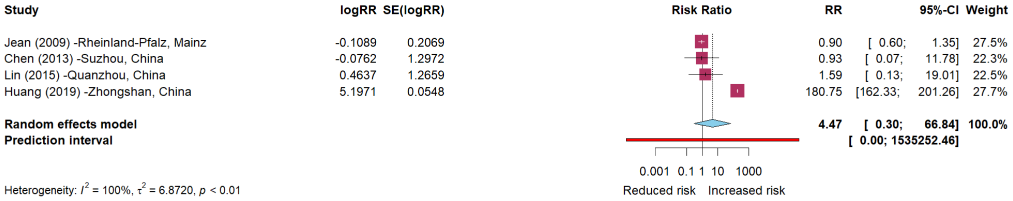

Figure S16-1. Subgroup analysis of *Chlamydia pneumoniae* meta-analysis by Köppen-Geiger climate

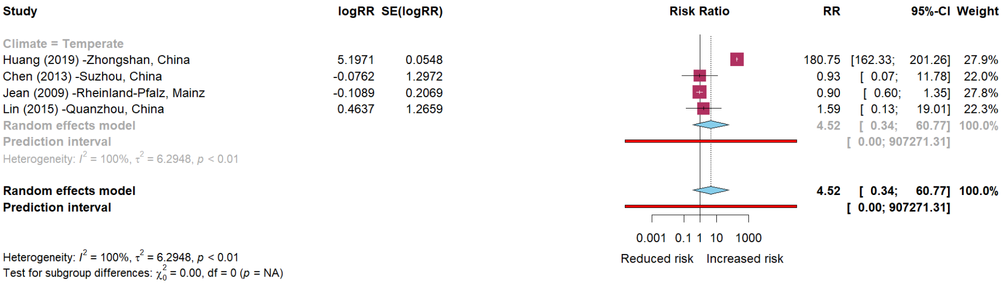

Figure S16-2. Subgroup analysis of *Chlamydia pneumonia* meta-analysis by World Bank income category

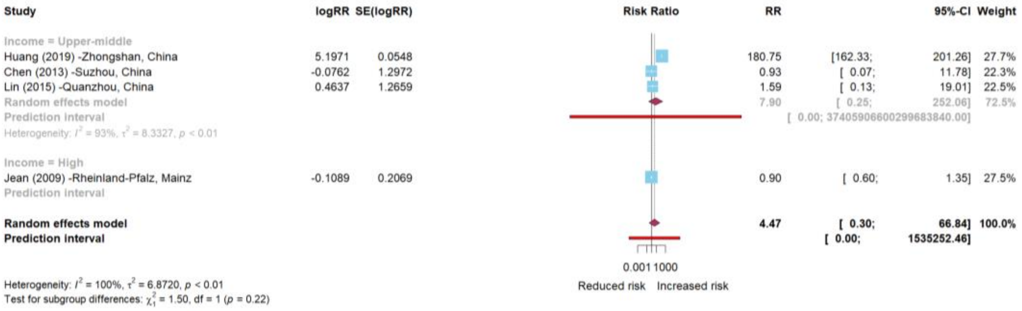

Classified as ECDC NORMAL

Figure S16-3. Subgroup analysis of *Chlamydia pneumoniae* meta-analysis by temporal resolution

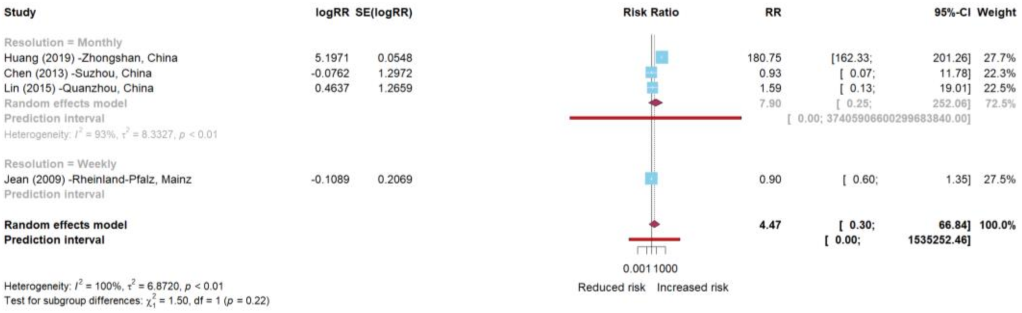

Figure S16-4. Subgroup analysis of *Chlamydia pneumoniae* meta-analysis by exposure measure

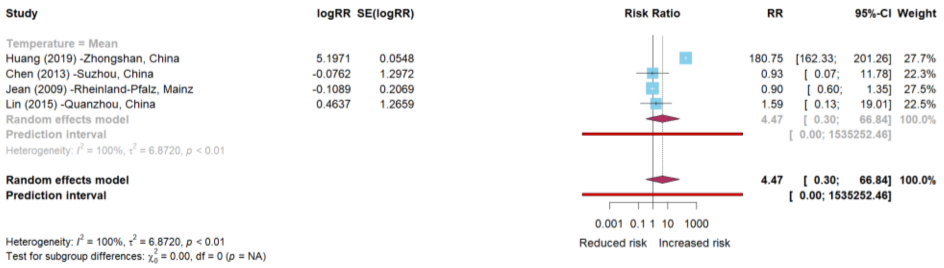

Figure S16-5. Subgroup analysis of *Chlamydia pneumoniae* meta-analysis by modelling approach

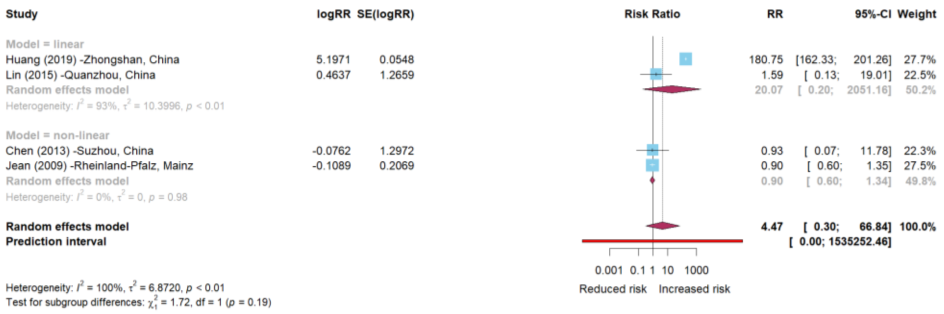

Figure S16-6. Leave-one-out analysis of *Chlamydia pneumoniae* meta-analysis

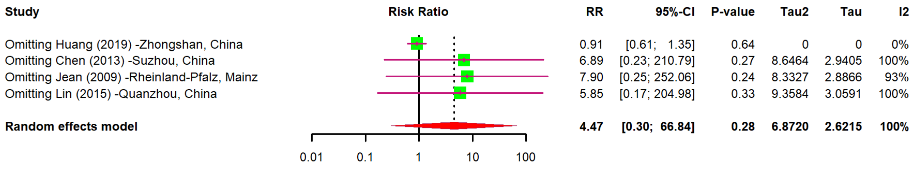

Figure S16-7. Sensitivity analysis by excluding study with high risk of bias for Huang et al 2019

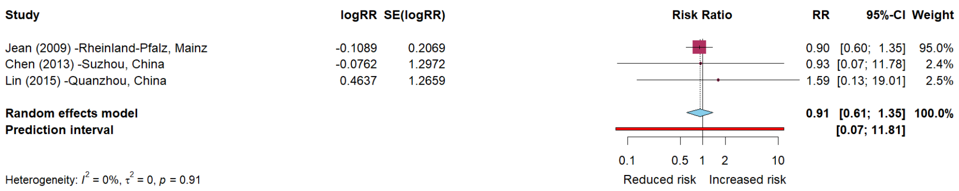

Classified as ECDC NORMAL

Table X1. Estimates of individual effects from 137 included studies

Study	Location	Cases	Pathogen	RR	lower	upper
Santiago (2004)	Leon, Spain	221	Respiratory syncytial virus -unsubtyped	0.4588	0.2859	0.7274
Santiago (2004)	Leon, Spain	221	Respiratory syncytial virus -unsubtyped	0.5767	0.362	0.9113
Santiago (2004)	Leon, Spain	221	Respiratory syncytial virus -unsubtyped	0.5149	0.3221	0.8159
Zhang (2013)	Suzhou, China	42664	Respiratory syncytial virus -unsubtyped	0.004	0.001	0.011
Liu (2019)	Guangzhou, China	11398	Respiratory syncytial virus -unsubtyped	14.16	5.66	39.99
Natalia (2016)	Athens, Greece	7516	Respiratory syncytial virus -unsubtyped	0.76	0.74	0.79
Natalia (2016)	Athens, Greece	7516	Respiratory syncytial virus -unsubtyped	0.72	0.68	0.8
Silvia (2013)	Bologna, Italy	327	Respiratory syncytial virus -unsubtyped	0.3196	0.1958	0.5348
Omer (2008)	Lombok, Indonesia	2878	Respiratory syncytial virus -unsubtyped	1.44	1.24	1.66
Omer (2008)	Lombok, Indonesia	2878	Respiratory syncytial virus -unsubtyped	1.39	1.22	1.5
Virginia (2015)	Nine states, United States	Not reported	Respiratory syncytial virus -unsubtyped	0.0078	0.0036	0.0159
Patrick (2015)	Kuala Lumpur, Malaysia	5691	Respiratory syncytial virus -unsubtyped	0.2253	0.127	0.3878
Patrick (2015)	Kuala Lumpur, Malaysia	2561	Respiratory syncytial virus -unsubtyped	0.2937	0.1682	0.5002
Patrick (2015)	Kuala Lumpur, Malaysia	2959	Respiratory syncytial virus -unsubtyped	0.2151	0.1209	0.3711
Hailin (2019)	Wenzhou, China	89898	Respiratory syncytial virus -unsubtyped	0.0327	0.0125	0.0764
Zheng (2014)	Jiangsu, China	42104	Respiratory syncytial virus -unsubtyped	0.0039	0.0011	0.0113
Ilada (2020)	Bangkok, Thailand	8209	Respiratory syncytial virus -unsubtyped	0.6215	0.2767	1.3652
Nicklas (2016)	Gothenburg, Sweden	20062	Respiratory syncytial virus -unsubtyped	0.0012	0.0003	0.0039
Jean-Baptist (2009)	Rheinland-Pfalz, Mainz	3044	Respiratory syncytial virus -unsubtyped	0.0144	0.0075	0.0261
Noyola (2009)	San Luis Potosi, Mexico	1393	Respiratory syncytial virus -unsubtyped	0.32	0.04	1.3
Noyola (2009)	San Luis Potosi, Mexico	1393	Respiratory syncytial virus -unsubtyped	2.55	1.34	5.63
Rodriguez (2015)	Bogota, Colombia	3931	Respiratory syncytial virus -unsubtyped	3.14	1.56	6.31
Zhi (2021)	Suzhou, China	1157	Respiratory syncytial virus -A	0.0726	0.0131	0.2951
Zhi (2021)	Suzhou, China	1157	Respiratory syncytial virus -B	0.0792	0.0146	0.318
Gamba (2016)	Bogota, Colombia	3931	Respiratory syncytial virus -unsubtyped	2.25	1.11	4.53
Cheng (2022)	Macao, China	4880	Respiratory syncytial virus -A	0.7982	0.6621	0.9618

Classified as ECDC NORMAL

Cheng (2022)	Macao, China	4880	Respiratory syncytial virus -B	1.2346	1.0247	1.4884
Chee (2023)	Kuala Lumpur, Malaysia	2950	Respiratory syncytial virus -unsubtyped	1.1605	1.022	3.0255
Chen (2014)	Suzhou, China	998	Respiratory syncytial virus -unsubtyped	0.0002	0.0001	0.0054
Liu (2021)	Chongqing, China	3107	Respiratory syncytial virus -unsubtyped	1.93	1.08	3.46
Liu (2021)	Chongqing, China	3107	Respiratory syncytial virus -unsubtyped	0.87	0.77	0.98
Liu (2021)	Chongqing, China	3107	Respiratory syncytial virus -unsubtyped	1.14	1.03	1.26
Liu (2021)	Chongqing, China	3107	Respiratory syncytial virus -unsubtyped	1.06	1.02	1.09
Liu (2021)	Chongqing, China	3107	Respiratory syncytial virus -unsubtyped	0.93	0.87	0.98
Liu (2021)	Chongqing, China	3107	Respiratory syncytial virus -unsubtyped	0.4395	0.3896	0.4955
Liu (2021)	Chongqing, China	3107	Respiratory syncytial virus -unsubtyped	0.4413	0.3911	0.4974
Liu (2021)	Chongqing, China	3107	Respiratory syncytial virus -unsubtyped	0.4395	0.3896	0.4955
Giselmo (2020)	Maranhao, Brazil	151	Respiratory syncytial virus -unsubtyped	0.5348	0.0353	6.145
Giselmo (2020)	Maranhao, Brazil	151	Respiratory syncytial virus -unsubtyped	0.0109	0.0001	0.2325
Rory (2019)	Edinburgh, UK	52060	Respiratory syncytial virus -unsubtyped	0.827	0.808	0.847
Tian (2017)	Hangzhou, China	36500	Respiratory syncytial virus -unsubtyped	0.0074	0.0038	0.0136
Tian (2017)	Hangzhou, China	36500	Respiratory syncytial virus -unsubtyped	0.0122	0.0066	0.0215
Tian (2017)	Hangzhou, China	36500	Respiratory syncytial virus -unsubtyped	0.0288	0.0168	0.0478
Cui (2015)	Chaoshan, China	1074	Respiratory syncytial virus -unsubtyped	0.51	0.5	0.6
Lu (2015)	Suzhou, China	1803	Respiratory syncytial virus -unsubtyped	0.0054	0.0009	0.0241
Cristiana (2010)	Salvador, Brazil	184	Respiratory syncytial virus -unsubtyped	0.2053	0.1042	0.3873
Maria (2009)	Kathmandu, Nepal	887	Respiratory syncytial virus -unsubtyped	0.81	0.2305	2.7781
Magali (2018)	Dijon, France	4300	Respiratory syncytial virus -unsubtyped	0.5987	0.5365	0.6679
Magali (2018)	Dijon, France	4300	Respiratory syncytial virus -unsubtyped	0.7205	0.646	0.8033
Therese (2021)	Nairobi, Kenya	17261	Respiratory syncytial virus -unsubtyped	1.68	1.03	2.76
Therese (2021)	Nairobi, Kenya	17261	Respiratory syncytial virus -unsubtyped	1.61	0.95	2.73
Li (2013)	Zhuhai, China	924	Respiratory syncytial virus -unsubtyped	0.0288	0.0004	0.4608
Daniel (2012)	Mallorca, Spain	2384	Respiratory syncytial virus -unsubtyped	0.0109	0.0039	0.0266
Daniel (2012)	Mallorca, Spain	2384	Respiratory syncytial virus -unsubtyped	0.0126	0.0046	0.0302

Classified as ECDC NORMAL

Daniel (2012)	Mallorca, Spain	2384	Respiratory syncytial virus -unsubtyped	0.0067	0.0023	0.0173
Daniel (2012)	Mallorca, Spain	2384	Respiratory syncytial virus -unsubtyped	0.0003	0.0002	0.0007
Daniel (2012)	Mallorca, Spain	2384	Respiratory syncytial virus -unsubtyped	0.0006	0.0003	0.0011
Daniel (2012)	Mallorca, Spain	2384	Respiratory syncytial virus -unsubtyped	0.0002	0.0001	0.0004
Asma (2008)	Doha, Qatar	3121	Respiratory syncytial virus -unsubtyped	0.0188	0.0045	0.0622
Raffaella (2017)	Rome, Italy	723	Respiratory syncytial virus -unsubtyped	0.1527	0.0733	0.3008
Sheikh (2020)	Bukit Timah, Singapore	9905	Respiratory syncytial virus -unsubtyped	1.12	1.16	1.24
Sheikh (2020)	Bukit Timah, Singapore	9905	Respiratory syncytial virus -unsubtyped	1.04	1.12	1.16
Sheikh (2020)	Bukit Timah, Singapore	9905	Respiratory syncytial virus -unsubtyped	1.24	1.19	1.44
Ines (2018)	Sousse, Northern Africa	5131	Respiratory syncytial virus -unsubtyped	0.0109	0.0041	0.0257
You (2022)	Gwangju, Korea	3922	Respiratory syncytial virus -unsubtyped	0.1691	0.0473	0.5186
Chee-Sieng (2012)	Kuala Lumpur, Malaysia	10269	Respiratory syncytial virus -unsubtyped	0.6546	0.4387	0.9721
Rosalie (2021)	Amsterdam, The Netherlands	2161	Respiratory syncytial virus -unsubtyped	0.1287	0.0111	0.2463
Rosalie (2021)	Amsterdam, The Netherlands	2161	Respiratory syncytial virus -unsubtyped	0.254	0.156	0.352
Rosalie (2021)	Amsterdam, The Netherlands	2161	Respiratory syncytial virus -unsubtyped	0.1427	0.0055	0.2799
Rosalie (2021)	Amsterdam, The Netherlands	2161	Respiratory syncytial virus -unsubtyped	0.3166	0.2186	0.4146
Rosalie (2021)	Amsterdam, The Netherlands	2161	Respiratory syncytial virus -unsubtyped	0.493	0.3754	0.6106
Rosalie (2021)	Amsterdam, The Netherlands	2161	Respiratory syncytial virus -unsubtyped	0.249	0.1706	0.3274
Wei (2016)	Shanghai, China	2819	Respiratory syncytial virus -unsubtyped	0.4678	0.1358	1.4916
Keita (2023)	47 prefectures, Japan	721709	Respiratory syncytial virus -unsubtyped	1.053	1.015	1.093
Keita (2023)	47 prefectures, Japan	721709	Respiratory syncytial virus -unsubtyped	0.936	0.91	0.964
Qing (2016)	Hangzhou, China	36500	Respiratory syncytial virus -unsubtyped	0.0074	0.0038	0.0136
Jang (2017)	Cheonan, Korea	6279	Respiratory syncytial virus -A	0.8159	0.6667	0.9978
Jang (2017)	Cheonan, Korea	6279	Respiratory syncytial virus -B	0.7781	0.6357	0.9517
Terezinha (2017)	Sao Paulo, Brazil	Not reported	Respiratory syncytial virus -unsubtyped	0.2694	0.1507	0.4684
Terezinha (2017)	Sao Paulo, Brazil	Not reported	Respiratory syncytial virus -unsubtyped	0.2053	0.1128	0.3612
You (2022)	13 European countries	30965	Respiratory syncytial virus -unsubtyped	0.06	0.05	0.07
Mariana (2004)	Buenos Aires, Argentina	18561	Respiratory syncytial virus -unsubtyped	0.0006	0.0001	0.0039

Ping-Ing (2023)	Taiwan, China	1740	Respiratory syncytial virus -unsubtyped	1.4241	0.8641	2.3629
Ping-Ing (2023)	Taipei City, China	1740	Respiratory syncytial virus -unsubtyped	2.0969	1.2669	3.5199
Ping-Ing (2023)	Tainan City, China	1740	Respiratory syncytial virus -unsubtyped	3.405	2.0309	5.8431
Chan (2021)	Hong Kong, China	9635	Respiratory syncytial virus -unsubtyped	15.5187	12.2083	19.8827
Onozuka (2014)	Fukuoka, Japan	30215	Respiratory syncytial virus -unsubtyped	0.51	0.39	0.68
Benjamin (2018)	Nha Trang, central Vietnam	2998	Respiratory syncytial virus -unsubtyped	1.14	0.98	1.33
Meng (2023)	Kent Hill, Singapore	15715	Respiratory syncytial virus -unsubtyped	1.168	1.068	1.277
Meng (2023)	Kent Hill, Singapore	15715	Respiratory syncytial virus -unsubtyped	0.92	0.863	0.981
TNS (2012)	Middle Anatolian, Turkey	3464	Respiratory syncytial virus -unsubtyped	0.0288	0.003	0.1632
TNS (2012)	Mediterranean, Aegean, Marmara, Turkey	3464	Respiratory syncytial virus -unsubtyped	0.005	0.0003	0.0423
TNS (2012)	Black Sea, Turkey	3464	Respiratory syncytial virus -unsubtyped	0.0499	0.0062	0.2546
TNS (2012)	Anatolian Eastern, Turkey	3464	Respiratory syncytial virus -unsubtyped	0.0895	0.0134	0.4156
Wang (2005)	Hangzhou, China	13642	Respiratory syncytial virus -unsubtyped	0.0045	0.0003	0.0316
Geng (2015)	Suzhou, China	42664	Respiratory syncytial virus -unsubtyped	0.0036	0.0011	0.0106
Jung (2018)	Seoul, Korea	9113	Respiratory syncytial virus -unsubtyped	0.0658	0.0247	0.1567
Lin (2015)	Quanzhou, China	6020	Respiratory syncytial virus -unsubtyped	0.19	0.01	2.26
Wu (2021)	Haikou, China	309	Respiratory syncytial virus -A	0.0696	0.0124	0.2849
Wu (2021)	Haikou, China	309	Respiratory syncytial virus -B	0.0751	0.0137	0.3038
Tang (2013)	Suzhou, China	1883	Respiratory syncytial virus -unsubtyped	0.001	0.0001	0.002
Wan (2013)	Suzhou, China	28871	Respiratory syncytial virus -unsubtyped	0.0081	0.002	0.026
Chen (2016)	Suzhou, China	3672	Respiratory syncytial virus -unsubtyped	0.0086	0.0004	0.0774
Zhang (2022)	Jiangyin, China	12294	Respiratory syncytial virus -unsubtyped	0.0112	0.0025	0.0391
Ji (2011)	Suzhou, China	6655	Respiratory syncytial virus -unsubtyped	0.0012	0.0001	0.0087
Chen (2016)	Suzhou, China	3672	Parainfluenza virus -1	1.7339	0.362	9.1988
Chen (2016)	Suzhou, China	3672	Parainfluenza virus -2	2.2662	0.4746	12.5981
Chen (2016)	Suzhou, China	3672	Parainfluenza virus -3	16.3229	14.0957	18.9573
Zhang (2022)	Jiangyin, China	12294	Parainfluenza virus -3	9.4827	8.7965	10.2296
Yan (2015)	Shanghai, China	2526	Parainfluenza viruses -unsubtyped	14.9778	12.5783	17.9086

Classified as ECDC NORMAL

Zhang (2020)	Suzhou, China	7525	Parainfluenza virus -3	27.8768	24.9627	31.1838
Liu (2019)	Guangzhou, China	11398	human metapneumovirus (HMPV)	0.9436	0.4276	2.079
Nicklas (2016)	Gothenburg, Sweden	20062	human metapneumovirus (HMPV)	0.4769	0.2572	0.8668
Jean (2009)	Rheinland-Pfalz, Mainz	3044	human metapneumovirus (HMPV)	0.0975	0.0594	0.1556
Cheng (2022)	Macao, China	4880	human metapneumovirus (HMPV)	0.7048	0.5842	0.8495
Chen (2014)	Suzhou, China	998	human metapneumovirus (HMPV)	0.1374	0.0185	0.7074
Rory (2019)	Edinburgh, UK	52060	human metapneumovirus (HMPV)	0.901	0.883	0.92
Maria (2009)	Kathmandu, Nepal	887	human metapneumovirus (HMPV)	0.5767	0.1591	1.9639
Magali (2018)	Dijon, France	4300	human metapneumovirus (HMPV)	0.6693	0.6	0.7464
Yong (2019)	Seoul, Korea	23694	human metapneumovirus (HMPV)	0.7913	0.5779	1.0816
Yong (2019)	Seoul, Korea	23694	human metapneumovirus (HMPV)	0.9123	0.667	1.247
Yong (2019)	Seoul, Korea	23694	human metapneumovirus (HMPV)	1.2871	0.9415	1.7627
Li (2013)	Zhuhai, China	924	human metapneumovirus (HMPV)	0.2556	0.0125	2.8583
Wang (2013)	Suzhou, China	6655	human metapneumovirus (HMPV)	0.1996	0.0574	0.6038
You (2022)	Gwangju, Korea	4195	human metapneumovirus (HMPV)	0.3486	0.1087	1.0205
Wang (2011)	Suzhou, China	6599	human metapneumovirus (HMPV)	0.1777	0.0501	0.5427
Ji (2011)	Suzhou, China	6655	human metapneumovirus (HMPV)	0.1777	0.0501	0.5427
You (2022)	Gwangju, Korea	4195	Human coronavirus (HCoV) -unsubtyped	0.03	0.01	0.13
Jean (2009)	Rheinland-Pfalz, Mainz	3044	Human coronavirus (HCoV) -unsubtyped	0.06	0.04	0.1
Jang (2020)	Cheonan, Korea	9010	Human coronavirus (HCoV)- 229E	0.4	0.28	0.59
Jang (2021)	Cheonan, Korea	9010	Human coronavirus (HCoV)- OC43	0.54	0.42	0.7
Nicklas (2016)	Gothenburg, Sweden	20062	Human coronavirus (HCoV) -unsubtyped	0.04	0.02	0.09
Cui (2015)	Chaoshan, China	1074	Human coronavirus (HCoV) -unsubtyped	1.1	1	1.2
Wei (2016)	Shanghai, China	2819	Human coronavirus (HCoV) -unsubtyped	0.68	0.2	2.17
Jean (2009)	Rheinland-Pfalz, Mainz	3044	Human rhinovirus	0.1866	0.1181	0.2886
Giselmo (2020)	Maranhao, Brazil	151	Human rhinovirus	5.3594	0.4718	127.9191
Giselmo (2020)	Maranhao, Brazil	151	Human rhinovirus	2.2662	0.2005	36.7868
Cui (2015)	Chaoshan, China	1074	Human rhinovirus	1	0.9	1.1

Classified as ECDC NORMAL

Tiina (2016)	Kajaani, Finland	386	Human rhinovirus	0.96	0.92	1
Tiina (2016)	Kajaani, Finland	386	Human rhinovirus	1.04	0.98	1.1
Yan (2017)	Suzhou, China	6194	Human rhinovirus	10.5699	2.6955	54.6864
Jaqueline (2016)	Curitiba, Brazil	755	Human rhinovirus	0.0503	0.0048	0.3027
You (2022)	Gwangju, Korea	4195	Human rhinovirus	0.7048	0.2358	2.0445
Dong (2021)	Cheonan, Korea	1920	Human rhinovirus	1.575	1.443	1.72
Benjamin (2018)	Nha Trang, Vietnam	2998	Human rhinovirus	0.86	0.79	0.94
Zhou 2018	Suzhou, China	5994	Human rhinovirus	4.1649	3.7751	4.5991
Zhou 2017	Huangshi, China	2326	Human rhinovirus	26.184	21.5272	32.0206
Huang 2015	Suzhou, China	1926	Human rhinovirus	32.3742	25.9496	40.6707
Zheng (2014)	Jiangsu, China	42104	human adenoviruses (HAdVs)	7.9418	3.9794	16.8532
Nicklas (2016)	Gothenburg, Sweden	20062	human adenoviruses (HAdVs)	0.5554	0.3016	0.993
Jean (2009)	Rheinland-Pfalz, Mainz	3044	human adenoviruses (HAdVs)	0.1866	0.1181	0.2886
Cheng (2022)	Macao, China	4880	human adenoviruses (HAdVs)	1.1355	0.9425	1.3684
Chen (2014)	Suzhou, China	998	human adenoviruses (HAdVs)	1.1563	0.2365	5.8082
Rory (2019)	Edinburgh, UK	52060	human adenoviruses (HAdVs)	0.972	0.955	0.988
Therese (2021)	Nairobi, Kenya	17261	human adenoviruses (HAdVs)	1.06	0.76	1.47
Therese (2021)	Nairobi, Kenya	17261	human adenoviruses (HAdVs)	0.99	0.7	1.41
Li (2013)	Zhuhai, China	924	human adenoviruses (HAdVs)	34.668	25.1629	48.4754
You (2022)	Gwangju, Korea	4195	human adenoviruses (HAdVs)	0.6215	0.2059	1.8007
Mariana (2004)	Buenos Aires, Argentina	18561	human adenoviruses (HAdVs)	0.1958	0.0652	0.5264
Eun (2021)	Cheonan, Korea	9010	human adenoviruses (HAdVs)	0.772	0.679	0.878
Ji (2011)	Suzhou, China	6655	human adenoviruses (HAdVs)	3.8783	1.3047	12.9526
Lin (2015)	Quanzhou, China	6020	human adenoviruses (HAdVs)	1.48	0.12	20.97
Chen (2012)	Suzhou, China	8197	human adenoviruses (HAdVs)	4.6479	1.7406	13.7737
Chen (2016)	Suzhou, China	3672	human adenoviruses (HAdVs)	3.5087	3.1008	3.9754
Zhang (2022)	Jiangyin, China	12294	human adenoviruses (HAdVs)	2.721	1.142	6.8566
You (2022)	Gwangju, Korea	4195	Enterovirus (EV)	4.3778	1.4613	14.8906

Classified as ECDC NORMAL

Cui (2015)	Chaoshan, China	1074	Enterovirus (EV)	2.4	2.1	2.7
Jean (2009)	Rheinland-Pfalz, Mainz	3044	Enterovirus (EV)	0.7753	0.5156	1.162
Wei (2016)	Shanghai, China	2819	Enterovirus (EV)	1.6702	0.5244	5.6051
Shiv (2020)	Fifty U.S. states and Washington D.C.	974	SARS-CoV-2	0.85	0.76	0.96
Shiv (2020)	Fifty U.S. states and Washington D.C.	974	SARS-CoV-2	0.98	0.97	0.99
Aleix (2021)	52 Spanish provinces	2418250	SARS-CoV-2	0.08	0.07	0.09
Aleix (2021)	52 Spanish provinces	2418250	SARS-CoV-2	0.07	0.06	0.08
Aleix (2021)	52 Spanish provinces	2418250	SARS-CoV-2	0.1	0.09	0.11
Keita (2022)	Six Japanese prefectures	2982262	SARS-CoV-2	1	0.98	1.02
Keita (2022)	Six Japanese prefectures	2982262	SARS-CoV-2	0.99	0.99	1
Keita (2022)	Six Japanese prefectures	2982262	SARS-CoV-2	0.99	0.98	1
Keita (2022)	Six Japanese prefectures	2982262	SARS-CoV-2	0.98	0.97	0.99
Keita (2022)	Six Japanese prefectures	2982262	SARS-CoV-2	1.11	1.05	1.17
Keita (2022)	Six Japanese prefectures	2982262	SARS-CoV-2	0.99	0.98	0.99
Keita (2022)	Six Japanese prefectures	2982262	SARS-CoV-2	0.97	0.95	1
Keita (2022)	Six Japanese prefectures	2982262	SARS-CoV-2	0.97	0.92	1.02
Chen (2021)	Guangdong, China	1347	SARS-CoV-2	0.94	0.93	0.96
Sung (2022)	Changwon, Korea	3234	SARS-CoV-2	0.99	0.98	0.99
Lisa (2022)	Helsinki, Finland	48013	SARS-CoV-2	3.95	2.88	5.42
Kong(2020)	Wuhan, China	Not reported	SARS-CoV-2	10.25	1.76	96.61
Simone (2020)	Milan, Italy	Not reported	SARS-CoV-2	0.26	0.12	0.54
Simone (2020)	Milan, Italy	Not reported	SARS-CoV-2	0.25	0.11	0.52
Simone (2020)	Milan, Italy	Not reported	SARS-CoV-2	0.24	0.11	0.5
Simone (2020)	Florence, Italy	Not reported	SARS-CoV-2	0.35	0.16	0.72
Simone (2020)	Florence, Italy	Not reported	SARS-CoV-2	0.27	0.12	0.56
Simone (2020)	Florence, Italy	Not reported	SARS-CoV-2	0.25	0.11	0.52
Simone (2020)	Trento, Italy	Not reported	SARS-CoV-2	0.22	0.1	0.45
Simone (2020)	Trento, Italy	Not reported	SARS-CoV-2	0.25	0.11	0.52

Classified as ECDC NORMAL

Simone (2020)	Trento, Italy	Not reported	SARS-CoV-2	0.26	0.12	0.54
Asmaa (2020)	Riyadh, Saudi Arabia	Not reported	MERS-CoV	1.054	1.043	1.065
Cheng (2022)	Macao, China	4880	Human rhinoviruses/enteroviruses (HRVs/EVs)	1.2035	0.9989	1.4508
Raymundo (2023)	Oahu, USA	7143	Human rhinoviruses/enteroviruses (HRVs/EVs)	1.16	0.97	1.39
Bi (2006)	Hong Kong, China	2142	SARS-CoV	0.01	0.0006	0.07
Bi (2006)	Beijing, China	2142	SARS-CoV	0.2	0.04	0.83
Bi (2006)	Hong Kong, China	2142	SARS-CoV	0.01	0.0012	0.09
Feng (2005)	Guangzhou, China	138	SARS-CoV	0.79	0.43	1.46
Feng (2005)	Hong Kong, China	108	SARS-CoV	1.53	0.76	3.11
Feng (2005)	Toronto, Canada	116	SARS-CoV	0.73	0.37	1.42
Feng (2005)	Singapore	72	SARS-CoV	1.03	0.43	2.44
Feng (2005)	Taiwan, China	106	SARS-CoV	0.78	0.38	1.56
Feng (2005)	Beijing, China	41	SARS-CoV	1.02	0.32	3.31
Liu (2021)	Moscow	Not reported	SARS-CoV	4.65	2.2	10.41
Liu (2021)	Moscow	Not reported	SARS-CoV	3.71	1.78	8.15
Liu (2021)	Moscow	Not reported	SARS-CoV	4.65	2.2	10.41
Huang (2004)	Huangping, China	1491	SARS-CoV	0.56	0.29	1.05
Huang (2004)	Huangping, China	1491	SARS-CoV	0.52	0.27	0.99
Huang (2004)	Huangping, China	1491	SARS-CoV	0.59	0.31	1.12
Susana (2012)	Valencia, Spain	243	Streptococcus pneumoniae	0.2814	0.0853	0.8321
Cristiana (2010)	Salvador, Brazil	184	Streptococcus pneumoniae	0.2053	0.1048	0.3919
Katerina (2019)	Liverpool, UK	374	Streptococcus pneumoniae	0.79	0.63	0.99
Onozuka (2009)	Fukuoka, Japan	13056	Streptococcus pneumoniae	1.17	1.11	1.23
Daniel (2015)	Mallorca, Spain	60659	Streptococcal pharyngitis	0.4588	0.3016	0.6911
Huang (2019)	Zhongshan, China	13705	Moraxella catarrhalis	0.0687	0.0073	0.3909
Sun (2014)	Suzhou, China	8143	Moraxella catarrhalis	5.628	2.0781	16.3229
PSOTER (2016)	48 states of USA	3463	Pseudomonas aeruginosa	1.04	1.03	1.05
Susana (2012)	Valencia, Spain	243	Legionella pneumophila	2.4518	1.1877	7.7123

Classified as ECDC NORMAL

Lin (2015)	Quanzhou, China	6020	Legionella pneumophila	6.15	0.53	149.9
Huang (2022)	Suzhou, China	7940	Haemophilus influenzae	0.4276	0.1367	1.2438
Jean (2009)	Rheinland-Pfalz, Mainz	3044	Mycoplasma pneumoniae	0.4956	0.3265	0.7456
Chen (2020)	Suzhou, China	998	Mycoplasma pneumoniae	22.2606	17.6757	28.2451
Chen (2013)	Suzhou, China	1598	Mycoplasma pneumoniae	180.7475	146.4838	275.1224
Chen (2013)	Suzhou, China	8157	Mycoplasma pneumoniae	5.5715	2.0588	16.9388
Huang (2019)	Zhongshan, China	13705	Mycoplasma pneumoniae	180.7475	179.2791	222.2747
Tian (2017)	Hangzhou, China	3769	Mycoplasma pneumoniae	4.0363	2.7227	6.0729
Tian (2017)	Hangzhou, China	3769	Mycoplasma pneumoniae	4.2408	2.8561	6.3941
Tian (2017)	Hangzhou, China	3769	Mycoplasma pneumoniae	3.539	2.3968	5.2961
Na (2022)	Nantong, China	51665	Mycoplasma pneumoniae	3.0394	1.6853	5.6372
Wang (2017)	Suzhou, China	15098	Mycoplasma pneumoniae	3.6796	1.7246	8.2816
Lei (2021)	Chengdu, China	22882	Mycoplasma pneumoniae	1.696	0.7718	3.8203
Onozuka (2008)	Fukuoka, Japan	13056	Mycoplasma pneumoniae	1.17	1.11	1.23
Yuan (2022)	Chendu, China	5127	Mycoplasma pneumoniae	1.68	1.32	2.13
Lin (2015)	Quanzhou, China	6020	Mycoplasma pneumoniae	9.31	0.77	274.79
Wang (2013)	Guilin, China	1342	Mycoplasma pneumoniae	19.2665	15.0558	24.8649
Wu (2019)	Wu zhishan, China	1597	Mycoplasma pneumoniae	39.5804	30.8471	51.2382
Na (2021)	Tianjin, China	63821	Mycoplasma pneumoniae	0.046	0.0098	1.0362
Ni (2014)	Suzhou, China	10596	Mycoplasma pneumoniae	8.1661	3.1915	23.4988
Yin (2013)	Luoyang, China	8368	Mycoplasma pneumoniae	9.5957	2.9793	37.5377
Zheng (2013)	Zhengzhou, China	256	Mycoplasma pneumoniae	0.00001	0.00001	0.0022
Shi (2016)	Suzhou, China	2323	Mycoplasma pneumoniae	0.0807	0.015	0.3235
Huang (2019)	Zhongshan, China	13705	Chlamydia pneumoniae	180.7475	179.2791	222.2747
Chen (2013)	Suzhou, China	1598	Chlamydia pneumoniae	0.9266	0.0717	11.5859
Jean (2009)	Rheinland-Pfalz, Mainz	3044	Chlamydia pneumoniae	0.8968	0.5975	1.3444
Lin (2015)	Quanzhou, China	6020	Chlamydia pneumoniae	1.59	0.16	22.87

The code is as follows:

Classified as ECDC NORMAL

```
setwd(dir="C:/Users/86183/Desktop/data.csv")
getwd()
bin.metagen <- read.csv("C:/Users/86183/Desktop/data.csv")
head(bin.metagen,150)
View(bin.metagen)
library("meta")
library(base)
bin.metagen$RR <- log(bin.metagen$RR)
bin.metagen$lower <- log(bin.metagen$lower)
bin.metagen$upper <- log(bin.metagen$upper)
bin.metagen$seTE <- (bin.metagen$upper - bin.metagen$lower)/3.92
fit<-metagen(TE=RR,
            seTE=seTE,
            studlab = paste(Study), method.tau="REML",
            sm = "RR", data = bin.metagen,byvar = Suboutcome,print.byvar = FALSE,
            comb.random = TRUE,
            comb.fixed = FALSE, prediction= TRUE, prediction.subgroup = TRUE,
            label.left ="Reduced risk",
            label.right ="Increased risk")
forest(fit,col.diamond ="skyblue",col.square="maroon",col.diamond.lines ="black", sortvar = TE,
      base_family = "Times New Roman", family="sans",fontsize=11,
      col.predict = "#FF0000",colgap.studlab = "4cm", colgap.forest.left = "5.0cm",
      leftlabs = c("Study"))
forest(fit,col.diamond = "maroon",col.square="skyblue",col.diamond.lines = "black",sortvar = TE,
      family="mono",fontsize=11,colgap.studlab = "4cm", colgap.forest.left = "5.0cm",
```


Classified as ECDC NORMAL

```
      base_family = "Times New Roman",col.predict = "#FF0000",
      leftlabs = c("Study"))
```

Subgroup analysis

```
fit<-metagen(TE=RR,
           seTE=seTE,
           studlab = paste(Study),
           method.tau="REML",
           sm = "RR",
           data = bin.metagen,byvar = Climate/Income/Resolution/Measurement/Model/Lag,
           comb.random = TRUE,
           comb.fixed = FALSE, prediction.subgroup = TRUE, prediction= TRUE,
           label.left ="Reduced risk",
           label.right ="Increased risk")
forest(fit,col.diamond ="skyblue",col.square="maroon",col.diamond.lines ="black",
      base_family = "Times New Roman",colgap.studlab = "4cm", colgap.forest.left = "5.0cm",
      family="sans",fontsize=11, col.predict = "#FF0000",
      leftlabs = c("Study"))
forest(fit,col.diamond ="maroon",col.square="skyblue",col.diamond.lines ="black",
      base_family = "Times New Roman",
      family="sans",fontsize=11, col.predict = "#FF0000",colgap.studlab = "4cm",colgap.forest.left = "5.0cm",
      leftlabs = c("Study"))
```

Sensitivity analysis

```
fit<-metagen(TE=RR,
```


Classified as ECDC NORMAL

```
            seTE=seTE,
            studlab = paste(Study),
            method.tau="REML",
            sm = "RR",
            data = bin.metagen,
            comb.random = TRUE,
            comb.fixed = FALSE,
            label.left ="Reduced risk",
            label.right ="Increased risk")
forest(metainf(fit,pooled="random"),
       comb.random=TRUE,family="sans",base_family = "Arial",refline_col = "#004CFFFF",
       fontsize=11,lwd=2,col.diamond.fixed="#4D9221",col.diamond.lines.fixed="#4D9221",
       col.diamond.random="#FF0000",col.diamond.lines.random="maroon",
       col.square="#00FF00",col.study="#C51B7D",lty.fixed=4,
       plotwidth="8cm",colgap.forest.left="1cm",
       colgap.forest.right="1cm",just.forest="right",colgap.left="0.5cm",
       colgap.right="0.5cm")

funnel(fit,
       contour.levels = c (0.9,0.95,0.99),studlab = TRUE,
       col.contour = c('darkgray', 'gray', 'lightgray'))
legend(1,0.02, c("0.1 > p > 0.05", "0.05 > p >0.01", "< 0.01"), fill=c("white", "gray", "darkgray"))
par(mfrow=c(2,2))
dev.off()
```


Classified as ECDC NORMAL

```
tf.publ<-trimfill(fit)
summary(tf.publ)
funnel (tf.publ)
dev.off()

metabias(fit,method="linreg")
```


Classified as ECDC NORMAL
